# Two-Sample Mendelian Randomization Analysis Investigates Causal Associations Between Gut Microbial Genera and Inflammatory Bowel Disease, and Specificity Causal Associations in Ulcerative Colitis or Crohn’s Disease

**DOI:** 10.3389/fimmu.2022.921546

**Published:** 2022-07-04

**Authors:** Bin Liu, Ding Ye, Hong Yang, Jie Song, Xiaohui Sun, Yingying Mao, Zhixing He

**Affiliations:** ^1^ Department of Epidemiology, School of Public Health, Zhejiang Chinese Medical University, Hangzhou, China; ^2^ Institute of Basic Research in Clinical Medicine, School of Basic Medical Science, Zhejiang Chinese Medical University, Hangzhou, China

**Keywords:** inflammatory bowel disease, gut microbial genera, Mendelian randomization, ulcerative colitis, Crohn’s disease

## Abstract

**Background:**

Intestinal dysbiosis is associated with inflammatory bowel disease (IBD). Ulcerative colitis (UC) and Crohn’s disease (CD), two subtypes of IBD, are characterized by unique microbial signatures, respectively. However, it is unclear whether UC or CD has a specific causal relationship with gut microbiota.

**Objective:**

To investigate the potential causal associations between gut microbial genera and IBD, UC, or CD, two-sample Mendelian randomization (MR) analyses were conducted.

**Materials and Methods:**

We obtained genome-wide association study (GWAS) summary statistics of gut microbiota and IBD, UC, or CD from published GWASs. Two-sample MR analyses were performed to identify potential causal gut microbial genera for IBD, UC, and CD using the inverse-variance weighted (IVW) method. Sensitivity analyses were also conducted to validate the robustness of the primary results of the MR analyses. Finally, a reverse MR analysis was performed to evaluate the possibility of reverse causation.

**Results:**

Combining the results from the primary and sensitivity analyses, six bacterial genera were associated with the risk of IBD, UC, or CD in the IVW method. Briefly, *Eubacterium ventriosum* group was associated with a lower risk of IBD (*P*=0.011) and UC (*P*=1.00×10^-4^), whereas *Coprococcus* 2 was associated with a higher risk of IBD (*P*=0.022) and UC (*P*=0.007). In addition, we found a positive association between *Oxalobacter* with IBD (*P*=0.001) and CD (*P*=0.002), and *Ruminococcaceae* UCG014 with IBD (*P*=0.005) and CD (*P*=0.007). We also noticed a negative association between *Enterorhabdus* (*P*=0.044) and IBD, and between *Lachnospiraceae* UCG001 (*P*=0.023) and CD. We did not find causal effects of IBD, UC, or CD on these bacterial genera in the reverse MR analysis.

**Conclusion:**

This study expanded gut microbial genera that were causally associated with the risk of IBD, and also revealed specificity-gut microbial genera for UC or CD.

## Introduction

Inflammatory bowel disease (IBD) is a chronic and life-threatening inflammatory disease of gastroenteric tissue (1). The main symptoms of IBD contain diarrhea, abdominal pain, rectal bleeding, and weight loss (2). As a lifelong disease, IBD occurs early in life among both males and females, which causes high morbidity and mortality worldwide (3). The risk of IBD is mainly attributed to the interaction between genetic factors and gut microbiota which influence the immune responses (4, 5). Therefore, documentation of host genetic SNPs-gut microbiota interaction may play an important role in the pathogenesis of IBD.

Recently, some studies have reported the causal relationship between gut microbiota and IBD using the two-sample Mendelian randomization (MR) analysis (6, 7). The MR analysis successfully identified that the genus *Akkermansia* and *Dorea* were causally associated with the risk of IBD (6). In addition, some clinical observational studies drew inconsistent results related to some microbial genera in patients with IBDs, such as *Bacteroides*, *Akkermansia*, *Bifidobacterium*, and *Lactobacillus* (8). However, these studies did not reveal the differences in the relationship between gut microbial genera and ulcerative colitis (UC) or Crohn’s disease (CD), which are the two main subtypes of IBD. The varying affected areas of the digestive tract was the main differences between UC and CD. CD discontinuously affects the terminal ileum, cecum, perianal area, and colon, while UC usually involves the rectum and continuously affects part or the entire colon (9–11). Not limited to that, UC and CD could also be distinguished by gut microbial genera or host genetic loci (12, 13). Therefore, it is necessary to reveal the differences in the potential causal relationships of gut microbial genera with UC and CD.

Mendelian randomization approach could examine the potential causal association from exposure to outcome using instrumental variables (IVs). Recently, MR analysis has been applied to investigate relationships between gut microbiota and many diseases (14–16). However, results of MR analysis mainly depend on the selection of the GWAS database and the filtering of instrumental variables. This study conducted an MR analysis using the most up-to-date GWAS databases from a previous study (17) to investigate the potential causal associations of gut microbial genera with the risk of IBD, UC, and CD.

## Materials and Methods

### Study Design

**Figure 1:**
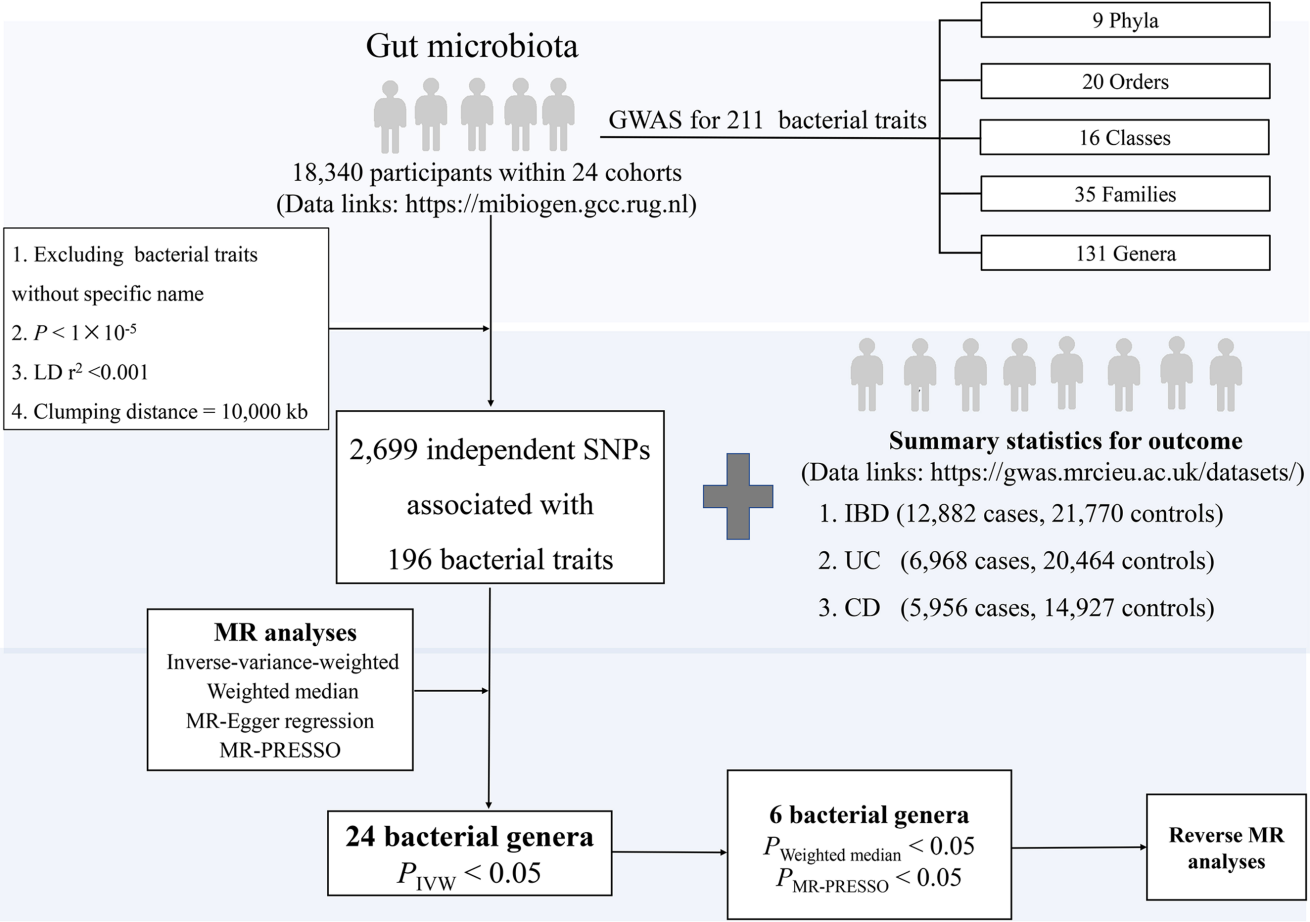
The study design of the present MR study of the associations of gut microbiota and inflammatory bowel disease. Abbreviations: CD, Crohn’s disease; LD, linkage disequilibrium, which used to measure the correlations between SNPs; IBD, inflammatory bowel disease; IVW, Inverse-variance-weighted, the main analyses to evaluate the relationship between exposure and outcome; MR-PRESSO, Mendelian Randomization Pleiotropy RESidual Sum and Outlier, a method test the pleiotropic biases in the SNPs and correct the pleiotropic effects; MR, Mendelian randomization; SNP, single nucleotide polymorphism, as instrumental variables for the exposures and outcomes; UC, ulcerative colitis.

The overall study design is presented in **Figure 1**. In particular, we investigated the associations of gut microbial genera and the risk of IBD, UC, and CD using a two-sample MR method. In order to reduce the influence of bias on the results, there are three key assumptions we tried to satisfy when we used the MR approach. First, the IVs are significantly associated with gut microbiota (18). Second, the IVs are independent which means they are not associated with other confounding factors (18). Finally, in addition to exposure factors, the IVs should not affect the outcome through other pathways (18).

### Data Sources and Instruments

#### Human Gut Microbiome

For human gut microbiota composition, summary-level data were drawn from a GWAS meta-analysis (data link: https://mibiogen.gcc.rug.nl) within 24 population-based cohorts (N=18,340 participants) from Europe, North America, and East Asia (17). First, we excluded the 15 bacterial traits without specific species name (unknown family or genus), leaving 196 bacterial traits, including five biological classifications: phylum, class, order, family, and genus. Second, we selected IVs at *P*<1×10^-5^ to obtain a more comprehensive result. Third, to reduce the influence of correlations between SNPs [i.e., linkage disequilibrium (LD)], we performed LD-clumping for all the IVs (r^2^ <0.001, distance=10,000 kb) and retained SNPs with the lowest *P*-value for the exposure of interest, resulting in 2699 independent SNPs associated with 196 bacterial traits. Since 30 SNPs were not available in the outcome dataset, 2669 SNPs associated with 196 bacterial traits were included in the MR analyses.

#### IBD, UC, and CD

Briefly, summary statistics for IBD were obtained from a GWAS meta-analysis (data link: https://gwas.mrcieu.ac.uk/datasets/) including 12,882 cases and 21,770 controls with a total of 11,555,662 SNPs (19). The genetic association data consisted of 27,432 participants (N=6968 cases, 20,464 controls) with UC and 20,883 participants (N=5956 cases, 14,927 controls) with CD, covering a total of 11,113,951 SNPs in UC and 11,002,658 SNPs in CD, respectively (19). All cases and controls were Europeans and the diagnosis of patients meets the accepted radiological, endoscopic, and histopathological evaluation (19). In the reverse MR analysis, a number of quality control steps were performed to obtain eligible IVs to meet the three assumptions of MR, with details described elsewhere (20). Briefly, a stricter threshold was used to select IVs, where the significance threshold was set to *P*< 5×10^−8^ (**Supplemental Table S1**). No additional ethical approval or consent to participate was required because we used published studies and public summary statistics.

#### Statistical Analysis

First, the inverse-variance-weighted (IVW) method was used as the main MR analysis to evaluate the relationships between gut microbial genera and IBD, UC, or CD, which combined Wald estimator from SNP to get the estimates of the effect (21). The result of IVW method would be credible if each SNP satisfies the assumptions of MR (no horizontal pleiotropy) (21). In order to test the heterogeneity of each SNP, we performed Cochran’s Q test. A random-effects IVW model was used if significant heterogeneity (*P*<0.05) was observed, otherwise, a fixed-effects IVW model was applied (22). To assess the robustness of our results, we further performed sensitivity analyses, including the weighted-median method, MR-Egger regression, and MR pleiotropy residual sum and outlier (MR-PRESSO) test. In particular, the weighted median estimator provided valid causal effect estimates when less than 50% of information comes from invalid instruments (23). The *P*-value of the intercept term can be used as an indicator of directional pleiotropy (*P*<0.05 were considered statistically significant) in MR-Egger regression (24). As for MR-PRESSO test, it was performed to test the pleiotropic biases and corrected the pleiotropic effects by removing the outliers. Finally, reverse MR analysis was performed to examine whether a reverse causal association existed between IBD (UC, CD) and gut microbiota.

To examine whether the effect estimates of the causal associations were likely to be affected by weak instrument bias, the strength of IVs was tested using F statistics. F statistics were calculated using the following equation: F=R^2^(n-k-1)/k(1-R^2^), in which R^2^ represents the variance explained by the IVs (each gut microbiome) and n represents the sample size (25). R^2^ was estimated by minor allele frequency (MAF) and β value, using the equation: R^2^ = 2 × MAF × (1−MAF) × β^2^ (26).

If the result of all MR analyses reached a nominal significance, we considered the gut microbial genera were potentially associated with the risk of IBD, UC, or CD. Then, the reverse-direction MR analysis would be performed. All MR analyses were performed in R (version 3.6.3) using the “Mendelian Randomization” and “MR-PRESSO” packages.

## Results

### Overview

**Figure 2:**
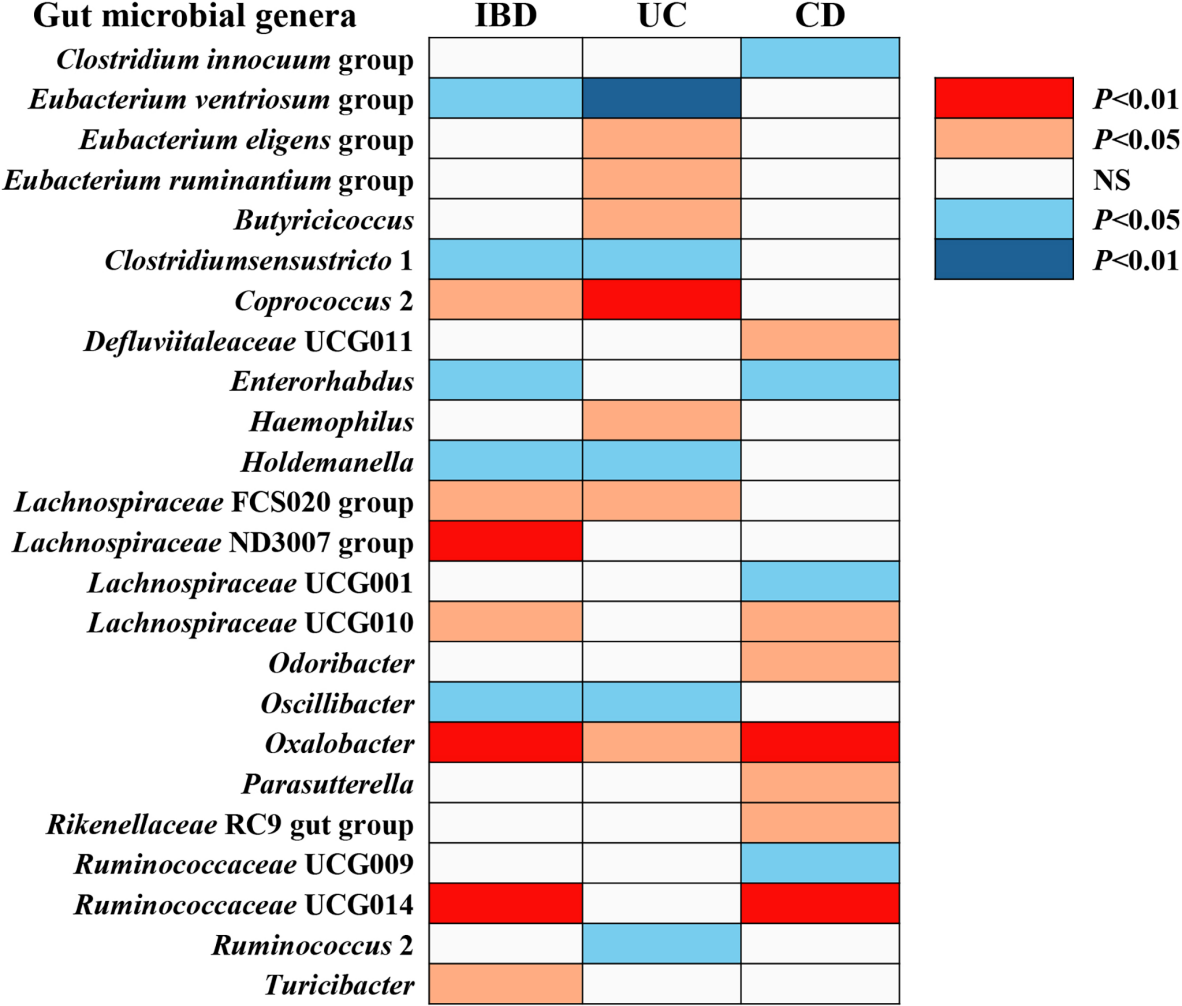
The causal effect of gut microbial genera on IBD, UC, and CD identified at the nominal significance (*P* < 0.05/0.01). Shown are the results derived from the IVW method. Red represents the risk genera for IBD, blue represents the protective genera for IBD, and white represents no causal genera for IBD. Abbreviations: CD, Crohn’s disease; IBD, inflammatory bowel disease; UC, ulcerative colitis; NS, no significant association.

The F-statistic for the human gut microbiota ranged from 21.63 to 144.84, all meeting the threshold of >10, suggesting that it was less likely to suffer from weak instrument bias. The results of the associations between 196 bacterial traits and the risk of IBD, UC, or CD are presented in **Supplemental Tables S2-S4** respectively. Briefly, we identified 24 bacterial genera associated with the risk of IBD, UC, or CD (**Table 1** and **Figure 2**). However, sensitivity analyses only supported six microbial genera which remained stable for IBD, UC, and CD. The details of IVs used are listed in **Supplemental Table S5.**

**Table 1:** Summary of causal association between gut microbial genera and the risk of IBD, UC, or CD by using the IVW method.

Human gut microbiota	N	Traits 1	Traits 2	OR	95%CI	*P*-value
*Clostridium innocuum* group	11	CD	\	0.87	0.76-1.00	0.046
*Eubacterium ventriosum* group	17	UC	IBD	0.68	0.56-0.83	1.00×10^-4^
*Eubacterium eligens* group	11	UC	\	1.26	1.00-1.58	0.047
*Eubacterium ruminantium* group	19	UC	\	1.14	1.00-1.30	0.043
*Butyricicoccus*	9	UC	\	1.31	1.04-1.64	0.020
*Clostridium sensustricto* 1	9	IBD	UC	0.79	0.66-0.97	0.009
*Coprococcus* 2	12	UC	IBD	1.32	1.08-1.62	0.006
*Defluviitaleaceae* UCG011	11	CD	\	1.25	1.03–1.50	0.023
*Enterorhabdus*	9	CD	IBD	0.82	0.68-0.98	0.032
*Haemophilus*	14	UC	\	1.17	1.01-1.36	0.033
*Holdemanella*	14	IBD	UC	0.87	0.77-0.97	0.014
*Lachnospiraceae* FCS020 group	17	IBD	UC	1.17	1.02-1.34	0.026
*Lachnospiraceae* ND3007 group	4	IBD	\	1.62	1.13-2.32	0.008
*Lachnospiraceae* UCG001	15	CD	\	0.81	0.67-0.97	0.023
*Lachnospiraceae* UCG010	13	IBD	CD	1.22	1.05-1.43	0.012
*Odoribacter*	9	CD	\	1.38	1.04-1.83	0.023
*Oscillibacter*	16	UC	IBD	0.84	0.72-0.97	0.018
*Oxalobacter*	12	IBD	UC, CD	1.17	1.07-1.29	0.001
*Parasutterella*	17	CD	\	1.22	1.03-1.45	0.023
*Rikenellaceae* RC9 gut group	15	CD	\	1.15	1.03-1.28	0.011
*Ruminococcaceae* UCG009	13	CD	\	0.81	0.69-0.96	0.014
*Ruminococcaceae* UCG014	17	IBD	CD	1.23	1.06-1.42	0.005
*Ruminococcus* 2	15	UC	\	0.81	0.67-0.99	0.039
*Turicibacter*	14	IBD	\	1.15	1.01-1.31	0.033

If microbiota is significant across different phenotypes, we only present the association with smallest P-values. CD, Crohn’s disease; CI, confidence interval; IBD, inflammatory bowel disease; IVW, inverse-variance weighted; OR, odds ratio; UC, ulcerative colitis.

### IBD

**Figure 3:**
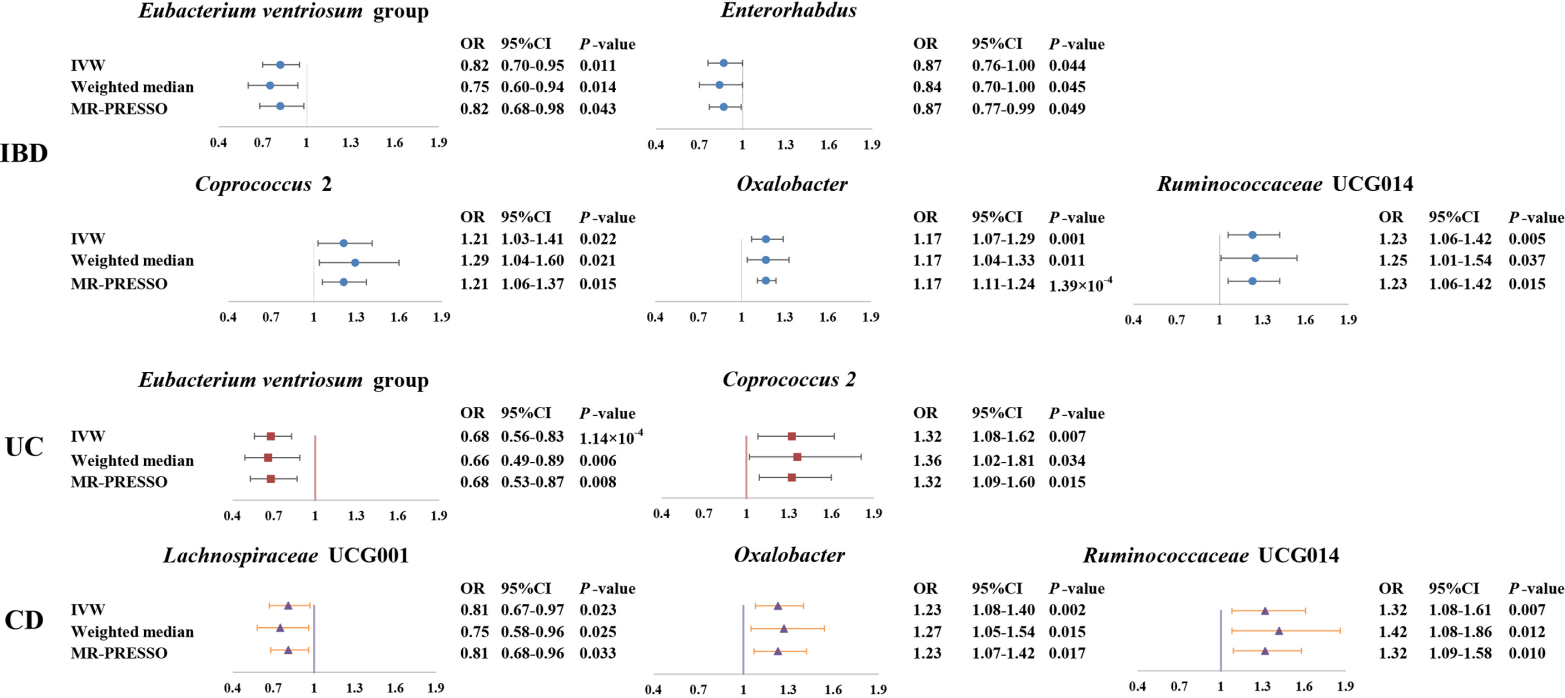
Forest plot of the associations between genetically determined six gut microbial genera with the risks of IBD, UC, or CD. Presented are the gut microbiota genera that were statistically significant across all analyses (IVW, weighted median, MR-PROSSE). Abbreviations: CD, Crohn’s disease; CI, confidence interval; IBD, inflammatory bowel disease; IVW, inverse-variance weighted; MR-PRESSO, Mendelian Randomization Pleiotropy RESidual Sum and Outlier; OR, odds ratio; UC, ulcerative colitis.

Overall, in primary MR analyses, 12 bacterial genera were statistically associated with the risk of IBD, suggesting these bacterial genera might impose effects on the development of IBD (**Table 1** and **Figure 2**). However, only five microbial genera remained stable in other sensitivity methods (**Figure 3**).

As shown in **Figure 3**, we observed that genus *Coprococcus* 2, *Oxalobacter* and *Ruminococcaceae* UCG014 were associated with a higher risk of IBD [odds ratio (OR): 1.21, 95% confidence interval (CI): 1.03-1.41, *P*=0.022 for *Coprococcus* 2; OR: 1.17, 95% CI: 1.07-1.29, *P*=0.001 for *Oxalobacter;* and OR: 1.23, 95% CI=1.06-1.42, *P*=0.005 for *Ruminococcaceae* UCG014], whereas genus *Eubacterium ventriosum* group and *Enterorhabdus* were associated with a lower risk of IBD (OR: 0.82, 95% CI: 0.70-0.95, *P*=0.011 for *Eubacterium ventriosum* group and OR: 0.87, 95% CI: 0.76-1.00, *P*=0.044 for *Enterorhabdus*).

In sensitivity analyses, the weighted median method produced similar estimates (OR: 1.29, 95% CI: 1.04-1.61, *P*=0.021 for *Coprococcus* 2; OR: 1.17, 95% CI: 1.04-1.33, *P*=0.011 for *Oxalobacter*; OR:1.25, 95% CI: 1.01-1.54, *P*=0.037 for *Ruminococcaceae* UCG014; OR: 0.75, 95% CI: 0.60-0.94, *P*=0.014 for *Eubacterium ventriosum* group; OR: 0.84, 95% CI: 0.70-1.00, *P*=0.045 for *Enterorhabdus*), though with wider CIs. Furthermore, little evidence of directional pleiotropy was found for these microbial genera in MR-Egger regression (intercept *P*=0.477 for *Coprococcus* 2; intercept *P*=0.620 for *Oxalobacter*; intercept *P*=0.445 for *Ruminococcaceae* UCG014; intercept *P*=0.869 for *Eubacterium ventriosum* group; intercept *P*=0.132 for *Enterorhabdus*), and no outliers were detected with the MR-PRESSO test (**Supplemental Table S2**).

### UC

We identified a total of 12 bacterial traits associated with UC and seven of them were also associated with IBD in IVW method (**Table 1** and **Figure 2**). In the sensitivity analyses, the results remained stable for *Eubacterium ventriosum* and *Coprococcus* 2 (**Figure 3**).

We found a negative association between genus *Eubacterium ventriosum* group and UC in the IVW method (OR: 0.68, 95% CI=0.56-0.83, *P*=1.00×10^-4^). In sensitivity analyses, the association was similar in the weighted median method (OR: 0.66, 95% CI: 0.49-0.89, *P*=0.006) and MR-PRESSO test (OR: 0.68, 95% CI: 0.53-0.87, *P*=0.008) (**Figure 3**). The MR-Egger regression did not suggest evidence of directional pleiotropy (intercept *P*=0.972) (**Supplemental Table S3**).

On the contrary, genetically predicted genus *Coprococcus* 2 was associated with a higher risk of UC (OR: 1.32, 95% CI: 1.08-1.62, *P*=0.007 in IVW method) (**Figure 3**). Other sensitivity analyses also supported the result of primary analysis (OR: 1.36, 95% CI: 1.02-1.81, *P*=0.034 in the weighted median method; OR: 1.32, 95% CI: 1.09-1.60, *P*=0.015 in MR-PRESSO test and intercept *P*=0.491 in MR-Egger regression) (**Figure 3** and **Supplemental Table S3**).

### CD

We noticed 11 bacterial traits associated with CD, while only four of them were also associated with IBD in the IVW method (**Table 1** and **Figure 2**). However, we found the results of only three gut microbial genera that were stable in the sensitivity methods (**Figure 3**).

Genus *Lachnospiraceae* UCG001 were negatively correlated with the risk of CD in the IVW method (OR=0.81, 95% CI: 0.67–0.97, *P*=0.023). The MR estimates of weighted median and MR-PROSSO indicated similar results (OR=0.75, 95% CI: 0.58-0.95, *P*=0.025 in weighted median analysis and OR=0.81, 95% CI: 0.68-0.96, *P*=0.033 in MR-PRESSO) (**Figure 3**). Additionally, little evidence of directional pleiotropy was found for genus *Lachnospiraceae* UCG001 in MR-Egger regression (intercept *P*=0.940) (**Supplemental Table S4**).

As for genus *Oxalobacter* and *Ruminococcaceae* UCG014, we found positive associations with the risk of CD in the IVW method (OR=1.23, 95% CI: 1.08-1.40, *P*=0.002 for *Oxalobacter* and OR=1.32, 95% CI: 1.08-1.61, *P*=0.007 for *Ruminococcaceae* UCG014) (**Figure 3**). The other sensitivity methods also supported their relationship (OR=1.27, 95% CI: 1.05-1.54, *P*=0.015 for *Oxalobacter* and OR=1.42, 95% CI: 1.08-1.86, *P*=0.012 for *Ruminococcaceae* UCG014 in the weighted median method; OR=1.23, 95% CI: 1.07–1.42, *P*=0.017 for *Oxalobacter* and OR=1.32, 95% CI: 1.09-1.58, *P*=0.010 for *Ruminococcaceae* UCG014 in MR-PRESSO; intercept *P*=0.618 for *Oxalobacter* and intercept *P*=0.618 for *Ruminococcaceae* UCG014) (**Figure 3** and **Supplemental Table S4**).

### Reverse MR Analyses

Finally, we performed a reverse MR analysis between these six gut microbial genera and IBD, UC, or CD, and we did not find reverse causal relationships between them in the IVW method. The results of other sensitivity methods are listed in **Supplemental Table S6**.

## Discussion

This study was not the first to reveal the causal association between gut microbiota and IBD, but it had the following innovations: i) The GWAS database of human gut microbiota used in this study was a big and newly GWAS database, which contained a larger population; ii) This study revealed the difference in causally associated gut microbiota between UC and CD at the genus level. There was no overlap between the above gut microbial genera and those genera being previously reported to be causally associated with IBD (6, 7). Therefore, our finding expanded the gut microbial genera that were causally associated with the IBD, and deeply implicated the regulatory role of gut microbiota in IBD.

In this study, a two-sample MR analysis successfully identified that *Coprococcus 2*, *Oxalobacter*, and *Ruminococcaceae* UCG014 were positively related to the risk of IBD. Genus *Coprococcus*, a butyrate-producing bacteria, were significantly reduced in IBD patients (27). Agglutinating antibodies for *Coprococcus* were considered as a biomarker for screening CD (28). *Oxalobacter formigenes*, one species of genus *Oxalobacter*, were significantly lower in IBD patients than healthy subjects and this might contribute to hyperoxaluria in IBD (29). *Ruminococcaceae* UCG-014 had been reported to perturb in the process of constructing and treating IBD mice (30, 31). Interestingly, the above gut bacterial traits being positively associated with IBD were all reported to be reduced in IBD patients. The reason might be that these bacterial traits were the initiating factors of IBD, the host could produce specific antibodies to reduce the abundance of these bacterial traits after IBD occurring.

In addition, the two-sample MR analysis also identified two gut microbial genera being negatively related to the risk of IBD, including genus *Enterorhabdus* and *Eubacterium ventriosum* group. Genus *Enterorhabdus* was associated with a genetic variant of the human leukocyte antigen complex, which has been related to inflammatory diseases (32). Besides, a reduction of *Enterorhabdus* was associated with smoking aggravating IBD (33). *Eubacterium ventriosum* group was less present in the IBD group than in the healthy group (34).

For a long time, researchers have tried to reveal the differences in the pathogenesis of UC and CD from the perspective of gut microbiota. A previous study found significant disease-specific alterations at or below the order level in the taxonomic rank in UC vs. CD (12). Our two-sample MR analysis also identified that genus *Coprococcus* 2 and *Eubacterium ventriosum* group were specifically causally associated with UC, *Lachnospiraceae* UCG001, *Ruminococcaceae* UCG014, and *Oxalobacter* were specifically causally associated with CD. These specific bacterial genera of UC or CD was firstly reported in UC or CD patients, respectively. Therefore, our findings provided a new direction for revealing the difference in gut microbial genera mediating pathogenesis of UC and CD.

In conclusion, this MR study confirms once again that gut microbiota has causal effects on IBD. Not only that, this study provides the specific gut microbial genera involved in the pathogenesis of UC or CD. However, some limitations should be noted. First, this study was unable to determine whether overlapping participants were enrolled in the exposure and outcome GWAS used in the two-sample MR analyses. Second, bacterial taxa were only analyzed at the genus level but not at a more specialized level such as species or strain levels. Third, this study could not further answer why there is a difference in UC-specific and CD-specific gut microbial genera. Above all, our finding could offer new insights into the development and treatment of IBD, UC, and CD.

## Data Availability Statement

The original contributions presented in the study are included in the article/**Supplementary Material**. Further inquiries can be directed to the corresponding authors.

## Author Contributions

BL, YM, and ZH designed the research. BL, XS, HY, JS, and YM collected and analyzed the data. BL, DY, YM, and ZH performed the literature search. BL and ZH drafted the article. DY and YM supervised the study. All authors were involved in writing the paper. All authors contributed to the article and approved the submitted version.

## Funding

This work was jointly supported by the National Natural Science Foundation of China (No. 82174208, 82074217) and the Research Project of Zhejiang Chinese Medical University (No. 2021JKZKTS001A, 2021JKZKTS004A).

## Conflict of Interest

The authors declare that the research was conducted in the absence of any commercial or financial relationships that could be construed as a potential conflict of interest.

## Publisher’s Note

All claims expressed in this article are solely those of the authors and do not necessarily represent those of their affiliated organizations, or those of the publisher, the editors and the reviewers. Any product that may be evaluated in this article, or claim that may be made by its manufacturer, is not guaranteed or endorsed by the publisher.
