# Supplementary material for: Two-Sample Mendelian Randomization Analysis Investigates Causal Associations Between Gut Microbial Genera and Inflammatory Bowel Disease, and Specificity Causal Associations in Ulcerative Colitis or Crohn’s Disease
Source: Front Immunol. 2022 Jul 4;13:921546. doi: 10.3389/fimmu.2022.921546 (PMC9289607; doi:10.3389/fimmu.2022.921546)
Supplement: Supplementary file 1 [file Table_1.docx]

Supplementary Material

**Supplementary Table 1** Characteristics of the genetic variants associated with the risk of IBD, UC and CD.

Abbreviations: CD, crohn's disease; Chr, chromosome; IBD, inflammatory bowel disease; SE, standard error; SNP, single nucleotide polymorphism; UC, ulcerative colitis.

**Supplementary Table 2** Effect estimates of the associations between 196 bacterial traits and risk of inflammatory bowel disease in MR analyses

Abbreviations: CI, confidence interval; MR, Mendelian randomization; MR-PRESSO test, MR Pleiotropy RESidual Sum and Outlier test; OR, odds ratio; SNP, single nucleotide polymorphism. **P*-value of the intercept from MR-Egger regression analysis.

**Supplementary Table 3** Effect estimates of the associations between 196 bacterial traits and risk of ulcerative colitis in MR analyses

Abbreviations: CI, confidence interval; MR, Mendelian randomization; MR-PRESSO test, MR Pleiotropy RESidual Sum and Outlier test; OR, odds ratio; SNP, single nucleotide polymorphism. **P*-value of the intercept from MR-Egger regression analysis.

**Supplementary Table 4** Effect estimates of the associations between 196 bacterial traits and risk of Crohn's disease in MR analyses

Abbreviations: CI, confidence interval; MR, Mendelian randomization; MR-PRESSO test, MR Pleiotropy RESidual Sum and Outlier test; OR, odds ratio; SNP, single nucleotide polymorphism. **P*-value of the intercept from MR-Egger regression analysis.

**Supplementary Table 5** Characteristics of the genetic variants associated with six bacterial that have been identified to be associated with the risk of IBD UC, CD.

Abbreviations: Chr, chromosome; CD, crohn's disease; IBD, inflammatory bowel disease; SE, standard error; SNP, single nucleotide polymorphism; UC, ulcerative colitis.

**Supplementary Table 6** Effect estimates of the associations between IBD, UC, CD and risk of six bacterial traits in the reverse MR analyses.

Abbreviations: CD, crohn's disease; CI, confidence interval; IBD, inflammatory bowel disease; MR, Mendelian randomization; MR-PRESSO test, MR Pleiotropy RESidual Sum and Outlier test; OR, odds ratio; SNP, single nucleotide polymorphism. **P*-value of the intercept from MR-Egger regression analysis.

| **Supplementary Table 1** Characteristics of the genetic variants associated with the risk of IBD, UC and CD.   \| Trait \| SNP \| Chr \| Position \| Effect allele \| Beta \| SE \| P-value \| \| --- \| --- \| --- \| --- \| --- \| --- \| --- \| --- \| \| IBD \| rs10800309 \| 1 \| 161472158 \| G \| -0.132 \| 0.010 \| 6.15E-37 \| \| IBD \| rs12103 \| 1 \| 1247494 \| C \| -0.087 \| 0.013 \| 3.28E-11 \| \| IBD \| rs12411259 \| 1 \| 172866210 \| A \| 0.067 \| 0.012 \| 6.18E-09 \| \| IBD \| rs16841904 \| 1 \| 197701992 \| T \| 0.099 \| 0.012 \| 5.51E-16 \| \| IBD \| rs2297559 \| 1 \| 160854526 \| A \| 0.074 \| 0.011 \| 1.88E-11 \| \| IBD \| rs2974935 \| 1 \| 155181843 \| T \| 0.069 \| 0.010 \| 8.87E-12 \| \| IBD \| rs3024493 \| 1 \| 206943968 \| A \| 0.197 \| 0.013 \| 1.65E-50 \| \| IBD \| rs34856868 \| 1 \| 92554283 \| A \| -0.195 \| 0.034 \| 9.80E-09 \| \| IBD \| rs59655222 \| 1 \| 200875897 \| C \| -0.159 \| 0.011 \| 1.43E-44 \| \| IBD \| rs6588248 \| 1 \| 67652984 \| G \| 0.082 \| 0.010 \| 1.38E-16 \| \| IBD \| rs72634258 \| 1 \| 8150638 \| C \| -0.127 \| 0.014 \| 1.25E-19 \| \| IBD \| rs7523442 \| 1 \| 20165971 \| T \| 0.125 \| 0.010 \| 2.76E-36 \| \| IBD \| rs7547569 \| 1 \| 67731368 \| C \| -0.647 \| 0.023 \| 1.65E-170 \| \| IBD \| rs11677953 \| 2 \| 219121663 \| A \| 0.079 \| 0.010 \| 2.92E-15 \| \| IBD \| rs11691685 \| 2 \| 145481827 \| G \| -0.122 \| 0.019 \| 7.27E-11 \| \| IBD \| rs13407913 \| 2 \| 25097644 \| G \| 0.092 \| 0.010 \| 1.69E-20 \| \| IBD \| rs1420098 \| 2 \| 102984279 \| C \| -0.095 \| 0.010 \| 1.83E-20 \| \| IBD \| rs1517352 \| 2 \| 191931464 \| C \| 0.078 \| 0.010 \| 3.87E-14 \| \| IBD \| rs1990760 \| 2 \| 163124051 \| T \| -0.067 \| 0.011 \| 3.56E-10 \| \| IBD \| rs35256947 \| 2 \| 231161026 \| C \| 0.082 \| 0.011 \| 3.87E-13 \| \| IBD \| rs6708373 \| 2 \| 234172846 \| G \| 0.134 \| 0.010 \| 1.43E-41 \| \| IBD \| rs6740462 \| 2 \| 65667272 \| A \| 0.080 \| 0.012 \| 5.59E-12 \| \| IBD \| rs6745185 \| 2 \| 241586960 \| G \| 0.070 \| 0.012 \| 1.37E-09 \| \| IBD \| rs72924296 \| 2 \| 199543967 \| G \| -0.064 \| 0.011 \| 1.44E-08 \| \| IBD \| rs7608910 \| 2 \| 61204856 \| G \| 0.126 \| 0.010 \| 2.60E-36 \| \| IBD \| rs77059113 \| 2 \| 43672508 \| G \| 0.151 \| 0.019 \| 1.39E-15 \| \| IBD \| rs780094 \| 2 \| 27741237 \| C \| -0.078 \| 0.010 \| 3.88E-15 \| \| IBD \| rs11713774 \| 3 \| 18765978 \| C \| 0.094 \| 0.014 \| 3.92E-11 \| \| IBD \| rs9836291 \| 3 \| 49697459 \| A \| 0.161 \| 0.011 \| 9.61E-53 \| \| IBD \| rs13107612 \| 4 \| 102739980 \| T \| 0.073 \| 0.011 \| 1.62E-11 \| \| IBD \| rs4692386 \| 4 \| 26132361 \| C \| 0.058 \| 0.010 \| 1.21E-08 \| \| IBD \| rs7657746 \| 4 \| 123161619 \| G \| -0.087 \| 0.012 \| 1.83E-13 \| \| IBD \| rs974801 \| 4 \| 106071064 \| G \| -0.073 \| 0.010 \| 7.07E-13 \| \| IBD \| rs1363907 \| 5 \| 96252803 \| A \| 0.082 \| 0.010 \| 4.87E-15 \| \| IBD \| rs181826 \| 5 \| 141526057 \| A \| 0.082 \| 0.010 \| 4.05E-15 \| \| IBD \| rs272882 \| 5 \| 131669161 \| T \| 0.166 \| 0.011 \| 1.47E-52 \| \| IBD \| rs34804116 \| 5 \| 72539850 \| A \| -0.057 \| 0.010 \| 3.62E-08 \| \| IBD \| rs3776414 \| 5 \| 10689562 \| G \| 0.077 \| 0.010 \| 2.65E-14 \| \| IBD \| rs4703855 \| 5 \| 71693899 \| T \| -0.071 \| 0.011 \| 7.16E-11 \| \| IBD \| rs4976646 \| 5 \| 176788570 \| C \| 0.073 \| 0.010 \| 3.23E-12 \| \| IBD \| rs56167332 \| 5 \| 158827769 \| A \| 0.156 \| 0.010 \| 7.17E-50 \| \| IBD \| rs71593329 \| 5 \| 158620079 \| G \| -0.098 \| 0.013 \| 1.19E-14 \| \| IBD \| rs7711427 \| 5 \| 40414886 \| C \| 0.175 \| 0.010 \| 4.63E-66 \| \| IBD \| rs79980175 \| 5 \| 40521892 \| C \| -0.095 \| 0.015 \| 1.30E-10 \| \| IBD \| rs11152949 \| 6 \| 106449085 \| G \| 0.105 \| 0.011 \| 7.25E-23 \| \| IBD \| rs1267499 \| 6 \| 14715882 \| C \| 0.082 \| 0.013 \| 5.22E-11 \| \| IBD \| rs13204742 \| 6 \| 128245765 \| T \| 0.092 \| 0.015 \| 5.39E-10 \| \| IBD \| rs1847472 \| 6 \| 90973159 \| A \| -0.067 \| 0.011 \| 6.63E-10 \| \| IBD \| rs2328546 \| 6 \| 20657345 \| C \| 0.094 \| 0.013 \| 1.30E-13 \| \| IBD \| rs62434177 \| 6 \| 138087506 \| A \| -0.179 \| 0.031 \| 1.14E-08 \| \| IBD \| rs6456426 \| 6 \| 21438889 \| A \| -0.064 \| 0.010 \| 8.18E-11 \| \| IBD \| rs6933404 \| 6 \| 137959235 \| C \| 0.096 \| 0.012 \| 5.84E-15 \| \| IBD \| rs769177 \| 6 \| 31547611 \| T \| 0.261 \| 0.029 \| 6.53E-20 \| \| IBD \| rs7773324 \| 6 \| 382559 \| A \| 0.062 \| 0.011 \| 5.84E-09 \| \| IBD \| rs9264942 \| 6 \| 31274380 \| C \| 0.095 \| 0.011 \| 1.55E-18 \| \| IBD \| rs9273363 \| 6 \| 32626272 \| A \| -0.193 \| 0.012 \| 3.30E-58 \| \| IBD \| rs9400484 \| 6 \| 111949779 \| G \| 0.104 \| 0.016 \| 1.96E-10 \| \| IBD \| rs9457247 \| 6 \| 167392174 \| T \| 0.089 \| 0.010 \| 2.48E-18 \| \| IBD \| rs1182188 \| 7 \| 2869985 \| C \| -0.066 \| 0.011 \| 1.08E-09 \| \| IBD \| rs12718244 \| 7 \| 50175654 \| A \| 0.076 \| 0.010 \| 3.35E-14 \| \| IBD \| rs2538470 \| 7 \| 148220448 \| G \| -0.068 \| 0.010 \| 3.00E-11 \| \| IBD \| rs3801835 \| 7 \| 26852053 \| T \| 0.064 \| 0.011 \| 1.47E-09 \| \| IBD \| rs740287 \| 7 \| 107584780 \| T \| -0.081 \| 0.010 \| 5.04E-16 \| \| IBD \| rs1551399 \| 8 \| 126539965 \| C \| 0.083 \| 0.010 \| 2.64E-16 \| \| IBD \| rs6651252 \| 8 \| 129567181 \| C \| -0.091 \| 0.015 \| 9.08E-10 \| \| IBD \| rs7011507 \| 8 \| 49129242 \| A \| -0.085 \| 0.015 \| 2.03E-08 \| \| IBD \| rs7015630 \| 8 \| 90875918 \| C \| -0.063 \| 0.011 \| 2.90E-08 \| \| IBD \| rs10758669 \| 9 \| 4981602 \| A \| -0.149 \| 0.010 \| 4.70E-48 \| \| IBD \| rs11793497 \| 9 \| 139271850 \| G \| 0.156 \| 0.010 \| 1.71E-54 \| \| IBD \| rs4743820 \| 9 \| 93928416 \| T \| 0.064 \| 0.011 \| 3.80E-09 \| \| IBD \| rs7848647 \| 9 \| 117569046 \| C \| 0.132 \| 0.011 \| 3.16E-35 \| \| IBD \| rs10761659 \| 10 \| 64445564 \| G \| 0.154 \| 0.010 \| 4.97E-53 \| \| IBD \| rs1250566 \| 10 \| 81046453 \| A \| -0.101 \| 0.011 \| 4.77E-20 \| \| IBD \| rs12722515 \| 10 \| 6081230 \| A \| -0.099 \| 0.014 \| 4.57E-12 \| \| IBD \| rs2050392 \| 10 \| 30691503 \| A \| 0.069 \| 0.010 \| 1.87E-11 \| \| IBD \| rs2153283 \| 10 \| 59972299 \| A \| -0.086 \| 0.013 \| 1.54E-11 \| \| IBD \| rs2274351 \| 10 \| 104264107 \| T \| 0.060 \| 0.010 \| 6.93E-09 \| \| IBD \| rs2497318 \| 10 \| 94432000 \| T \| -0.064 \| 0.010 \| 1.36E-10 \| \| IBD \| rs2688608 \| 10 \| 75658349 \| T \| 0.062 \| 0.010 \| 2.75E-10 \| \| IBD \| rs34779708 \| 10 \| 35466185 \| G \| 0.107 \| 0.010 \| 2.07E-25 \| \| IBD \| rs6584281 \| 10 \| 101286480 \| G \| -0.165 \| 0.010 \| 9.36E-62 \| \| IBD \| rs11230563 \| 11 \| 60776209 \| T \| -0.081 \| 0.011 \| 1.71E-14 \| \| IBD \| rs11236797 \| 11 \| 76299649 \| A \| 0.151 \| 0.010 \| 9.32E-52 \| \| IBD \| rs12796489 \| 11 \| 3059360 \| A \| -0.760 \| 0.043 \| 2.87E-69 \| \| IBD \| rs559928 \| 11 \| 64150370 \| C \| 0.094 \| 0.013 \| 3.33E-13 \| \| IBD \| rs648541 \| 11 \| 114429934 \| G \| -0.065 \| 0.011 \| 1.22E-09 \| \| IBD \| rs10878386 \| 12 \| 40725928 \| G \| -0.109 \| 0.019 \| 5.51E-09 \| \| IBD \| rs12318183 \| 12 \| 68503836 \| A \| 0.110 \| 0.010 \| 1.67E-27 \| \| IBD \| rs1388585 \| 12 \| 40531691 \| A \| -0.305 \| 0.032 \| 6.85E-22 \| \| IBD \| rs3184504 \| 12 \| 111884608 \| C \| -0.060 \| 0.010 \| 1.29E-09 \| \| IBD \| rs12585310 \| 13 \| 27528347 \| A \| 0.071 \| 0.011 \| 5.25E-11 \| \| IBD \| rs6561151 \| 13 \| 44484706 \| A \| 0.100 \| 0.012 \| 3.53E-17 \| \| IBD \| rs941823 \| 13 \| 41013977 \| C \| 0.083 \| 0.012 \| 6.19E-13 \| \| IBD \| rs9557207 \| 13 \| 100036418 \| G \| -0.088 \| 0.012 \| 3.52E-13 \| \| IBD \| rs10142466 \| 14 \| 69271784 \| G \| -0.058 \| 0.010 \| 1.08E-08 \| \| IBD \| rs1569328 \| 14 \| 75741751 \| T \| -0.081 \| 0.014 \| 3.21E-09 \| \| IBD \| rs55808324 \| 14 \| 88444752 \| A \| 0.141 \| 0.017 \| 5.13E-17 \| \| IBD \| rs17293632 \| 15 \| 67442596 \| T \| 0.107 \| 0.012 \| 2.71E-20 \| \| IBD \| rs17651741 \| 15 \| 38869666 \| A \| 0.070 \| 0.013 \| 2.81E-08 \| \| IBD \| rs11117431 \| 16 \| 86015316 \| G \| -0.111 \| 0.013 \| 1.01E-16 \| \| IBD \| rs2270395 \| 16 \| 50846832 \| T \| 0.078 \| 0.012 \| 5.17E-11 \| \| IBD \| rs367569 \| 16 \| 11365500 \| T \| -0.096 \| 0.011 \| 1.93E-17 \| \| IBD \| rs62037363 \| 16 \| 28865042 \| C \| 0.099 \| 0.010 \| 6.36E-22 \| \| IBD \| rs6500315 \| 16 \| 50508101 \| G \| 0.077 \| 0.012 \| 1.12E-10 \| \| IBD \| rs7194886 \| 16 \| 50725193 \| T \| -0.126 \| 0.010 \| 2.53E-36 \| \| IBD \| rs1292053 \| 17 \| 57963537 \| G \| 0.070 \| 0.010 \| 9.89E-13 \| \| IBD \| rs17780256 \| 17 \| 70642923 \| C \| -0.083 \| 0.013 \| 3.19E-11 \| \| IBD \| rs4795397 \| 17 \| 38023745 \| G \| 0.138 \| 0.010 \| 8.30E-44 \| \| IBD \| rs744166 \| 17 \| 40514201 \| G \| -0.100 \| 0.010 \| 1.14E-22 \| \| IBD \| rs9889296 \| 17 \| 32570547 \| A \| -0.105 \| 0.011 \| 1.35E-20 \| \| IBD \| rs2847278 \| 18 \| 12778715 \| T \| -0.145 \| 0.013 \| 8.33E-28 \| \| IBD \| rs67643815 \| 18 \| 67561508 \| T \| -0.063 \| 0.010 \| 6.42E-10 \| \| IBD \| rs7240004 \| 18 \| 46395022 \| G \| -0.067 \| 0.010 \| 1.01E-10 \| \| IBD \| rs17694108 \| 19 \| 33731551 \| A \| 0.086 \| 0.011 \| 1.21E-14 \| \| IBD \| rs2024092 \| 19 \| 1124031 \| A \| 0.107 \| 0.012 \| 1.12E-18 \| \| IBD \| rs35164067 \| 19 \| 10525181 \| A \| -0.118 \| 0.013 \| 2.66E-20 \| \| IBD \| rs516246 \| 19 \| 49206172 \| T \| 0.076 \| 0.010 \| 1.15E-13 \| \| IBD \| rs7253253 \| 19 \| 10714058 \| T \| -0.134 \| 0.023 \| 6.19E-09 \| \| IBD \| rs259964 \| 20 \| 57824309 \| G \| -0.067 \| 0.010 \| 6.93E-12 \| \| IBD \| rs6058869 \| 20 \| 31348750 \| T \| 0.056 \| 0.010 \| 2.63E-08 \| \| IBD \| rs6062496 \| 20 \| 62329099 \| A \| 0.123 \| 0.010 \| 2.11E-33 \| \| IBD \| rs6074022 \| 20 \| 44740196 \| T \| -0.074 \| 0.011 \| 8.32E-11 \| \| IBD \| rs6111031 \| 20 \| 1682037 \| T \| -0.264 \| 0.015 \| 1.23E-71 \| \| IBD \| rs913678 \| 20 \| 48955424 \| C \| -0.069 \| 0.011 \| 5.35E-11 \| \| IBD \| rs1297258 \| 21 \| 16806709 \| T \| -0.115 \| 0.010 \| 5.38E-30 \| \| IBD \| rs2836883 \| 21 \| 40466744 \| A \| -0.168 \| 0.012 \| 3.38E-48 \| \| IBD \| rs8127691 \| 21 \| 45614860 \| C \| -0.114 \| 0.010 \| 8.98E-30 \| \| IBD \| rs2143178 \| 22 \| 39660829 \| C \| -0.177 \| 0.014 \| 4.80E-38 \| \| UC \| rs10910092 \| 1 \| 2501516 \| G \| -0.086 \| 0.013 \| 1.42E-11 \| \| UC \| rs111830527 \| 1 \| 22687173 \| A \| -0.192 \| 0.029 \| 5.09E-11 \| \| UC \| rs12103 \| 1 \| 1247494 \| C \| -0.100 \| 0.016 \| 9.96E-10 \| \| UC \| rs16841904 \| 1 \| 197701992 \| T \| 0.086 \| 0.015 \| 1.90E-08 \| \| UC \| rs1801274 \| 1 \| 161479745 \| G \| -0.171 \| 0.013 \| 1.43E-41 \| \| UC \| rs3024493 \| 1 \| 206943968 \| A \| 0.226 \| 0.016 \| 1.42E-43 \| \| UC \| rs35223180 \| 1 \| 8185902 \| T \| -0.141 \| 0.018 \| 1.04E-15 \| \| UC \| rs4656958 \| 1 \| 160856964 \| G \| 0.082 \| 0.014 \| 2.82E-09 \| \| UC \| rs59655222 \| 1 \| 200875897 \| C \| -0.167 \| 0.014 \| 3.81E-31 \| \| UC \| rs6426833 \| 1 \| 20171860 \| A \| 0.232 \| 0.013 \| 3.77E-76 \| \| UC \| rs7547569 \| 1 \| 67731368 \| C \| -0.496 \| 0.029 \| 8.71E-65 \| \| UC \| rs10185424 \| 2 \| 102662888 \| G \| -0.097 \| 0.013 \| 1.47E-14 \| \| UC \| rs10460566 \| 2 \| 25483121 \| A \| -0.082 \| 0.014 \| 1.60E-08 \| \| UC \| rs11676348 \| 2 \| 219010146 \| T \| 0.074 \| 0.012 \| 2.08E-09 \| \| UC \| rs13430791 \| 2 \| 43481013 \| A \| 0.106 \| 0.019 \| 1.39E-08 \| \| UC \| rs1990760 \| 2 \| 163124051 \| T \| -0.086 \| 0.013 \| 1.78E-10 \| \| UC \| rs4676410 \| 2 \| 241563739 \| A \| 0.142 \| 0.016 \| 1.85E-19 \| \| UC \| rs4973341 \| 2 \| 228660362 \| T \| 0.073 \| 0.013 \| 2.25E-08 \| \| UC \| rs7608910 \| 2 \| 61204856 \| G \| 0.127 \| 0.013 \| 1.25E-23 \| \| UC \| rs9941524 \| 2 \| 199499443 \| G \| 0.098 \| 0.013 \| 2.15E-14 \| \| UC \| rs9836291 \| 3 \| 49697459 \| A \| 0.170 \| 0.013 \| 8.20E-38 \| \| UC \| rs13136827 \| 4 \| 123171318 \| C \| -0.112 \| 0.018 \| 2.35E-10 \| \| UC \| rs3774937 \| 4 \| 103434253 \| C \| 0.099 \| 0.013 \| 4.61E-14 \| \| UC \| rs272882 \| 5 \| 131669161 \| T \| 0.146 \| 0.014 \| 6.67E-26 \| \| UC \| rs36070529 \| 5 \| 158619835 \| A \| -0.092 \| 0.016 \| 1.04E-08 \| \| UC \| rs3776414 \| 5 \| 10689562 \| G \| 0.070 \| 0.013 \| 4.10E-08 \| \| UC \| rs4976646 \| 5 \| 176788570 \| C \| 0.079 \| 0.013 \| 2.52E-09 \| \| UC \| rs56167332 \| 5 \| 158827769 \| A \| 0.141 \| 0.013 \| 7.27E-27 \| \| UC \| rs7711427 \| 5 \| 40414886 \| C \| 0.089 \| 0.013 \| 3.67E-12 \| \| UC \| rs2516440 \| 6 \| 31440497 \| A \| -0.100 \| 0.014 \| 4.40E-13 \| \| UC \| rs34659678 \| 6 \| 111888540 \| T \| 0.210 \| 0.025 \| 5.95E-17 \| \| UC \| rs4712520 \| 6 \| 20640871 \| C \| 0.093 \| 0.017 \| 2.21E-08 \| \| UC \| rs4947328 \| 6 \| 31561747 \| G \| 0.239 \| 0.038 \| 3.38E-10 \| \| UC \| rs6920220 \| 6 \| 138006504 \| A \| 0.147 \| 0.015 \| 4.78E-22 \| \| UC \| rs7738430 \| 6 \| 31508836 \| C \| 0.368 \| 0.034 \| 3.51E-27 \| \| UC \| rs9271255 \| 6 \| 32580357 \| T \| -0.285 \| 0.014 \| 1.31E-94 \| \| UC \| rs9271858 \| 6 \| 32595223 \| G \| 0.123 \| 0.013 \| 2.42E-21 \| \| UC \| rs1077773 \| 7 \| 17442679 \| A \| 0.072 \| 0.012 \| 5.96E-09 \| \| UC \| rs1182188 \| 7 \| 2869985 \| C \| -0.108 \| 0.014 \| 5.03E-15 \| \| UC \| rs12718244 \| 7 \| 50175654 \| A \| 0.072 \| 0.013 \| 1.41E-08 \| \| UC \| rs4380874 \| 7 \| 107480315 \| C \| -0.131 \| 0.013 \| 6.43E-25 \| \| UC \| rs4728142 \| 7 \| 128573967 \| A \| 0.097 \| 0.013 \| 1.92E-14 \| \| UC \| rs76546301 \| 7 \| 50498389 \| A \| 0.265 \| 0.041 \| 1.05E-10 \| \| UC \| rs13255292 \| 8 \| 129076573 \| T \| -0.075 \| 0.014 \| 3.82E-08 \| \| UC \| rs10758669 \| 9 \| 4981602 \| A \| -0.143 \| 0.013 \| 1.04E-28 \| \| UC \| rs11793497 \| 9 \| 139271850 \| G \| 0.134 \| 0.013 \| 1.74E-26 \| \| UC \| rs4366152 \| 9 \| 117564875 \| C \| 0.120 \| 0.014 \| 7.79E-19 \| \| UC \| rs4743820 \| 9 \| 93928416 \| T \| 0.081 \| 0.014 \| 4.05E-09 \| \| UC \| rs10748783 \| 10 \| 101285872 \| A \| -0.165 \| 0.013 \| 7.73E-39 \| \| UC \| rs10761659 \| 10 \| 64445564 \| G \| 0.117 \| 0.013 \| 1.50E-20 \| \| UC \| rs2274351 \| 10 \| 104264107 \| T \| 0.071 \| 0.013 \| 4.90E-08 \| \| UC \| rs2497318 \| 10 \| 94432000 \| T \| -0.071 \| 0.013 \| 1.15E-08 \| \| UC \| rs4747886 \| 10 \| 6176166 \| T \| 0.074 \| 0.013 \| 9.58E-09 \| \| UC \| rs59418206 \| 10 \| 35331624 \| A \| 0.074 \| 0.013 \| 1.45E-08 \| \| UC \| rs11229555 \| 11 \| 58408687 \| T \| -0.082 \| 0.014 \| 1.21E-08 \| \| UC \| rs11230563 \| 11 \| 60776209 \| T \| -0.075 \| 0.013 \| 1.90E-08 \| \| UC \| rs12796489 \| 11 \| 3059360 \| A \| -0.676 \| 0.056 \| 1.22E-33 \| \| UC \| rs483905 \| 11 \| 96023427 \| A \| 0.085 \| 0.014 \| 3.16E-10 \| \| UC \| rs61893460 \| 11 \| 76291154 \| A \| 0.121 \| 0.013 \| 4.60E-22 \| \| UC \| rs661054 \| 11 \| 114430410 \| G \| -0.125 \| 0.014 \| 3.18E-20 \| \| UC \| rs12318183 \| 12 \| 68503836 \| A \| 0.162 \| 0.013 \| 1.44E-37 \| \| UC \| rs76904798 \| 12 \| 40614434 \| T \| 0.105 \| 0.018 \| 2.78E-09 \| \| UC \| rs1359946 \| 13 \| 27536972 \| A \| 0.131 \| 0.016 \| 9.64E-17 \| \| UC \| rs941823 \| 13 \| 41013977 \| C \| 0.109 \| 0.015 \| 1.39E-13 \| \| UC \| rs55808324 \| 14 \| 88444752 \| A \| 0.127 \| 0.021 \| 1.47E-09 \| \| UC \| rs11150589 \| 16 \| 30482494 \| C \| -0.080 \| 0.013 \| 3.28E-10 \| \| UC \| rs11641184 \| 16 \| 11704651 \| A \| 0.078 \| 0.012 \| 4.24E-10 \| \| UC \| rs7404095 \| 16 \| 23864590 \| C \| 0.072 \| 0.013 \| 1.52E-08 \| \| UC \| rs79045992 \| 16 \| 68518992 \| A \| 0.118 \| 0.021 \| 1.43E-08 \| \| UC \| rs17780256 \| 17 \| 70642923 \| C \| -0.115 \| 0.016 \| 6.13E-13 \| \| UC \| rs4795397 \| 17 \| 38023745 \| G \| 0.140 \| 0.013 \| 1.01E-28 \| \| UC \| rs9891119 \| 17 \| 40507980 \| C \| -0.090 \| 0.013 \| 1.72E-11 \| \| UC \| rs7240004 \| 18 \| 46395022 \| G \| -0.082 \| 0.013 \| 2.50E-10 \| \| UC \| rs8096327 \| 18 \| 12887750 \| G \| 0.094 \| 0.013 \| 2.24E-13 \| \| UC \| rs11083840 \| 19 \| 47119910 \| G \| 0.069 \| 0.013 \| 3.41E-08 \| \| UC \| rs12720356 \| 19 \| 10469975 \| C \| 0.153 \| 0.023 \| 1.67E-11 \| \| UC \| rs17694108 \| 19 \| 33731551 \| A \| 0.096 \| 0.014 \| 6.17E-12 \| \| UC \| rs4812833 \| 20 \| 43068996 \| A \| 0.103 \| 0.013 \| 1.87E-16 \| \| UC \| rs6062496 \| 20 \| 62329099 \| A \| 0.114 \| 0.013 \| 9.14E-19 \| \| UC \| rs6111031 \| 20 \| 1682037 \| T \| -0.261 \| 0.019 \| 1.33E-42 \| \| UC \| rs913678 \| 20 \| 48955424 \| C \| -0.076 \| 0.013 \| 1.23E-08 \| \| UC \| rs1297256 \| 21 \| 16805676 \| T \| -0.101 \| 0.013 \| 2.10E-15 \| \| UC \| rs2836883 \| 21 \| 40466744 \| A \| -0.227 \| 0.015 \| 1.47E-53 \| \| UC \| rs4456788 \| 21 \| 45616324 \| A \| -0.103 \| 0.013 \| 7.07E-16 \| \| UC \| rs9611131 \| 22 \| 39662480 \| C \| -0.143 \| 0.018 \| 3.84E-15 \| \| CD \| rs10798069 \| 1 \| 186875459 \| T \| -0.070 \| 0.012 \| 4.25E-09 \| \| CD \| rs10800309 \| 1 \| 161472158 \| G \| -0.090 \| 0.013 \| 8.48E-13 \| \| CD \| rs12131796 \| 1 \| 200878727 \| A \| -0.150 \| 0.014 \| 1.71E-27 \| \| CD \| rs12411259 \| 1 \| 172866210 \| A \| 0.134 \| 0.014 \| 1.43E-22 \| \| CD \| rs17129991 \| 1 \| 67862986 \| T \| -0.284 \| 0.045 \| 2.81E-10 \| \| CD \| rs17391694 \| 1 \| 78623626 \| T \| -0.119 \| 0.020 \| 2.62E-09 \| \| CD \| rs2488389 \| 1 \| 197631141 \| A \| 0.113 \| 0.015 \| 8.59E-15 \| \| CD \| rs2641348 \| 1 \| 120437884 \| G \| -0.121 \| 0.020 \| 9.65E-10 \| \| CD \| rs2974935 \| 1 \| 155181843 \| T \| 0.076 \| 0.012 \| 5.80E-10 \| \| CD \| rs3024505 \| 1 \| 206939904 \| A \| 0.165 \| 0.016 \| 3.95E-25 \| \| CD \| rs36016881 \| 1 \| 8051241 \| G \| -0.109 \| 0.017 \| 1.60E-10 \| \| CD \| rs6679677 \| 1 \| 114303808 \| A \| -0.185 \| 0.022 \| 4.67E-17 \| \| CD \| rs7517847 \| 1 \| 67681669 \| G \| -0.336 \| 0.012 \| 1.38E-159 \| \| CD \| rs11691685 \| 2 \| 145481827 \| G \| -0.158 \| 0.023 \| 1.35E-11 \| \| CD \| rs12694846 \| 2 \| 231148128 \| G \| 0.115 \| 0.014 \| 2.50E-17 \| \| CD \| rs13001325 \| 2 \| 102939036 \| T \| -0.123 \| 0.013 \| 1.68E-22 \| \| CD \| rs13407913 \| 2 \| 25097644 \| G \| 0.115 \| 0.012 \| 9.64E-22 \| \| CD \| rs1517352 \| 2 \| 191931464 \| C \| 0.080 \| 0.012 \| 1.31E-10 \| \| CD \| rs35320439 \| 2 \| 242737341 \| C \| 0.084 \| 0.014 \| 9.89E-10 \| \| CD \| rs6738394 \| 2 \| 219110625 \| A \| 0.078 \| 0.012 \| 8.98E-11 \| \| CD \| rs6738490 \| 2 \| 234161583 \| C \| 0.226 \| 0.012 \| 4.26E-78 \| \| CD \| rs6740462 \| 2 \| 65667272 \| A \| 0.100 \| 0.014 \| 1.74E-12 \| \| CD \| rs7608910 \| 2 \| 61204856 \| G \| 0.121 \| 0.012 \| 2.95E-23 \| \| CD \| rs77981966 \| 2 \| 43777964 \| T \| 0.183 \| 0.022 \| 2.19E-16 \| \| CD \| rs780094 \| 2 \| 27741237 \| C \| -0.116 \| 0.012 \| 4.56E-22 \| \| CD \| rs11713774 \| 3 \| 18765978 \| C \| 0.133 \| 0.017 \| 1.09E-14 \| \| CD \| rs3197999 \| 3 \| 49721532 \| A \| 0.155 \| 0.013 \| 2.05E-33 \| \| CD \| rs6827756 \| 4 \| 123184411 \| C \| -0.079 \| 0.013 \| 3.27E-10 \| \| CD \| rs7438704 \| 4 \| 48363245 \| G \| 0.084 \| 0.013 \| 3.42E-11 \| \| CD \| rs1363907 \| 5 \| 96252803 \| A \| 0.103 \| 0.013 \| 3.89E-16 \| \| CD \| rs17622378 \| 5 \| 131778452 \| G \| 0.190 \| 0.012 \| 7.17E-56 \| \| CD \| rs181826 \| 5 \| 141526057 \| A \| 0.100 \| 0.013 \| 4.53E-15 \| \| CD \| rs34804116 \| 5 \| 72539850 \| A \| -0.094 \| 0.013 \| 1.27E-13 \| \| CD \| rs3776414 \| 5 \| 10689562 \| G \| 0.089 \| 0.012 \| 5.04E-13 \| \| CD \| rs4703855 \| 5 \| 71693899 \| T \| -0.073 \| 0.013 \| 3.03E-08 \| \| CD \| rs56163845 \| 5 \| 173373948 \| G \| -0.092 \| 0.013 \| 9.40E-12 \| \| CD \| rs6556417 \| 5 \| 158823786 \| G \| 0.155 \| 0.017 \| 2.05E-19 \| \| CD \| rs71624119 \| 5 \| 55440730 \| A \| -0.092 \| 0.015 \| 6.57E-10 \| \| CD \| rs7711427 \| 5 \| 40414886 \| C \| 0.248 \| 0.012 \| 5.17E-88 \| \| CD \| rs79980175 \| 5 \| 40521892 \| C \| -0.134 \| 0.018 \| 1.70E-13 \| \| CD \| rs11152949 \| 6 \| 106449085 \| G \| 0.134 \| 0.013 \| 2.18E-25 \| \| CD \| rs1267501 \| 6 \| 14715257 \| C \| 0.087 \| 0.015 \| 9.69E-09 \| \| CD \| rs1847472 \| 6 \| 90973159 \| A \| -0.085 \| 0.013 \| 1.09E-10 \| \| CD \| rs212388 \| 6 \| 159490436 \| T \| -0.102 \| 0.012 \| 1.80E-16 \| \| CD \| rs3129871 \| 6 \| 32406342 \| C \| -0.088 \| 0.013 \| 1.80E-11 \| \| CD \| rs438475 \| 6 \| 32186245? \| A \| 0.159 \| 0.017 \| 3.42E-20 \| \| CD \| rs6456426 \| 6 \| 21438889 \| A \| -0.099 \| 0.012 \| 1.37E-16 \| \| CD \| rs6908425 \| 6 \| 20728731 \| C \| 0.104 \| 0.015 \| 4.81E-12 \| \| CD \| rs7773324 \| 6 \| 382559 \| A \| 0.079 \| 0.013 \| 1.06E-09 \| \| CD \| rs9264942 \| 6 \| 31274380 \| C \| 0.151 \| 0.013 \| 6.78E-32 \| \| CD \| rs9457247 \| 6 \| 167392174 \| T \| 0.124 \| 0.012 \| 2.08E-23 \| \| CD \| rs9491892 \| 6 \| 128280358 \| G \| 0.138 \| 0.016 \| 3.80E-17 \| \| CD \| rs1456896 \| 7 \| 50304461 \| T \| 0.098 \| 0.013 \| 1.03E-13 \| \| CD \| rs2395022 \| 7 \| 98750379 \| C \| -0.177 \| 0.028 \| 3.13E-10 \| \| CD \| rs2538470 \| 7 \| 148220448 \| G \| -0.075 \| 0.012 \| 1.05E-09 \| \| CD \| rs3801810 \| 7 \| 26892531 \| A \| 0.105 \| 0.014 \| 6.63E-14 \| \| CD \| rs7786444 \| 7 \| 28154384 \| T \| 0.112 \| 0.018 \| 9.83E-10 \| \| CD \| rs6651252 \| 8 \| 129567181 \| C \| -0.149 \| 0.018 \| 3.86E-16 \| \| CD \| rs7015630 \| 8 \| 90875918 \| C \| -0.084 \| 0.014 \| 9.00E-10 \| \| CD \| rs921720 \| 8 \| 126534671 \| G \| 0.118 \| 0.012 \| 1.12E-21 \| \| CD \| rs10758669 \| 9 \| 4981602 \| A \| -0.150 \| 0.012 \| 4.19E-34 \| \| CD \| rs11793497 \| 9 \| 139271850 \| G \| 0.169 \| 0.012 \| 9.80E-44 \| \| CD \| rs7848647 \| 9 \| 117569046 \| C \| 0.141 \| 0.013 \| 1.55E-27 \| \| CD \| rs1250573 \| 10 \| 81042475 \| A \| -0.142 \| 0.013 \| 5.86E-26 \| \| CD \| rs12766391 \| 10 \| 64441204 \| A \| 0.187 \| 0.012 \| 9.59E-53 \| \| CD \| rs2227551 \| 10 \| 75669190 \| T \| 0.099 \| 0.014 \| 4.72E-13 \| \| CD \| rs2790241 \| 10 \| 60015313 \| T \| -0.097 \| 0.015 \| 3.29E-10 \| \| CD \| rs303429 \| 10 \| 30708441 \| T \| 0.076 \| 0.012 \| 8.38E-10 \| \| CD \| rs34779708 \| 10 \| 35466185 \| G \| 0.134 \| 0.012 \| 1.90E-27 \| \| CD \| rs61839660 \| 10 \| 6094697 \| T \| 0.148 \| 0.020 \| 3.19E-13 \| \| CD \| rs7085798 \| 10 \| 101288347 \| A \| -0.174 \| 0.012 \| 1.53E-47 \| \| CD \| rs11236797 \| 11 \| 76299649 \| A \| 0.181 \| 0.012 \| 8.54E-51 \| \| CD \| rs12796489 \| 11 \| 3059360 \| A \| -0.792 \| 0.053 \| 4.96E-51 \| \| CD \| rs34787213 \| 11 \| 60799046 \| T \| -0.150 \| 0.018 \| 2.85E-16 \| \| CD \| rs559928 \| 11 \| 64150370 \| C \| 0.099 \| 0.016 \| 3.75E-10 \| \| CD \| rs11175963 \| 12 \| 40702771 \| T \| -0.158 \| 0.024 \| 4.83E-11 \| \| CD \| rs28999107 \| 12 \| 6493100 \| T \| 0.086 \| 0.013 \| 1.29E-11 \| \| CD \| rs3184504 \| 12 \| 111884608 \| C \| -0.068 \| 0.012 \| 1.71E-08 \| \| CD \| rs76906269 \| 12 \| 40607709 \| G \| 0.394 \| 0.037 \| 1.75E-26 \| \| CD \| rs7969592 \| 12 \| 68579649 \| G \| -0.073 \| 0.012 \| 1.04E-09 \| \| CD \| rs6561151 \| 13 \| 44484706 \| A \| 0.147 \| 0.014 \| 4.68E-25 \| \| CD \| rs915286 \| 13 \| 40695992 \| A \| 0.067 \| 0.012 \| 2.59E-08 \| \| CD \| rs9554587 \| 13 \| 100040654 \| G \| -0.095 \| 0.015 \| 8.29E-11 \| \| CD \| rs9594766 \| 13 \| 43040043 \| A \| -0.074 \| 0.012 \| 1.39E-09 \| \| CD \| rs11159833 \| 14 \| 88476004 \| T \| 0.155 \| 0.021 \| 7.59E-14 \| \| CD \| rs1569328 \| 14 \| 75741751 \| T \| -0.109 \| 0.017 \| 6.47E-11 \| \| CD \| rs17293632 \| 15 \| 67442596 \| T \| 0.128 \| 0.014 \| 3.70E-20 \| \| CD \| rs72727394 \| 15 \| 38847022 \| T \| 0.103 \| 0.015 \| 5.28E-12 \| \| CD \| rs11117431 \| 16 \| 86015316 \| G \| -0.149 \| 0.016 \| 1.09E-19 \| \| CD \| rs1646019 \| 16 \| 11359680 \| T \| -0.111 \| 0.013 \| 8.62E-17 \| \| CD \| rs2270395 \| 16 \| 50846832 \| T \| 0.124 \| 0.014 \| 8.93E-18 \| \| CD \| rs26528 \| 16 \| 28517709 \| C \| 0.120 \| 0.012 \| 1.29E-22 \| \| CD \| rs6500315 \| 16 \| 50508101 \| G \| 0.146 \| 0.015 \| 2.18E-23 \| \| CD \| rs7194886 \| 16 \| 50725193 \| T \| -0.227 \| 0.012 \| 1.42E-77 \| \| CD \| rs1292053 \| 17 \| 57963537 \| G \| 0.091 \| 0.012 \| 1.75E-14 \| \| CD \| rs12949918 \| 17 \| 40526273 \| C \| -0.104 \| 0.012 \| 3.47E-17 \| \| CD \| rs4795397 \| 17 \| 38023745 \| G \| 0.132 \| 0.012 \| 3.84E-28 \| \| CD \| rs9889296 \| 17 \| 32570547 \| A \| -0.143 \| 0.014 \| 2.96E-25 \| \| CD \| rs2847278 \| 18 \| 12778715 \| T \| -0.167 \| 0.016 \| 6.46E-26 \| \| CD \| rs7236492 \| 18 \| 77220616 \| T \| -0.100 \| 0.017 \| 9.09E-09 \| \| CD \| rs17694108 \| 19 \| 33731551 \| A \| 0.080 \| 0.013 \| 3.29E-09 \| \| CD \| rs2024092 \| 19 \| 1124031 \| A \| 0.148 \| 0.014 \| 7.13E-25 \| \| CD \| rs35164067 \| 19 \| 10525181 \| A \| -0.143 \| 0.016 \| 3.19E-20 \| \| CD \| rs516246 \| 19 \| 49206172 \| T \| 0.115 \| 0.012 \| 1.33E-20 \| \| CD \| rs640466 \| 19 \| 34670725 \| C \| -0.076 \| 0.012 \| 1.31E-09 \| \| CD \| rs259964 \| 20 \| 57824309 \| G \| -0.071 \| 0.012 \| 2.08E-09 \| \| CD \| rs6062496 \| 20 \| 62329099 \| A \| 0.120 \| 0.012 \| 3.82E-22 \| \| CD \| rs6074022 \| 20 \| 44740196 \| T \| -0.096 \| 0.014 \| 2.70E-12 \| \| CD \| rs6111031 \| 20 \| 1682037 \| T \| -0.282 \| 0.018 \| 9.61E-55 \| \| CD \| rs1297258 \| 21 \| 16806709 \| T \| -0.127 \| 0.012 \| 2.11E-25 \| \| CD \| rs2284553 \| 21 \| 34776695 \| G \| 0.103 \| 0.012 \| 5.63E-17 \| \| CD \| rs8127691 \| 21 \| 45614860 \| C \| -0.123 \| 0.012 \| 4.48E-24 \| \| CD \| rs2413583 \| 22 \| 39659773 \| T \| -0.210 \| 0.017 \| 7.72E-36 \| \| CD \| rs727563 \| 22 \| 41867377 \| T \| -0.092 \| 0.014 \| 1.88E-10 \|   Abbreviations: CD, crohn's disease; Chr, chromosome;IBD, inflammatory bowel disease; SE, standard error; SNP, single nucleotide polymorphism; UC, ulcerative colitis |
| --- | --- | --- | --- | --- | --- | --- | --- | --- | --- | --- | --- | --- | --- | --- | --- | --- | --- | --- | --- | --- | --- | --- | --- | --- | --- | --- | --- | --- | --- | --- | --- | --- | --- | --- | --- | --- | --- | --- | --- | --- | --- | --- | --- | --- | --- | --- | --- | --- | --- | --- | --- | --- | --- | --- | --- | --- | --- | --- | --- | --- | --- | --- | --- | --- | --- | --- | --- | --- | --- | --- | --- | --- | --- | --- | --- | --- | --- | --- | --- | --- | --- | --- | --- | --- | --- | --- | --- | --- | --- | --- | --- | --- | --- | --- | --- | --- | --- | --- | --- | --- | --- | --- | --- | --- | --- | --- | --- | --- | --- | --- | --- | --- | --- | --- | --- | --- | --- | --- | --- | --- | --- | --- | --- | --- | --- | --- | --- | --- | --- | --- | --- | --- | --- | --- | --- | --- | --- | --- | --- | --- | --- | --- | --- | --- | --- | --- | --- | --- | --- | --- | --- | --- | --- | --- | --- | --- | --- | --- | --- | --- | --- | --- | --- | --- | --- | --- | --- | --- | --- | --- | --- | --- | --- | --- | --- | --- | --- | --- | --- | --- | --- | --- | --- | --- | --- | --- | --- | --- | --- | --- | --- | --- | --- | --- | --- | --- | --- | --- | --- | --- | --- | --- | --- | --- | --- | --- | --- | --- | --- | --- | --- | --- | --- | --- | --- | --- | --- | --- | --- | --- | --- | --- | --- | --- | --- | --- | --- | --- | --- | --- | --- | --- | --- | --- | --- | --- | --- | --- | --- | --- | --- | --- | --- | --- | --- | --- | --- | --- | --- | --- | --- | --- | --- | --- | --- | --- | --- | --- | --- | --- | --- | --- | --- | --- | --- | --- | --- | --- | --- | --- | --- | --- | --- | --- | --- | --- | --- | --- | --- | --- | --- | --- | --- | --- | --- | --- | --- | --- | --- | --- | --- | --- | --- | --- | --- | --- | --- | --- | --- | --- | --- | --- | --- | --- | --- | --- | --- | --- | --- | --- | --- | --- | --- | --- | --- | --- | --- | --- | --- | --- | --- | --- | --- | --- | --- | --- | --- | --- | --- | --- | --- | --- | --- | --- | --- | --- | --- | --- | --- | --- | --- | --- | --- | --- | --- | --- | --- | --- | --- | --- | --- | --- | --- | --- | --- | --- | --- | --- | --- | --- | --- | --- | --- | --- | --- | --- | --- | --- | --- | --- | --- | --- | --- | --- | --- | --- | --- | --- | --- | --- | --- | --- | --- | --- | --- | --- | --- | --- | --- | --- | --- | --- | --- | --- | --- | --- | --- | --- | --- | --- | --- | --- | --- | --- | --- | --- | --- | --- | --- | --- | --- | --- | --- | --- | --- | --- | --- | --- | --- | --- | --- | --- | --- | --- | --- | --- | --- | --- | --- | --- | --- | --- | --- | --- | --- | --- | --- | --- | --- | --- | --- | --- | --- | --- | --- | --- | --- | --- | --- | --- | --- | --- | --- | --- | --- | --- | --- | --- | --- | --- | --- | --- | --- | --- | --- | --- | --- | --- | --- | --- | --- | --- | --- | --- | --- | --- | --- | --- | --- | --- | --- | --- | --- | --- | --- | --- | --- | --- | --- | --- | --- | --- | --- | --- | --- | --- | --- | --- | --- | --- | --- | --- | --- | --- | --- | --- | --- | --- | --- | --- | --- | --- | --- | --- | --- | --- | --- | --- | --- | --- | --- | --- | --- | --- | --- | --- | --- | --- | --- | --- | --- | --- | --- | --- | --- | --- | --- | --- | --- | --- | --- | --- | --- | --- | --- | --- | --- | --- | --- | --- | --- | --- | --- | --- | --- | --- | --- | --- | --- | --- | --- | --- | --- | --- | --- | --- | --- | --- | --- | --- | --- | --- | --- | --- | --- | --- | --- | --- | --- | --- | --- | --- | --- | --- | --- | --- | --- | --- | --- | --- | --- | --- | --- | --- | --- | --- | --- | --- | --- | --- | --- | --- | --- | --- | --- | --- | --- | --- | --- | --- | --- | --- | --- | --- | --- | --- | --- | --- | --- | --- | --- | --- | --- | --- | --- | --- | --- | --- | --- | --- | --- | --- | --- | --- | --- | --- | --- | --- | --- | --- | --- | --- | --- | --- | --- | --- | --- | --- | --- | --- | --- | --- | --- | --- | --- | --- | --- | --- | --- | --- | --- | --- | --- | --- | --- | --- | --- | --- | --- | --- | --- | --- | --- | --- | --- | --- | --- | --- | --- | --- | --- | --- | --- | --- | --- | --- | --- | --- | --- | --- | --- | --- | --- | --- | --- | --- | --- | --- | --- | --- | --- | --- | --- | --- | --- | --- | --- | --- | --- | --- | --- | --- | --- | --- | --- | --- | --- | --- | --- | --- | --- | --- | --- | --- | --- | --- | --- | --- | --- | --- | --- | --- | --- | --- | --- | --- | --- | --- | --- | --- | --- | --- | --- | --- | --- | --- | --- | --- | --- | --- | --- | --- | --- | --- | --- | --- | --- | --- | --- | --- | --- | --- | --- | --- | --- | --- | --- | --- | --- | --- | --- | --- | --- | --- | --- | --- | --- | --- | --- | --- | --- | --- | --- | --- | --- | --- | --- | --- | --- | --- | --- | --- | --- | --- | --- | --- | --- | --- | --- | --- | --- | --- | --- | --- | --- | --- | --- | --- | --- | --- | --- | --- | --- | --- | --- | --- | --- | --- | --- | --- | --- | --- | --- | --- | --- | --- | --- | --- | --- | --- | --- | --- | --- | --- | --- | --- | --- | --- | --- | --- | --- | --- | --- | --- | --- | --- | --- | --- | --- | --- | --- | --- | --- | --- | --- | --- | --- | --- | --- | --- | --- | --- | --- | --- | --- | --- | --- | --- | --- | --- | --- | --- | --- | --- | --- | --- | --- | --- | --- | --- | --- | --- | --- | --- | --- | --- | --- | --- | --- | --- | --- | --- | --- | --- | --- | --- | --- | --- | --- | --- | --- | --- | --- | --- | --- | --- | --- | --- | --- | --- | --- | --- | --- | --- | --- | --- | --- | --- | --- | --- | --- | --- | --- | --- | --- | --- | --- | --- | --- | --- | --- | --- | --- | --- | --- | --- | --- | --- | --- | --- | --- | --- | --- | --- | --- | --- | --- | --- | --- | --- | --- | --- | --- | --- | --- | --- | --- | --- | --- | --- | --- | --- | --- | --- | --- | --- | --- | --- | --- | --- | --- | --- | --- | --- | --- | --- | --- | --- | --- | --- | --- | --- | --- | --- | --- | --- | --- | --- | --- | --- | --- | --- | --- | --- | --- | --- | --- | --- | --- | --- | --- | --- | --- | --- | --- | --- | --- | --- | --- | --- | --- | --- | --- | --- | --- | --- | --- | --- | --- | --- | --- | --- | --- | --- | --- | --- | --- | --- | --- | --- | --- | --- | --- | --- | --- | --- | --- | --- | --- | --- | --- | --- | --- | --- | --- | --- | --- | --- | --- | --- | --- | --- | --- | --- | --- | --- | --- | --- | --- | --- | --- | --- | --- | --- | --- | --- | --- | --- | --- | --- | --- | --- | --- | --- | --- | --- | --- | --- | --- | --- | --- | --- | --- | --- | --- | --- | --- | --- | --- | --- | --- | --- | --- | --- | --- | --- | --- | --- | --- | --- | --- | --- | --- | --- | --- | --- | --- | --- | --- | --- | --- | --- | --- | --- | --- | --- | --- | --- | --- | --- | --- | --- | --- | --- | --- | --- | --- | --- | --- | --- | --- | --- | --- | --- | --- | --- | --- | --- | --- | --- | --- | --- | --- | --- | --- | --- | --- | --- | --- | --- | --- | --- | --- | --- | --- | --- | --- | --- | --- | --- | --- | --- | --- | --- | --- | --- | --- | --- | --- | --- | --- | --- | --- | --- | --- | --- | --- | --- | --- | --- | --- | --- | --- | --- | --- | --- | --- | --- | --- | --- | --- | --- | --- | --- | --- | --- | --- | --- | --- | --- | --- | --- | --- | --- | --- | --- | --- | --- | --- | --- | --- | --- | --- | --- | --- | --- | --- | --- | --- | --- | --- | --- | --- | --- | --- | --- | --- | --- | --- | --- | --- | --- | --- | --- | --- | --- | --- | --- | --- | --- | --- | --- | --- | --- | --- | --- | --- | --- | --- | --- | --- | --- | --- | --- | --- | --- | --- | --- | --- | --- | --- | --- | --- | --- | --- | --- | --- | --- | --- | --- | --- | --- | --- | --- | --- | --- | --- | --- | --- | --- | --- | --- | --- | --- | --- | --- | --- | --- | --- | --- | --- | --- | --- | --- | --- | --- | --- | --- | --- | --- | --- | --- | --- | --- | --- | --- | --- | --- | --- | --- | --- | --- | --- | --- | --- | --- | --- | --- | --- | --- | --- | --- | --- | --- | --- | --- | --- | --- | --- | --- | --- | --- | --- | --- | --- | --- | --- | --- | --- | --- | --- | --- | --- | --- | --- | --- | --- | --- | --- | --- | --- | --- | --- | --- | --- | --- | --- | --- | --- | --- | --- | --- | --- | --- | --- | --- | --- | --- | --- | --- | --- | --- | --- | --- | --- | --- | --- | --- | --- | --- | --- | --- | --- | --- | --- | --- | --- | --- | --- | --- | --- | --- | --- | --- | --- | --- | --- | --- | --- | --- | --- | --- | --- | --- | --- | --- | --- | --- | --- | --- | --- | --- | --- | --- | --- | --- | --- | --- | --- | --- | --- | --- | --- | --- | --- | --- | --- | --- | --- | --- | --- | --- | --- | --- | --- | --- | --- | --- | --- | --- | --- | --- | --- | --- | --- | --- | --- | --- | --- | --- | --- | --- | --- | --- | --- | --- | --- | --- | --- | --- | --- | --- | --- | --- | --- | --- | --- | --- | --- | --- | --- | --- | --- | --- | --- | --- | --- | --- | --- | --- | --- | --- | --- | --- | --- | --- | --- | --- | --- | --- | --- | --- | --- | --- | --- | --- | --- | --- | --- | --- | --- | --- | --- | --- | --- | --- | --- | --- | --- | --- | --- | --- | --- | --- | --- | --- | --- | --- | --- | --- | --- | --- | --- | --- | --- | --- | --- | --- | --- | --- | --- | --- | --- | --- | --- | --- | --- | --- | --- | --- | --- | --- | --- | --- | --- | --- | --- | --- | --- | --- | --- | --- | --- | --- | --- | --- | --- | --- | --- | --- | --- | --- | --- | --- | --- | --- | --- | --- | --- | --- | --- | --- | --- | --- | --- | --- | --- | --- | --- | --- | --- | --- | --- | --- | --- | --- | --- | --- | --- | --- | --- | --- | --- | --- | --- | --- | --- | --- | --- | --- | --- | --- | --- | --- | --- | --- | --- | --- | --- | --- | --- | --- | --- | --- | --- | --- | --- | --- | --- | --- | --- | --- | --- | --- | --- | --- | --- | --- | --- | --- | --- | --- | --- | --- | --- | --- | --- | --- | --- | --- | --- | --- | --- | --- | --- | --- | --- | --- | --- | --- | --- | --- | --- | --- | --- | --- | --- | --- | --- | --- | --- | --- | --- | --- | --- | --- | --- | --- | --- | --- | --- | --- | --- | --- | --- | --- | --- | --- | --- | --- | --- | --- | --- | --- | --- | --- | --- | --- | --- | --- | --- | --- | --- | --- | --- | --- | --- | --- | --- | --- | --- | --- | --- | --- | --- | --- | --- | --- | --- | --- | --- | --- | --- | --- | --- | --- | --- | --- | --- | --- | --- | --- | --- | --- | --- | --- | --- | --- | --- | --- | --- | --- | --- | --- | --- | --- | --- | --- | --- | --- | --- | --- | --- | --- | --- | --- | --- | --- | --- | --- | --- | --- | --- | --- | --- | --- | --- | --- | --- | --- | --- | --- | --- | --- | --- | --- | --- | --- | --- | --- | --- | --- | --- | --- | --- | --- | --- | --- | --- | --- | --- | --- | --- | --- | --- | --- | --- | --- | --- | --- | --- | --- | --- | --- | --- | --- | --- | --- | --- | --- | --- | --- | --- | --- | --- | --- | --- | --- | --- | --- | --- | --- | --- | --- | --- | --- | --- | --- | --- | --- | --- | --- | --- | --- | --- | --- | --- | --- | --- | --- | --- | --- | --- | --- | --- | --- | --- | --- | --- | --- | --- | --- | --- | --- | --- | --- | --- | --- | --- | --- | --- | --- | --- | --- | --- | --- | --- | --- | --- | --- | --- | --- | --- | --- | --- | --- | --- | --- | --- | --- | --- | --- | --- | --- | --- | --- | --- | --- | --- | --- | --- | --- | --- | --- | --- | --- | --- | --- | --- | --- | --- | --- | --- | --- | --- | --- | --- | --- | --- | --- | --- | --- | --- | --- | --- | --- | --- | --- | --- | --- | --- | --- | --- | --- | --- | --- | --- | --- | --- | --- | --- | --- | --- | --- | --- | --- | --- | --- | --- | --- | --- | --- | --- | --- | --- | --- | --- | --- | --- | --- | --- | --- | --- | --- | --- | --- | --- | --- | --- | --- | --- | --- | --- | --- | --- | --- | --- | --- | --- | --- | --- | --- | --- | --- | --- | --- | --- | --- | --- | --- | --- | --- | --- | --- | --- | --- | --- | --- | --- | --- | --- | --- | --- | --- | --- | --- | --- | --- | --- | --- | --- | --- | --- | --- | --- | --- | --- | --- | --- | --- | --- | --- | --- | --- | --- | --- | --- | --- | --- | --- | --- | --- | --- | --- | --- | --- | --- | --- | --- | --- | --- | --- | --- | --- | --- | --- | --- | --- | --- | --- | --- | --- | --- | --- | --- | --- | --- | --- | --- | --- | --- | --- | --- | --- | --- | --- | --- | --- | --- | --- | --- | --- | --- | --- | --- | --- | --- | --- | --- | --- | --- | --- | --- | --- | --- | --- | --- | --- | --- | --- | --- | --- | --- | --- | --- | --- | --- | --- | --- | --- | --- | --- | --- | --- | --- | --- | --- | --- | --- | --- | --- | --- | --- | --- | --- | --- | --- | --- | --- | --- | --- | --- | --- | --- | --- | --- | --- | --- | --- | --- | --- | --- | --- | --- | --- | --- | --- | --- | --- | --- | --- | --- | --- | --- | --- | --- | --- | --- | --- | --- | --- | --- | --- | --- | --- | --- | --- | --- | --- | --- | --- | --- | --- | --- | --- | --- | --- | --- | --- | --- | --- | --- | --- | --- | --- | --- | --- | --- | --- | --- | --- | --- | --- | --- | --- | --- | --- | --- | --- | --- | --- | --- | --- | --- | --- | --- | --- | --- | --- | --- | --- | --- | --- | --- | --- | --- | --- | --- | --- | --- | --- | --- | --- | --- | --- | --- | --- | --- | --- | --- | --- | --- | --- | --- | --- | --- | --- | --- | --- | --- | --- | --- | --- | --- | --- | --- | --- | --- | --- | --- | --- | --- | --- | --- | --- | --- | --- | --- | --- | --- | --- | --- | --- | --- | --- | --- | --- | --- | --- | --- | --- | --- | --- | --- | --- | --- | --- | --- | --- | --- | --- | --- | --- | --- | --- | --- | --- | --- | --- | --- | --- | --- | --- | --- | --- | --- | --- | --- | --- | --- | --- | --- | --- | --- | --- | --- | --- | --- | --- | --- | --- | --- | --- | --- | --- | --- | --- | --- | --- | --- | --- | --- | --- | --- | --- | --- | --- | --- | --- | --- | --- | --- | --- | --- | --- | --- | --- | --- | --- | --- | --- | --- | --- | --- | --- | --- | --- | --- | --- | --- | --- | --- | --- | --- | --- | --- | --- | --- | --- | --- | --- | --- | --- | --- | --- | --- | --- | --- | --- | --- | --- | --- | --- | --- | --- | --- | --- | --- | --- | --- | --- | --- | --- | --- | --- | --- | --- | --- | --- | --- | --- | --- | --- | --- | --- | --- | --- | --- | --- | --- | --- | --- | --- | --- | --- | --- | --- | --- | --- | --- | --- | --- | --- | --- | --- | --- | --- | --- | --- | --- | --- | --- | --- | --- | --- | --- | --- | --- | --- | --- | --- | --- | --- | --- | --- | --- | --- | --- | --- | --- | --- | --- | --- | --- | --- | --- | --- | --- | --- | --- | --- | --- | --- | --- | --- | --- | --- | --- | --- | --- | --- | --- | --- | --- | --- | --- | --- | --- | --- | --- | --- | --- | --- | --- | --- | --- | --- | --- | --- | --- | --- | --- | --- | --- | --- | --- | --- | --- | --- | --- | --- | --- | --- | --- | --- | --- | --- | --- | --- | --- | --- | --- | --- | --- | --- | --- | --- | --- | --- | --- | --- | --- | --- | --- | --- | --- | --- | --- | --- | --- | --- | --- | --- | --- | --- | --- | --- | --- | --- | --- | --- | --- | --- | --- | --- | --- | --- | --- | --- | --- | --- | --- | --- | --- | --- | --- | --- | --- | --- | --- | --- | --- | --- | --- | --- | --- | --- | --- | --- | --- | --- | --- | --- | --- | --- | --- | --- | --- | --- | --- | --- | --- | --- | --- | --- | --- | --- | --- | --- | --- | --- | --- | --- | --- | --- | --- | --- | --- | --- | --- | --- | --- | --- | --- | --- | --- | --- | --- | --- | --- | --- | --- | --- | --- | --- | --- | --- | --- | --- | --- | --- | --- | --- | --- | --- | --- | --- | --- | --- | --- | --- | --- | --- | --- | --- | --- | --- | --- | --- | --- | --- | --- | --- | --- | --- | --- | --- | --- | --- | --- | --- | --- | --- | --- | --- | --- | --- | --- | --- | --- | --- | --- | --- | --- |

**Supplementary Table 2** Effect estimates of the associations between 196 bacterial traits and risk of inflammatory bowel disease in MR analyses

| Gut microbiota | R^2^ | F statistic | Methods | N.SNP | OR | 95% CI | *P*-value | Qrs |
| --- | --- | --- | --- | --- | --- | --- | --- | --- |
| Phylum |  |  |  |  |  |  |  |  |
| Actinobacteria | 2.80% | 26.43 |  |  |  |  |  |  |
|  |  |  | Inverse-variance weighted (fixed) | 20 | 0.94 | 0.81-1.09 | 0.420 | 0.179 |
|  |  |  | Weighted median | 20 | 0.87 | 0.71-1.08 | 0.206 |  |
|  |  |  | MR-PRESSO test | 20 | 0.94 | 0.80-1.11 | 0.486 |  |
|  |  |  | MR-Egger | 20 | / | / | 0.889* |  |
| Bacteroidetes | 2.21% | 29.61 |  |  |  |  |  |  |
|  |  |  | Inverse-variance weighted (fixed) | 14 | 0.95 | 0.80-1.12 | 0.533 | 0.231 |
|  |  |  | Weighted median | 14 | 0.96 | 0.76-1.22 | 0.761 |  |
|  |  |  | MR-PRESSO test | 14 | 0.95 | 0.79-1.14 | 0.588 |  |
|  |  |  | MR-Egger | 14 | / | / | 0.031* |  |
| Cyanobacteria | 3.53% | 67.16 |  |  |  |  |  |  |
|  |  |  | Inverse-variance weighted (fixed) | 10 | 0.93 | 0.82-1.06 | 0.292 | 0.585 |
|  |  |  | Weighted median | 10 | 0.85 | 0.71-1.01 | 0.061 |  |
|  |  |  | MR-PRESSO test | 10 | 0.93 | 0.83-1.05 | 0.278 |  |
|  |  |  | MR-Egger | 10 | / | / | 0.913* |  |
| Euryarchaeota | 7.39% | 112.50 |  |  |  |  |  |  |
|  |  |  | Inverse-variance weighted (fixed) | 13 | 0.98 | 0.90-1.07 | 0.603 | 0.237 |
|  |  |  | Weighted median | 13 | 1.01 | 0.90-1.15 | 0.823 |  |
|  |  |  | MR-PRESSO test | 13 | 0.98 | 0.89-1.08 | 0.651 |  |
|  |  |  | MR-Egger | 13 | / | / | 0.077* |  |
| Firmicutes | 2.49% | 24.65 |  |  |  |  |  |  |
|  |  |  | Inverse-variance weighted (fixed) | 19 | 0.98 | 0.84-1.15 | 0.836 | 0.125 |
|  |  |  | Weighted median | 19 | 1.01 | 0.81-1.27 | 0.914 |  |
|  |  |  | MR-PRESSO test | 19 | 0.98 | 0.82-1.18 | 0.862 |  |
|  |  |  | MR-Egger | 19 | / | / | 0.609* |  |
| Lentisphaerae | 5.25% | 92.39 |  |  |  |  |  |  |
|  |  |  | Inverse-variance weighted (fixed) | 11 | 0.88 | 0.79-0.98 | 0.020 | 0.755 |
|  |  |  | Weighted median | 11 | 0.86 | 0.75-0.99 | 0.037 |  |
|  |  |  | MR-PRESSO test | 11 | 0.88 | 0.81-0.96 | 0.017 |  |
|  |  |  | MR-Egger | 11 | / | / | 0.776* |  |
| Proteobacteria | 1.39% | 28.61 |  |  |  |  |  |  |
|  |  |  | Inverse-variance weighted (random) | 13 | 1.04 | 0.78-1.38 | 0.812 | 0.006 |
|  |  |  | Weighted median | 13 | 1.01 | 0.77-1.32 | 0.964 |  |
|  |  |  | MR-PRESSO test | 13 | 1.04 | 0.78-1.38 | 0.816 |  |
|  |  |  | Outlier corrected (MR-PRESSO) | 11 | 1.03 | 0.87-1.21 | 0.756 |  |
|  |  |  | MR-Egger | 13 | / | / | 0.379* |  |
| Tenericutes | 2.60% | 40.77 |  |  |  |  |  |  |
|  |  |  | Inverse-variance weighted (fixed) | 12 | 1.02 | 0.88-1.18 | 0.820 | 0.355 |
|  |  |  | Weighted median | 12 | 0.91 | 0.73-1.12 | 0.376 |  |
|  |  |  | MR-PRESSO test | 12 | 1.02 | 0.87-1.19 | 0.833 |  |
|  |  |  | MR-Egger | 12 | / | / | 0.801* |  |
| Verrucomicrobia | 2.42% | 37.94 |  |  |  |  |  |  |
|  |  |  | Inverse-variance weighted (fixed) | 12 | 1.00 | 0.86-1.17 | 0.996 | 0.245 |
|  |  |  | Weighted median | 12 | 1.05 | 0.85-1.30 | 0.629 |  |
|  |  |  | MR-PRESSO test | 12 | 1.00 | 0.84-1.19 | 0.996 |  |
|  |  |  | MR-Egger | 12 | / | / | 0.482* |  |
| Class |  |  |  |  |  |  |  |  |
| Actinobacteria | 4.64% | 38.76 |  |  |  |  |  |  |
|  |  |  | Inverse-variance weighted (random) | 23 | 0.91 | 0.78-1.07 | 0.257 | 0.037 |
|  |  |  | Weighted median | 23 | 0.96 | 0.80-1.15 | 0.636 |  |
|  |  |  | MR-PRESSO test | 23 | 0.91 | 0.78-1.07 | 0.269 |  |
|  |  |  | MR-Egger | 23 | / | / | 0.932* |  |
| Alphaproteobacteria | 2.30% | 43.24 |  |  |  |  |  |  |
|  |  |  | Inverse-variance weighted (fixed) | 10 | 1.07 | 0.91-1.26 | 0.406 | 0.877 |
|  |  |  | Weighted median | 10 | 1.15 | 0.92-1.42 | 0.214 |  |
|  |  |  | MR-PRESSO test | 10 | 1.07 | 0.96-1.20 | 0.269 |  |
|  |  |  | MR-Egger | 10 | / | / | 0.503* |  |
| Bacilli | 3.50% | 30.17 |  |  |  |  |  |  |
|  |  |  | Inverse-variance weighted (fixed) | 22 | 0.96 | 0.84-1.10 | 0.554 | 0.081 |
|  |  |  | Weighted median | 22 | 0.98 | 0.79-1.20 | 0.813 |  |
|  |  |  | MR-PRESSO test | 22 | 0.96 | 0.82-1.13 | 0.629 |  |
|  |  |  | MR-Egger | 22 | / | / | 0.532* |  |
| Bacteroidia | 2.28% | 28.54 |  |  |  |  |  |  |
|  |  |  | Inverse-variance weighted (fixed) | 15 | 0.94 | 0.80-1.10 | 0.423 | 0.312 |
|  |  |  | Weighted median | 15 | 0.88 | 0.69-1.11 | 0.271 |  |
|  |  |  | MR-PRESSO test | 15 | 0.94 | 0.79-1.11 | 0.466 |  |
|  |  |  | MR-Egger | 15 | / | / | 0.035* |  |
|  | 2.02% | 25.21 |  |  |  |  |  |  |
| Betaproteobacteria |  |  | Inverse-variance weighted (fixed) | 15 | 0.96 | 0.81-1.13 | 0.613 | 0.313 |
|  |  |  | Weighted median | 15 | 0.96 | 0.76-1.21 | 0.714 |  |
|  |  |  | MR-PRESSO test | 15 | 0.96 | 0.80-1.15 | 0.643 |  |
|  |  |  | MR-Egger | 15 | / | / | 0.413* |  |
| Clostridia | 1.97% | 21.63 |  |  |  |  |  |  |
|  |  |  | Inverse-variance weighted (fixed) | 17 | 1.13 | 0.95-1.34 | 0.173 | 0.373 |
|  |  |  | Weighted median | 17 | 1.13 | 0.88-1.43 | 0.336 |  |
|  |  |  | MR-PRESSO test | 17 | 1.13 | 0.94-1.35 | 0.207 |  |
|  |  |  | MR-Egger | 17 | / | / | 0.604* |  |
| Coriobacteriia | 2.99% | 26.93 |  |  |  |  |  |  |
|  |  |  | Inverse-variance weighted (fixed) | 21 | 1.03 | 0.89-1.18 | 0.715 | 0.610 |
|  |  |  | Weighted median | 21 | 1.04 | 0.84-1.29 | 0.732 |  |
|  |  |  | MR-PRESSO test | 21 | 1.03 | 0.90-1.17 | 0.701 |  |
|  |  |  | MR-Egger | 21 | / | / | 0.692* |  |
| Deltaproteobacteria | 2.22% | 29.78 |  |  |  |  |  |  |
|  |  |  | Inverse-variance weighted (fixed) | 14 | 1.08 | 0.92-1.27 | 0.324 | 0.799 |
|  |  |  | Weighted median | 14 | 1.11 | 0.89-1.38 | 0.358 |  |
|  |  |  | MR-PRESSO test | 14 | 1.08 | 0.95-1.23 | 0.248 |  |
|  |  |  | MR-Egger | 14 | / | / | 0.661* |  |
| Erysipelotrichia | 1.65% | 23.71 |  |  |  |  |  |  |
|  |  |  | Inverse-variance weighted (fixed) | 13 | 1.03 | 0.85-1.25 | 0.765 | 0.845 |
|  |  |  | Weighted median | 13 | 1.04 | 0.81-1.33 | 0.781 |  |
|  |  |  | MR-PRESSO test | 13 | 1.03 | 0.89-1.19 | 0.706 |  |
|  |  |  | MR-Egger | 13 | / | / | 0.479* |  |
| Gammaproteobacteria | 1.39% | 28.61 |  |  |  |  |  |  |
|  |  |  | Inverse-variance weighted (fixed) | 9 | 0.90 | 0.73-1.10 | 0.309 | 0.595 |
|  |  |  | Weighted median | 9 | 0.88 | 0.68-1.14 | 0.328 |  |
|  |  |  | MR-PRESSO test | 9 | 0.90 | 0.75-1.08 | 0.291 |  |
|  |  |  | MR-Egger | 9 | / | / | 0.389* |  |
| Lentisphaeria | 4.81% | 92.58 |  |  |  |  |  |  |
|  |  |  | Inverse-variance weighted (fixed) | 10 | 0.89 | 0.79-0.99 | 0.034 | 0.672 |
|  |  |  | Weighted median | 10 | 0.88 | 0.76-1.03 | 0.102 |  |
|  |  |  | MR-PRESSO test | 10 | 0.89 | 0.81-0.98 | 0.036 |  |
|  |  |  | MR-Egger | 10 | / | / | 0.687* |  |
| Melainabacteria | 5.25% | 78.13 |  |  |  |  |  |  |
|  |  |  | Inverse-variance weighted (random) | 13 | 0.98 | 0.85-1.12 | 0.728 | 0.049 |
|  |  |  | Weighted median | 13 | 0.98 | 0.85-1.13 | 0.769 |  |
|  |  |  | MR-PRESSO test | 13 | 0.98 | 0.85-1.12 | 0.734 |  |
|  |  |  | Outlier corrected (MR-PRESSO) | 12 | 0.93 | 0.84-1.03 | 0.190 |  |
|  |  |  | MR-Egger | 13 | / | / | 0.746* |  |
| Methanobacteria | 7.23% | 119.05 |  |  |  |  |  |  |
|  |  |  | Inverse-variance weighted (fixed) | 12 | 0.96 | 0.88-1.05 | 0.335 | 0.688 |
|  |  |  | Weighted median | 12 | 0.97 | 0.86-1.09 | 0.611 |  |
|  |  |  | MR-PRESSO test | 12 | 0.96 | 0.89-1.03 | 0.290 |  |
|  |  |  | MR-Egger | 12 | / | / | 0.871* |  |
| Mollicutes | 2.60% | 40.77 |  |  |  |  |  |  |
|  |  |  | Inverse-variance weighted (fixed) | 12 | 1.02 | 0.88-1.18 | 0.820 | 0.355 |
|  |  |  | Weighted median | 12 | 0.91 | 0.73-1.12 | 0.376 |  |
|  |  |  | MR-PRESSO test | 12 | 1.02 | 0.87-1.19 | 0.833 |  |
|  |  |  | MR-Egger | 12 | / | / | 0.801* |  |
| Negativicutes | 1.67% | 23.92 |  |  |  |  |  |  |
|  |  |  | Inverse-variance weighted (fixed) | 13 | 0.90 | 0.74-1.08 | 0.262 | 0.376 |
|  |  |  | Weighted median | 13 | 0.87 | 0.67-1.13 | 0.303 |  |
|  |  |  | MR-PRESSO test | 13 | 0.90 | 0.74-1.09 | 0.300 |  |
|  |  |  | MR-Egger | 13 | / | / | 0.523* |  |
| Verrucomicrobiae | 2.63% | 38.04 |  |  |  |  |  |  |
|  |  |  | Inverse-variance weighted (fixed) | 13 | 0.95 | 0.82-1.11 | 0.541 | 0.204 |
|  |  |  | Weighted median | 13 | 0.83 | 0.68-1.02 | 0.084 |  |
|  |  |  | MR-PRESSO test | 13 | 0.95 | 0.80-1.13 | 0.603 |  |
|  |  |  | MR-Egger | 13 | / | / | 0.138* |  |
| Order |  |  |  |  |  |  |  |  |
| Actinomycetales | 1.75% | 65.35 |  |  |  |  |  |  |
|  |  |  | Inverse-variance weighted (fixed) | 5 | 1.05 | 0.86-1.28 | 0.633 | 0.381 |
|  |  |  | Weighted median | 5 | 1.14 | 0.89-1.48 | 0.300 |  |
|  |  |  | MR-PRESSO test | 5 | 1.05 | 0.86-1.28 | 0.665 |  |
|  |  |  | MR-Egger | 5 | / | / | 0.905* |  |
| Bacillales | 7.14% | 128.04 |  |  |  |  |  |  |
|  |  |  | Inverse-variance weighted (fixed) | 11 | 0.94 | 0.86-1.03 | 0.199 | 0.908 |
|  |  |  | Weighted median | 11 | 0.97 | 0.86-1.08 | 0.547 |  |
|  |  |  | MR-PRESSO test | 11 | 0.94 | 0.89-1.00 | 0.091 |  |
|  |  |  | MR-Egger | 11 | / | / | 0.534* |  |
| Bacteroidales | 2.28% | 28.54 |  |  |  |  |  |  |
|  |  |  | Inverse-variance weighted (fixed) | 15 | 0.94 | 0.80-1.10 | 0.423 | 0.312 |
|  |  |  | Weighted median | 15 | 0.88 | 0.69-1.11 | 0.271 |  |
|  |  |  | MR-PRESSO test | 15 | 0.94 | 0.79-1.11 | 0.466 |  |
|  |  |  | MR-Egger | 15 | / | / | 0.035* |  |
| Bifidobacteriales | 4.44% | 34.00 |  |  |  |  |  |  |
|  |  |  | Inverse-variance weighted (fixed) | 25 | 0.92 | 0.81-1.03 | 0.157 | 0.051 |
|  |  |  | Weighted median | 25 | 0.91 | 0.75-1.10 | 0.329 |  |
|  |  |  | MR-PRESSO test | 25 | 0.92 | 0.79-1.06 | 0.261 |  |
|  |  |  | MR-Egger | 25 | / | / | 0.721* |  |
| Burkholderiales | 1.76% | 25.27 |  |  |  |  |  |  |
|  |  |  | Inverse-variance weighted (fixed) | 13 | 1.01 | 0.84-1.21 | 0.940 | 0.417 |
|  |  |  | Weighted median | 13 | 0.98 | 0.77-1.26 | 0.896 |  |
|  |  |  | MR-PRESSO test | 13 | 1.01 | 0.84-1.21 | 0.942 |  |
|  |  |  | MR-Egger | 13 | / | / | 0.224* |  |
| Clostridiales | 1.97% | 21.66 |  |  |  |  |  |  |
|  |  |  | Inverse-variance weighted (fixed) | 17 | 1.13 | 0.95-1.34 | 0.166 | 0.289 |
|  |  |  | Weighted median | 17 | 1.13 | 0.88-1.44 | 0.352 |  |
|  |  |  | MR-PRESSO test | 17 | 1.13 | 0.94-1.36 | 0.218 |  |
|  |  |  | MR-Egger | 17 | / | / | 0.499* |  |
| Coriobacteriales | 2.99% | 26.93 |  |  |  |  |  |  |
|  |  |  | Inverse-variance weighted (fixed) | 21 | 1.03 | 0.89-1.18 | 0.715 | 0.610 |
|  |  |  | Weighted median | 21 | 1.04 | 0.84-1.29 | 0.732 |  |
|  |  |  | MR-PRESSO test | 21 | 1.03 | 0.90-1.17 | 0.701 |  |
|  |  |  | MR-Egger | 21 | / | / | 0.692* |  |
| Desulfovibrionales | 2.13% | 30.69 |  |  |  |  |  |  |
|  |  |  | Inverse-variance weighted (fixed) | 13 | 1.13 | 0.96-1.33 | 0.148 | 0.890 |
|  |  |  | Weighted median | 13 | 1.13 | 0.90-1.41 | 0.302 |  |
|  |  |  | MR-PRESSO test | 13 | 1.13 | 1.00-1.27 | 0.073 |  |
|  |  |  | MR-Egger | 13 | / | / | 0.799* |  |
| Enterobacteriales | 1.84% | 31.17 |  |  |  |  |  |  |
|  |  |  | Inverse-variance weighted (fixed) | 11 | 1.15 | 0.95-1.39 | 0.143 | 0.322 |
|  |  |  | Weighted median | 11 | 1.09 | 0.85-1.40 | 0.506 |  |
|  |  |  | MR-PRESSO test | 11 | 1.15 | 0.94-1.41 | 0.202 |  |
|  |  |  | MR-Egger | 11 | / | / | 0.096* |  |
| Erysipelotrichales | 1.65% | 23.71 |  |  |  |  |  |  |
|  |  |  | Inverse-variance weighted (fixed) | 13 | 1.03 | 0.85-1.25 | 0.765 | 0.845 |
|  |  |  | Weighted median | 13 | 1.04 | 0.81-1.33 | 0.781 |  |
|  |  |  | MR-PRESSO test | 13 | 1.03 | 0.89-1.19 | 0.706 |  |
|  |  |  | MR-Egger | 13 | / | / | 0.479* |  |
| Gastranaerophilales | 4.90% | 78.66 |  |  |  |  |  |  |
|  |  |  | Inverse-variance weighted (random) | 12 | 1.02 | 0.88-1.18 | 0.807 | 0.031 |
|  |  |  | Weighted median | 12 | 0.99 | 0.85-1.14 | 0.883 |  |
|  |  |  | MR-PRESSO test | 12 | 1.02 | 0.88-1.18 | 0.812 |  |
|  |  |  | Outlier corrected (MR-PRESSO) | 11 | 0.97 | 0.86-1.09 | 0.625 |  |
|  |  |  | MR-Egger | 12 | / | / | 0.757* |  |
| Lactobacillales | 3.12% | 31.02 |  |  |  |  |  |  |
|  |  |  | Inverse-variance weighted (fixed) | 19 | 0.96 | 0.83-1.11 | 0.589 | 0.050 |
|  |  |  | Weighted median | 19 | 0.98 | 0.78-1.23 | 0.855 |  |
|  |  |  | MR-PRESSO test | 19 | 0.96 | 0.80-1.15 | 0.675 |  |
|  |  |  | MR-Egger | 19 | / | / | 0.684* |  |
| Methanobacteriales | 7.23% | 119.05 |  |  |  |  |  |  |
|  |  |  | Inverse-variance weighted (fixed) | 12 | 0.96 | 0.88-1.05 | 0.335 | 0.688 |
|  |  |  | Weighted median | 12 | 0.97 | 0.86-1.09 | 0.611 |  |
|  |  |  | MR-PRESSO test | 12 | 0.96 | 0.89-1.03 | 0.290 |  |
|  |  |  | MR-Egger | 12 | / | / | 0.871* |  |
| MollicutesRF9 | 3.80% | 42.57 |  |  |  |  |  |  |
|  |  |  | Inverse-variance weighted (fixed) | 17 | 0.97 | 0.86-1.10 | 0.645 | 0.952 |
|  |  |  | Weighted median | 17 | 0.97 | 0.82-1.14 | 0.696 |  |
|  |  |  | MR-PRESSO test | 17 | 0.97 | 0.89-1.06 | 0.521 |  |
|  |  |  | MR-Egger | 17 | / | / | 0.250* |  |
| NB1n | 7.59% | 88.52 |  |  |  |  |  |  |
|  |  |  | Inverse-variance weighted (fixed) | 17 | 1.13 | 1.03-1.23 | 0.008 | 0.110 |
|  |  |  | Weighted median | 17 | 1.10 | 0.97-1.25 | 0.131 |  |
|  |  |  | MR-PRESSO test | 17 | 1.13 | 1.01-1.25 | 0.043 |  |
|  |  |  | MR-Egger | 17 | / | / | 0.113* |  |
| Pasteurellales | 5.52% | 56.29 |  |  |  |  |  |  |
|  |  |  | Inverse-variance weighted (fixed) | 19 | 1.00 | 0.90-1.11 | 0.963 | 0.154 |
|  |  |  | Weighted median | 19 | 0.93 | 0.79-1.08 | 0.326 |  |
|  |  |  | MR-PRESSO test | 19 | 1.00 | 0.89-1.13 | 0.968 |  |
|  |  |  | MR-Egger | 19 | / | / | 0.885* |  |
| Rhodospirillales | 3.39% | 45.92 |  |  |  |  |  |  |
|  |  |  | Inverse-variance weighted (fixed) | 14 | 1.10 | 0.97-1.25 | 0.129 | 0.204 |
|  |  |  | Weighted median | 14 | 1.19 | 0.99-1.43 | 0.066 |  |
|  |  |  | MR-PRESSO test | 14 | 1.10 | 0.95-1.28 | 0.206 |  |
|  |  |  | MR-Egger | 14 | / | / | 0.285* |  |
| Selenomonadales | 1.67% | 23.92 |  |  |  |  |  |  |
|  |  |  | Inverse-variance weighted (fixed) | 13 | 0.90 | 0.74-1.08 | 0.262 | 0.376 |
|  |  |  | Weighted median | 13 | 0.87 | 0.67-1.13 | 0.303 |  |
|  |  |  | MR-PRESSO test | 13 | 0.90 | 0.74-1.09 | 0.300 |  |
|  |  |  | MR-Egger | 13 | / | / | 0.523* |  |
| Verrucomicrobiales | 2.63% | 38.04 |  |  |  |  |  |  |
|  |  |  | Inverse-variance weighted (fixed) | 13 | 0.95 | 0.82-1.11 | 0.541 | 0.204 |
|  |  |  | Weighted median | 13 | 0.83 | 0.68-1.02 | 0.084 |  |
|  |  |  | MR-PRESSO test | 13 | 0.95 | 0.80-1.13 | 0.603 |  |
|  |  |  | MR-Egger | 13 | / | / | 0.138* |  |
| Victivallales | 4.81% | 92.58 |  |  |  |  |  |  |
|  |  |  | Inverse-variance weighted (fixed) | 10 | 0.89 | 0.79-0.99 | 0.034 | 0.672 |
|  |  |  | Weighted median | 10 | 0.88 | 0.76-1.03 | 0.102 |  |
|  |  |  | MR-PRESSO test | 10 | 0.89 | 0.81-0.98 | 0.036 |  |
|  |  |  | MR-Egger | 10 | / | / | 0.687* |  |
| Family |  |  |  |  |  |  |  |  |
| Acidaminococcaceae | 1.50% | 35.00 |  |  |  |  |  |  |
|  |  |  | Inverse-variance weighted (fixed) | 8 | 0.95 | 0.78-1.16 | 0.630 | 0.872 |
|  |  |  | Weighted median | 8 | 0.98 | 0.76-1.27 | 0.883 |  |
|  |  |  | MR-PRESSO test | 8 | 0.95 | 0.83-1.09 | 0.495 |  |
|  |  |  | MR-Egger | 8 | / | / | 0.979* |  |
| Actinomycetaceae | 1.76% | 65.52 |  |  |  |  |  |  |
|  |  |  | Inverse-variance weighted (fixed) | 5 | 1.05 | 0.86-1.28 | 0.635 | 0.381 |
|  |  |  | Weighted median | 5 | 1.14 | 0.89-1.48 | 0.301 |  |
|  |  |  | MR-PRESSO test | 5 | 1.05 | 0.86-1.28 | 0.667 |  |
|  |  |  | MR-Egger | 5 | / | / | 0.900* |  |
| Alcaligenaceae | 2.77% | 26.11 |  |  |  |  |  |  |
|  |  |  | Inverse-variance weighted (fixed) | 20 | 1.01 | 0.87-1.17 | 0.861 | 0.483 |
|  |  |  | Weighted median | 20 | 1.03 | 0.84-1.27 | 0.753 |  |
|  |  |  | MR-PRESSO test | 20 | 1.01 | 0.88-1.17 | 0.862 |  |
|  |  |  | MR-Egger | 20 | / | / | 0.124* |  |
| Bacteroidaceae | 1.23% | 22.84 |  |  |  |  |  |  |
|  |  |  | Inverse-variance weighted (fixed) | 10 | 1.10 | 0.88-1.37 | 0.394 | 0.749 |
|  |  |  | Weighted median | 10 | 1.16 | 0.87-1.54 | 0.309 |  |
|  |  |  | MR-PRESSO test | 10 | 1.10 | 0.92-1.31 | 0.321 |  |
|  |  |  | MR-Egger | 10 | / | / | 0.645* |  |
| BacteroidalesS24.7 | 3.06% | 57.94 |  |  |  |  |  |  |
|  |  |  | Inverse-variance weighted (fixed) | 10 | 1.02 | 0.89-1.17 | 0.771 | 0.450 |
|  |  |  | Weighted median | 10 | 1.02 | 0.86-1.22 | 0.807 |  |
|  |  |  | MR-PRESSO test | 10 | 1.02 | 0.89-1.17 | 0.776 |  |
|  |  |  | MR-Egger | 10 | / | / | 0.147* |  |
| Bifidobacteriaceae | 4.44% | 34.00 |  |  |  |  |  |  |
|  |  |  | Inverse-variance weighted (fixed) | 25 | 0.92 | 0.81-1.03 | 0.157 | 0.051 |
|  |  |  | Weighted median | 25 | 0.91 | 0.75-1.10 | 0.329 |  |
|  |  |  | MR-PRESSO test | 25 | 0.92 | 0.79-1.06 | 0.261 |  |
|  |  |  | MR-Egger | 25 | / | / | 0.721* |  |
| Christensenellaceae | 2.03% | 31.59 |  |  |  |  |  |  |
|  |  |  | Inverse-variance weighted (fixed) | 12 | 0.89 | 0.75-1.05 | 0.172 | 0.475 |
|  |  |  | Weighted median | 12 | 0.88 | 0.69-1.11 | 0.274 |  |
|  |  |  | MR-PRESSO test | 12 | 0.89 | 0.75-1.05 | 0.192 |  |
|  |  |  | MR-Egger | 12 | / | / | 0.908* |  |
| Clostridiaceae1 | 1.71% | 29.00 |  |  |  |  |  |  |
|  |  |  | Inverse-variance weighted (fixed) | 11 | 0.84 | 0.70-1.01 | 0.059 | 0.214 |
|  |  |  | Weighted median | 11 | 0.85 | 0.67-1.10 | 0.217 |  |
|  |  |  | MR-PRESSO test | 11 | 0.84 | 0.68-1.03 | 0.131 |  |
|  |  |  | MR-Egger | 11 | / | / | 0.946* |  |
| ClostridialesvadinBB60 | 3.73% | 41.81 |  |  |  |  |  |  |
|  |  |  | Inverse-variance weighted (fixed) | 17 | 0.93 | 0.82-1.05 | 0.243 | 0.546 |
|  |  |  | Weighted median | 17 | 0.96 | 0.82-1.14 | 0.671 |  |
|  |  |  | MR-PRESSO test | 17 | 0.93 | 0.83-1.04 | 0.241 |  |
|  |  |  | MR-Egger | 17 | / | / | 0.343* |  |
| Coriobacteriaceae | 2.99% | 26.93 |  |  |  |  |  |  |
|  |  |  | Inverse-variance weighted (fixed) | 21 | 1.03 | 0.89-1.18 | 0.715 | 0.610 |
|  |  |  | Weighted median | 21 | 1.04 | 0.84-1.29 | 0.732 |  |
|  |  |  | MR-PRESSO test | 21 | 1.03 | 0.90-1.17 | 0.701 |  |
|  |  |  | MR-Egger | 21 | / | / | 0.692* |  |
| Defluviitaleaceae | 3.59% | 52.53 |  |  |  |  |  |  |
|  |  |  | Inverse-variance weighted (fixed) | 13 | 1.00 | 0.88-1.14 | 0.998 | 0.296 |
|  |  |  | Weighted median | 13 | 1.01 | 0.84-1.21 | 0.927 |  |
|  |  |  | MR-PRESSO test | 13 | 1.00 | 0.87-1.15 | 0.998 |  |
|  |  |  | MR-Egger | 13 | / | / | 0.833* |  |
| Desulfovibrionaceae | 2.00% | 31.20 |  |  |  |  |  |  |
|  |  |  | Inverse-variance weighted (fixed) | 12 | 1.13 | 0.95-1.33 | 0.157 | 0.840 |
|  |  |  | Weighted median | 12 | 1.16 | 0.92-1.46 | 0.207 |  |
|  |  |  | MR-PRESSO test | 12 | 1.13 | 0.99-1.28 | 0.092 |  |
|  |  |  | MR-Egger | 12 | / | / | 0.811* |  |
| Enterobacteriaceae | 1.84% | 31.17 |  |  |  |  |  |  |
|  |  |  | Inverse-variance weighted (fixed) | 11 | 1.15 | 0.95-1.39 | 0.143 | 0.322 |
|  |  |  | Weighted median | 11 | 1.09 | 0.85-1.40 | 0.506 |  |
|  |  |  | MR-PRESSO test | 11 | 1.15 | 0.94-1.41 | 0.202 |  |
|  |  |  | MR-Egger | 11 | / | / | 0.096* |  |
| Erysipelotrichaceae | 1.65% | 23.71 |  |  |  |  |  |  |
|  |  |  | Inverse-variance weighted (fixed) | 13 | 1.03 | 0.85-1.25 | 0.765 | 0.845 |
|  |  |  | Weighted median | 13 | 1.04 | 0.81-1.33 | 0.781 |  |
|  |  |  | MR-PRESSO test | 13 | 1.03 | 0.89-1.19 | 0.706 |  |
|  |  |  | MR-Egger | 13 | / | / | 0.479* |  |
| FamilyXI (ID: 1936) | 6.97% | 137.38 |  |  |  |  |  |  |
|  |  |  | Inverse-variance weighted (fixed) | 10 | 1.00 | 0.92-1.10 | 0.968 | 0.551 |
|  |  |  | Weighted median | 10 | 1.00 | 0.89-1.13 | 0.966 |  |
|  |  |  | MR-PRESSO test | 10 | 1.00 | 0.92-1.09 | 0.967 |  |
|  |  |  | MR-Egger | 10 | / | / | 0.717* |  |
| FamilyXIII (ID: 1957) | 2.24% | 30.01 |  |  |  |  |  |  |
|  |  |  | Inverse-variance weighted (fixed) | 12 | 0.99 | 0.82-1.20 | 0.925 | 0.682 |
|  |  |  | Weighted median | 12 | 0.95 | 0.74-1.22 | 0.685 |  |
|  |  |  | MR-PRESSO test | 12 | 0.99 | 0.84-1.17 | 0.916 |  |
|  |  |  | MR-Egger | 12 | / | / | 0.876* |  |
| Lachnospiraceae | 2.61% | 27.33 |  |  |  |  |  |  |
|  |  |  | Inverse-variance weighted (fixed) | 18 | 1.15 | 0.99-1.34 | 0.065 | 0.840 |
|  |  |  | Weighted median | 18 | 1.22 | 0.99-1.49 | 0.059 |  |
|  |  |  | MR-PRESSO test | 18 | 1.15 | 1.02-1.30 | 0.037 |  |
|  |  |  | MR-Egger | 18 | / | / | 0.733* |  |
| Lactobacillaceae | 4.43% | 65.42 |  |  |  |  |  |  |
|  |  |  | Inverse-variance weighted (fixed) | 13 | 0.91 | 0.81-1.02 | 0.109 | 0.513 |
|  |  |  | Weighted median | 13 | 0.90 | 0.77-1.05 | 0.185 |  |
|  |  |  | MR-PRESSO test | 13 | 0.91 | 0.81-1.02 | 0.123 |  |
|  |  |  | MR-Egger | 13 | / | / | 0.765* |  |
| Methanobacteriaceae | 7.23% | 119.05 |  |  |  |  |  |  |
|  |  |  | Inverse-variance weighted (fixed) | 12 | 0.96 | 0.88-1.05 | 0.335 | 0.688 |
|  |  |  | Weighted median | 12 | 0.97 | 0.86-1.09 | 0.611 |  |
|  |  |  | MR-PRESSO test | 12 | 0.96 | 0.89-1.03 | 0.290 |  |
|  |  |  | MR-Egger | 12 | / | / | 0.871* |  |
| Oxalobacteraceae | 6.93% | 90.93 |  |  |  |  |  |  |
|  |  |  | Inverse-variance weighted (fixed) | 15 | 1.11 | 1.01-1.22 | 0.027 | 0.181 |
|  |  |  | Weighted median | 15 | 1.18 | 1.03-1.35 | 0.014 |  |
|  |  |  | MR-PRESSO test | 15 | 1.11 | 1.00-1.24 | 0.075 |  |
|  |  |  | MR-Egger | 15 | / | / | 0.648* |  |
| Pasteurellaceae | 5.52% | 56.29 |  |  |  |  |  |  |
|  |  |  | Inverse-variance weighted (fixed) | 19 | 1.00 | 0.90-1.11 | 0.963 | 0.154 |
|  |  |  | Weighted median | 19 | 0.93 | 0.79-1.08 | 0.326 |  |
|  |  |  | MR-PRESSO test | 19 | 1.00 | 0.89-1.13 | 0.968 |  |
|  |  |  | MR-Egger | 19 | / | / | 0.885* |  |
| Peptococcaceae | 2.80% | 52.72 |  |  |  |  |  |  |
|  |  |  | Inverse-variance weighted (fixed) | 10 | 0.90 | 0.78-1.04 | 0.148 | 0.149 |
|  |  |  | Weighted median | 10 | 0.82 | 0.66-1.01 | 0.062 |  |
|  |  |  | MR-PRESSO test | 10 | 0.90 | 0.75-1.07 | 0.265 |  |
|  |  |  | MR-Egger | 10 | / | / | 0.635* |  |
| Peptostreptococcaceae | 3.05% | 36.03 |  |  |  |  |  |  |
|  |  |  | Inverse-variance weighted (fixed) | 16 | 1.02 | 0.88-1.18 | 0.790 | 0.570 |
|  |  |  | Weighted median | 16 | 1.04 | 0.84-1.27 | 0.738 |  |
|  |  |  | MR-PRESSO test | 16 | 1.02 | 0.89-1.17 | 0.782 |  |
|  |  |  | MR-Egger | 16 | / | / | 0.097* |  |
| Porphyromonadaceae | 1.46% | 22.60 |  |  |  |  |  |  |
|  |  |  | Inverse-variance weighted (fixed) | 12 | 1.02 | 0.84-1.24 | 0.823 | 0.100 |
|  |  |  | Weighted median | 12 | 1.12 | 0.85-1.49 | 0.416 |  |
|  |  |  | MR-PRESSO test | 12 | 1.02 | 0.80-1.31 | 0.862 |  |
|  |  |  | MR-Egger | 12 | / | / | 0.044* |  |
| Prevotellaceae | 3.07% | 32.27 |  |  |  |  |  |  |
|  |  |  | Inverse-variance weighted (fixed) | 18 | 0.91 | 0.79-1.04 | 0.163 | 0.198 |
|  |  |  | Weighted median | 18 | 0.88 | 0.72-1.07 | 0.198 |  |
|  |  |  | MR-PRESSO test | 18 | 0.91 | 0.77-1.06 | 0.233 |  |
|  |  |  | MR-Egger | 18 | / | / | 0.323* |  |
| Rhodospirillaceae | 3.91% | 46.76 |  |  |  |  |  |  |
|  |  |  | Inverse-variance weighted (fixed) | 16 | 1.10 | 0.98-1.24 | 0.107 | 0.516 |
|  |  |  | Weighted median | 16 | 1.14 | 0.96-1.36 | 0.135 |  |
|  |  |  | MR-PRESSO test | 16 | 1.10 | 0.98-1.24 | 0.118 |  |
|  |  |  | MR-Egger | 16 | / | / | 0.129* |  |
| Rikenellaceae | 3.07% | 25.20 |  |  |  |  |  |  |
|  |  |  | Inverse-variance weighted (fixed) | 23 | 1.10 | 0.96-1.26 | 0.176 | 0.527 |
|  |  |  | Weighted median | 23 | 1.11 | 0.91-1.36 | 0.297 |  |
|  |  |  | MR-PRESSO test | 23 | 1.10 | 0.96-1.26 | 0.179 |  |
|  |  |  | MR-Egger | 23 | / | / | 0.911* |  |
| Ruminococcaceae | 1.90% | 29.58 |  |  |  |  |  |  |
|  |  |  | Inverse-variance weighted (fixed) | 12 | 0.94 | 0.79-1.12 | 0.482 | 0.554 |
|  |  |  | Weighted median | 12 | 0.96 | 0.76-1.23 | 0.765 |  |
|  |  |  | MR-PRESSO test | 12 | 0.94 | 0.79-1.11 | 0.470 |  |
|  |  |  | MR-Egger | 12 | / | / | 0.429* |  |
| Streptococcaceae | 2.70% | 26.71 |  |  |  |  |  |  |
|  |  |  | Inverse-variance weighted (fixed) | 19 | 1.02 | 0.88-1.18 | 0.781 | 0.340 |
|  |  |  | Weighted median | 19 | 1.06 | 0.86-1.31 | 0.591 |  |
|  |  |  | MR-PRESSO test | 19 | 1.02 | 0.87-1.19 | 0.794 |  |
|  |  |  | MR-Egger | 19 | / | / | 0.418* |  |
| Veillonellaceae | 3.79% | 34.40 |  |  |  |  |  |  |
|  |  |  | Inverse-variance weighted (fixed) | 21 | 0.96 | 0.85-1.09 | 0.512 | 0.730 |
|  |  |  | Weighted median | 21 | 1.01 | 0.84-1.20 | 0.941 |  |
|  |  |  | MR-PRESSO test | 21 | 0.96 | 0.86-1.07 | 0.469 |  |
|  |  |  | MR-Egger | 21 | / | / | 0.839* |  |
| Verrucomicrobiaceae | 2.63% | 38.04 |  |  |  |  |  |  |
|  |  |  | Inverse-variance weighted (fixed) | 13 | 0.95 | 0.82-1.11 | 0.540 | 0.204 |
|  |  |  | Weighted median | 13 | 0.83 | 0.68-1.02 | 0.084 |  |
|  |  |  | MR-PRESSO test | 13 | 0.95 | 0.80-1.13 | 0.602 |  |
|  |  |  | MR-Egger | 13 | / | / | 0.137* |  |
| Victivallaceae | 9.17% | 123.35 |  |  |  |  |  |  |
|  |  |  | Inverse-variance weighted (random) | 15 | 0.99 | 0.89-1.10 | 0.800 | 0.042 |
|  |  |  | Weighted median | 15 | 0.98 | 0.88-1.10 | 0.747 |  |
|  |  |  | MR-PRESSO test | 15 | 0.99 | 0.89-1.10 | 0.804 |  |
|  |  |  | Outlier corrected (MR-PRESSO) | 14 | 1.02 | 0.94-1.11 | 0.627 |  |
|  |  |  | MR-Egger | 15 | / | / | 0.216* |  |
| Genus |  |  |  |  |  |  |  |  |
| Clostridiuminnocuum | 5.90% | 104.48 |  |  |  |  |  |  |
|  |  |  | Inverse-variance weighted (fixed) | 11 | 0.93 | 0.84-1.02 | 0.137 | 0.373 |
|  |  |  | Weighted median | 11 | 0.92 | 0.80-1.05 | 0.225 |  |
|  |  |  | MR-PRESSO test | 11 | 0.93 | 0.83-1.03 | 0.183 |  |
|  |  |  | MR-Egger | 11 | / | / | 0.147* |  |
| Eubacteriumbrachy | 5.35% | 103.67 |  |  |  |  |  |  |
|  |  |  | Inverse-variance weighted (fixed) | 10 | 0.98 | 0.89-1.09 | 0.749 | 0.441 |
|  |  |  | Weighted median | 10 | 0.94 | 0.82-1.09 | 0.433 |  |
|  |  |  | MR-PRESSO test | 10 | 0.98 | 0.89-1.09 | 0.756 |  |
|  |  |  | MR-Egger | 10 | / | / | 0.862* |  |
| Eubacteriumcoprostanoligenes | 1.95% | 24.32 |  |  |  |  |  |  |
|  |  |  | Inverse-variance weighted (fixed) | 15 | 0.95 | 0.80-1.14 | 0.599 | 0.780 |
|  |  |  | Weighted median | 15 | 1.03 | 0.81-1.31 | 0.808 |  |
|  |  |  | MR-PRESSO test | 15 | 0.95 | 0.83-1.10 | 0.539 |  |
|  |  |  | MR-Egger | 15 | / | / | 0.161* |  |
| Eubacteriumeligens | 1.85% | 31.49 |  |  |  |  |  |  |
|  |  |  | Inverse-variance weighted (fixed) | 11 | 1.15 | 0.96-1.38 | 0.134 | 0.060 |
|  |  |  | Weighted median | 11 | 1.10 | 0.86-1.42 | 0.453 |  |
|  |  |  | MR-PRESSO test | 11 | 1.15 | 0.90-1.47 | 0.286 |  |
|  |  |  | MR-Egger | 11 | / | / | 0.073* |  |
| Eubacteriumfissicatena | 5.01% | 107.36 |  |  |  |  |  |  |
|  |  |  | Inverse-variance weighted (fixed) | 9 | 1.04 | 0.93-1.16 | 0.472 | 0.467 |
|  |  |  | Weighted median | 9 | 1.05 | 0.90-1.22 | 0.531 |  |
|  |  |  | MR-PRESSO test | 9 | 1.04 | 0.94-1.16 | 0.484 |  |
|  |  |  | MR-Egger | 9 | / | / | 0.525* |  |
| Eubacteriumhallii | 2.82% | 33.25 |  |  |  |  |  |  |
|  |  |  | Inverse-variance weighted (random) | 16 | 0.83 | 0.65-1.07 | 0.155 | 0.001 |
|  |  |  | Weighted median | 16 | 0.83 | 0.65-1.06 | 0.130 |  |
|  |  |  | MR-PRESSO test | 16 | 0.83 | 0.65-1.07 | 0.175 |  |
|  |  |  | Outlier corrected (MR-PRESSO) | 15 | 0.89 | 0.71-1.11 | 0.321 |  |
|  |  |  | MR-Egger | 16 | / | / | 0.003* |  |
| Eubacteriumnodatum | 7.15% | 128.36 |  |  |  |  |  |  |
|  |  |  | Inverse-variance weighted (fixed) | 11 | 0.96 | 0.88-1.05 | 0.426 | 0.757 |
|  |  |  | Weighted median | 11 | 0.99 | 0.88-1.11 | 0.863 |  |
|  |  |  | MR-PRESSO test | 11 | 0.96 | 0.90-1.04 | 0.352 |  |
|  |  |  | MR-Egger | 11 | / | / | 0.989* |  |
| Eubacteriumoxidoreducens | 2.20% | 82.61 |  |  |  |  |  |  |
|  |  |  | Inverse-variance weighted (fixed) | 5 | 0.89 | 0.75-1.05 | 0.176 | 0.241 |
|  |  |  | Weighted median | 5 | 0.97 | 0.78-1.21 | 0.771 |  |
|  |  |  | MR-PRESSO test | 5 | 0.89 | 0.73-1.08 | 0.312 |  |
|  |  |  | MR-Egger | 5 | / | / | 0.161* |  |
| Eubacteriumrectale | 1.78% | 25.52 |  |  |  |  |  |  |
|  |  |  | Inverse-variance weighted (fixed) | 13 | 1.08 | 0.91-1.29 | 0.379 | 0.688 |
|  |  |  | Weighted median | 13 | 1.13 | 0.89-1.42 | 0.320 |  |
|  |  |  | MR-PRESSO test | 13 | 1.08 | 0.93-1.27 | 0.334 |  |
|  |  |  | MR-Egger | 13 | / | / | 0.086* |  |
| Eubacteriumruminantium | 5.88% | 60.26 |  |  |  |  |  |  |
|  |  |  | Inverse-variance weighted (fixed) | 19 | 1.08 | 0.98-1.20 | 0.132 | 0.969 |
|  |  |  | Weighted median | 19 | 1.11 | 0.97-1.27 | 0.130 |  |
|  |  |  | MR-PRESSO test | 19 | 1.08 | 1.01-1.16 | 0.042 |  |
|  |  |  | MR-Egger | 19 | / | / | 0.740* |  |
| Eubacteriumventriosum | 2.60% | 28.78 |  |  |  |  |  |  |
|  |  |  | Inverse-variance weighted (fixed) | 17 | 0.82 | 0.70-0.95 | 0.011 | 0.167 |
|  |  |  | Weighted median | 17 | 0.75 | 0.60-0.94 | 0.014 |  |
|  |  |  | MR-PRESSO test | 17 | 0.82 | 0.68-0.98 | 0.043 |  |
|  |  |  | MR-Egger | 17 | / | / | 0.869* |  |
| Eubacteriumxylanophilum | 2.11% | 32.91 |  |  |  |  |  |  |
|  |  |  | Inverse-variance weighted (fixed) | 12 | 1.05 | 0.89-1.25 | 0.575 | 0.477 |
|  |  |  | Weighted median | 12 | 1.15 | 0.92-1.43 | 0.213 |  |
|  |  |  | MR-PRESSO test | 12 | 1.05 | 0.89-1.24 | 0.579 |  |
|  |  |  | MR-Egger | 12 | / | / | 0.264* |  |
| Ruminococcusgauvreauii | 2.15% | 31.00 |  |  |  |  |  |  |
|  |  |  | Inverse-variance weighted (random) | 13 | 0.90 | 0.72-1.12 | 0.339 | 0.044 |
|  |  |  | Weighted median | 13 | 0.93 | 0.74-1.17 | 0.523 |  |
|  |  |  | MR-PRESSO test | 13 | 0.90 | 0.72-1.12 | 0.358 |  |
|  |  |  | Outlier corrected (MR-PRESSO) | 12 | 0.97 | 0.82-1.15 | 0.758 |  |
|  |  |  | MR-Egger | 13 | / | / | 0.769* |  |
| Ruminococcusgnavus | 4.08% | 64.94 |  |  |  |  |  |  |
|  |  |  | Inverse-variance weighted (fixed) | 12 | 1.00 | 0.89-1.12 | 0.954 | 0.261 |
|  |  |  | Weighted median | 12 | 0.97 | 0.83-1.13 | 0.679 |  |
|  |  |  | MR-PRESSO test | 12 | 1.00 | 0.87-1.14 | 0.960 |  |
|  |  |  | MR-Egger | 12 | / | / | 0.070* |  |
| Ruminococcustorques | 2.08% | 26.00 |  |  |  |  |  |  |
|  |  |  | Inverse-variance weighted (random) | 15 | 0.92 | 0.73-1.17 | 0.512 | 0.012 |
|  |  |  | Weighted median | 15 | 0.87 | 0.68-1.11 | 0.252 |  |
|  |  |  | MR-PRESSO test | 15 | 0.92 | 0.73-1.17 | 0.523 |  |
|  |  |  | Outlier corrected (MR-PRESSO) | 14 | 0.86 | 0.71-1.05 | 0.162 |  |
|  |  |  | MR-Egger | 15 | / | / | 0.085* |  |
| Actinomyces | 2.66% | 62.61 |  |  |  |  |  |  |
|  |  |  | Inverse-variance weighted (fixed) | 8 | 0.97 | 0.84-1.12 | 0.646 | 0.320 |
|  |  |  | Weighted median | 8 | 1.05 | 0.86-1.28 | 0.645 |  |
|  |  |  | MR-PRESSO test | 8 | 0.97 | 0.83-1.13 | 0.683 |  |
|  |  |  | MR-Egger | 8 | / | / | 0.213* |  |
| Adlercreutzia | 3.11% | 49.06 |  |  |  |  |  |  |
|  |  |  | Inverse-variance weighted (fixed) | 12 | 1.06 | 0.93-1.22 | 0.380 | 0.857 |
|  |  |  | Weighted median | 12 | 0.98 | 0.82-1.17 | 0.843 |  |
|  |  |  | MR-PRESSO test | 12 | 1.06 | 0.96-1.18 | 0.268 |  |
|  |  |  | MR-Egger | 12 | / | / | 0.976* |  |
| Akkermansia | 2.63% | 38.06 |  |  |  |  |  |  |
|  |  |  | Inverse-variance weighted (fixed) | 13 | 0.95 | 0.82-1.11 | 0.539 | 0.204 |
|  |  |  | Weighted median | 13 | 0.83 | 0.68-1.02 | 0.083 |  |
|  |  |  | MR-PRESSO test | 13 | 0.95 | 0.80-1.13 | 0.601 |  |
|  |  |  | MR-Egger | 13 | / | / | 0.136* |  |
| Alistipes | 1.80% | 22.37 |  |  |  |  |  |  |
|  |  |  | Inverse-variance weighted (fixed) | 15 | 1.05 | 0.88-1.25 | 0.602 | 0.187 |
|  |  |  | Weighted median | 15 | 1.12 | 0.87-1.45 | 0.390 |  |
|  |  |  | MR-PRESSO test | 15 | 1.05 | 0.86-1.29 | 0.656 |  |
|  |  |  | MR-Egger | 15 | / | / | 0.058* |  |
| Allisonella | 6.06% | 131.44 |  |  |  |  |  |  |
|  |  |  | Inverse-variance weighted (fixed) | 9 | 1.05 | 0.95-1.15 | 0.369 | 0.433 |
|  |  |  | Weighted median | 9 | 1.04 | 0.92-1.18 | 0.537 |  |
|  |  |  | MR-PRESSO test | 9 | 1.05 | 0.95-1.15 | 0.396 |  |
|  |  |  | MR-Egger | 9 | / | / | 0.067* |  |
| Alloprevotella | 4.85% | 133.35 |  |  |  |  |  |  |
|  |  |  | Inverse-variance weighted (fixed) | 7 | 1.01 | 0.91-1.13 | 0.834 | 0.349 |
|  |  |  | Weighted median | 7 | 1.07 | 0.92-1.24 | 0.367 |  |
|  |  |  | MR-PRESSO test | 7 | 1.01 | 0.90-1.13 | 0.850 |  |
|  |  |  | MR-Egger | 7 | / | / | 0.587* |  |
| Anaerofilum | 5.45% | 88.11 |  |  |  |  |  |  |
|  |  |  | Inverse-variance weighted (fixed) | 12 | 0.97 | 0.87-1.07 | 0.506 | 0.233 |
|  |  |  | Weighted median | 12 | 0.96 | 0.83-1.12 | 0.617 |  |
|  |  |  | MR-PRESSO test | 12 | 0.97 | 0.86-1.09 | 0.567 |  |
|  |  |  | MR-Egger | 12 | / | / | 0.906* |  |
| Anaerostipes | 2.19% | 27.40 |  |  |  |  |  |  |
|  |  |  | Inverse-variance weighted (fixed) | 15 | 1.04 | 0.88-1.23 | 0.629 | 0.795 |
|  |  |  | Weighted median | 15 | 1.02 | 0.81-1.28 | 0.885 |  |
|  |  |  | MR-PRESSO test | 15 | 1.04 | 0.91-1.20 | 0.567 |  |
|  |  |  | MR-Egger | 15 | / | / | 0.305* |  |
| Anaerotruncus | 2.14% | 25.10 |  |  |  |  |  |  |
|  |  |  | Inverse-variance weighted (fixed) | 15 | 0.86 | 0.73-1.02 | 0.077 | 0.221 |
|  |  |  | Weighted median | 15 | 0.83 | 0.64-1.07 | 0.160 |  |
|  |  |  | MR-PRESSO test | 15 | 0.86 | 0.71-1.04 | 0.139 |  |
|  |  |  | MR-Egger | 15 | / | / | 0.940* |  |
| Bacteroides | 1.47% | 27.38 |  |  |  |  |  |  |
|  |  |  | Inverse-variance weighted (fixed) | 10 | 1.10 | 0.88-1.37 | 0.394 | 0.749 |
|  |  |  | Weighted median | 10 | 1.16 | 0.87-1.54 | 0.309 |  |
|  |  |  | MR-PRESSO test | 10 | 1.10 | 0.92-1.31 | 0.321 |  |
|  |  |  | MR-Egger | 10 | / | / | 0.645* |  |
| Barnesiella | 2.63% | 31.01 |  |  |  |  |  |  |
|  |  |  | Inverse-variance weighted (fixed) | 16 | 1.14 | 0.98-1.33 | 0.098 | 0.477 |
|  |  |  | Weighted median | 16 | 1.12 | 0.90-1.39 | 0.319 |  |
|  |  |  | MR-PRESSO test | 16 | 1.14 | 0.98-1.33 | 0.115 |  |
|  |  |  | MR-Egger | 16 | / | / | 0.310* |  |
| Bifidobacterium | 4.32% | 37.58 |  |  |  |  |  |  |
|  |  |  | Inverse-variance weighted (random) | 22 | 0.94 | 0.80-1.09 | 0.399 | 0.044 |
|  |  |  | Weighted median | 22 | 0.91 | 0.76-1.09 | 0.300 |  |
|  |  |  | MR-PRESSO test | 22 | 0.94 | 0.80-1.09 | 0.408 |  |
|  |  |  | MR-Egger | 22 | / | / | 0.290* |  |
| Bilophila | 2.82% | 31.30 |  |  |  |  |  |  |
|  |  |  | Inverse-variance weighted (fixed) | 17 | 1.11 | 0.96-1.28 | 0.152 | 0.581 |
|  |  |  | Weighted median | 17 | 1.08 | 0.89-1.31 | 0.449 |  |
|  |  |  | MR-PRESSO test | 17 | 1.11 | 0.97-1.27 | 0.149 |  |
|  |  |  | MR-Egger | 17 | / | / | 0.572* |  |
| Blautia | 2.01% | 28.89 |  |  |  |  |  |  |
|  |  |  | Inverse-variance weighted (fixed) | 13 | 1.11 | 0.93-1.32 | 0.243 | 0.831 |
|  |  |  | Weighted median | 13 | 1.13 | 0.90-1.42 | 0.283 |  |
|  |  |  | MR-PRESSO test | 13 | 1.11 | 0.97-1.27 | 0.162 |  |
|  |  |  | MR-Egger | 13 | / | / | 0.546* |  |
| Butyricicoccus | 1.95% | 40.56 |  |  |  |  |  |  |
|  |  |  | Inverse-variance weighted (random) | 9 | 1.10 | 0.80-1.51 | 0.570 | 0.001 |
|  |  |  | Weighted median | 9 | 0.94 | 0.73-1.21 | 0.620 |  |
|  |  |  | MR-PRESSO test | 9 | 1.10 | 0.80-1.51 | 0.586 |  |
|  |  |  | Outlier corrected (MR-PRESSO) | 8 | 1.00 | 0.78-1.29 | 0.988 |  |
|  |  |  | MR-Egger | 9 | / | / | 0.484* |  |
| Butyricimonas | 3.92% | 41.48 |  |  |  |  |  |  |
|  |  |  | Inverse-variance weighted (fixed) | 18 | 1.01 | 0.89-1.14 | 0.913 | 0.281 |
|  |  |  | Weighted median | 18 | 1.11 | 0.94-1.33 | 0.223 |  |
|  |  |  | MR-PRESSO test | 18 | 1.01 | 0.88-1.15 | 0.921 |  |
|  |  |  | MR-Egger | 18 | / | / | 0.803* |  |
| Butyrivibrio | 10.11% | 128.73 |  |  |  |  |  |  |
|  |  |  | Inverse-variance weighted (fixed) | 16 | 0.96 | 0.89-1.04 | 0.306 | 0.206 |
|  |  |  | Weighted median | 16 | 0.94 | 0.84-1.05 | 0.248 |  |
|  |  |  | MR-PRESSO test | 16 | 0.96 | 0.88-1.05 | 0.380 |  |
|  |  |  | MR-Egger | 16 | / | / | 0.072* |  |
| CandidatusSoleaferrea | 5.83% | 70.84 |  |  |  |  |  |  |
|  |  |  | Inverse-variance weighted (fixed) | 16 | 0.98 | 0.89-1.09 | 0.732 | 0.535 |
|  |  |  | Weighted median | 16 | 0.94 | 0.82-1.09 | 0.407 |  |
|  |  |  | MR-PRESSO test | 16 | 0.98 | 0.89-1.08 | 0.726 |  |
|  |  |  | MR-Egger | 16 | / | / | 0.438* |  |
| Catenibacterium | 3.28% | 124.43 |  |  |  |  |  |  |
|  |  |  | Inverse-variance weighted (fixed) | 5 | 0.89 | 0.78-1.01 | 0.077 | 0.209 |
|  |  |  | Weighted median | 5 | 0.82 | 0.69-0.99 | 0.034 |  |
|  |  |  | MR-PRESSO test | 5 | 0.89 | 0.76-1.04 | 0.218 |  |
|  |  |  | MR-Egger | 5 | / | / | 0.103* |  |
| ChristensenellaceaeR.7 | 1.50% | 25.33 |  |  |  |  |  |  |
|  |  |  | Inverse-variance weighted (fixed) | 11 | 0.93 | 0.76-1.13 | 0.451 | 0.140 |
|  |  |  | Weighted median | 11 | 1.11 | 0.83-1.47 | 0.489 |  |
|  |  |  | MR-PRESSO test | 11 | 0.93 | 0.72-1.18 | 0.549 |  |
|  |  |  | MR-Egger | 11 | / | / | 0.445* |  |
| Clostridiumsensustricto1 | 1.86% | 38.54 |  |  |  |  |  |  |
|  |  |  | Inverse-variance weighted (fixed) | 9 | 0.79 | 0.66-0.94 | 0.010 | 0.256 |
|  |  |  | Weighted median | 9 | 0.82 | 0.64-1.04 | 0.096 |  |
|  |  |  | MR-PRESSO test | 9 | 0.79 | 0.65-0.97 | 0.050 |  |
|  |  |  | MR-Egger | 9 | / | / | 0.259* |  |
| Collinsella | 1.93% | 27.67 |  |  |  |  |  |  |
|  |  |  | Inverse-variance weighted (fixed) | 13 | 0.95 | 0.80-1.13 | 0.547 | 0.537 |
|  |  |  | Weighted median | 13 | 1.03 | 0.82-1.30 | 0.795 |  |
|  |  |  | MR-PRESSO test | 13 | 0.95 | 0.81-1.12 | 0.539 |  |
|  |  |  | MR-Egger | 13 | / | / | 0.783* |  |
| Coprobacter | 5.29% | 73.18 |  |  |  |  |  |  |
|  |  |  | Inverse-variance weighted (fixed) | 14 | 0.96 | 0.86-1.07 | 0.438 | 0.056 |
|  |  |  | Weighted median | 14 | 0.95 | 0.80-1.13 | 0.560 |  |
|  |  |  | MR-PRESSO test | 14 | 0.96 | 0.83-1.11 | 0.561 |  |
|  |  |  | MR-Egger | 14 | / | / | 0.061* |  |
| Coprococcus1 | 2.38% | 31.91 |  |  |  |  |  |  |
|  |  |  | Inverse-variance weighted (fixed) | 14 | 1.05 | 0.89-1.23 | 0.585 | 0.452 |
|  |  |  | Weighted median | 14 | 1.02 | 0.81-1.28 | 0.897 |  |
|  |  |  | MR-PRESSO test | 14 | 1.05 | 0.89-1.23 | 0.594 |  |
|  |  |  | MR-Egger | 14 | / | / | 0.667* |  |
| Coprococcus2 | 2.36% | 36.88 |  |  |  |  |  |  |
|  |  |  | Inverse-variance weighted (fixed) | 12 | 1.21 | 1.03-1.41 | 0.022 | 0.805 |
|  |  |  | Weighted median | 12 | 1.29 | 1.04-1.60 | 0.021 |  |
|  |  |  | MR-PRESSO test | 12 | 1.21 | 1.06-1.37 | 0.015 |  |
|  |  |  | MR-Egger | 12 | / | / | 0.477* |  |
| Coprococcus3 | 1.62% | 27.50 |  |  |  |  |  |  |
|  |  |  | Inverse-variance weighted (fixed) | 11 | 1.04 | 0.85-1.26 | 0.717 | 0.053 |
|  |  |  | Weighted median | 11 | 0.99 | 0.74-1.32 | 0.948 |  |
|  |  |  | MR-PRESSO test | 11 | 1.04 | 0.80-1.35 | 0.793 |  |
|  |  |  | MR-Egger | 11 | / | / | 0.173* |  |
| DefluviitaleaceaeUCG011 | 3.18% | 54.72 |  |  |  |  |  |  |
|  |  |  | Inverse-variance weighted (fixed) | 11 | 1.10 | 0.95-1.26 | 0.197 | 0.821 |
|  |  |  | Weighted median | 11 | 1.08 | 0.91-1.29 | 0.373 |  |
|  |  |  | MR-PRESSO test | 11 | 1.10 | 0.98-1.22 | 0.125 |  |
|  |  |  | MR-Egger | 11 | / | / | 0.550* |  |
| Desulfovibrio | 3.18% | 50.13 |  |  |  |  |  |  |
|  |  |  | Inverse-variance weighted (fixed) | 12 | 1.05 | 0.92-1.21 | 0.476 | 0.712 |
|  |  |  | Weighted median | 12 | 1.09 | 0.90-1.32 | 0.374 |  |
|  |  |  | MR-PRESSO test | 12 | 1.05 | 0.93-1.18 | 0.421 |  |
|  |  |  | MR-Egger | 12 | / | / | 0.978* |  |
| Dialister | 2.16% | 33.65 |  |  |  |  |  |  |
|  |  |  | Inverse-variance weighted (fixed) | 12 | 0.99 | 0.84-1.16 | 0.857 | 0.383 |
|  |  |  | Weighted median | 12 | 1.08 | 0.86-1.35 | 0.513 |  |
|  |  |  | MR-PRESSO test | 12 | 0.99 | 0.83-1.17 | 0.865 |  |
|  |  |  | MR-Egger | 12 | / | / | 0.432* |  |
| Dorea | 1.82% | 26.17 |  |  |  |  |  |  |
|  |  |  | Inverse-variance weighted (fixed) | 13 | 1.04 | 0.87-1.24 | 0.675 | 0.845 |
|  |  |  | Weighted median | 13 | 1.12 | 0.89-1.40 | 0.340 |  |
|  |  |  | MR-PRESSO test | 13 | 1.04 | 0.90-1.19 | 0.598 |  |
|  |  |  | MR-Egger | 13 | / | / | 0.863* |  |
| Eggerthella | 4.13% | 79.01 |  |  |  |  |  |  |
|  |  |  | Inverse-variance weighted (fixed) | 10 | 0.97 | 0.86-1.10 | 0.680 | 0.634 |
|  |  |  | Weighted median | 10 | 0.93 | 0.79-1.09 | 0.384 |  |
|  |  |  | MR-PRESSO test | 10 | 0.97 | 0.88-1.08 | 0.652 |  |
|  |  |  | MR-Egger | 10 | / | / | 0.705* |  |
| Eisenbergiella | 4.52% | 72.37 |  |  |  |  |  |  |
|  |  |  | Inverse-variance weighted (fixed) | 12 | 1.02 | 0.92-1.14 | 0.688 | 0.094 |
|  |  |  | Weighted median | 12 | 1.06 | 0.91-1.24 | 0.445 |  |
|  |  |  | MR-PRESSO test | 12 | 1.02 | 0.89-1.18 | 0.757 |  |
|  |  |  | MR-Egger | 12 | / | / | 0.376* |  |
| Enterorhabdus | 3.22% | 67.98 |  |  |  |  |  |  |
|  |  |  | Inverse-variance weighted (fixed) | 9 | 0.87 | 0.76-1.00 | 0.044 | 0.554 |
|  |  |  | Weighted median | 9 | 0.84 | 0.70-1.00 | 0.045 |  |
|  |  |  | MR-PRESSO test | 9 | 0.87 | 0.77-0.99 | 0.061 |  |
|  |  |  | MR-Egger | 9 | / | / | 0.132* |  |
| Erysipelatoclostridium | 4.07% | 45.68 |  |  |  |  |  |  |
|  |  |  | Inverse-variance weighted (fixed) | 17 | 0.98 | 0.86-1.10 | 0.691 | 0.139 |
|  |  |  | Weighted median | 17 | 1.02 | 0.85-1.21 | 0.856 |  |
|  |  |  | MR-PRESSO test | 17 | 0.98 | 0.84-1.13 | 0.739 |  |
|  |  |  | MR-Egger | 17 | / | / | 0.105* |  |
| ErysipelotrichaceaeUCG003 | 3.28% | 34.53 |  |  |  |  |  |  |
|  |  |  | Inverse-variance weighted (fixed) | 18 | 1.05 | 0.91-1.20 | 0.493 | 0.603 |
|  |  |  | Weighted median | 18 | 1.04 | 0.86-1.25 | 0.708 |  |
|  |  |  | MR-PRESSO test | 18 | 1.05 | 0.92-1.19 | 0.474 |  |
|  |  |  | MR-Egger | 18 | / | / | 0.988* |  |
| Escherichia.Shigella | 2.97% | 37.39 |  |  |  |  |  |  |
|  |  |  | Inverse-variance weighted (fixed) | 15 | 1.06 | 0.91-1.23 | 0.470 | 0.791 |
|  |  |  | Weighted median | 15 | 1.04 | 0.85-1.27 | 0.705 |  |
|  |  |  | MR-PRESSO test | 15 | 1.06 | 0.93-1.20 | 0.397 |  |
|  |  |  | MR-Egger | 15 | / | / | 0.743* |  |
| Faecalibacterium | 2.60% | 37.59 |  |  |  |  |  |  |
|  |  |  | Inverse-variance weighted (fixed) | 13 | 1.05 | 0.90-1.24 | 0.517 | 0.644 |
|  |  |  | Weighted median | 13 | 0.96 | 0.77-1.21 | 0.745 |  |
|  |  |  | MR-PRESSO test | 13 | 1.05 | 0.91-1.22 | 0.485 |  |
|  |  |  | MR-Egger | 13 | / | / | 0.523* |  |
| FamilyXIIIAD3011 (ID: 11293) | 2.24% | 30.01 |  |  |  |  |  |  |
|  |  |  | Inverse-variance weighted (fixed) | 14 | 1.00 | 0.85-1.18 | 0.972 | 0.428 |
|  |  |  | Weighted median | 14 | 0.96 | 0.76-1.20 | 0.699 |  |
|  |  |  | MR-PRESSO test | 14 | 1.00 | 0.85-1.18 | 0.973 |  |
|  |  |  | MR-Egger | 14 | / | / | 0.958* |  |
| FamilyXIIIUCG001 (ID: 11294) | 1.94% | 36.25 |  |  |  |  |  |  |
|  |  |  | Inverse-variance weighted (fixed) | 10 | 1.13 | 0.95-1.35 | 0.162 | 0.085 |
|  |  |  | Weighted median | 10 | 1.20 | 0.92-1.55 | 0.172 |  |
|  |  |  | MR-PRESSO test | 10 | 1.13 | 0.90-1.43 | 0.311 |  |
|  |  |  | MR-Egger | 10 | / | / | 0.912* |  |
| Flavonifractor | 2.19% | 40.97 |  |  |  |  |  |  |
|  |  |  | Inverse-variance weighted (fixed) | 10 | 0.92 | 0.77-1.08 | 0.311 | 0.050 |
|  |  |  | Weighted median | 10 | 0.96 | 0.75-1.23 | 0.761 |  |
|  |  |  | MR-PRESSO test | 10 | 0.92 | 0.73-1.15 | 0.479 |  |
|  |  |  | MR-Egger | 10 | / | / | 0.641* |  |
| Fusicatenibacter | 2.60% | 24.45 |  |  |  |  |  |  |
|  |  |  | Inverse-variance weighted (fixed) | 20 | 0.96 | 0.82-1.11 | 0.571 | 0.417 |
|  |  |  | Weighted median | 20 | 0.93 | 0.75-1.15 | 0.506 |  |
|  |  |  | MR-PRESSO test | 20 | 0.96 | 0.82-1.12 | 0.583 |  |
|  |  |  | MR-Egger | 20 | / | / | 0.839* |  |
| Gordonibacter | 9.26% | 124.67 |  |  |  |  |  |  |
|  |  |  | Inverse-variance weighted (fixed) | 15 | 1.02 | 0.94-1.10 | 0.694 | 0.176 |
|  |  |  | Weighted median | 15 | 0.98 | 0.87-1.10 | 0.703 |  |
|  |  |  | MR-PRESSO test | 15 | 1.02 | 0.93-1.11 | 0.739 |  |
|  |  |  | MR-Egger | 15 | / | / | 0.548* |  |
| Haemophilus | 4.10% | 55.89 |  |  |  |  |  |  |
|  |  |  | Inverse-variance weighted (fixed) | 14 | 1.11 | 0.99-1.25 | 0.079 | 0.151 |
|  |  |  | Weighted median | 14 | 1.07 | 0.90-1.28 | 0.418 |  |
|  |  |  | MR-PRESSO test | 14 | 1.11 | 0.97-1.28 | 0.161 |  |
|  |  |  | MR-Egger | 14 | / | / | 0.363* |  |
| Holdemanella | 4.79% | 65.87 |  |  |  |  |  |  |
|  |  |  | Inverse-variance weighted (fixed) | 14 | 0.87 | 0.77-0.97 | 0.014 | 0.274 |
|  |  |  | Weighted median | 14 | 0.87 | 0.74-1.03 | 0.102 |  |
|  |  |  | MR-PRESSO test | 14 | 0.87 | 0.77-0.98 | 0.042 |  |
|  |  |  | MR-Egger | 14 | / | / | 0.420* |  |
| Holdemania | 4.84% | 51.77 |  |  |  |  |  |  |
|  |  |  | Inverse-variance weighted (fixed) | 18 | 0.96 | 0.86-1.07 | 0.444 | 0.359 |
|  |  |  | Weighted median | 18 | 0.92 | 0.79-1.08 | 0.322 |  |
|  |  |  | MR-PRESSO test | 18 | 0.96 | 0.86-1.07 | 0.473 |  |
|  |  |  | MR-Egger | 18 | / | / | 0.078* |  |
| Howardella | 6.28% | 111.64 |  |  |  |  |  |  |
|  |  |  | Inverse-variance weighted (fixed) | 11 | 0.96 | 0.87-1.06 | 0.425 | 0.988 |
|  |  |  | Weighted median | 11 | 0.96 | 0.85-1.08 | 0.482 |  |
|  |  |  | MR-PRESSO test | 11 | 0.96 | 0.92-1.01 | 0.156 |  |
|  |  |  | MR-Egger | 11 | / | / | 0.672* |  |
| Hungatella | 2.50% | 93.98 |  |  |  |  |  |  |
|  |  |  | Inverse-variance weighted (fixed) | 5 | 0.94 | 0.80-1.09 | 0.403 | 0.665 |
|  |  |  | Weighted median | 5 | 0.90 | 0.74-1.10 | 0.289 |  |
|  |  |  | MR-PRESSO test | 5 | 0.94 | 0.83-1.05 | 0.340 |  |
|  |  |  | MR-Egger | 5 | / | / | 0.597* |  |
| Intestinibacter | 2.71% | 33.98 |  |  |  |  |  |  |
|  |  |  | Inverse-variance weighted (fixed) | 15 | 0.98 | 0.85-1.13 | 0.753 | 0.725 |
|  |  |  | Weighted median | 15 | 1.02 | 0.85-1.23 | 0.848 |  |
|  |  |  | MR-PRESSO test | 15 | 0.98 | 0.87-1.10 | 0.722 |  |
|  |  |  | MR-Egger | 15 | / | / | 0.085* |  |
| Intestinimonas | 4.41% | 42.28 |  |  |  |  |  |  |
|  |  |  | Inverse-variance weighted (fixed) | 20 | 0.97 | 0.86-1.09 | 0.623 | 0.862 |
|  |  |  | Weighted median | 20 | 0.96 | 0.81-1.13 | 0.596 |  |
|  |  |  | MR-PRESSO test | 20 | 0.97 | 0.88-1.07 | 0.552 |  |
|  |  |  | MR-Egger | 20 | / | / | 0.870* |  |
| Lachnoclostridium | 1.86% | 23.18 |  |  |  |  |  |  |
|  |  |  | Inverse-variance weighted (fixed) | 15 | 0.92 | 0.77-1.10 | 0.371 | 0.082 |
|  |  |  | Weighted median | 15 | 0.97 | 0.75-1.25 | 0.807 |  |
|  |  |  | MR-PRESSO test | 15 | 0.92 | 0.74-1.15 | 0.485 |  |
|  |  |  | MR-Egger | 15 | / | / | 0.014* |  |
| Lachnospira | 0.95% | 25.08 |  |  |  |  |  |  |
|  |  |  | Inverse-variance weighted (random) | 7 | 0.85 | 0.59-1.22 | 0.374 | 0.044 |
|  |  |  | Weighted median | 7 | 0.92 | 0.66-1.30 | 0.638 |  |
|  |  |  | MR-PRESSO test | 7 | 0.85 | 0.59-1.22 | 0.408 |  |
|  |  |  | Outlier corrected (MR-PRESSO) | 6 | 0.99 | 0.78-1.25 | 0.924 |  |
|  |  |  | MR-Egger | 7 | / | / | 0.135* |  |
| LachnospiraceaeFCS020 | 3.11% | 34.54 |  |  |  |  |  |  |
|  |  |  | Inverse-variance weighted (fixed) | 17 | 1.17 | 1.02-1.34 | 0.026 | 0.231 |
|  |  |  | Weighted median | 17 | 1.13 | 0.93-1.38 | 0.205 |  |
|  |  |  | MR-PRESSO test | 17 | 1.17 | 1.00-1.36 | 0.062 |  |
|  |  |  | MR-Egger | 17 | / | / | 0.278* |  |
| LachnospiraceaeNC2004 | 3.49% | 66.37 |  |  |  |  |  |  |
|  |  |  | Inverse-variance weighted (fixed) | 10 | 1.04 | 0.92-1.19 | 0.532 | 0.384 |
|  |  |  | Weighted median | 10 | 1.08 | 0.90-1.30 | 0.379 |  |
|  |  |  | MR-PRESSO test | 10 | 1.04 | 0.91-1.19 | 0.560 |  |
|  |  |  | MR-Egger | 10 | / | / | 0.450* |  |
| LachnospiraceaeND3007 | 0.57% | 26.17 |  |  |  |  |  |  |
|  |  |  | Inverse-variance weighted (fixed) | 4 | 1.62 | 1.13-2.32 | 0.008 | 0.467 |
|  |  |  | Weighted median | 4 | 1.43 | 0.91-2.24 | 0.118 |  |
|  |  |  | MR-PRESSO test | 4 | 1.62 | 1.16-2.25 | 0.065 |  |
|  |  |  | MR-Egger | 4 | / | / | 0.347* |  |
| LachnospiraceaeNK4A136 | 2.75% | 32.32 |  |  |  |  |  |  |
|  |  |  | Inverse-variance weighted (fixed) | 16 | 0.96 | 0.83-1.12 | 0.631 | 0.963 |
|  |  |  | Weighted median | 16 | 1.01 | 0.82-1.24 | 0.909 |  |
|  |  |  | MR-PRESSO test | 16 | 0.96 | 0.87-1.06 | 0.486 |  |
|  |  |  | MR-Egger | 16 | / | / | 0.270* |  |
| LachnospiraceaeUCG001 | 3.38% | 42.75 |  |  |  |  |  |  |
|  |  |  | Inverse-variance weighted (fixed) | 15 | 0.93 | 0.82-1.07 | 0.317 | 0.350 |
|  |  |  | Weighted median | 15 | 0.97 | 0.80-1.16 | 0.723 |  |
|  |  |  | MR-PRESSO test | 15 | 0.93 | 0.81-1.07 | 0.357 |  |
|  |  |  | MR-Egger | 15 | / | / | 0.870* |  |
| LachnospiraceaeUCG004 | 2.28% | 28.45 |  |  |  |  |  |  |
|  |  |  | Inverse-variance weighted (fixed) | 15 | 0.92 | 0.79-1.08 | 0.328 | 0.874 |
|  |  |  | Weighted median | 15 | 0.92 | 0.75-1.13 | 0.440 |  |
|  |  |  | MR-PRESSO test | 15 | 0.92 | 0.82-1.04 | 0.225 |  |
|  |  |  | MR-Egger | 15 | / | / | 0.695* |  |
| LachnospiraceaeUCG008 | 4.84% | 66.62 |  |  |  |  |  |  |
|  |  |  | Inverse-variance weighted (fixed) | 14 | 1.09 | 0.97-1.22 | 0.142 | 0.668 |
|  |  |  | Weighted median | 14 | 1.05 | 0.90-1.23 | 0.508 |  |
|  |  |  | MR-PRESSO test | 14 | 1.09 | 0.98-1.20 | 0.123 |  |
|  |  |  | MR-Egger | 14 | / | / | 0.923* |  |
| LachnospiraceaeUCG010 | 2.52% | 36.40 |  |  |  |  |  |  |
|  |  |  | Inverse-variance weighted (fixed) | 13 | 1.22 | 1.05-1.43 | 0.012 | 0.062 |
|  |  |  | Weighted median | 13 | 1.36 | 1.08-1.70 | 0.009 |  |
|  |  |  | MR-PRESSO test | 13 | 1.22 | 1.00-1.49 | 0.077 |  |
|  |  |  | MR-Egger | 13 | / | / | 0.613* |  |
| Lactobacillus | 4.16% | 66.24 |  |  |  |  |  |  |
|  |  |  | Inverse-variance weighted (fixed) | 12 | 0.89 | 0.79-1.01 | 0.061 | 0.266 |
|  |  |  | Weighted median | 12 | 0.89 | 0.76-1.06 | 0.187 |  |
|  |  |  | MR-PRESSO test | 12 | 0.89 | 0.78-1.02 | 0.118 |  |
|  |  |  | MR-Egger | 12 | / | / | 0.929* |  |
| Lactococcus | 5.95% | 105.41 |  |  |  |  |  |  |
|  |  |  | Inverse-variance weighted (fixed) | 11 | 1.00 | 0.91-1.10 | 0.993 | 0.217 |
|  |  |  | Weighted median | 11 | 1.00 | 0.87-1.15 | 0.999 |  |
|  |  |  | MR-PRESSO test | 11 | 1.00 | 0.90-1.12 | 0.994 |  |
|  |  |  | MR-Egger | 11 | / | / | 0.880* |  |
| Marvinbryantia | 2.43% | 35.13 |  |  |  |  |  |  |
|  |  |  | Inverse-variance weighted (fixed) | 13 | 1.08 | 0.92-1.26 | 0.333 | 0.579 |
|  |  |  | Weighted median | 13 | 1.04 | 0.83-1.28 | 0.754 |  |
|  |  |  | MR-PRESSO test | 13 | 1.08 | 0.93-1.25 | 0.319 |  |
|  |  |  | MR-Egger | 13 | / | / | 0.475* |  |
| Methanobrevibacter | 4.61% | 110.62 |  |  |  |  |  |  |
|  |  |  | Inverse-variance weighted (random) | 8 | 1.03 | 0.88-1.20 | 0.752 | 0.044 |
|  |  |  | Weighted median | 8 | 1.00 | 0.86-1.17 | 0.965 |  |
|  |  |  | MR-PRESSO test | 8 | 1.03 | 0.88-1.20 | 0.761 |  |
|  |  |  | MR-Egger | 8 | / | / | 0.397* |  |
| Odoribacter | 1.45% | 30.01 |  |  |  |  |  |  |
|  |  |  | Inverse-variance weighted (fixed) | 9 | 1.15 | 0.94-1.41 | 0.177 | 0.790 |
|  |  |  | Weighted median | 9 | 1.10 | 0.85-1.43 | 0.476 |  |
|  |  |  | MR-PRESSO test | 9 | 1.15 | 0.98-1.35 | 0.116 |  |
|  |  |  | MR-Egger | 9 | / | / | 0.693* |  |
| Olsenella | 6.41% | 114.06 |  |  |  |  |  |  |
|  |  |  | Inverse-variance weighted (fixed) | 11 | 0.92 | 0.83-1.01 | 0.070 | 0.546 |
|  |  |  | Weighted median | 11 | 0.91 | 0.8-1.04 | 0.165 |  |
|  |  |  | MR-PRESSO test | 11 | 0.92 | 0.84-1.00 | 0.083 |  |
|  |  |  | MR-Egger | 11 | / | / | 0.923* |  |
| Oscillibacter | 4.29% | 51.30 |  |  |  |  |  |  |
|  |  |  | Inverse-variance weighted (fixed) | 16 | 0.88 | 0.78-0.99 | 0.034 | 0.532 |
|  |  |  | Weighted median | 16 | 0.88 | 0.75-1.04 | 0.144 |  |
|  |  |  | MR-PRESSO test | 16 | 0.88 | 0.79-0.99 | 0.044 |  |
|  |  |  | MR-Egger | 16 | / | / | 0.891* |  |
| Oscillospira | 2.17% | 40.72 |  |  |  |  |  |  |
|  |  |  | Inverse-variance weighted (fixed) | 10 | 0.88 | 0.75-1.04 | 0.127 | 0.270 |
|  |  |  | Weighted median | 10 | 0.92 | 0.74-1.16 | 0.480 |  |
|  |  |  | MR-PRESSO test | 10 | 0.88 | 0.73-1.06 | 0.202 |  |
|  |  |  | MR-Egger | 10 | / | / | 0.794* |  |
| Oxalobacter | 6.51% | 106.27 |  |  |  |  |  |  |
|  |  |  | Inverse-variance weighted (fixed) | 12 | 1.17 | 1.07-1.29 | 0.001 | 0.980 |
|  |  |  | Weighted median | 12 | 1.17 | 1.04-1.33 | 0.011 |  |
|  |  |  | MR-PRESSO test | 12 | 1.17 | 1.11-1.24 | 0.000 |  |
|  |  |  | MR-Egger | 12 | / | / | 0.620* |  |
| Parabacteroides | 1.47% | 27.38 |  |  |  |  |  |  |
|  |  |  | Inverse-variance weighted (fixed) | 10 | 0.99 | 0.81-1.20 | 0.911 | 0.299 |
|  |  |  | Weighted median | 10 | 1.13 | 0.86-1.47 | 0.380 |  |
|  |  |  | MR-PRESSO test | 10 | 0.99 | 0.80-1.23 | 0.921 |  |
|  |  |  | MR-Egger | 10 | / | / | 0.100* |  |
| Paraprevotella | 4.40% | 64.84 |  |  |  |  |  |  |
|  |  |  | Inverse-variance weighted (fixed) | 13 | 0.98 | 0.87-1.10 | 0.705 | 0.399 |
|  |  |  | Weighted median | 13 | 1.00 | 0.86-1.17 | 0.980 |  |
|  |  |  | MR-PRESSO test | 13 | 0.98 | 0.87-1.10 | 0.718 |  |
|  |  |  | MR-Egger | 13 | / | / | 0.646* |  |
| Parasutterella | 3.69% | 41.32 |  |  |  |  |  |  |
|  |  |  | Inverse-variance weighted (fixed) | 17 | 1.07 | 0.94-1.21 | 0.293 | 0.464 |
|  |  |  | Weighted median | 17 | 1.04 | 0.87-1.24 | 0.692 |  |
|  |  |  | MR-PRESSO test | 17 | 1.07 | 0.94-1.21 | 0.306 |  |
|  |  |  | MR-Egger | 17 | / | / | 0.354* |  |
| Peptococcus | 6.62% | 81.14 |  |  |  |  |  |  |
|  |  |  | Inverse-variance weighted (fixed) | 16 | 1.05 | 0.95-1.15 | 0.363 | 0.620 |
|  |  |  | Weighted median | 16 | 0.98 | 0.86-1.12 | 0.783 |  |
|  |  |  | MR-PRESSO test | 16 | 1.05 | 0.96-1.14 | 0.339 |  |
|  |  |  | MR-Egger | 16 | / | / | 0.144* |  |
| Phascolarctobacterium | 2.71% | 39.22 |  |  |  |  |  |  |
|  |  |  | Inverse-variance weighted (random) | 13 | 1.01 | 0.83-1.23 | 0.934 | 0.034 |
|  |  |  | Weighted median | 13 | 0.99 | 0.80-1.24 | 0.962 |  |
|  |  |  | MR-PRESSO test | 13 | 1.01 | 0.83-1.23 | 0.935 |  |
|  |  |  | MR-Egger | 13 | / | / | 0.972* |  |
| Prevotella7 | 7.34% | 121.04 |  |  |  |  |  |  |
|  |  |  | Inverse-variance weighted (fixed) | 12 | 0.98 | 0.90-1.08 | 0.724 | 0.161 |
|  |  |  | Weighted median | 12 | 0.99 | 0.87-1.14 | 0.923 |  |
|  |  |  | MR-PRESSO test | 12 | 0.98 | 0.88-1.10 | 0.772 |  |
|  | 5.00% | 48.25 | MR-Egger | 12 | / | / | 0.687* |  |
| Prevotella9 |  |  |  |  |  |  |  |  |
|  |  |  | Inverse-variance weighted (fixed) | 20 | 1.01 | 0.90-1.13 | 0.882 | 0.600 |
|  |  |  | Weighted median | 20 | 0.95 | 0.80-1.12 | 0.533 |  |
|  |  |  | MR-PRESSO test | 20 | 1.01 | 0.91-1.12 | 0.876 |  |
|  |  |  | MR-Egger | 20 | / | / | 0.142* |  |
| RikenellaceaeRC9 | 9.72% | 131.46 |  |  |  |  |  |  |
|  |  |  | Inverse-variance weighted (random) | 15 | 0.99 | 0.88-1.10 | 0.816 | 0.015 |
|  |  |  | Weighted median | 15 | 0.93 | 0.82-1.05 | 0.248 |  |
|  |  |  | MR-PRESSO test | 15 | 0.99 | 0.88-1.10 | 0.819 |  |
|  |  |  | MR-Egger | 15 | / | / | 0.027* |  |
| Romboutsia | 2.87% | 36.08 |  |  |  |  |  |  |
|  |  |  | Inverse-variance weighted (fixed) | 15 | 0.97 | 0.84-1.13 | 0.712 | 0.138 |
|  |  |  | Weighted median | 15 | 1.02 | 0.82-1.26 | 0.878 |  |
|  |  |  | MR-PRESSO test | 15 | 0.97 | 0.82-1.16 | 0.761 |  |
|  |  |  | MR-Egger | 15 | / | / | 0.818* |  |
| Roseburia | 2.57% | 26.88 |  |  |  |  |  |  |
|  |  |  | Inverse-variance weighted (fixed) | 18 | 1.05 | 0.90-1.23 | 0.510 | 0.840 |
|  |  |  | Weighted median | 18 | 1.05 | 0.85-1.30 | 0.657 |  |
|  |  |  | MR-PRESSO test | 18 | 1.05 | 0.93-1.20 | 0.431 |  |
|  |  |  | MR-Egger | 18 | / | / | 0.375* |  |
| Ruminiclostridium5 | 2.06% | 25.73 |  |  |  |  |  |  |
|  |  |  | Inverse-variance weighted (random) | 15 | 0.86 | 0.67-1.11 | 0.258 | 0.004 |
|  |  |  | Weighted median | 15 | 1.03 | 0.80-1.33 | 0.808 |  |
|  |  |  | MR-PRESSO test | 15 | 0.86 | 0.67-1.11 | 0.277 |  |
|  |  |  | Outlier corrected (MR-PRESSO) | 14 | 0.95 | 0.78-1.15 | 0.586 |  |
|  |  |  | MR-Egger | 15 | / | / | 0.445* |  |
| Ruminiclostridium6 | 3.28% | 36.50 |  |  |  |  |  |  |
|  |  |  | Inverse-variance weighted (random) | 17 | 0.96 | 0.80-1.16 | 0.707 | 0.022 |
|  |  |  | Weighted median | 17 | 0.87 | 0.71-1.06 | 0.159 |  |
|  |  |  | MR-PRESSO test | 17 | 0.96 | 0.80-1.16 | 0.711 |  |
|  |  |  | Outlier corrected (MR-PRESSO) | 16 | 0.91 | 0.79-1.05 | 0.206 |  |
|  |  |  | MR-Egger | 17 | / | / | 0.186* |  |
| Ruminiclostridium9 | 2.06% | 25.75 |  |  |  |  |  |  |
|  |  |  | Inverse-variance weighted (random) | 15 | 1.11 | 0.88-1.40 | 0.367 | 0.030 |
|  |  |  | Weighted median | 15 | 1.10 | 0.86-1.40 | 0.459 |  |
|  |  |  | MR-PRESSO test | 15 | 1.11 | 0.88-1.40 | 0.383 |  |
|  |  |  | MR-Egger | 15 | / | / | 0.632* |  |
| RuminococcaceaeNK4A214 | 2.76% | 28.93 |  |  |  |  |  |  |
|  |  |  | Inverse-variance weighted (random) | 18 | 0.91 | 0.75-1.10 | 0.332 | 0.024 |
|  |  |  | Weighted median | 18 | 0.99 | 0.79-1.23 | 0.911 |  |
|  |  |  | MR-PRESSO test | 18 | 0.91 | 0.75-1.10 | 0.346 |  |
|  |  |  | MR-Egger | 18 | / | / | 0.762* |  |
| RuminococcaceaeUCG002 | 3.86% | 28.25 |  |  |  |  |  |  |
|  |  |  | Inverse-variance weighted (fixed) | 26 | 0.94 | 0.83-1.07 | 0.361 | 0.703 |
|  |  |  | Weighted median | 26 | 0.92 | 0.77-1.09 | 0.346 |  |
|  |  |  | MR-PRESSO test | 26 | 0.94 | 0.84-1.06 | 0.326 |  |
|  |  |  | MR-Egger | 26 | / | / | 0.612* |  |
| RuminococcaceaeUCG003 | 2.28% | 30.60 |  |  |  |  |  |  |
|  |  |  | Inverse-variance weighted (random) | 14 | 0.88 | 0.68-1.13 | 0.309 | 0.002 |
|  |  |  | Weighted median | 14 | 0.95 | 0.74-1.21 | 0.656 |  |
|  |  |  | MR-PRESSO test | 14 | 0.88 | 0.68-1.13 | 0.327 |  |
|  |  |  | Outlier corrected (MR-PRESSO) | 13 | 0.79 | 0.66-0.95 | 0.026 |  |
|  |  |  | MR-Egger | 14 | / | / | 0.711* |  |
| RuminococcaceaeUCG004 | 2.57% | 43.94 |  |  |  |  |  |  |
|  |  |  | Inverse-variance weighted (fixed) | 11 | 1.07 | 0.92-1.24 | 0.389 | 0.441 |
|  |  |  | Weighted median | 11 | 1.07 | 0.87-1.30 | 0.532 |  |
|  |  |  | MR-PRESSO test | 11 | 1.07 | 0.92-1.24 | 0.409 |  |
|  |  |  | MR-Egger | 11 | / | / | 0.316* |  |
| RuminococcaceaeUCG005 | 2.73% | 30.20 |  |  |  |  |  |  |
|  |  |  | Inverse-variance weighted (fixed) | 17 | 1.00 | 0.87-1.16 | 0.965 | 0.077 |
|  |  |  | Weighted median | 17 | 1.01 | 0.82-1.24 | 0.926 |  |
|  |  |  | MR-PRESSO test | 17 | 1.00 | 0.84-1.20 | 0.972 |  |
|  |  |  | MR-Egger | 17 | / | / | 0.691* |  |
| RuminococcaceaeUCG009 | 4.30% | 59.42 |  |  |  |  |  |  |
|  |  |  | Inverse-variance weighted (fixed) | 13 | 0.91 | 0.81-1.03 | 0.144 | 0.708 |
|  |  |  | Weighted median | 13 | 0.91 | 0.78-1.08 | 0.281 |  |
|  |  |  | MR-PRESSO test | 13 | 0.91 | 0.82-1.01 | 0.116 |  |
|  |  |  | MR-Egger | 13 | / | / | 0.863* |  |
| RuminococcaceaeUCG010 | 1.51% | 35.03 |  |  |  |  |  |  |
|  |  |  | Inverse-variance weighted (fixed) | 8 | 1.02 | 0.84-1.25 | 0.830 | 0.384 |
|  |  |  | Weighted median | 8 | 1.01 | 0.78-1.32 | 0.941 |  |
|  |  |  | MR-PRESSO test | 8 | 1.02 | 0.83-1.26 | 0.841 |  |
|  |  |  | MR-Egger | 8 | / | / | 0.506* |  |
| RuminococcaceaeUCG011 | 4.90% | 118.09 |  |  |  |  |  |  |
|  |  |  | Inverse-variance weighted (fixed) | 8 | 0.96 | 0.87-1.07 | 0.473 | 0.475 |
|  |  |  | Weighted median | 8 | 0.94 | 0.82-1.07 | 0.345 |  |
|  |  |  | MR-PRESSO test | 8 | 0.96 | 0.87-1.07 | 0.483 |  |
|  |  |  | MR-Egger | 8 | / | / | 0.880* |  |
| RuminococcaceaeUCG013 | 2.09% | 26.01 |  |  |  |  |  |  |
|  |  |  | Inverse-variance weighted (fixed) | 15 | 1.07 | 0.91-1.26 | 0.406 | 0.261 |
|  |  |  | Weighted median | 15 | 1.00 | 0.79-1.27 | 0.979 |  |
|  |  |  | MR-PRESSO test | 15 | 1.07 | 0.90-1.28 | 0.462 |  |
|  |  |  | MR-Egger | 15 | / | / | 0.113* |  |
| RuminococcaceaeUCG014 | 2.88% | 31.95 |  |  |  |  |  |  |
|  |  |  | Inverse-variance weighted (fixed) | 17 | 1.23 | 1.06-1.42 | 0.005 | 0.422 |
|  |  |  | Weighted median | 17 | 1.25 | 1.01-1.54 | 0.037 |  |
|  |  |  | MR-PRESSO test | 17 | 1.23 | 1.06-1.42 | 0.015 |  |
|  |  |  | MR-Egger | 17 | / | / | 0.445* |  |
| Ruminococcus1 | 2.14% | 28.60 |  |  |  |  |  |  |
|  |  |  | Inverse-variance weighted (fixed) | 14 | 1.09 | 0.92-1.30 | 0.314 | 0.086 |
|  |  |  | Weighted median | 14 | 1.01 | 0.78-1.30 | 0.952 |  |
|  |  |  | MR-PRESSO test | 14 | 1.09 | 0.88-1.35 | 0.436 |  |
|  |  |  | MR-Egger | 14 | / | / | 0.858* |  |
| Ruminococcus2 | 2.76% | 34.63 |  |  |  |  |  |  |
|  |  |  | Inverse-variance weighted (fixed) | 15 | 0.90 | 0.77-1.06 | 0.209 | 0.808 |
|  |  |  | Weighted median | 15 | 0.85 | 0.69-1.06 | 0.151 |  |
|  |  |  | MR-PRESSO test | 15 | 0.90 | 0.80-1.03 | 0.147 |  |
|  |  |  | MR-Egger | 15 | / | / | 0.194* |  |
| Sellimonas | 9.32% | 144.84 |  |  |  |  |  |  |
|  |  |  | Inverse-variance weighted (fixed) | 13 | 0.98 | 0.90-1.06 | 0.593 | 0.436 |
|  |  |  | Weighted median | 13 | 0.94 | 0.84-1.05 | 0.284 |  |
|  |  |  | MR-PRESSO test | 13 | 0.98 | 0.90-1.06 | 0.604 |  |
|  |  |  | MR-Egger | 13 | / | / | 0.702* |  |
| Senegalimassilia | 2.58% | 60.59 |  |  |  |  |  |  |
|  |  |  | Inverse-variance weighted (fixed) | 8 | 1.01 | 0.87-1.17 | 0.896 | 0.459 |
|  |  |  | Weighted median | 8 | 1.02 | 0.84-1.23 | 0.870 |  |
|  |  |  | MR-PRESSO test | 8 | 1.01 | 0.87-1.17 | 0.897 |  |
|  |  |  | MR-Egger | 8 | / | / | 0.144* |  |
| Slackia | 2.81% | 66.15 |  |  |  |  |  |  |
|  |  |  | Inverse-variance weighted (fixed) | 8 | 0.93 | 0.81-1.07 | 0.306 | 0.581 |
|  |  |  | Weighted median | 8 | 0.91 | 0.76-1.09 | 0.310 |  |
|  |  |  | MR-PRESSO test | 8 | 0.93 | 0.82-1.05 | 0.292 |  |
|  |  |  | MR-Egger | 8 | / | / | 0.869* |  |
| Streptococcus | 2.68% | 26.56 |  |  |  |  |  |  |
|  |  |  | Inverse-variance weighted (fixed) | 19 | 1.02 | 0.88-1.18 | 0.802 | 0.255 |
|  |  |  | Weighted median | 19 | 1.06 | 0.85-1.32 | 0.596 |  |
|  |  |  | MR-PRESSO test | 19 | 1.02 | 0.87-1.20 | 0.821 |  |
|  |  |  | MR-Egger | 19 | / | / | 0.969* |  |
| Subdoligranulum | 1.86% | 24.81 |  |  |  |  |  |  |
|  |  |  | Inverse-variance weighted (fixed) | 14 | 1.14 | 0.96-1.35 | 0.138 | 0.390 |
|  |  |  | Weighted median | 14 | 1.10 | 0.86-1.40 | 0.453 |  |
|  |  |  | MR-PRESSO test | 14 | 1.14 | 0.95-1.36 | 0.174 |  |
|  |  |  | MR-Egger | 14 | / | / | 0.420* |  |
| Sutterella | 1.97% | 30.70 |  |  |  |  |  |  |
|  |  |  | Inverse-variance weighted (fixed) | 12 | 0.90 | 0.76-1.06 | 0.205 | 0.499 |
|  |  |  | Weighted median | 12 | 0.90 | 0.73-1.11 | 0.319 |  |
|  |  |  | MR-PRESSO test | 12 | 0.90 | 0.76-1.06 | 0.218 |  |
|  |  |  | MR-Egger | 12 | / | / | 0.623* |  |
| Terrisporobacter | 2.14% | 66.94 |  |  |  |  |  |  |
|  |  |  | Inverse-variance weighted (fixed) | 6 | 0.96 | 0.82-1.13 | 0.630 | 0.421 |
|  |  |  | Weighted median | 6 | 0.95 | 0.77-1.17 | 0.622 |  |
|  |  |  | MR-PRESSO test | 6 | 0.96 | 0.82-1.13 | 0.649 |  |
|  |  |  | MR-Egger | 6 | / | / | 0.765* |  |
| Turicibacter | 3.35% | 45.43 |  |  |  |  |  |  |
|  |  |  | Inverse-variance weighted (fixed) | 14 | 1.15 | 1.01-1.31 | 0.033 | 0.803 |
|  |  |  | Weighted median | 14 | 1.15 | 0.97-1.36 | 0.110 |  |
|  |  |  | MR-PRESSO test | 14 | 1.15 | 1.04-1.28 | 0.021 |  |
|  |  |  | MR-Egger | 14 | / | / | 0.104* |  |
| Tyzzerella3 | 6.44% | 90.12 |  |  |  |  |  |  |
|  |  |  | Inverse-variance weighted (fixed) | 14 | 1.04 | 0.94-1.15 | 0.442 | 0.877 |
|  |  |  | Weighted median | 14 | 1.00 | 0.87-1.14 | 0.982 |  |
|  |  |  | MR-PRESSO test | 14 | 1.04 | 0.96-1.12 | 0.329 |  |
|  |  |  | MR-Egger | 14 | / | / | 0.180* |  |
| Veillonella | 2.77% | 47.44 |  |  |  |  |  |  |
|  |  |  | Inverse-variance weighted (fixed) | 11 | 0.91 | 0.78-1.06 | 0.211 | 0.288 |
|  |  |  | Weighted median | 11 | 0.97 | 0.78-1.20 | 0.752 |  |
|  |  |  | MR-PRESSO test | 11 | 0.91 | 0.77-1.07 | 0.279 |  |
|  |  |  | MR-Egger | 11 | / | / | 0.577* |  |
| Victivallis | 7.29% | 130.96 |  |  |  |  |  |  |
|  |  |  | Inverse-variance weighted (fixed) | 11 | 1.08 | 0.98-1.18 | 0.111 | 0.905 |
|  |  |  | Weighted median | 11 | 1.06 | 0.94-1.20 | 0.324 |  |
|  |  |  | MR-PRESSO test | 11 | 1.08 | 1.01-1.14 | 0.044 |  |
|  |  |  | MR-Egger | 11 | / | / | 0.752* |  |

Abbreviations: CI, confidence interval; MR, Mendelian randomization; MR-PRESSO test, MR Pleiotropy RESidual Sum and Outlier test; OR, odds ratio; SNP, single nucleotide polymorphism. **P*-value of the intercept from MR-Egger regression analysis.

**Supplementary Table 3** Effect estimates of the associations between 196 bacterial traits and risk of ulcerative colitis in MR analyses

| Gut microbiota | R^2^ | F statistic | Methods | N.SNP | OR | 95% CI | *P*-value | Qrs |
| --- | --- | --- | --- | --- | --- | --- | --- | --- |
| Phylum |  |  |  |  |  |  |  |  |
| Actinobacteria | 2.80% | 26.43 |  |  |  |  |  |  |
|  |  |  | Inverse-variance weighted (fixed) | 20 | 0.96 | 0.80-1.16 | 0.689 | 0.645 |
|  |  |  | Weighted median | 20 | 0.91 | 0.71-1.17 | 0.470 |  |
|  |  |  | MR-PRESSO test | 20 | 0.96 | 0.81-1.14 | 0.669 |  |
|  |  |  | MR-Egger | 20 | / | / | 0.373* |  |
| Bacteroidetes | 2.21% | 29.61 |  |  |  |  |  |  |
|  |  |  | Inverse-variance weighted (fixed) | 14 | 0.93 | 0.75-1.14 | 0.475 | 0.617 |
|  |  |  | Weighted median | 14 | 0.95 | 0.72-1.27 | 0.750 |  |
|  |  |  | MR-PRESSO test | 14 | 0.93 | 0.77-1.12 | 0.450 |  |
|  |  |  | MR-Egger | 14 | / | / | 0.061* |  |
| Cyanobacteria | 3.53% | 67.16 |  |  |  |  |  |  |
|  |  |  | Inverse-variance weighted (fixed) | 10 | 0.95 | 0.81-1.12 | 0.550 | 0.393 |
|  |  |  | Weighted median | 10 | 0.95 | 0.76-1.19 | 0.674 |  |
|  |  |  | MR-PRESSO test | 10 | 0.95 | 0.81-1.12 | 0.575 |  |
|  |  |  | MR-Egger | 10 | / | / | 0.466* |  |
| Euryarchaeota | 7.39% | 112.50 |  |  |  |  |  |  |
|  |  |  | Inverse-variance weighted (fixed) | 13 | 0.95 | 0.85-1.06 | 0.364 | 0.060 |
|  |  |  | Weighted median | 13 | 1.00 | 0.86-1.16 | 0.995 |  |
|  |  |  | MR-PRESSO test | 13 | 0.95 | 0.82-1.10 | 0.500 |  |
|  |  |  | MR-Egger | 13 | / | / | 0.446* |  |
| Firmicutes | 2.49% | 24.65 |  |  |  |  |  |  |
|  |  |  | Inverse-variance weighted (fixed) | 19 | 1.05 | 0.87-1.27 | 0.621 | 0.062 |
|  |  |  | Weighted median | 19 | 0.97 | 0.73-1.28 | 0.811 |  |
|  |  |  | MR-PRESSO test | 19 | 1.05 | 0.83-1.33 | 0.696 |  |
|  |  |  | MR-Egger | 19 | / | / | 0.771* |  |
| Lentisphaerae | 5.25% | 92.39 |  |  |  |  |  |  |
|  |  |  | Inverse-variance weighted (fixed) | 11 | 0.85 | 0.74-0.97 | 0.015 | 0.464 |
|  |  |  | Weighted median | 11 | 0.80 | 0.66-0.97 | 0.020 |  |
|  |  |  | MR-PRESSO test | 11 | 0.85 | 0.74-0.97 | 0.034 |  |
|  |  |  | MR-Egger | 11 | / | / | 0.903* |  |
| Proteobacteria | 1.39% | 28.61 |  |  |  |  |  |  |
|  |  |  | Inverse-variance weighted (random) | 13 | 1.02 | 0.74-1.41 | 0.914 | 0.032 |
|  |  |  | Weighted median | 13 | 1.05 | 0.73-1.51 | 0.792 |  |
|  |  |  | MR-PRESSO test | 13 | 1.02 | 0.74-1.41 | 0.916 |  |
|  |  |  | MR-Egger | 13 | / | / | 0.529* |  |
| Tenericutes | 2.60% | 40.77 |  |  |  |  |  |  |
|  |  |  | Inverse-variance weighted (fixed) | 12 | 0.99 | 0.82-1.20 | 0.948 | 0.206 |
|  |  |  | Weighted median | 12 | 0.92 | 0.69-1.23 | 0.584 |  |
|  |  |  | MR-PRESSO test | 12 | 0.99 | 0.80-1.24 | 0.956 |  |
|  |  |  | MR-Egger | 12 | / | / | 0.327* |  |
| Verrucomicrobia | 2.42% | 37.94 |  |  |  |  |  |  |
|  |  |  | Inverse-variance weighted (fixed) | 12 | 1.05 | 0.86-1.27 | 0.624 | 0.334 |
|  |  |  | Weighted median | 12 | 1.00 | 0.77-1.31 | 0.998 |  |
|  |  |  | MR-PRESSO test | 12 | 1.05 | 0.85-1.29 | 0.653 |  |
|  |  |  | MR-Egger | 12 | / | / | 0.390* |  |
| Class |  |  |  |  |  |  |  |  |
| Actinobacteria | 4.64% | 38.76 |  |  |  |  |  |  |
|  |  |  | Inverse-variance weighted (fixed) | 23 | 0.87 | 0.75-1.02 | 0.094 | 0.196 |
|  |  |  | Weighted median | 23 | 0.93 | 0.74-1.16 | 0.522 |  |
|  |  |  | MR-PRESSO test | 23 | 0.87 | 0.73-1.04 | 0.147 |  |
|  |  |  | MR-Egger | 23 | / | / | 0.830* |  |
| Alphaproteobacteria | 2.30% | 43.24 |  |  |  |  |  |  |
|  |  |  | Inverse-variance weighted (fixed) | 10 | 1.13 | 0.93-1.39 | 0.226 | 0.213 |
|  |  |  | Weighted median | 10 | 1.07 | 0.80-1.43 | 0.651 |  |
|  |  |  | MR-PRESSO test | 10 | 1.13 | 0.90-1.43 | 0.322 |  |
|  |  |  | MR-Egger | 10 | / | / | 0.586* |  |
| Bacilli | 3.50% | 30.17 |  |  |  |  |  |  |
|  |  |  | Inverse-variance weighted (fixed) | 22 | 0.94 | 0.80-1.12 | 0.511 | 0.109 |
|  |  |  | Weighted median | 22 | 0.94 | 0.73-1.20 | 0.608 |  |
|  |  |  | MR-PRESSO test | 22 | 0.94 | 0.77-1.15 | 0.583 |  |
|  |  |  | MR-Egger | 22 | / | / | 0.872* |  |
| Bacteroidia | 2.28% | 28.54 |  |  |  |  |  |  |
|  |  |  | Inverse-variance weighted (fixed) | 15 | 0.90 | 0.74-1.11 | 0.319 | 0.432 |
|  |  |  | Weighted median | 15 | 0.86 | 0.65-1.15 | 0.309 |  |
|  |  |  | MR-PRESSO test | 15 | 0.90 | 0.73-1.11 | 0.340 |  |
|  |  |  | MR-Egger | 15 | / | / | 0.030* |  |
|  | 2.02% | 25.21 |  |  |  |  |  |  |
| Betaproteobacteria |  |  | Inverse-variance weighted (fixed) | 15 | 1.09 | 0.88-1.34 | 0.446 | 0.581 |
|  |  |  | Weighted median | 15 | 1.06 | 0.80-1.41 | 0.668 |  |
|  |  |  | MR-PRESSO test | 15 | 1.09 | 0.89-1.33 | 0.430 |  |
|  |  |  | MR-Egger | 15 | / | / | 0.984* |  |
| Clostridia | 1.97% | 21.63 |  |  |  |  |  |  |
|  |  |  | Inverse-variance weighted (fixed) | 17 | 1.21 | 0.98-1.51 | 0.077 | 0.606 |
|  |  |  | Weighted median | 17 | 1.42 | 1.05-1.92 | 0.024 |  |
|  |  |  | MR-PRESSO test | 17 | 1.21 | 0.99-1.49 | 0.076 |  |
|  |  |  | MR-Egger | 17 | / | / | 0.605* |  |
| Coriobacteriia | 2.99% | 26.93 |  |  |  |  |  |  |
|  |  |  | Inverse-variance weighted (fixed) | 21 | 1.11 | 0.93-1.33 | 0.257 | 0.202 |
|  |  |  | Weighted median | 21 | 1.12 | 0.86-1.48 | 0.398 |  |
|  |  |  | MR-PRESSO test | 21 | 1.11 | 0.91-1.36 | 0.323 |  |
|  |  |  | MR-Egger | 21 | / | / | 0.737* |  |
| Deltaproteobacteria | 2.22% | 29.78 |  |  |  |  |  |  |
|  |  |  | Inverse-variance weighted (fixed) | 14 | 1.04 | 0.85-1.27 | 0.718 | 0.220 |
|  |  |  | Weighted median | 14 | 1.04 | 0.79-1.38 | 0.780 |  |
|  |  |  | MR-PRESSO test | 14 | 1.04 | 0.83-1.30 | 0.754 |  |
|  |  |  | MR-Egger | 14 | / | / | 0.753* |  |
| Erysipelotrichia | 1.65% | 23.71 |  |  |  |  |  |  |
|  |  |  | Inverse-variance weighted (fixed) | 13 | 0.95 | 0.74-1.21 | 0.664 | 0.766 |
|  |  |  | Weighted median | 13 | 1.05 | 0.76-1.46 | 0.764 |  |
|  |  |  | MR-PRESSO test | 13 | 0.95 | 0.78-1.16 | 0.610 |  |
|  |  |  | MR-Egger | 13 | / | / | 0.527* |  |
| Gammaproteobacteria | 1.39% | 28.61 |  |  |  |  |  |  |
|  |  |  | Inverse-variance weighted (fixed) | 9 | 0.92 | 0.71-1.19 | 0.530 | 0.559 |
|  |  |  | Weighted median | 9 | 0.90 | 0.64-1.26 | 0.543 |  |
|  |  |  | MR-PRESSO test | 9 | 0.92 | 0.73-1.17 | 0.515 |  |
|  |  |  | MR-Egger | 9 | / | / | 0.494* |  |
| Lentisphaeria | 4.81% | 92.58 |  |  |  |  |  |  |
|  |  |  | Inverse-variance weighted (fixed) | 10 | 0.86 | 0.75-0.99 | 0.032 | 0.395 |
|  |  |  | Weighted median | 10 | 0.89 | 0.73-1.09 | 0.256 |  |
|  |  |  | MR-PRESSO test | 10 | 0.86 | 0.74-0.99 | 0.066 |  |
|  |  |  | MR-Egger | 10 | / | / | 0.815* |  |
| Melainabacteria | 5.25% | 78.13 |  |  |  |  |  |  |
|  |  |  | Inverse-variance weighted (fixed) | 13 | 0.99 | 0.87-1.13 | 0.866 | 0.334 |
|  |  |  | Weighted median | 13 | 0.98 | 0.82-1.17 | 0.854 |  |
|  |  |  | MR-PRESSO test | 13 | 0.99 | 0.86-1.14 | 0.876 |  |
|  |  |  | MR-Egger | 13 | / | / | 0.620* |  |
| Methanobacteria | 7.23% | 119.05 |  |  |  |  |  |  |
|  |  |  | Inverse-variance weighted (fixed) | 12 | 0.95 | 0.85-1.06 | 0.340 | 0.075 |
|  |  |  | Weighted median | 12 | 0.92 | 0.79-1.07 | 0.269 |  |
|  |  |  | MR-PRESSO test | 12 | 0.95 | 0.82-1.09 | 0.475 |  |
|  |  |  | MR-Egger | 12 | / | / | 0.856* |  |
| Mollicutes | 2.60% | 40.77 |  |  |  |  |  |  |
|  |  |  | Inverse-variance weighted (fixed) | 12 | 0.99 | 0.82-1.20 | 0.948 | 0.206 |
|  |  |  | Weighted median | 12 | 0.92 | 0.69-1.23 | 0.584 |  |
|  |  |  | MR-PRESSO test | 12 | 0.99 | 0.80-1.24 | 0.956 |  |
|  |  |  | MR-Egger | 12 | / | / | 0.327* |  |
| Negativicutes | 1.67% | 23.92 |  |  |  |  |  |  |
|  |  |  | Inverse-variance weighted (fixed) | 13 | 0.91 | 0.72-1.16 | 0.446 | 0.421 |
|  |  |  | Weighted median | 13 | 0.88 | 0.65-1.21 | 0.445 |  |
|  |  |  | MR-PRESSO test | 13 | 0.91 | 0.72-1.16 | 0.466 |  |
|  |  |  | MR-Egger | 13 | / | / | 0.535* |  |
| Verrucomicrobiae | 2.63% | 38.04 |  |  |  |  |  |  |
|  |  |  | Inverse-variance weighted (fixed) | 13 | 1.00 | 0.83-1.21 | 0.975 | 0.158 |
|  |  |  | Weighted median | 13 | 0.96 | 0.72-1.28 | 0.755 |  |
|  |  |  | MR-PRESSO test | 13 | 1.00 | 0.80-1.25 | 0.979 |  |
|  |  |  | MR-Egger | 13 | / | / | 0.026* |  |
| Order |  |  |  |  |  |  |  |  |
| Actinomycetales | 1.75% | 65.35 |  |  |  |  |  |  |
|  |  |  | Inverse-variance weighted (fixed) | 5 | 1.08 | 0.84-1.39 | 0.548 | 0.699 |
|  |  |  | Weighted median | 5 | 1.20 | 0.86-1.67 | 0.280 |  |
|  |  |  | MR-PRESSO test | 5 | 1.08 | 0.90-1.30 | 0.464 |  |
|  |  |  | MR-Egger | 5 | / | / | 0.524* |  |
| Bacillales | 7.14% | 128.04 |  |  |  |  |  |  |
|  |  |  | Inverse-variance weighted (fixed) | 11 | 0.89 | 0.80-1.00 | 0.045 | 0.895 |
|  |  |  | Weighted median | 11 | 0.91 | 0.79-1.05 | 0.195 |  |
|  |  |  | MR-PRESSO test | 11 | 0.89 | 0.82-0.96 | 0.017 |  |
|  |  |  | MR-Egger | 11 | / | / | 0.625* |  |
| Bacteroidales | 2.28% | 28.54 |  |  |  |  |  |  |
|  |  |  | Inverse-variance weighted (fixed) | 15 | 0.90 | 0.74-1.11 | 0.319 | 0.432 |
|  |  |  | Weighted median | 15 | 0.86 | 0.65-1.15 | 0.309 |  |
|  |  |  | MR-PRESSO test | 15 | 0.90 | 0.73-1.11 | 0.340 |  |
|  |  |  | MR-Egger | 15 | / | / | 0.030* |  |
| Bifidobacteriales | 4.44% | 34.00 |  |  |  |  |  |  |
|  |  |  | Inverse-variance weighted (fixed) | 25 | 0.86 | 0.74-1.00 | 0.045 | 0.069 |
|  |  |  | Weighted median | 25 | 0.93 | 0.74-1.18 | 0.564 |  |
|  |  |  | MR-PRESSO test | 25 | 0.86 | 0.71-1.03 | 0.109 |  |
|  |  |  | MR-Egger | 25 | / | / | 0.422* |  |
| Burkholderiales | 1.76% | 25.27 |  |  |  |  |  |  |
|  |  |  | Inverse-variance weighted (fixed) | 13 | 1.11 | 0.88-1.39 | 0.387 | 0.390 |
|  |  |  | Weighted median | 13 | 1.08 | 0.79-1.49 | 0.616 |  |
|  |  |  | MR-PRESSO test | 13 | 1.11 | 0.87-1.40 | 0.417 |  |
|  |  |  | MR-Egger | 13 | / | / | 0.832* |  |
| Clostridiales | 1.97% | 21.66 |  |  |  |  |  |  |
|  |  |  | Inverse-variance weighted (fixed) | 17 | 1.23 | 0.99-1.53 | 0.062 | 0.632 |
|  |  |  | Weighted median | 17 | 1.46 | 1.08-1.97 | 0.015 |  |
|  |  |  | MR-PRESSO test | 17 | 1.23 | 1.01-1.50 | 0.059 |  |
|  |  |  | MR-Egger | 17 | / | / | 0.551* |  |
| Coriobacteriales | 2.99% | 26.93 |  |  |  |  |  |  |
|  |  |  | Inverse-variance weighted (fixed) | 21 | 1.11 | 0.93-1.33 | 0.257 | 0.202 |
|  |  |  | Weighted median | 21 | 1.12 | 0.86-1.48 | 0.398 |  |
|  |  |  | MR-PRESSO test | 21 | 1.11 | 0.91-1.36 | 0.323 |  |
|  |  |  | MR-Egger | 21 | / | / | 0.737* |  |
| Desulfovibrionales | 2.13% | 30.69 |  |  |  |  |  |  |
|  |  |  | Inverse-variance weighted (fixed) | 13 | 1.17 | 0.95-1.43 | 0.136 | 0.448 |
|  |  |  | Weighted median | 13 | 1.13 | 0.85-1.50 | 0.404 |  |
|  |  |  | MR-PRESSO test | 13 | 1.17 | 0.95-1.43 | 0.161 |  |
|  |  |  | MR-Egger | 13 | / | / | 0.690* |  |
| Enterobacteriales | 1.84% | 31.17 |  |  |  |  |  |  |
|  |  |  | Inverse-variance weighted (fixed) | 11 | 1.15 | 0.90-1.45 | 0.261 | 0.934 |
|  |  |  | Weighted median | 11 | 1.13 | 0.85-1.51 | 0.408 |  |
|  |  |  | MR-PRESSO test | 11 | 1.15 | 0.98-1.34 | 0.117 |  |
|  |  |  | MR-Egger | 11 | / | / | 0.238* |  |
| Erysipelotrichales | 1.65% | 23.71 |  |  |  |  |  |  |
|  |  |  | Inverse-variance weighted (fixed) | 13 | 0.95 | 0.74-1.21 | 0.664 | 0.766 |
|  |  |  | Weighted median | 13 | 1.05 | 0.76-1.46 | 0.764 |  |
|  |  |  | MR-PRESSO test | 13 | 0.95 | 0.78-1.16 | 0.610 |  |
|  |  |  | MR-Egger | 13 | / | / | 0.527* |  |
| Gastranaerophilales | 4.90% | 78.66 |  |  |  |  |  |  |
|  |  |  | Inverse-variance weighted (fixed) | 12 | 1.03 | 0.90-1.18 | 0.666 | 0.201 |
|  |  |  | Weighted median | 12 | 1.00 | 0.82-1.20 | 0.961 |  |
|  |  |  | MR-PRESSO test | 12 | 1.03 | 0.88-1.20 | 0.715 |  |
|  |  |  | MR-Egger | 12 | / | / | 0.914* |  |
| Lactobacillales | 3.12% | 31.02 |  |  |  |  |  |  |
|  |  |  | Inverse-variance weighted (fixed) | 19 | 0.91 | 0.76-1.10 | 0.331 | 0.122 |
|  |  |  | Weighted median | 19 | 0.92 | 0.71-1.21 | 0.556 |  |
|  |  |  | MR-PRESSO test | 19 | 0.91 | 0.74-1.13 | 0.422 |  |
|  |  |  | MR-Egger | 19 | / | / | 0.627* |  |
| Methanobacteriales | 7.23% | 119.05 |  |  |  |  |  |  |
|  |  |  | Inverse-variance weighted (fixed) | 12 | 0.95 | 0.85-1.06 | 0.340 | 0.075 |
|  |  |  | Weighted median | 12 | 0.92 | 0.79-1.07 | 0.269 |  |
|  |  |  | MR-PRESSO test | 12 | 0.95 | 0.82-1.09 | 0.475 |  |
|  |  |  | MR-Egger | 12 | / | / | 0.856* |  |
| MollicutesRF9 | 3.80% | 42.57 |  |  |  |  |  |  |
|  |  |  | Inverse-variance weighted (fixed) | 17 | 0.92 | 0.78-1.07 | 0.265 | 0.984 |
|  |  |  | Weighted median | 17 | 0.90 | 0.73-1.10 | 0.298 |  |
|  |  |  | MR-PRESSO test | 17 | 0.92 | 0.83-1.01 | 0.096 |  |
|  |  |  | MR-Egger | 17 | / | / | 0.255* |  |
| NB1n | 7.59% | 88.52 |  |  |  |  |  |  |
|  |  |  | Inverse-variance weighted (random) | 17 | 1.11 | 0.95-1.29 | 0.178 | 0.021 |
|  |  |  | Weighted median | 17 | 1.10 | 0.93-1.29 | 0.254 |  |
|  |  |  | MR-PRESSO test | 17 | 1.11 | 0.95-1.29 | 0.196 |  |
|  |  |  | MR-Egger | 17 | / | / | 0.029* |  |
| Pasteurellales | 5.52% | 56.29 |  |  |  |  |  |  |
|  |  |  | Inverse-variance weighted (fixed) | 19 | 1.04 | 0.92-1.19 | 0.515 | 0.197 |
|  |  |  | Weighted median | 19 | 1.01 | 0.84-1.23 | 0.882 |  |
|  |  |  | MR-PRESSO test | 19 | 1.04 | 0.90-1.21 | 0.571 |  |
|  |  |  | MR-Egger | 19 | / | / | 0.155* |  |
| Rhodospirillales | 3.39% | 45.92 |  |  |  |  |  |  |
|  |  |  | Inverse-variance weighted (random) | 14 | 1.10 | 0.86-1.40 | 0.441 | 0.005 |
|  |  |  | Weighted median | 14 | 1.15 | 0.91-1.47 | 0.245 |  |
|  |  |  | MR-PRESSO test | 14 | 1.10 | 0.86-1.40 | 0.455 |  |
|  |  |  | Outlier corrected (MR-PRESSO) | 13 | 1.18 | 0.95-1.46 | 0.160 |  |
|  |  |  | MR-Egger | 14 | / | / | 0.674* |  |
| Selenomonadales | 1.67% | 23.92 |  |  |  |  |  |  |
|  |  |  | Inverse-variance weighted (fixed) | 13 | 0.91 | 0.72-1.16 | 0.446 | 0.421 |
|  |  |  | Weighted median | 13 | 0.88 | 0.65-1.21 | 0.445 |  |
|  |  |  | MR-PRESSO test | 13 | 0.91 | 0.72-1.16 | 0.466 |  |
|  |  |  | MR-Egger | 13 | / | / | 0.535* |  |
| Verrucomicrobiales | 2.63% | 38.04 |  |  |  |  |  |  |
|  |  |  | Inverse-variance weighted (fixed) | 13 | 1.00 | 0.83-1.21 | 0.975 | 0.158 |
|  |  |  | Weighted median | 13 | 0.96 | 0.72-1.28 | 0.755 |  |
|  |  |  | MR-PRESSO test | 13 | 1.00 | 0.80-1.25 | 0.979 |  |
|  |  |  | MR-Egger | 13 | / | / | 0.026* |  |
| Victivallales | 4.81% | 92.58 |  |  |  |  |  |  |
|  |  |  | Inverse-variance weighted (fixed) | 10 | 0.86 | 0.75-0.99 | 0.032 | 0.395 |
|  |  |  | Weighted median | 10 | 0.89 | 0.73-1.09 | 0.256 |  |
|  |  |  | MR-PRESSO test | 10 | 0.86 | 0.74-0.99 | 0.066 |  |
|  |  |  | MR-Egger | 10 | / | / | 0.815* |  |
| Family |  |  |  |  |  |  |  |  |
| Acidaminococcaceae | 1.50% | 35.00 |  |  |  |  |  |  |
|  |  |  | Inverse-variance weighted (fixed) | 8 | 0.82 | 0.64-1.05 | 0.123 | 0.968 |
|  |  |  | Weighted median | 8 | 0.80 | 0.58-1.10 | 0.164 |  |
|  |  |  | MR-PRESSO test | 8 | 0.82 | 0.72-0.93 | 0.020 |  |
|  |  |  | MR-Egger | 8 | / | / | 0.917* |  |
| Actinomycetaceae | 1.76% | 65.52 |  |  |  |  |  |  |
|  |  |  | Inverse-variance weighted (fixed) | 5 | 1.08 | 0.84-1.39 | 0.549 | 0.698 |
|  |  |  | Weighted median | 5 | 1.20 | 0.86-1.66 | 0.280 |  |
|  |  |  | MR-PRESSO test | 5 | 1.08 | 0.90-1.30 | 0.464 |  |
|  |  |  | MR-Egger | 5 | / | / | 0.525* |  |
| Alcaligenaceae | 2.77% | 26.11 |  |  |  |  |  |  |
|  |  |  | Inverse-variance weighted (fixed) | 20 | 1.00 | 0.83-1.20 | 0.964 | 0.778 |
|  |  |  | Weighted median | 20 | 1.04 | 0.81-1.34 | 0.745 |  |
|  |  |  | MR-PRESSO test | 20 | 1.00 | 0.85-1.17 | 0.958 |  |
|  |  |  | MR-Egger | 20 | / | / | 0.953* |  |
| Bacteroidaceae | 1.23% | 22.84 |  |  |  |  |  |  |
|  |  |  | Inverse-variance weighted (fixed) | 10 | 0.96 | 0.73-1.27 | 0.790 | 0.555 |
|  |  |  | Weighted median | 10 | 0.98 | 0.68-1.42 | 0.927 |  |
|  |  |  | MR-PRESSO test | 10 | 0.96 | 0.75-1.24 | 0.782 |  |
|  |  |  | MR-Egger | 10 | / | / | 0.762* |  |
| BacteroidalesS24.7 | 3.06% | 57.94 |  |  |  |  |  |  |
|  |  |  | Inverse-variance weighted (fixed) | 10 | 0.97 | 0.82-1.15 | 0.756 | 0.674 |
|  |  |  | Weighted median | 10 | 1.03 | 0.83-1.29 | 0.769 |  |
|  |  |  | MR-PRESSO test | 10 | 0.97 | 0.84-1.13 | 0.726 |  |
|  |  |  | MR-Egger | 10 | / | / | 0.152* |  |
| Bifidobacteriaceae | 4.44% | 34.00 |  |  |  |  |  |  |
|  |  |  | Inverse-variance weighted (fixed) | 25 | 0.86 | 0.74-1.00 | 0.045 | 0.069 |
|  |  |  | Weighted median | 25 | 0.93 | 0.74-1.18 | 0.564 |  |
|  |  |  | MR-PRESSO test | 25 | 0.86 | 0.71-1.03 | 0.109 |  |
|  |  |  | MR-Egger | 25 | / | / | 0.422* |  |
| Christensenellaceae | 2.03% | 31.59 |  |  |  |  |  |  |
|  |  |  | Inverse-variance weighted (fixed) | 12 | 0.87 | 0.70-1.08 | 0.210 | 0.444 |
|  |  |  | Weighted median | 12 | 0.77 | 0.58-1.04 | 0.085 |  |
|  |  |  | MR-PRESSO test | 12 | 0.87 | 0.70-1.08 | 0.235 |  |
|  |  |  | MR-Egger | 12 | / | / | 0.830* |  |
| Clostridiaceae1 | 1.71% | 29.00 |  |  |  |  |  |  |
|  |  |  | Inverse-variance weighted (fixed) | 11 | 0.90 | 0.71-1.12 | 0.343 | 0.696 |
|  |  |  | Weighted median | 11 | 0.86 | 0.64-1.15 | 0.302 |  |
|  |  |  | MR-PRESSO test | 11 | 0.90 | 0.74-1.09 | 0.293 |  |
|  |  |  | MR-Egger | 11 | / | / | 0.884* |  |
| ClostridialesvadinBB60 | 3.73% | 41.81 |  |  |  |  |  |  |
|  |  |  | Inverse-variance weighted (fixed) | 17 | 0.94 | 0.80-1.09 | 0.393 | 0.904 |
|  |  |  | Weighted median | 17 | 0.97 | 0.79-1.19 | 0.785 |  |
|  |  |  | MR-PRESSO test | 17 | 0.94 | 0.83-1.05 | 0.277 |  |
|  |  |  | MR-Egger | 17 | / | / | 0.980* |  |
| Coriobacteriaceae | 2.99% | 26.93 |  |  |  |  |  |  |
|  |  |  | Inverse-variance weighted (fixed) | 21 | 1.11 | 0.93-1.33 | 0.257 | 0.202 |
|  |  |  | Weighted median | 21 | 1.12 | 0.86-1.48 | 0.398 |  |
|  |  |  | MR-PRESSO test | 21 | 1.11 | 0.91-1.36 | 0.323 |  |
|  |  |  | MR-Egger | 21 | / | / | 0.737* |  |
| Defluviitaleaceae | 3.59% | 52.53 |  |  |  |  |  |  |
|  |  |  | Inverse-variance weighted (fixed) | 13 | 0.88 | 0.75-1.04 | 0.136 | 0.308 |
|  |  |  | Weighted median | 13 | 0.96 | 0.77-1.21 | 0.756 |  |
|  |  |  | MR-PRESSO test | 13 | 0.88 | 0.74-1.05 | 0.191 |  |
|  |  |  | MR-Egger | 13 | / | / | 0.634* |  |
| Desulfovibrionaceae | 2.00% | 31.20 |  |  |  |  |  |  |
|  |  |  | Inverse-variance weighted (fixed) | 12 | 1.14 | 0.92-1.40 | 0.236 | 0.440 |
|  |  |  | Weighted median | 12 | 1.10 | 0.82-1.48 | 0.526 |  |
|  |  |  | MR-PRESSO test | 12 | 1.14 | 0.92-1.40 | 0.261 |  |
|  |  |  | MR-Egger | 12 | / | / | 0.886* |  |
| Enterobacteriaceae | 1.84% | 31.17 |  |  |  |  |  |  |
|  |  |  | Inverse-variance weighted (fixed) | 11 | 1.15 | 0.90-1.45 | 0.261 | 0.934 |
|  |  |  | Weighted median | 11 | 1.13 | 0.85-1.51 | 0.408 |  |
|  |  |  | MR-PRESSO test | 11 | 1.15 | 0.98-1.34 | 0.117 |  |
|  |  |  | MR-Egger | 11 | / | / | 0.238* |  |
| Erysipelotrichaceae | 1.65% | 23.71 |  |  |  |  |  |  |
|  |  |  | Inverse-variance weighted (fixed) | 13 | 0.95 | 0.74-1.21 | 0.664 | 0.766 |
|  |  |  | Weighted median | 13 | 1.05 | 0.76-1.46 | 0.764 |  |
|  |  |  | MR-PRESSO test | 13 | 0.95 | 0.78-1.16 | 0.610 |  |
|  |  |  | MR-Egger | 13 | / | / | 0.527* |  |
| FamilyXI (ID: 1936) | 6.97% | 137.38 |  |  |  |  |  |  |
|  |  |  | Inverse-variance weighted (fixed) | 10 | 0.99 | 0.89-1.11 | 0.875 | 0.539 |
|  |  |  | Weighted median | 10 | 1.03 | 0.89-1.20 | 0.660 |  |
|  |  |  | MR-PRESSO test | 10 | 0.99 | 0.89-1.10 | 0.871 |  |
|  |  |  | MR-Egger | 10 | / | / | 0.477* |  |
| FamilyXIII (ID: 1957) | 2.24% | 30.01 |  |  |  |  |  |  |
|  |  |  | Inverse-variance weighted (fixed) | 12 | 0.94 | 0.74-1.20 | 0.625 | 0.700 |
|  |  |  | Weighted median | 12 | 0.95 | 0.70-1.29 | 0.734 |  |
|  |  |  | MR-PRESSO test | 12 | 0.94 | 0.76-1.16 | 0.582 |  |
|  |  |  | MR-Egger | 12 | / | / | 0.658* |  |
| Lachnospiraceae | 2.61% | 27.33 |  |  |  |  |  |  |
|  |  |  | Inverse-variance weighted (fixed) | 18 | 1.13 | 0.94-1.37 | 0.192 | 0.511 |
|  |  |  | Weighted median | 18 | 1.18 | 0.90-1.55 | 0.235 |  |
|  |  |  | MR-PRESSO test | 18 | 1.13 | 0.94-1.37 | 0.199 |  |
|  |  |  | MR-Egger | 18 | / | / | 0.339* |  |
| Lactobacillaceae | 4.43% | 65.42 |  |  |  |  |  |  |
|  |  |  | Inverse-variance weighted (fixed) | 13 | 0.87 | 0.76-1.01 | 0.071 | 0.366 |
|  |  |  | Weighted median | 13 | 0.89 | 0.73-1.10 | 0.295 |  |
|  |  |  | MR-PRESSO test | 13 | 0.87 | 0.75-1.02 | 0.109 |  |
|  |  |  | MR-Egger | 13 | / | / | 0.877* |  |
| Methanobacteriaceae | 7.23% | 119.05 |  |  |  |  |  |  |
|  |  |  | Inverse-variance weighted (fixed) | 12 | 0.95 | 0.85-1.06 | 0.340 | 0.075 |
|  |  |  | Weighted median | 12 | 0.92 | 0.79-1.07 | 0.269 |  |
|  |  |  | MR-PRESSO test | 12 | 0.95 | 0.82-1.09 | 0.475 |  |
|  |  |  | MR-Egger | 12 | / | / | 0.856* |  |
| Oxalobacteraceae | 6.93% | 90.93 |  |  |  |  |  |  |
|  |  |  | Inverse-variance weighted (fixed) | 15 | 1.14 | 1.01-1.28 | 0.034 | 0.294 |
|  |  |  | Weighted median | 15 | 1.05 | 0.89-1.24 | 0.538 |  |
|  |  |  | MR-PRESSO test | 15 | 1.14 | 1.00-1.29 | 0.069 |  |
|  |  |  | MR-Egger | 15 | / | / | 0.731* |  |
| Pasteurellaceae | 5.52% | 56.29 |  |  |  |  |  |  |
|  |  |  | Inverse-variance weighted (fixed) | 19 | 1.04 | 0.92-1.19 | 0.515 | 0.197 |
|  |  |  | Weighted median | 19 | 1.01 | 0.84-1.23 | 0.882 |  |
|  |  |  | MR-PRESSO test | 19 | 1.04 | 0.90-1.21 | 0.571 |  |
|  |  |  | MR-Egger | 19 | / | / | 0.155* |  |
| Peptococcaceae | 2.80% | 52.72 |  |  |  |  |  |  |
|  |  |  | Inverse-variance weighted (fixed) | 10 | 0.82 | 0.68-0.98 | 0.033 | 0.694 |
|  |  |  | Weighted median | 10 | 0.83 | 0.64-1.08 | 0.172 |  |
|  |  |  | MR-PRESSO test | 10 | 0.82 | 0.70-0.96 | 0.033 |  |
|  |  |  | MR-Egger | 10 | / | / | 0.724* |  |
| Peptostreptococcaceae | 3.05% | 36.03 |  |  |  |  |  |  |
|  |  |  | Inverse-variance weighted (fixed) | 16 | 1.06 | 0.88-1.27 | 0.542 | 0.471 |
|  |  |  | Weighted median | 16 | 1.20 | 0.92-1.56 | 0.177 |  |
|  |  |  | MR-PRESSO test | 16 | 1.06 | 0.88-1.27 | 0.548 |  |
|  |  |  | MR-Egger | 16 | / | / | 0.124* |  |
| Porphyromonadaceae | 1.46% | 22.60 |  |  |  |  |  |  |
|  |  |  | Inverse-variance weighted (fixed) | 12 | 1.05 | 0.82-1.34 | 0.714 | 0.128 |
|  |  |  | Weighted median | 12 | 1.16 | 0.81-1.67 | 0.420 |  |
|  |  |  | MR-PRESSO test | 12 | 1.05 | 0.78-1.42 | 0.769 |  |
|  |  |  | MR-Egger | 12 | / | / | 0.075* |  |
| Prevotellaceae | 3.07% | 32.27 |  |  |  |  |  |  |
|  |  |  | Inverse-variance weighted (random) | 18 | 0.99 | 0.78-1.26 | 0.930 | 0.014 |
|  |  |  | Weighted median | 18 | 0.98 | 0.76-1.27 | 0.894 |  |
|  |  |  | MR-PRESSO test | 18 | 0.99 | 0.78-1.26 | 0.931 |  |
|  |  |  | MR-Egger | 18 | / | / | 0.804* |  |
| Rhodospirillaceae | 3.91% | 46.76 |  |  |  |  |  |  |
|  |  |  | Inverse-variance weighted (fixed) | 16 | 1.12 | 0.96-1.30 | 0.147 | 0.065 |
|  |  |  | Weighted median | 16 | 1.10 | 0.88-1.38 | 0.385 |  |
|  |  |  | MR-PRESSO test | 16 | 1.12 | 0.92-1.35 | 0.270 |  |
|  |  |  | MR-Egger | 16 | / | / | 0.188* |  |
| Rikenellaceae | 3.07% | 25.20 |  |  |  |  |  |  |
|  |  |  | Inverse-variance weighted (fixed) | 23 | 1.13 | 0.95-1.34 | 0.182 | 0.231 |
|  |  |  | Weighted median | 23 | 1.10 | 0.85-1.41 | 0.470 |  |
|  |  |  | MR-PRESSO test | 23 | 1.13 | 0.93-1.36 | 0.237 |  |
|  |  |  | MR-Egger | 23 | / | / | 0.811* |  |
| Ruminococcaceae | 1.90% | 29.58 |  |  |  |  |  |  |
|  |  |  | Inverse-variance weighted (fixed) | 12 | 1.05 | 0.84-1.31 | 0.646 | 0.552 |
|  |  |  | Weighted median | 12 | 0.94 | 0.69-1.28 | 0.709 |  |
|  |  |  | MR-PRESSO test | 12 | 1.05 | 0.86-1.30 | 0.636 |  |
|  |  |  | MR-Egger | 12 | / | / | 0.630* |  |
| Streptococcaceae | 2.70% | 26.71 |  |  |  |  |  |  |
|  |  |  | Inverse-variance weighted (fixed) | 19 | 0.99 | 0.82-1.19 | 0.929 | 0.089 |
|  |  |  | Weighted median | 19 | 1.09 | 0.83-1.43 | 0.534 |  |
|  |  |  | MR-PRESSO test | 19 | 0.99 | 0.79-1.24 | 0.942 |  |
|  |  |  | MR-Egger | 19 | / | / | 0.815* |  |
| Veillonellaceae | 3.79% | 34.40 |  |  |  |  |  |  |
|  |  |  | Inverse-variance weighted (fixed) | 21 | 0.94 | 0.81-1.11 | 0.476 | 0.894 |
|  |  |  | Weighted median | 21 | 1.00 | 0.81-1.23 | 0.992 |  |
|  |  |  | MR-PRESSO test | 21 | 0.94 | 0.83-1.07 | 0.380 |  |
|  |  |  | MR-Egger | 21 | / | / | 0.847* |  |
| Verrucomicrobiaceae | 2.63% | 38.04 |  |  |  |  |  |  |
|  |  |  | Inverse-variance weighted (fixed) | 13 | 1.00 | 0.83-1.21 | 0.975 | 0.158 |
|  |  |  | Weighted median | 13 | 0.96 | 0.72-1.28 | 0.755 |  |
|  |  |  | MR-PRESSO test | 13 | 1.00 | 0.80-1.25 | 0.980 |  |
|  |  |  | MR-Egger | 13 | / | / | 0.026* |  |
| Victivallaceae | 9.17% | 123.35 |  |  |  |  |  |  |
|  |  |  | Inverse-variance weighted (random) | 15 | 1.03 | 0.89-1.20 | 0.669 | 0.010 |
|  |  |  | Weighted median | 15 | 1.06 | 0.92-1.23 | 0.422 |  |
|  |  |  | MR-PRESSO test | 15 | 1.03 | 0.89-1.20 | 0.676 |  |
|  |  |  | Outlier corrected (MR-PRESSO) | 14 | 1.07 | 0.95-1.22 | 0.291 |  |
|  |  |  | MR-Egger | 15 | / | / | 0.114* |  |
| Genus |  |  |  |  |  |  |  |  |
| Clostridiuminnocuum | 5.90% | 104.48 |  |  |  |  |  |  |
|  |  |  | Inverse-variance weighted (fixed) | 11 | 0.94 | 0.83-1.07 | 0.322 | 0.358 |
|  |  |  | Weighted median | 11 | 0.94 | 0.79-1.11 | 0.465 |  |
|  |  |  | MR-PRESSO test | 11 | 0.94 | 0.82-1.07 | 0.368 |  |
|  |  |  | MR-Egger | 11 | / | / | 0.719* |  |
| Eubacteriumbrachy | 5.35% | 103.67 |  |  |  |  |  |  |
|  |  |  | Inverse-variance weighted (fixed) | 10 | 1.01 | 0.88-1.15 | 0.917 | 0.658 |
|  |  |  | Weighted median | 10 | 0.94 | 0.79-1.13 | 0.520 |  |
|  |  |  | MR-PRESSO test | 10 | 1.01 | 0.90-1.13 | 0.907 |  |
|  |  |  | MR-Egger | 10 | / | / | 0.915* |  |
| Eubacteriumcoprostanoligenes | 1.95% | 24.32 |  |  |  |  |  |  |
|  |  |  | Inverse-variance weighted (fixed) | 15 | 0.92 | 0.74-1.15 | 0.470 | 0.770 |
|  |  |  | Weighted median | 15 | 0.88 | 0.66-1.19 | 0.415 |  |
|  |  |  | MR-PRESSO test | 15 | 0.92 | 0.77-1.11 | 0.404 |  |
|  |  |  | MR-Egger | 15 | / | / | 0.200* |  |
| Eubacteriumeligens | 1.85% | 31.49 |  |  |  |  |  |  |
|  |  |  | Inverse-variance weighted (fixed) | 11 | 1.26 | 1.00-1.58 | 0.047 | 0.816 |
|  |  |  | Weighted median | 11 | 1.25 | 0.92-1.69 | 0.151 |  |
|  |  |  | MR-PRESSO test | 11 | 1.26 | 1.06-1.50 | 0.028 |  |
|  |  |  | MR-Egger | 11 | / | / | 0.305* |  |
| Eubacteriumfissicatena | 5.01% | 107.36 |  |  |  |  |  |  |
|  |  |  | Inverse-variance weighted (fixed) | 9 | 1.06 | 0.93-1.21 | 0.397 | 0.243 |
|  |  |  | Weighted median | 9 | 1.10 | 0.91-1.33 | 0.341 |  |
|  |  |  | MR-PRESSO test | 9 | 1.06 | 0.91-1.24 | 0.478 |  |
|  |  |  | MR-Egger | 9 | / | / | 0.890* |  |
| Eubacteriumhallii | 2.82% | 33.25 |  |  |  |  |  |  |
|  |  |  | Inverse-variance weighted (fixed) | 16 | 0.92 | 0.75-1.11 | 0.382 | 0.070 |
|  |  |  | Weighted median | 16 | 1.02 | 0.76-1.37 | 0.882 |  |
|  |  |  | MR-PRESSO test | 16 | 0.92 | 0.72-1.17 | 0.498 |  |
|  |  |  | MR-Egger | 16 | / | / | 0.003* |  |
| Eubacteriumnodatum | 7.15% | 128.36 |  |  |  |  |  |  |
|  |  |  | Inverse-variance weighted (fixed) | 11 | 0.98 | 0.88-1.10 | 0.763 | 0.951 |
|  |  |  | Weighted median | 11 | 1.01 | 0.88-1.16 | 0.867 |  |
|  |  |  | MR-PRESSO test | 11 | 0.98 | 0.92-1.05 | 0.641 |  |
|  |  |  | MR-Egger | 11 | / | / | 0.614* |  |
| Eubacteriumoxidoreducens | 2.20% | 82.61 |  |  |  |  |  |  |
|  |  |  | Inverse-variance weighted (fixed) | 5 | 0.83 | 0.68-1.03 | 0.092 | 0.802 |
|  |  |  | Weighted median | 5 | 0.79 | 0.61-1.02 | 0.072 |  |
|  |  |  | MR-PRESSO test | 5 | 0.83 | 0.73-0.95 | 0.058 |  |
|  |  |  | MR-Egger | 5 | / | / | 0.954* |  |
| Eubacteriumrectale | 1.78% | 25.52 |  |  |  |  |  |  |
|  |  |  | Inverse-variance weighted (fixed) | 13 | 1.15 | 0.92-1.44 | 0.215 | 0.445 |
|  |  |  | Weighted median | 13 | 1.15 | 0.85-1.57 | 0.369 |  |
|  |  |  | MR-PRESSO test | 13 | 1.15 | 0.92-1.44 | 0.239 |  |
|  |  |  | MR-Egger | 13 | / | / | 0.309* |  |
| Eubacteriumruminantium | 5.88% | 60.26 |  |  |  |  |  |  |
|  |  |  | Inverse-variance weighted (fixed) | 19 | 1.14 | 1.00-1.30 | 0.043 | 0.301 |
|  |  |  | Weighted median | 19 | 1.26 | 1.04-1.51 | 0.016 |  |
|  |  |  | MR-PRESSO test | 19 | 1.14 | 0.99-1.31 | 0.075 |  |
|  |  |  | MR-Egger | 19 | / | / | 0.647* |  |
| Eubacteriumventriosum | 2.60% | 28.78 |  |  |  |  |  |  |
|  |  |  | Inverse-variance weighted (fixed) | 17 | 0.68 | 0.56-0.83 | 0.000 | 0.056 |
|  |  |  | Weighted median | 17 | 0.66 | 0.49-0.89 | 0.006 |  |
|  |  |  | MR-PRESSO test | 17 | 0.68 | 0.53-0.87 | 0.008 |  |
|  |  |  | MR-Egger | 17 | / | / | 0.972* |  |
| Eubacteriumxylanophilum | 2.11% | 32.91 |  |  |  |  |  |  |
|  |  |  | Inverse-variance weighted (fixed) | 12 | 1.07 | 0.86-1.32 | 0.541 | 0.241 |
|  |  |  | Weighted median | 12 | 1.15 | 0.85-1.54 | 0.365 |  |
|  |  |  | MR-PRESSO test | 12 | 1.07 | 0.84-1.36 | 0.597 |  |
|  |  |  | MR-Egger | 12 | / | / | 0.006* |  |
| Ruminococcusgauvreauii | 2.15% | 31.00 |  |  |  |  |  |  |
|  |  |  | Inverse-variance weighted (fixed) | 13 | 0.87 | 0.71-1.07 | 0.197 | 0.117 |
|  |  |  | Weighted median | 13 | 0.88 | 0.66-1.18 | 0.395 |  |
|  |  |  | MR-PRESSO test | 13 | 0.87 | 0.68-1.12 | 0.312 |  |
|  |  |  | MR-Egger | 13 | / | / | 0.721* |  |
| Ruminococcusgnavus | 4.08% | 64.94 |  |  |  |  |  |  |
|  |  |  | Inverse-variance weighted (fixed) | 12 | 0.96 | 0.83-1.11 | 0.569 | 0.586 |
|  |  |  | Weighted median | 12 | 0.94 | 0.77-1.15 | 0.529 |  |
|  |  |  | MR-PRESSO test | 12 | 0.96 | 0.83-1.10 | 0.551 |  |
|  |  |  | MR-Egger | 12 | / | / | 0.265* |  |
| Ruminococcustorques | 2.08% | 26.00 |  |  |  |  |  |  |
|  |  |  | Inverse-variance weighted (fixed) | 15 | 1.03 | 0.83-1.26 | 0.811 | 0.294 |
|  |  |  | Weighted median | 15 | 0.92 | 0.69-1.23 | 0.581 |  |
|  |  |  | MR-PRESSO test | 15 | 1.03 | 0.82-1.28 | 0.828 |  |
|  |  |  | MR-Egger | 15 | / | / | 0.033* |  |
| Actinomyces | 2.66% | 62.61 |  |  |  |  |  |  |
|  |  |  | Inverse-variance weighted (fixed) | 8 | 0.95 | 0.79-1.14 | 0.599 | 0.502 |
|  |  |  | Weighted median | 8 | 1.02 | 0.80-1.31 | 0.857 |  |
|  |  |  | MR-PRESSO test | 8 | 0.95 | 0.80-1.13 | 0.597 |  |
|  |  |  | MR-Egger | 8 | / | / | 0.074* |  |
| Adlercreutzia | 3.11% | 49.06 |  |  |  |  |  |  |
|  |  |  | Inverse-variance weighted (fixed) | 12 | 1.05 | 0.88-1.25 | 0.577 | 0.886 |
|  |  |  | Weighted median | 12 | 0.99 | 0.79-1.24 | 0.921 |  |
|  |  |  | MR-PRESSO test | 12 | 1.05 | 0.93-1.19 | 0.458 |  |
|  |  |  | MR-Egger | 12 | / | / | 0.935* |  |
| Akkermansia | 2.63% | 38.06 |  |  |  |  |  |  |
|  |  |  | Inverse-variance weighted (fixed) | 13 | 1.00 | 0.83-1.21 | 0.977 | 0.158 |
|  |  |  | Weighted median | 13 | 0.95 | 0.72-1.27 | 0.754 |  |
|  |  |  | MR-PRESSO test | 13 | 1.00 | 0.80-1.25 | 0.981 |  |
|  |  |  | MR-Egger | 13 | / | / | 0.026* |  |
| Alistipes | 1.80% | 22.37 |  |  |  |  |  |  |
|  |  |  | Inverse-variance weighted (fixed) | 15 | 1.11 | 0.89-1.39 | 0.365 | 0.388 |
|  |  |  | Weighted median | 15 | 1.17 | 0.86-1.60 | 0.307 |  |
|  |  |  | MR-PRESSO test | 15 | 1.11 | 0.88-1.40 | 0.394 |  |
|  |  |  | MR-Egger | 15 | / | / | 0.024* |  |
| Allisonella | 6.06% | 131.44 |  |  |  |  |  |  |
|  |  |  | Inverse-variance weighted (fixed) | 9 | 1.05 | 0.93-1.18 | 0.460 | 0.167 |
|  |  |  | Weighted median | 9 | 0.96 | 0.82-1.13 | 0.657 |  |
|  |  |  | MR-PRESSO test | 9 | 1.05 | 0.90-1.21 | 0.558 |  |
|  |  |  | MR-Egger | 9 | / | / | 0.003* |  |
| Alloprevotella | 4.85% | 133.35 |  |  |  |  |  |  |
|  |  |  | Inverse-variance weighted (fixed) | 7 | 1.05 | 0.92-1.20 | 0.461 | 0.250 |
|  |  |  | Weighted median | 7 | 1.11 | 0.92-1.34 | 0.286 |  |
|  |  |  | MR-PRESSO test | 7 | 1.05 | 0.90-1.23 | 0.543 |  |
|  |  |  | MR-Egger | 7 | / | / | 0.406* |  |
| Anaerofilum | 5.45% | 88.11 |  |  |  |  |  |  |
|  |  |  | Inverse-variance weighted (fixed) | 12 | 0.94 | 0.83-1.08 | 0.393 | 0.179 |
|  |  |  | Weighted median | 12 | 0.99 | 0.82-1.19 | 0.938 |  |
|  |  |  | MR-PRESSO test | 12 | 0.94 | 0.81-1.10 | 0.481 |  |
|  |  |  | MR-Egger | 12 | / | / | 0.420* |  |
| Anaerostipes | 2.19% | 27.40 |  |  |  |  |  |  |
|  |  |  | Inverse-variance weighted (fixed) | 15 | 0.96 | 0.77-1.19 | 0.687 | 0.793 |
|  |  |  | Weighted median | 15 | 1.00 | 0.74-1.33 | 0.981 |  |
|  |  |  | MR-PRESSO test | 15 | 0.96 | 0.80-1.14 | 0.634 |  |
|  |  |  | MR-Egger | 15 | / | / | 0.117* |  |
| Anaerotruncus | 2.14% | 25.10 |  |  |  |  |  |  |
|  |  |  | Inverse-variance weighted (fixed) | 15 | 0.88 | 0.72-1.09 | 0.256 | 0.292 |
|  |  |  | Weighted median | 15 | 0.90 | 0.65-1.23 | 0.500 |  |
|  |  |  | MR-PRESSO test | 15 | 0.88 | 0.70-1.11 | 0.311 |  |
|  |  |  | MR-Egger | 15 | / | / | 0.739* |  |
| Bacteroides | 1.47% | 27.38 |  |  |  |  |  |  |
|  |  |  | Inverse-variance weighted (fixed) | 10 | 0.96 | 0.73-1.27 | 0.790 | 0.555 |
|  |  |  | Weighted median | 10 | 0.98 | 0.68-1.42 | 0.927 |  |
|  |  |  | MR-PRESSO test | 10 | 0.96 | 0.75-1.24 | 0.782 |  |
|  |  |  | MR-Egger | 10 | / | / | 0.762* |  |
| Barnesiella | 2.63% | 31.01 |  |  |  |  |  |  |
|  |  |  | Inverse-variance weighted (fixed) | 16 | 1.21 | 0.99-1.47 | 0.056 | 0.805 |
|  |  |  | Weighted median | 16 | 1.14 | 0.87-1.50 | 0.328 |  |
|  |  |  | MR-PRESSO test | 16 | 1.21 | 1.03-1.42 | 0.035 |  |
|  |  |  | MR-Egger | 16 | / | / | 0.705* |  |
| Bifidobacterium | 4.32% | 37.58 |  |  |  |  |  |  |
|  |  |  | Inverse-variance weighted (random) | 22 | 0.82 | 0.66-1.01 | 0.068 | 0.008 |
|  |  |  | Weighted median | 22 | 0.93 | 0.73-1.18 | 0.564 |  |
|  |  |  | MR-PRESSO test | 22 | 0.82 | 0.66-1.01 | 0.082 |  |
|  |  |  | Outlier corrected (MR-PRESSO) | 21 | 0.87 | 0.74-1.04 | 0.142 |  |
|  |  |  | MR-Egger | 22 | / | / | 0.161* |  |
| Bilophila | 2.82% | 31.30 |  |  |  |  |  |  |
|  |  |  | Inverse-variance weighted (fixed) | 17 | 1.08 | 0.90-1.29 | 0.423 | 0.989 |
|  |  |  | Weighted median | 17 | 1.10 | 0.88-1.38 | 0.405 |  |
|  |  |  | MR-PRESSO test | 17 | 1.08 | 0.97-1.20 | 0.205 |  |
|  |  |  | MR-Egger | 17 | / | / | 0.214* |  |
| Blautia | 2.01% | 28.89 |  |  |  |  |  |  |
|  |  |  | Inverse-variance weighted (fixed) | 13 | 1.02 | 0.82-1.27 | 0.824 | 0.749 |
|  |  |  | Weighted median | 13 | 1.14 | 0.85-1.52 | 0.389 |  |
|  |  |  | MR-PRESSO test | 13 | 1.02 | 0.85-1.23 | 0.795 |  |
|  |  |  | MR-Egger | 13 | / | / | 0.331* |  |
| Butyricicoccus | 1.95% | 40.56 |  |  |  |  |  |  |
|  |  |  | Inverse-variance weighted (fixed) | 9 | 1.31 | 1.04-1.64 | 0.020 | 0.075 |
|  |  |  | Weighted median | 9 | 1.04 | 0.75-1.45 | 0.807 |  |
|  |  |  | MR-PRESSO test | 9 | 1.31 | 0.97-1.76 | 0.119 |  |
|  |  |  | MR-Egger | 9 | / | / | 0.109* |  |
| Butyricimonas | 3.92% | 41.48 |  |  |  |  |  |  |
|  |  |  | Inverse-variance weighted (fixed) | 18 | 1.05 | 0.90-1.22 | 0.565 | 0.701 |
|  |  |  | Weighted median | 18 | 1.14 | 0.92-1.40 | 0.230 |  |
|  |  |  | MR-PRESSO test | 18 | 1.05 | 0.91-1.20 | 0.527 |  |
|  |  |  | MR-Egger | 18 | / | / | 0.679* |  |
| Butyrivibrio | 10.11% | 128.73 |  |  |  |  |  |  |
|  |  |  | Inverse-variance weighted (fixed) | 16 | 1.01 | 0.92-1.11 | 0.805 | 0.450 |
|  |  |  | Weighted median | 16 | 1.02 | 0.89-1.16 | 0.775 |  |
|  |  |  | MR-PRESSO test | 16 | 1.01 | 0.92-1.11 | 0.808 |  |
|  |  |  | MR-Egger | 16 | / | / | 0.199* |  |
| CandidatusSoleaferrea | 5.83% | 70.84 |  |  |  |  |  |  |
|  |  |  | Inverse-variance weighted (fixed) | 16 | 0.99 | 0.87-1.12 | 0.822 | 0.394 |
|  |  |  | Weighted median | 16 | 0.94 | 0.79-1.12 | 0.487 |  |
|  |  |  | MR-PRESSO test | 16 | 0.99 | 0.86-1.12 | 0.830 |  |
|  |  |  | MR-Egger | 16 | / | / | 0.334* |  |
| Catenibacterium | 3.28% | 124.43 |  |  |  |  |  |  |
|  |  |  | Inverse-variance weighted (fixed) | 5 | 0.87 | 0.73-1.02 | 0.089 | 0.582 |
|  |  |  | Weighted median | 5 | 0.83 | 0.67-1.03 | 0.097 |  |
|  |  |  | MR-PRESSO test | 5 | 0.87 | 0.75-1.00 | 0.115 |  |
|  |  |  | MR-Egger | 5 | / | / | 0.445* |  |
| ChristensenellaceaeR.7 | 1.50% | 25.33 |  |  |  |  |  |  |
|  |  |  | Inverse-variance weighted (fixed) | 11 | 1.01 | 0.78-1.30 | 0.958 | 0.106 |
|  |  |  | Weighted median | 11 | 1.13 | 0.78-1.63 | 0.522 |  |
|  |  |  | MR-PRESSO test | 11 | 1.01 | 0.73-1.39 | 0.967 |  |
|  |  |  | MR-Egger | 11 | / | / | 0.809* |  |
| Clostridiumsensustricto1 | 1.86% | 38.54 |  |  |  |  |  |  |
|  |  |  | Inverse-variance weighted (fixed) | 9 | 0.79 | 0.64-0.99 | 0.039 | 0.151 |
|  |  |  | Weighted median | 9 | 0.78 | 0.58-1.06 | 0.110 |  |
|  |  |  | MR-PRESSO test | 9 | 0.79 | 0.60-1.04 | 0.130 |  |
|  |  |  | MR-Egger | 9 | / | / | 0.838* |  |
| Collinsella | 1.93% | 27.67 |  |  |  |  |  |  |
|  |  |  | Inverse-variance weighted (fixed) | 13 | 0.95 | 0.77-1.18 | 0.654 | 0.994 |
|  |  |  | Weighted median | 13 | 0.94 | 0.71-1.23 | 0.641 |  |
|  |  |  | MR-PRESSO test | 13 | 0.95 | 0.85-1.06 | 0.403 |  |
|  |  |  | MR-Egger | 13 | / | / | 0.862* |  |
| Coprobacter | 5.29% | 73.18 |  |  |  |  |  |  |
|  |  |  | Inverse-variance weighted (fixed) | 14 | 0.93 | 0.80-1.06 | 0.279 | 0.172 |
|  |  |  | Weighted median | 14 | 0.87 | 0.71-1.06 | 0.172 |  |
|  |  |  | MR-PRESSO test | 14 | 0.93 | 0.79-1.09 | 0.369 |  |
|  |  |  | MR-Egger | 14 | / | / | 0.067* |  |
| Coprococcus1 | 2.38% | 31.91 |  |  |  |  |  |  |
|  |  |  | Inverse-variance weighted (fixed) | 14 | 1.07 | 0.88-1.32 | 0.495 | 0.285 |
|  |  |  | Weighted median | 14 | 0.96 | 0.72-1.29 | 0.784 |  |
|  |  |  | MR-PRESSO test | 14 | 1.07 | 0.86-1.34 | 0.541 |  |
|  |  |  | MR-Egger | 14 | / | / | 0.427* |  |
| Coprococcus2 | 2.36% | 36.88 |  |  |  |  |  |  |
|  |  |  | Inverse-variance weighted (fixed) | 12 | 1.32 | 1.08-1.62 | 0.007 | 0.554 |
|  |  |  | Weighted median | 12 | 1.36 | 1.02-1.81 | 0.034 |  |
|  |  |  | MR-PRESSO test | 12 | 1.32 | 1.09-1.60 | 0.015 |  |
|  |  |  | MR-Egger | 12 | / | / | 0.491* |  |
| Coprococcus3 | 1.62% | 27.50 |  |  |  |  |  |  |
|  |  |  | Inverse-variance weighted (random) | 11 | 1.00 | 0.70-1.42 | 0.989 | 0.026 |
|  |  |  | Weighted median | 11 | 0.86 | 0.59-1.24 | 0.410 |  |
|  |  |  | MR-PRESSO test | 11 | 1.00 | 0.70-1.42 | 0.989 |  |
|  |  |  | MR-Egger | 11 | / | / | 0.169* |  |
| DefluviitaleaceaeUCG011 | 3.18% | 54.72 |  |  |  |  |  |  |
|  |  |  | Inverse-variance weighted (fixed) | 11 | 1.01 | 0.85-1.21 | 0.891 | 0.886 |
|  |  |  | Weighted median | 11 | 1.00 | 0.81-1.25 | 0.975 |  |
|  |  |  | MR-PRESSO test | 11 | 1.01 | 0.89-1.15 | 0.851 |  |
|  |  |  | MR-Egger | 11 | / | / | 0.456* |  |
| Desulfovibrio | 3.18% | 50.13 |  |  |  |  |  |  |
|  |  |  | Inverse-variance weighted (fixed) | 12 | 0.99 | 0.83-1.18 | 0.922 | 0.941 |
|  |  |  | Weighted median | 12 | 0.94 | 0.75-1.17 | 0.567 |  |
|  |  |  | MR-PRESSO test | 12 | 0.99 | 0.88-1.11 | 0.885 |  |
|  |  |  | MR-Egger | 12 | / | / | 0.417* |  |
| Dialister | 2.16% | 33.65 |  |  |  |  |  |  |
|  |  |  | Inverse-variance weighted (fixed) | 12 | 1.07 | 0.87-1.31 | 0.537 | 0.522 |
|  |  |  | Weighted median | 12 | 1.00 | 0.76-1.32 | 0.975 |  |
|  |  |  | MR-PRESSO test | 12 | 1.07 | 0.88-1.30 | 0.533 |  |
|  |  |  | MR-Egger | 12 | / | / | 0.752* |  |
| Dorea | 1.82% | 26.17 |  |  |  |  |  |  |
|  |  |  | Inverse-variance weighted (fixed) | 13 | 1.04 | 0.83-1.31 | 0.714 | 0.770 |
|  |  |  | Weighted median | 13 | 1.09 | 0.82-1.47 | 0.549 |  |
|  |  |  | MR-PRESSO test | 13 | 1.04 | 0.87-1.26 | 0.666 |  |
|  |  |  | MR-Egger | 13 | / | / | 0.561* |  |
| Eggerthella | 4.13% | 79.01 |  |  |  |  |  |  |
|  |  |  | Inverse-variance weighted (fixed) | 10 | 1.03 | 0.88-1.20 | 0.739 | 0.680 |
|  |  |  | Weighted median | 10 | 1.04 | 0.85-1.27 | 0.714 |  |
|  |  |  | MR-PRESSO test | 10 | 1.03 | 0.90-1.17 | 0.706 |  |
|  |  |  | MR-Egger | 10 | / | / | 0.926* |  |
| Eisenbergiella | 4.52% | 72.37 |  |  |  |  |  |  |
|  |  |  | Inverse-variance weighted (fixed) | 12 | 1.00 | 0.87-1.15 | 0.973 | 0.159 |
|  |  |  | Weighted median | 12 | 1.05 | 0.86-1.27 | 0.637 |  |
|  |  |  | MR-PRESSO test | 12 | 1.00 | 0.85-1.18 | 0.978 |  |
|  |  |  | MR-Egger | 12 | / | / | 0.531* |  |
| Enterorhabdus | 3.22% | 67.98 |  |  |  |  |  |  |
|  |  |  | Inverse-variance weighted (fixed) | 9 | 0.87 | 0.74-1.03 | 0.111 | 0.809 |
|  |  |  | Weighted median | 9 | 0.83 | 0.67-1.04 | 0.109 |  |
|  |  |  | MR-PRESSO test | 9 | 0.87 | 0.77-0.99 | 0.066 |  |
|  |  |  | MR-Egger | 9 | / | / | 0.080* |  |
| Erysipelatoclostridium | 4.07% | 45.68 |  |  |  |  |  |  |
|  |  |  | Inverse-variance weighted (fixed) | 17 | 1.05 | 0.90-1.23 | 0.514 | 0.284 |
|  |  |  | Weighted median | 17 | 1.12 | 0.90-1.39 | 0.319 |  |
|  |  |  | MR-PRESSO test | 17 | 1.05 | 0.89-1.24 | 0.554 |  |
|  |  |  | MR-Egger | 17 | / | / | 0.010* |  |
| ErysipelotrichaceaeUCG003 | 3.28% | 34.53 |  |  |  |  |  |  |
|  |  |  | Inverse-variance weighted (fixed) | 18 | 1.01 | 0.85-1.20 | 0.945 | 0.438 |
|  |  |  | Weighted median | 18 | 1.08 | 0.84-1.39 | 0.545 |  |
|  |  |  | MR-PRESSO test | 18 | 1.01 | 0.84-1.20 | 0.947 |  |
|  |  |  | MR-Egger | 18 | / | / | 0.692* |  |
| Escherichia.Shigella | 2.97% | 37.39 |  |  |  |  |  |  |
|  |  |  | Inverse-variance weighted (fixed) | 15 | 1.01 | 0.84-1.22 | 0.934 | 0.393 |
|  |  |  | Weighted median | 15 | 1.03 | 0.80-1.32 | 0.841 |  |
|  |  |  | MR-PRESSO test | 15 | 1.01 | 0.83-1.22 | 0.937 |  |
|  |  |  | MR-Egger | 15 | / | / | 0.899* |  |
| Faecalibacterium | 2.60% | 37.59 |  |  |  |  |  |  |
|  |  |  | Inverse-variance weighted (fixed) | 13 | 1.11 | 0.91-1.36 | 0.302 | 0.733 |
|  |  |  | Weighted median | 13 | 0.98 | 0.75-1.29 | 0.894 |  |
|  |  |  | MR-PRESSO test | 13 | 1.11 | 0.94-1.32 | 0.248 |  |
|  |  |  | MR-Egger | 13 | / | / | 0.971* |  |
| FamilyXIIIAD3011 (ID: 11293) | 2.24% | 30.01 |  |  |  |  |  |  |
|  |  |  | Inverse-variance weighted (fixed) | 14 | 0.98 | 0.80-1.21 | 0.851 | 0.229 |
|  |  |  | Weighted median | 14 | 0.90 | 0.67-1.20 | 0.478 |  |
|  |  |  | MR-PRESSO test | 14 | 0.98 | 0.78-1.24 | 0.869 |  |
|  |  |  | MR-Egger | 14 | / | / | 0.960* |  |
| FamilyXIIIUCG001 (ID: 11294) | 1.94% | 36.25 |  |  |  |  |  |  |
|  |  |  | Inverse-variance weighted (random) | 10 | 1.19 | 0.86-1.64 | 0.291 | 0.026 |
|  |  |  | Weighted median | 10 | 1.18 | 0.85-1.65 | 0.320 |  |
|  |  |  | MR-PRESSO test | 10 | 1.19 | 0.86-1.64 | 0.318 |  |
|  |  |  | MR-Egger | 10 | / | / | 0.851* |  |
| Flavonifractor | 2.19% | 40.97 |  |  |  |  |  |  |
|  |  |  | Inverse-variance weighted (fixed) | 10 | 0.93 | 0.75-1.15 | 0.490 | 0.167 |
|  |  |  | Weighted median | 10 | 1.03 | 0.77-1.37 | 0.845 |  |
|  |  |  | MR-PRESSO test | 10 | 0.93 | 0.72-1.20 | 0.578 |  |
|  |  |  | MR-Egger | 10 | / | / | 0.364* |  |
| Fusicatenibacter | 2.60% | 24.45 |  |  |  |  |  |  |
|  |  |  | Inverse-variance weighted (fixed) | 20 | 0.89 | 0.74-1.07 | 0.221 | 0.456 |
|  |  |  | Weighted median | 20 | 0.99 | 0.75-1.31 | 0.965 |  |
|  |  |  | MR-PRESSO test | 20 | 0.89 | 0.74-1.07 | 0.236 |  |
|  |  |  | MR-Egger | 20 | / | / | 0.662* |  |
| Gordonibacter | 9.26% | 124.67 |  |  |  |  |  |  |
|  |  |  | Inverse-variance weighted (random) | 15 | 0.97 | 0.85-1.11 | 0.622 | 0.031 |
|  |  |  | Weighted median | 15 | 0.91 | 0.78-1.05 | 0.198 |  |
|  |  |  | MR-PRESSO test | 15 | 0.97 | 0.85-1.11 | 0.630 |  |
|  |  |  | MR-Egger | 15 | / | / | 0.327* |  |
| Haemophilus | 4.10% | 55.89 |  |  |  |  |  |  |
|  |  |  | Inverse-variance weighted (fixed) | 14 | 1.17 | 1.01-1.36 | 0.033 | 0.518 |
|  |  |  | Weighted median | 14 | 1.22 | 0.99-1.50 | 0.059 |  |
|  |  |  | MR-PRESSO test | 14 | 1.17 | 1.02-1.35 | 0.046 |  |
|  |  |  | MR-Egger | 14 | / | / | 0.092* |  |
| Holdemanella | 4.79% | 65.87 |  |  |  |  |  |  |
|  |  |  | Inverse-variance weighted (fixed) | 14 | 0.84 | 0.73-0.96 | 0.014 | 0.928 |
|  |  |  | Weighted median | 14 | 0.85 | 0.71-1.02 | 0.085 |  |
|  |  |  | MR-PRESSO test | 14 | 0.84 | 0.76-0.92 | 0.004 |  |
|  |  |  | MR-Egger | 14 | / | / | 0.587* |  |
| Holdemania | 4.84% | 51.77 |  |  |  |  |  |  |
|  |  |  | Inverse-variance weighted (fixed) | 18 | 0.98 | 0.85-1.12 | 0.746 | 0.892 |
|  |  |  | Weighted median | 18 | 0.99 | 0.82-1.20 | 0.950 |  |
|  |  |  | MR-PRESSO test | 18 | 0.98 | 0.88-1.09 | 0.681 |  |
|  |  |  | MR-Egger | 18 | / | / | 0.261* |  |
| Howardella | 6.28% | 111.64 |  |  |  |  |  |  |
|  |  |  | Inverse-variance weighted (fixed) | 11 | 0.97 | 0.86-1.10 | 0.666 | 0.630 |
|  |  |  | Weighted median | 11 | 0.95 | 0.82-1.11 | 0.544 |  |
|  |  |  | MR-PRESSO test | 11 | 0.97 | 0.87-1.08 | 0.639 |  |
|  |  |  | MR-Egger | 11 | / | / | 0.796* |  |
| Hungatella | 2.50% | 93.98 |  |  |  |  |  |  |
|  |  |  | Inverse-variance weighted (fixed) | 5 | 1.03 | 0.85-1.25 | 0.771 | 0.137 |
|  |  |  | Weighted median | 5 | 0.90 | 0.69-1.17 | 0.418 |  |
|  |  |  | MR-PRESSO test | 5 | 1.03 | 0.80-1.33 | 0.836 |  |
|  |  |  | MR-Egger | 5 | / | / | 0.536* |  |
| Intestinibacter | 2.71% | 33.98 |  |  |  |  |  |  |
|  |  |  | Inverse-variance weighted (fixed) | 15 | 0.99 | 0.83-1.19 | 0.951 | 0.718 |
|  |  |  | Weighted median | 15 | 1.07 | 0.84-1.36 | 0.578 |  |
|  |  |  | MR-PRESSO test | 15 | 0.99 | 0.85-1.16 | 0.944 |  |
|  |  |  | MR-Egger | 15 | / | / | 0.546* |  |
| Intestinimonas | 4.41% | 42.28 |  |  |  |  |  |  |
|  |  |  | Inverse-variance weighted (fixed) | 20 | 0.95 | 0.81-1.10 | 0.469 | 0.933 |
|  |  |  | Weighted median | 20 | 0.93 | 0.76-1.14 | 0.486 |  |
|  |  |  | MR-PRESSO test | 20 | 0.95 | 0.85-1.06 | 0.347 |  |
|  |  |  | MR-Egger | 20 | / | / | 0.975* |  |
| Lachnoclostridium | 1.86% | 23.18 |  |  |  |  |  |  |
|  |  |  | Inverse-variance weighted (fixed) | 15 | 0.92 | 0.73-1.15 | 0.459 | 0.204 |
|  |  |  | Weighted median | 15 | 0.85 | 0.61-1.18 | 0.320 |  |
|  |  |  | MR-PRESSO test | 15 | 0.92 | 0.71-1.19 | 0.525 |  |
|  |  |  | MR-Egger | 15 | / | / | 0.028* |  |
| Lachnospira | 0.95% | 25.08 |  |  |  |  |  |  |
|  |  |  | Inverse-variance weighted (random) | 7 | 0.77 | 0.48-1.24 | 0.280 | 0.028 |
|  |  |  | Weighted median | 7 | 0.90 | 0.55-1.47 | 0.671 |  |
|  |  |  | MR-PRESSO test | 7 | 0.77 | 0.48-1.24 | 0.321 |  |
|  |  |  | MR-Egger | 7 | / | / | 0.345* |  |
| LachnospiraceaeFCS020 | 3.11% | 34.54 |  |  |  |  |  |  |
|  |  |  | Inverse-variance weighted (fixed) | 17 | 1.21 | 1.02-1.43 | 0.030 | 0.231 |
|  |  |  | Weighted median | 17 | 1.18 | 0.92-1.51 | 0.200 |  |
|  |  |  | MR-PRESSO test | 17 | 1.21 | 1.00-1.46 | 0.069 |  |
|  |  |  | MR-Egger | 17 | / | / | 0.130* |  |
| LachnospiraceaeNC2004 | 3.49% | 66.37 |  |  |  |  |  |  |
|  |  |  | Inverse-variance weighted (fixed) | 10 | 1.11 | 0.94-1.30 | 0.224 | 0.167 |
|  |  |  | Weighted median | 10 | 1.21 | 0.96-1.53 | 0.109 |  |
|  |  |  | MR-PRESSO test | 10 | 1.11 | 0.91-1.34 | 0.336 |  |
|  |  |  | MR-Egger | 10 | / | / | 0.289* |  |
| LachnospiraceaeND3007 | 0.57% | 26.17 |  |  |  |  |  |  |
|  |  |  | Inverse-variance weighted (fixed) | 4 | 1.44 | 0.92-2.27 | 0.114 | 0.354 |
|  |  |  | Weighted median | 4 | 1.44 | 0.82-2.52 | 0.205 |  |
|  |  |  | MR-PRESSO test | 4 | 1.44 | 0.90-2.31 | 0.226 |  |
|  |  |  | MR-Egger | 4 | / | / | 0.558* |  |
| LachnospiraceaeNK4A136 | 2.75% | 32.32 |  |  |  |  |  |  |
|  |  |  | Inverse-variance weighted (fixed) | 16 | 1.06 | 0.88-1.28 | 0.527 | 0.828 |
|  |  |  | Weighted median | 16 | 1.06 | 0.81-1.38 | 0.670 |  |
|  |  |  | MR-PRESSO test | 16 | 1.06 | 0.91-1.23 | 0.447 |  |
|  |  |  | MR-Egger | 16 | / | / | 0.585* |  |
| LachnospiraceaeUCG001 | 3.38% | 42.75 |  |  |  |  |  |  |
|  |  |  | Inverse-variance weighted (fixed) | 15 | 1.01 | 0.85-1.19 | 0.939 | 0.785 |
|  |  |  | Weighted median | 15 | 1.03 | 0.82-1.30 | 0.793 |  |
|  |  |  | MR-PRESSO test | 15 | 1.01 | 0.88-1.16 | 0.928 |  |
|  |  |  | MR-Egger | 15 | / | / | 0.887* |  |
| LachnospiraceaeUCG004 | 2.28% | 28.45 |  |  |  |  |  |  |
|  |  |  | Inverse-variance weighted (fixed) | 15 | 0.93 | 0.76-1.13 | 0.473 | 0.702 |
|  |  |  | Weighted median | 15 | 0.95 | 0.73-1.25 | 0.728 |  |
|  |  |  | MR-PRESSO test | 15 | 0.93 | 0.78-1.11 | 0.427 |  |
|  |  |  | MR-Egger | 15 | / | / | 0.741* |  |
| LachnospiraceaeUCG008 | 4.84% | 66.62 |  |  |  |  |  |  |
|  |  |  | Inverse-variance weighted (fixed) | 14 | 1.13 | 0.98-1.30 | 0.100 | 0.861 |
|  |  |  | Weighted median | 14 | 1.12 | 0.92-1.35 | 0.254 |  |
|  |  |  | MR-PRESSO test | 14 | 1.13 | 1.01-1.25 | 0.052 |  |
|  |  |  | MR-Egger | 14 | / | / | 0.942* |  |
| LachnospiraceaeUCG010 | 2.52% | 36.40 |  |  |  |  |  |  |
|  |  |  | Inverse-variance weighted (fixed) | 13 | 1.21 | 1.00-1.48 | 0.052 | 0.079 |
|  |  |  | Weighted median | 13 | 1.23 | 0.93-1.63 | 0.149 |  |
|  |  |  | MR-PRESSO test | 13 | 1.21 | 0.95-1.56 | 0.152 |  |
|  |  |  | MR-Egger | 13 | / | / | 0.716* |  |
| Lactobacillus | 4.16% | 66.24 |  |  |  |  |  |  |
|  |  |  | Inverse-variance weighted (fixed) | 12 | 0.87 | 0.75-1.02 | 0.083 | 0.371 |
|  |  |  | Weighted median | 12 | 0.83 | 0.67-1.02 | 0.076 |  |
|  |  |  | MR-PRESSO test | 12 | 0.87 | 0.75-1.02 | 0.124 |  |
|  |  |  | MR-Egger | 12 | / | / | 0.944* |  |
| Lactococcus | 5.95% | 105.41 |  |  |  |  |  |  |
|  |  |  | Inverse-variance weighted (fixed) | 11 | 1.03 | 0.91-1.16 | 0.642 | 0.241 |
|  |  |  | Weighted median | 11 | 1.00 | 0.85-1.17 | 0.985 |  |
|  |  |  | MR-PRESSO test | 11 | 1.03 | 0.90-1.18 | 0.689 |  |
|  |  |  | MR-Egger | 11 | / | / | 0.946* |  |
| Marvinbryantia | 2.43% | 35.13 |  |  |  |  |  |  |
|  |  |  | Inverse-variance weighted (fixed) | 13 | 1.14 | 0.94-1.39 | 0.184 | 0.516 |
|  |  |  | Weighted median | 13 | 1.09 | 0.84-1.42 | 0.506 |  |
|  |  |  | MR-PRESSO test | 13 | 1.14 | 0.95-1.38 | 0.194 |  |
|  |  |  | MR-Egger | 13 | / | / | 0.782* |  |
| Methanobrevibacter | 4.61% | 110.62 |  |  |  |  |  |  |
|  |  |  | Inverse-variance weighted (random) | 8 | 0.99 | 0.81-1.21 | 0.925 | 0.034 |
|  |  |  | Weighted median | 8 | 0.96 | 0.79-1.17 | 0.702 |  |
|  |  |  | MR-PRESSO test | 8 | 0.99 | 0.81-1.21 | 0.927 |  |
|  |  |  | Outlier corrected (MR-PRESSO) | 7 | 1.07 | 0.91-1.26 | 0.461 |  |
|  |  |  | MR-Egger | 8 | / | / | 0.673* |  |
| Odoribacter | 1.45% | 30.01 |  |  |  |  |  |  |
|  |  |  | Inverse-variance weighted (fixed) | 9 | 1.03 | 0.80-1.32 | 0.844 | 0.621 |
|  |  |  | Weighted median | 9 | 0.97 | 0.70-1.34 | 0.844 |  |
|  |  |  | MR-PRESSO test | 9 | 1.03 | 0.82-1.28 | 0.829 |  |
|  |  |  | MR-Egger | 9 | / | / | 0.966* |  |
| Olsenella | 6.41% | 114.06 |  |  |  |  |  |  |
|  |  |  | Inverse-variance weighted (fixed) | 11 | 0.93 | 0.82-1.05 | 0.222 | 0.360 |
|  |  |  | Weighted median | 11 | 0.93 | 0.79-1.09 | 0.371 |  |
|  |  |  | MR-PRESSO test | 11 | 0.93 | 0.82-1.05 | 0.271 |  |
|  |  |  | MR-Egger | 11 | / | / | 0.475* |  |
| Oscillibacter | 4.29% | 51.30 |  |  |  |  |  |  |
|  |  |  | Inverse-variance weighted (fixed) | 16 | 0.84 | 0.72-0.97 | 0.018 | 0.564 |
|  |  |  | Weighted median | 16 | 0.85 | 0.69-1.04 | 0.113 |  |
|  |  |  | MR-PRESSO test | 16 | 0.84 | 0.73-0.96 | 0.025 |  |
|  |  |  | MR-Egger | 16 | / | / | 0.772* |  |
| Oscillospira | 2.17% | 40.72 |  |  |  |  |  |  |
|  |  |  | Inverse-variance weighted (fixed) | 10 | 0.89 | 0.73-1.10 | 0.282 | 0.181 |
|  |  |  | Weighted median | 10 | 0.91 | 0.68-1.22 | 0.524 |  |
|  |  |  | MR-PRESSO test | 10 | 0.89 | 0.70-1.14 | 0.387 |  |
|  |  |  | MR-Egger | 10 | / | / | 0.316* |  |
| Oxalobacter | 6.51% | 106.27 |  |  |  |  |  |  |
|  |  |  | Inverse-variance weighted (fixed) | 12 | 1.14 | 1.01-1.28 | 0.037 | 0.392 |
|  |  |  | Weighted median | 12 | 1.04 | 0.87-1.23 | 0.673 |  |
|  |  |  | MR-PRESSO test | 12 | 1.14 | 1.00-1.29 | 0.067 |  |
|  |  |  | MR-Egger | 12 | / | / | 0.795* |  |
| Parabacteroides | 1.47% | 27.38 |  |  |  |  |  |  |
|  |  |  | Inverse-variance weighted (fixed) | 10 | 0.99 | 0.77-1.27 | 0.952 | 0.389 |
|  |  |  | Weighted median | 10 | 1.09 | 0.77-1.54 | 0.637 |  |
|  |  |  | MR-PRESSO test | 10 | 0.99 | 0.77-1.28 | 0.955 |  |
|  |  |  | MR-Egger | 10 | / | / | 0.689* |  |
| Paraprevotella | 4.40% | 64.84 |  |  |  |  |  |  |
|  |  |  | Inverse-variance weighted (fixed) | 13 | 0.97 | 0.84-1.13 | 0.713 | 0.088 |
|  |  |  | Weighted median | 13 | 0.92 | 0.74-1.14 | 0.433 |  |
|  |  |  | MR-PRESSO test | 13 | 0.97 | 0.81-1.17 | 0.775 |  |
|  |  |  | MR-Egger | 13 | / | / | 0.791* |  |
| Parasutterella | 3.69% | 41.32 |  |  |  |  |  |  |
|  |  |  | Inverse-variance weighted (fixed) | 17 | 0.95 | 0.81-1.11 | 0.496 | 0.382 |
|  |  |  | Weighted median | 17 | 0.88 | 0.70-1.11 | 0.291 |  |
|  |  |  | MR-PRESSO test | 17 | 0.95 | 0.81-1.11 | 0.519 |  |
|  |  |  | MR-Egger | 17 | / | / | 0.630* |  |
| Peptococcus | 6.62% | 81.14 |  |  |  |  |  |  |
|  |  |  | Inverse-variance weighted (fixed) | 16 | 1.02 | 0.90-1.15 | 0.779 | 0.885 |
|  |  |  | Weighted median | 16 | 0.96 | 0.82-1.12 | 0.613 |  |
|  |  |  | MR-PRESSO test | 16 | 1.02 | 0.93-1.12 | 0.719 |  |
|  |  |  | MR-Egger | 16 | / | / | 0.089* |  |
| Phascolarctobacterium | 2.71% | 39.22 |  |  |  |  |  |  |
|  |  |  | Inverse-variance weighted (fixed) | 13 | 1.03 | 0.86-1.24 | 0.752 | 0.099 |
|  |  |  | Weighted median | 13 | 0.92 | 0.70-1.20 | 0.536 |  |
|  |  |  | MR-PRESSO test | 13 | 1.03 | 0.82-1.29 | 0.804 |  |
|  |  |  | MR-Egger | 13 | / | / | 0.374* |  |
| Prevotella7 | 7.34% | 121.04 |  |  |  |  |  |  |
|  |  |  | Inverse-variance weighted (fixed) | 12 | 0.94 | 0.84-1.06 | 0.300 | 0.151 |
|  |  |  | Weighted median | 12 | 0.97 | 0.82-1.15 | 0.739 |  |
|  |  |  | MR-PRESSO test | 12 | 0.94 | 0.82-1.08 | 0.405 |  |
|  | 5.00% | 48.25 | MR-Egger | 12 | / | / | 0.966* |  |
| Prevotella9 |  |  |  |  |  |  |  |  |
|  |  |  | Inverse-variance weighted (fixed) | 20 | 0.97 | 0.84-1.12 | 0.699 | 0.384 |
|  |  |  | Weighted median | 20 | 0.96 | 0.78-1.19 | 0.715 |  |
|  |  |  | MR-PRESSO test | 20 | 0.97 | 0.84-1.12 | 0.712 |  |
|  |  |  | MR-Egger | 20 | / | / | 0.181* |  |
| RikenellaceaeRC9 | 9.72% | 131.46 |  |  |  |  |  |  |
|  |  |  | Inverse-variance weighted (random) | 15 | 0.87 | 0.75-1.01 | 0.073 | 0.007 |
|  |  |  | Weighted median | 15 | 0.86 | 0.73-1.01 | 0.067 |  |
|  |  |  | MR-PRESSO test | 15 | 0.87 | 0.75-1.01 | 0.095 |  |
|  |  |  | MR-Egger | 15 | / | / | 0.026* |  |
| Romboutsia | 2.87% | 36.08 |  |  |  |  |  |  |
|  |  |  | Inverse-variance weighted (fixed) | 15 | 0.94 | 0.78-1.13 | 0.486 | 0.257 |
|  |  |  | Weighted median | 15 | 1.00 | 0.78-1.29 | 0.986 |  |
|  |  |  | MR-PRESSO test | 15 | 0.94 | 0.76-1.15 | 0.537 |  |
|  |  |  | MR-Egger | 15 | / | / | 0.886* |  |
| Roseburia | 2.57% | 26.88 |  |  |  |  |  |  |
|  |  |  | Inverse-variance weighted (fixed) | 18 | 1.09 | 0.89-1.32 | 0.408 | 0.773 |
|  |  |  | Weighted median | 18 | 1.02 | 0.78-1.34 | 0.891 |  |
|  |  |  | MR-PRESSO test | 18 | 1.09 | 0.92-1.28 | 0.347 |  |
|  |  |  | MR-Egger | 18 | / | / | 0.043* |  |
| Ruminiclostridium5 | 2.06% | 25.73 |  |  |  |  |  |  |
|  |  |  | Inverse-variance weighted (fixed) | 15 | 0.88 | 0.71-1.09 | 0.244 | 0.162 |
|  |  |  | Weighted median | 15 | 1.02 | 0.75-1.37 | 0.919 |  |
|  |  |  | MR-PRESSO test | 15 | 0.88 | 0.69-1.13 | 0.335 |  |
|  |  |  | MR-Egger | 15 | / | / | 0.868* |  |
| Ruminiclostridium6 | 3.28% | 36.50 |  |  |  |  |  |  |
|  |  |  | Inverse-variance weighted (fixed) | 17 | 0.89 | 0.75-1.05 | 0.174 | 0.178 |
|  |  |  | Weighted median | 17 | 0.86 | 0.66-1.12 | 0.256 |  |
|  |  |  | MR-PRESSO test | 17 | 0.89 | 0.73-1.08 | 0.253 |  |
|  |  |  | MR-Egger | 17 | / | / | 0.425* |  |
| Ruminiclostridium9 | 2.06% | 25.75 |  |  |  |  |  |  |
|  |  |  | Inverse-variance weighted (fixed) | 15 | 1.18 | 0.96-1.46 | 0.122 | 0.650 |
|  |  |  | Weighted median | 15 | 1.09 | 0.82-1.45 | 0.544 |  |
|  |  |  | MR-PRESSO test | 15 | 1.18 | 0.98-1.43 | 0.109 |  |
|  |  |  | MR-Egger | 15 | / | / | 0.603* |  |
| RuminococcaceaeNK4A214 | 2.76% | 28.93 |  |  |  |  |  |  |
|  |  |  | Inverse-variance weighted (fixed) | 18 | 0.96 | 0.80-1.16 | 0.681 | 0.117 |
|  |  |  | Weighted median | 18 | 0.92 | 0.70-1.21 | 0.570 |  |
|  |  |  | MR-PRESSO test | 18 | 0.96 | 0.77-1.20 | 0.734 |  |
|  |  |  | MR-Egger | 18 | / | / | 0.486* |  |
| RuminococcaceaeUCG002 | 3.86% | 28.25 |  |  |  |  |  |  |
|  |  |  | Inverse-variance weighted (fixed) | 26 | 0.90 | 0.77-1.05 | 0.176 | 0.705 |
|  |  |  | Weighted median | 26 | 0.91 | 0.73-1.13 | 0.377 |  |
|  |  |  | MR-PRESSO test | 26 | 0.90 | 0.78-1.04 | 0.150 |  |
|  |  |  | MR-Egger | 26 | / | / | 0.733* |  |
| RuminococcaceaeUCG003 | 2.28% | 30.60 |  |  |  |  |  |  |
|  |  |  | Inverse-variance weighted (fixed) | 14 | 0.85 | 0.70-1.04 | 0.107 | 0.373 |
|  |  |  | Weighted median | 14 | 0.92 | 0.70-1.22 | 0.580 |  |
|  |  |  | MR-PRESSO test | 14 | 0.85 | 0.69-1.04 | 0.144 |  |
|  |  |  | MR-Egger | 14 | / | / | 0.569* |  |
| RuminococcaceaeUCG004 | 2.57% | 43.94 |  |  |  |  |  |  |
|  |  |  | Inverse-variance weighted (random) | 11 | 1.14 | 0.89-1.48 | 0.301 | 0.038 |
|  |  |  | Weighted median | 11 | 1.12 | 0.86-1.46 | 0.408 |  |
|  |  |  | MR-PRESSO test | 11 | 1.14 | 0.89-1.48 | 0.325 |  |
|  |  |  | MR-Egger | 11 | / | / | 0.308* |  |
| RuminococcaceaeUCG005 | 2.73% | 30.20 |  |  |  |  |  |  |
|  |  |  | Inverse-variance weighted (random) | 17 | 1.01 | 0.80-1.28 | 0.912 | 0.047 |
|  |  |  | Weighted median | 17 | 1.02 | 0.79-1.33 | 0.857 |  |
|  |  |  | MR-PRESSO test | 17 | 1.01 | 0.80-1.28 | 0.914 |  |
|  |  |  | MR-Egger | 17 | / | / | 0.325* |  |
| RuminococcaceaeUCG009 | 4.30% | 59.42 |  |  |  |  |  |  |
|  |  |  | Inverse-variance weighted (fixed) | 13 | 1.00 | 0.86-1.16 | 0.987 | 0.826 |
|  |  |  | Weighted median | 13 | 0.96 | 0.78-1.18 | 0.689 |  |
|  |  |  | MR-PRESSO test | 13 | 1.00 | 0.88-1.13 | 0.984 |  |
|  |  |  | MR-Egger | 13 | / | / | 0.566* |  |
| RuminococcaceaeUCG010 | 1.51% | 35.03 |  |  |  |  |  |  |
|  |  |  | Inverse-variance weighted (fixed) | 8 | 0.90 | 0.70-1.16 | 0.410 | 0.421 |
|  |  |  | Weighted median | 8 | 0.95 | 0.67-1.33 | 0.748 |  |
|  |  |  | MR-PRESSO test | 8 | 0.90 | 0.70-1.16 | 0.440 |  |
|  |  |  | MR-Egger | 8 | / | / | 0.766* |  |
| RuminococcaceaeUCG011 | 4.90% | 118.09 |  |  |  |  |  |  |
|  |  |  | Inverse-variance weighted (fixed) | 8 | 0.92 | 0.81-1.06 | 0.250 | 0.528 |
|  |  |  | Weighted median | 8 | 0.87 | 0.74-1.03 | 0.101 |  |
|  |  |  | MR-PRESSO test | 8 | 0.92 | 0.82-1.05 | 0.258 |  |
|  |  |  | MR-Egger | 8 | / | / | 0.846* |  |
| RuminococcaceaeUCG013 | 2.09% | 26.01 |  |  |  |  |  |  |
|  |  |  | Inverse-variance weighted (fixed) | 15 | 1.22 | 1.00-1.50 | 0.051 | 0.251 |
|  |  |  | Weighted median | 15 | 1.15 | 0.86-1.55 | 0.346 |  |
|  |  |  | MR-PRESSO test | 15 | 1.22 | 0.98-1.53 | 0.099 |  |
|  |  |  | MR-Egger | 15 | / | / | 0.472* |  |
| RuminococcaceaeUCG014 | 2.88% | 31.95 |  |  |  |  |  |  |
|  |  |  | Inverse-variance weighted (fixed) | 17 | 1.16 | 0.97-1.39 | 0.107 | 0.064 |
|  |  |  | Weighted median | 17 | 1.07 | 0.81-1.42 | 0.621 |  |
|  |  |  | MR-PRESSO test | 17 | 1.16 | 0.92-1.46 | 0.219 |  |
|  |  |  | MR-Egger | 17 | / | / | 0.416* |  |
| Ruminococcus1 | 2.14% | 28.60 |  |  |  |  |  |  |
|  |  |  | Inverse-variance weighted (fixed) | 14 | 1.13 | 0.91-1.40 | 0.274 | 0.066 |
|  |  |  | Weighted median | 14 | 1.23 | 0.89-1.71 | 0.203 |  |
|  |  |  | MR-PRESSO test | 14 | 1.13 | 0.86-1.48 | 0.409 |  |
|  |  |  | MR-Egger | 14 | / | / | 0.829* |  |
| Ruminococcus2 | 2.76% | 34.63 |  |  |  |  |  |  |
|  |  |  | Inverse-variance weighted (fixed) | 15 | 0.81 | 0.67-0.99 | 0.039 | 0.837 |
|  |  |  | Weighted median | 15 | 0.82 | 0.63-1.08 | 0.155 |  |
|  |  |  | MR-PRESSO test | 15 | 0.81 | 0.69-0.95 | 0.021 |  |
|  |  |  | MR-Egger | 15 | / | / | 0.157* |  |
| Sellimonas | 9.32% | 144.84 |  |  |  |  |  |  |
|  |  |  | Inverse-variance weighted (fixed) | 13 | 1.00 | 0.90-1.10 | 0.941 | 0.836 |
|  |  |  | Weighted median | 13 | 0.96 | 0.84-1.10 | 0.582 |  |
|  |  |  | MR-PRESSO test | 13 | 1.00 | 0.92-1.08 | 0.927 |  |
|  |  |  | MR-Egger | 13 | / | / | 0.466* |  |
| Senegalimassilia | 2.58% | 60.59 |  |  |  |  |  |  |
|  |  |  | Inverse-variance weighted (fixed) | 8 | 0.98 | 0.82-1.18 | 0.868 | 0.819 |
|  |  |  | Weighted median | 8 | 1.03 | 0.81-1.29 | 0.830 |  |
|  |  |  | MR-PRESSO test | 8 | 0.98 | 0.86-1.12 | 0.824 |  |
|  |  |  | MR-Egger | 8 | / | / | 0.225* |  |
| Slackia | 2.81% | 66.15 |  |  |  |  |  |  |
|  |  |  | Inverse-variance weighted (fixed) | 8 | 0.84 | 0.70-1.00 | 0.051 | 0.447 |
|  |  |  | Weighted median | 8 | 0.77 | 0.61-0.98 | 0.033 |  |
|  |  |  | MR-PRESSO test | 8 | 0.84 | 0.70-1.00 | 0.089 |  |
|  |  |  | MR-Egger | 8 | / | / | 0.908* |  |
| Streptococcus | 2.68% | 26.56 |  |  |  |  |  |  |
|  |  |  | Inverse-variance weighted (fixed) | 19 | 1.06 | 0.88-1.27 | 0.556 | 0.164 |
|  |  |  | Weighted median | 19 | 1.15 | 0.88-1.49 | 0.307 |  |
|  |  |  | MR-PRESSO test | 19 | 1.06 | 0.85-1.31 | 0.615 |  |
|  |  |  | MR-Egger | 19 | / | / | 0.738* |  |
| Subdoligranulum | 1.86% | 24.81 |  |  |  |  |  |  |
|  |  |  | Inverse-variance weighted (fixed) | 14 | 1.15 | 0.93-1.43 | 0.191 | 0.054 |
|  |  |  | Weighted median | 14 | 1.08 | 0.79-1.49 | 0.620 |  |
|  |  |  | MR-PRESSO test | 14 | 1.15 | 0.87-1.53 | 0.334 |  |
|  |  |  | MR-Egger | 14 | / | / | 0.264* |  |
| Sutterella | 1.97% | 30.70 |  |  |  |  |  |  |
|  |  |  | Inverse-variance weighted (fixed) | 12 | 0.89 | 0.72-1.09 | 0.257 | 0.524 |
|  |  |  | Weighted median | 12 | 0.81 | 0.62-1.06 | 0.126 |  |
|  |  |  | MR-PRESSO test | 12 | 0.89 | 0.72-1.08 | 0.261 |  |
|  |  |  | MR-Egger | 12 | / | / | 0.873* |  |
| Terrisporobacter | 2.14% | 66.94 |  |  |  |  |  |  |
|  |  |  | Inverse-variance weighted (fixed) | 6 | 0.92 | 0.75-1.13 | 0.447 | 0.248 |
|  |  |  | Weighted median | 6 | 0.87 | 0.66-1.14 | 0.310 |  |
|  |  |  | MR-PRESSO test | 6 | 0.92 | 0.73-1.17 | 0.539 |  |
|  |  |  | MR-Egger | 6 | / | / | 0.566* |  |
| Turicibacter | 3.35% | 45.43 |  |  |  |  |  |  |
|  |  |  | Inverse-variance weighted (fixed) | 14 | 1.16 | 0.99-1.37 | 0.066 | 0.962 |
|  |  |  | Weighted median | 14 | 1.20 | 0.97-1.48 | 0.090 |  |
|  |  |  | MR-PRESSO test | 14 | 1.16 | 1.05-1.29 | 0.015 |  |
|  |  |  | MR-Egger | 14 | / | / | 0.840* |  |
| Tyzzerella3 | 6.44% | 90.12 |  |  |  |  |  |  |
|  |  |  | Inverse-variance weighted (fixed) | 14 | 1.06 | 0.94-1.20 | 0.317 | 0.900 |
|  |  |  | Weighted median | 14 | 1.11 | 0.94-1.30 | 0.209 |  |
|  |  |  | MR-PRESSO test | 14 | 1.06 | 0.97-1.17 | 0.198 |  |
|  |  |  | MR-Egger | 14 | / | / | 0.594* |  |
| Veillonella | 2.77% | 47.44 |  |  |  |  |  |  |
|  |  |  | Inverse-variance weighted (fixed) | 11 | 0.91 | 0.75-1.09 | 0.296 | 0.628 |
|  |  |  | Weighted median | 11 | 0.98 | 0.76-1.27 | 0.894 |  |
|  |  |  | MR-PRESSO test | 11 | 0.91 | 0.77-1.07 | 0.270 |  |
|  |  |  | MR-Egger | 11 | / | / | 0.640* |  |
| Victivallis | 7.29% | 130.96 |  |  |  |  |  |  |
|  |  |  | Inverse-variance weighted (fixed) | 11 | 1.08 | 0.96-1.21 | 0.185 | 0.374 |
|  |  |  | Weighted median | 11 | 1.08 | 0.92-1.27 | 0.336 |  |
|  |  |  | MR-PRESSO test | 11 | 1.08 | 0.96-1.21 | 0.231 |  |
|  |  |  | MR-Egger | 11 | / | / | 0.540* |  |

Abbreviations: CI, confidence interval; MR, Mendelian randomization; MR-PRESSO test, MR Pleiotropy RESidual Sum and Outlier test; OR, odds ratio; SNP, single nucleotide polymorphism. **P*-value of the intercept from MR-Egger regression analysis.

**Supplementary Table 4** Effect estimates of the associations between 196 bacterial traits and risk of Crohn’s disease in MR analyses

| Gut microbiota | R^2^ | F statistic | Methods | N.SNP | OR | 95% CI | *P*-value | Qrs |
| --- | --- | --- | --- | --- | --- | --- | --- | --- |
| Phylum |  |  |  |  |  |  |  |  |
| Actinobacteria | 2.80% | 26.43 |  |  |  |  |  |  |
|  |  |  | Inverse-variance weighted (fixed) | 20 | 0.92 | 0.72-1.18 | 0.400 | 0.064 |
|  |  |  | Weighted median | 20 | 0.87 | 0.65-1.16 | 0.333 |  |
|  |  |  | MR-PRESSO test | 20 | 0.96 | 0.85-1.09 | 0.529 |  |
|  |  |  | MR-Egger | 20 | / | / | 0.619* |  |
| Bacteroidetes | 2.21% | 29.61 |  |  |  |  |  |  |
|  |  |  | Inverse-variance weighted (fixed) | 14 | 1.00 | 0.80-1.26 | 0.985 | 0.576 |
|  |  |  | Weighted median | 14 | 0.94 | 0.70-1.27 | 0.693 |  |
|  |  |  | MR-PRESSO test | 14 | 1.00 | 0.81-1.24 | 0.985 |  |
|  |  |  | MR-Egger | 14 | / | / | 0.069* |  |
| Cyanobacteria | 3.53% | 67.16 |  |  |  |  |  |  |
|  |  |  | Inverse-variance weighted (fixed) | 10 | 0.90 | 0.76-1.08 | 0.256 | 0.839 |
|  |  |  | Weighted median | 10 | 0.88 | 0.70-1.11 | 0.281 |  |
|  |  |  | MR-PRESSO test | 10 | 0.90 | 0.79-1.03 | 0.249 |  |
|  |  |  | MR-Egger | 10 | / | / | 0.879* |  |
| Euryarchaeota | 7.39% | 112.50 |  |  |  |  |  |  |
|  |  |  | Inverse-variance weighted (fixed) | 13 | 1.02 | 0.90-1.15 | 0.762 | 0.351 |
|  |  |  | Weighted median | 13 | 1.07 | 0.90-1.28 | 0.411 |  |
|  |  |  | MR-PRESSO test | 13 | 1.02 | 0.90-1.15 | 0.778 |  |
|  |  |  | MR-Egger | 13 | / | / | 0.304* |  |
| Firmicutes | 2.49% | 24.65 |  |  |  |  |  |  |
|  |  |  | Inverse-variance weighted (fixed) | 19 | 0.91 | 0.72-1.15 | 0.412 | 0.186 |
|  |  |  | Weighted median | 19 | 0.96 | 0.71-1.28 | 0.773 |  |
|  |  |  | MR-PRESSO test | 19 | 0.91 | 0.72-1.15 | 0.422 |  |
|  |  |  | MR-Egger | 19 | / | / | 0.096* |  |
| Lentisphaerae | 5.25% | 92.39 |  |  |  |  |  |  |
|  |  |  | Inverse-variance weighted (fixed) | 11 | 0.94 | 0.81-1.08 | 0.392 | 0.741 |
|  |  |  | Weighted median | 11 | 0.89 | 0.74-1.07 | 0.223 |  |
|  |  |  | MR-PRESSO test | 11 | 0.94 | 0.83-1.06 | 0.325 |  |
|  |  |  | MR-Egger | 11 | / | / | 0.757* |  |
| Proteobacteria | 1.39% | 28.61 |  |  |  |  |  |  |
|  |  |  | Inverse-variance weighted (random) | 13 | 1.02 | 0.79-1.32 | 0.882 | 0.019 |
|  |  |  | Weighted median | 13 | 1.04 | 0.71-1.52 | 0.857 |  |
|  |  |  | MR-PRESSO test | 13 | 1.02 | 0.71-1.47 | 0.919 |  |
|  |  |  | MR-Egger | 13 | / | / | 0.508* |  |
| Tenericutes | 2.60% | 40.77 |  |  |  |  |  |  |
|  |  |  | Inverse-variance weighted (fixed) | 12 | 1.03 | 0.83-1.27 | 0.791 | 0.355 |
|  |  |  | Weighted median | 12 | 1.07 | 0.80-1.43 | 0.635 |  |
|  |  |  | MR-PRESSO test | 12 | 1.03 | 0.83-1.27 | 0.805 |  |
|  |  |  | MR-Egger | 12 | / | / | 0.519* |  |
| Verrucomicrobia | 2.42% | 37.94 |  |  |  |  |  |  |
|  |  |  | Inverse-variance weighted (fixed) | 12 | 0.95 | 0.73-1.22 | 0.605 | 0.144 |
|  |  |  | Weighted median | 12 | 0.87 | 0.65-1.18 | 0.380 |  |
|  |  |  | MR-PRESSO test | 12 | 0.95 | 0.73-1.22 | 0.676 |  |
|  |  |  | MR-Egger | 12 | / | / | 0.802* |  |
| Class |  |  |  |  |  |  |  |  |
| Actinobacteria | 4.64% | 38.76 |  |  |  |  |  |  |
|  |  |  | Inverse-variance weighted (fixed) | 23 | 0.99 | 0.82-1.19 | 0.887 | 0.077 |
|  |  |  | Weighted median | 23 | 0.92 | 0.73-1.17 | 0.518 |  |
|  |  |  | MR-PRESSO test | 23 | 0.99 | 0.82-1.19 | 0.901 |  |
|  |  |  | MR-Egger | 23 | / | / | 0.885* |  |
| Alphaproteobacteria | 2.30% | 43.24 |  |  |  |  |  |  |
|  |  |  | Inverse-variance weighted (fixed) | 10 | 0.94 | 0.76-1.18 | 0.608 | 0.866 |
|  |  |  | Weighted median | 10 | 0.99 | 0.74-1.34 | 0.962 |  |
|  |  |  | MR-PRESSO test | 10 | 0.94 | 0.80-1.11 | 0.492 |  |
|  |  |  | MR-Egger | 10 | / | / | 0.876* |  |
| Bacilli | 3.50% | 30.17 |  |  |  |  |  |  |
|  |  |  | Inverse-variance weighted (fixed) | 22 | 1.01 | 0.84-1.21 | 0.936 | 0.149 |
|  |  |  | Weighted median | 22 | 1.03 | 0.78-1.37 | 0.829 |  |
|  |  |  | MR-PRESSO test | 22 | 1.01 | 0.81-1.24 | 0.945 |  |
|  |  |  | MR-Egger | 22 | / | / | 0.169* |  |
| Bacteroidia | 2.28% | 28.54 |  |  |  |  |  |  |
|  |  |  | Inverse-variance weighted (fixed) | 15 | 1.02 | 0.82-1.28 | 0.831 | 0.616 |
|  |  |  | Weighted median | 15 | 0.99 | 0.72-1.35 | 0.932 |  |
|  |  |  | MR-PRESSO test | 15 | 1.02 | 0.83-1.26 | 0.820 |  |
|  |  |  | MR-Egger | 15 | / | / | 0.360* |  |
|  | 2.02% | 25.21 |  |  |  |  |  |  |
| Betaproteobacteria |  |  | Inverse-variance weighted (fixed) | 15 | 0.87 | 0.69-1.10 | 0.249 | 0.336 |
|  |  |  | Weighted median | 15 | 1.06 | 0.77-1.48 | 0.709 |  |
|  |  |  | MR-PRESSO test | 15 | 0.87 | 0.68- 1.11 | 0.293 |  |
|  |  |  | MR-Egger | 15 | / | / | 0.257* |  |
| Clostridia | 1.97% | 21.63 |  |  |  |  |  |  |
|  |  |  | Inverse-variance weighted (random) | 17 | 1.07 | 0.77-1.48 | 0.698 | 0.014 |
|  |  |  | Weighted median | 17 | 1.02 | 0.74-1.42 | 0.888 |  |
|  |  |  | MR-PRESSO test | 17 | 1.07 | 0.77-1.48 | 0.703 |  |
|  |  |  | Outlier corrected (MR-PRESSO) | 16 | 1.18 | 0.91-1.54 | 0.234 |  |
|  |  |  | MR-Egger | 17 | / | / | 0.845* |  |
| Coriobacteriia | 2.99% | 26.93 |  |  |  |  |  |  |
|  |  |  | Inverse-variance weighted (fixed) | 21 | 0.99 | 0.81-1.20 | 0.900 | 0.683 |
|  |  |  | Weighted median | 21 | 1.01 | 0.76-1.34 | 0.945 |  |
|  |  |  | MR-PRESSO test | 21 | 0.99 | 0.83-1.18 | 0.891 |  |
|  |  |  | MR-Egger | 21 | / | / | 0.960* |  |
| Deltaproteobacteria | 2.22% | 29.78 |  |  |  |  |  |  |
|  |  |  | Inverse-variance weighted (fixed) | 14 | 1.10 | 0.88-1.36 | 0.398 | 0.276 |
|  |  |  | Weighted median | 14 | 1.18 | 0.88-1.57 | 0.260 |  |
|  |  |  | MR-PRESSO test | 14 | 1.10 | 0.87-1.39 | 0.453 |  |
|  |  |  | MR-Egger | 14 | / | / | 0.917* |  |
| Erysipelotrichia | 1.65% | 23.71 |  |  |  |  |  |  |
|  |  |  | Inverse-variance weighted (fixed) | 13 | 1.04 | 0.80-1.36 | 0.747 | 0.450 |
|  |  |  | Weighted median | 13 | 1.17 | 0.82-1.67 | 0.391 |  |
|  |  |  | MR-PRESSO test | 13 | 1.04 | 0.80-1.36 | 0.752 |  |
|  |  |  | MR-Egger | 13 | / | / | 0.184* |  |
| Gammaproteobacteria | 1.39% | 28.61 |  |  |  |  |  |  |
|  |  |  | Inverse-variance weighted (fixed) | 9 | 0.92 | 0.70-1.22 | 0.570 | 0.565 |
|  |  |  | Weighted median | 9 | 0.87 | 0.61-1.25 | 0.459 |  |
|  |  |  | MR-PRESSO test | 9 | 0.92 | 0.71-1.19 | 0.553 |  |
|  |  |  | MR-Egger | 9 | / | / | 0.750* |  |
| Lentisphaeria | 4.81% | 92.58 |  |  |  |  |  |  |
|  |  |  | Inverse-variance weighted (fixed) | 10 | 0.96 | 0.82-1.12 | 0.590 | 0.732 |
|  |  |  | Weighted median | 10 | 0.93 | 0.77-1.14 | 0.506 |  |
|  |  |  | MR-PRESSO test | 10 | 0.96 | 0.85-1.09 | 0.529 |  |
|  |  |  | MR-Egger | 10 | / | / | 0.619* |  |
| Melainabacteria | 5.25% | 78.13 |  |  |  |  |  |  |
|  |  |  | Inverse-variance weighted (fixed) | 13 | 0.98 | 0.85-1.13 | 0.797 | 0.538 |
|  |  |  | Weighted median | 13 | 0.94 | 0.78-1.12 | 0.476 |  |
|  |  |  | MR-PRESSO test | 13 | 0.98 | 0.86-1.12 | 0.791 |  |
|  |  |  | MR-Egger | 13 | / | / | 0.888* |  |
| Methanobacteria | 7.23% | 119.05 |  |  |  |  |  |  |
|  |  |  | Inverse-variance weighted (fixed) | 12 | 0.97 | 0.86-1.09 | 0.611 | 0.107 |
|  |  |  | Weighted median | 12 | 0.99 | 0.84-1.17 | 0.915 |  |
|  |  |  | MR-PRESSO test | 12 | 0.97 | 0.84-1.12 | 0.691 |  |
|  |  |  | MR-Egger | 12 | / | / | 0.812* |  |
| Mollicutes | 2.60% | 40.77 |  |  |  |  |  |  |
|  |  |  | Inverse-variance weighted (random) | 12 | 1.03 | 0.84-1.26 | 0.791 | 0.355 |
|  |  |  | Weighted median | 12 | 1.07 | 0.80-1.43 | 0.635 |  |
|  |  |  | MR-PRESSO test | 12 | 1.03 | 0.83-1.27 | 0.805 |  |
|  |  |  | MR-Egger | 12 | / | / | 0.519* |  |
| Negativicutes | 1.67% | 23.92 |  |  |  |  |  |  |
|  |  |  | Inverse-variance weighted (fixed) | 13 | 0.88 | 0.69-1.14 | 0.348 | 0.403 |
|  |  |  | Weighted median | 13 | 0.95 | 0.66-1.35 | 0.756 |  |
|  |  |  | MR-PRESSO test | 13 | 0.88 | 0.68-1.15 | 0.377 |  |
|  |  |  | MR-Egger | 13 | / | / | 0.961* |  |
| Verrucomicrobiae | 2.63% | 38.04 |  |  |  |  |  |  |
|  |  |  | Inverse-variance weighted (fixed) | 13 | 0.86 | 0.66-1.11 | 0.236 | 0.096 |
|  |  |  | Weighted median | 13 | 0.89 | 0.66-1.20 | 0.449 |  |
|  |  |  | MR-PRESSO test | 13 | 0.86 | 0.66-1.11 | 0.259 |  |
|  |  |  | MR-Egger | 13 | / | / | 0.931* |  |
| Order |  |  |  |  |  |  |  |  |
| Actinomycetales | 1.75% | 65.35 |  |  |  |  |  |  |
|  |  |  | Inverse-variance weighted (fixed) | 5 | 0.95 | 0.73-1.24 | 0.718 | 0.507 |
|  |  |  | Weighted median | 5 | 0.91 | 0.65-1.27 | 0.580 |  |
|  |  |  | MR-PRESSO test | 5 | 0.95 | 0.75-1.21 | 0.712 |  |
|  |  |  | MR-Egger | 5 | / | / | 0.371* |  |
| Bacillales | 7.14% | 128.04 |  |  |  |  |  |  |
|  |  |  | Inverse-variance weighted (fixed) | 11 | 1.02 | 0.90-1.15 | 0.749 | 0.814 |
|  |  |  | Weighted median | 11 | 1.05 | 0.90-1.23 | 0.519 |  |
|  |  |  | MR-PRESSO test | 11 | 1.02 | 0.93-1.12 | 0.688 |  |
|  |  |  | MR-Egger | 11 | / | / | 0.979* |  |
| Bacteroidales | 2.28% | 28.54 |  |  |  |  |  |  |
|  |  |  | Inverse-variance weighted (fixed) | 15 | 1.02 | 0.82-1.28 | 0.831 | 0.616 |
|  |  |  | Weighted median | 15 | 0.99 | 0.72-1.35 | 0.932 |  |
|  |  |  | MR-PRESSO test | 15 | 1.02 | 0.83-1.26 | 0.820 |  |
|  |  |  | MR-Egger | 15 | / | / | 0.360* |  |
| Bifidobacteriales | 4.44% | 34.00 |  |  |  |  |  |  |
|  |  |  | Inverse-variance weighted (fixed) | 25 | 0.93 | 0.79-1.09 | 0.362 | 0.415 |
|  |  |  | Weighted median | 25 | 0.98 | 0.78-1.24 | 0.866 |  |
|  |  |  | MR-PRESSO test | 25 | 0.93 | 0.79-1.09 | 0.379 |  |
|  |  |  | MR-Egger | 25 | / | / | 0.452* |  |
| Burkholderiales | 1.76% | 25.27 |  |  |  |  |  |  |
|  |  |  | Inverse-variance weighted (fixed) | 13 | 0.95 | 0.74-1.22 | 0.699 | 0.468 |
|  |  |  | Weighted median | 13 | 1.07 | 0.76-1.51 | 0.699 |  |
|  |  |  | MR-PRESSO test | 13 | 0.95 | 0.74-1.22 | 0.702 |  |
|  |  |  | MR-Egger | 13 | / | / | 0.172* |  |
| Clostridiales | 1.97% | 21.66 |  |  |  |  |  |  |
|  |  |  | Inverse-variance weighted (random) | 17 | 1.04 | 0.74-1.46 | 0.810 | 0.009 |
|  |  |  | Weighted median | 17 | 0.99 | 0.70-1.39 | 0.940 |  |
|  |  |  | MR-PRESSO test | 17 | 1.04 | 0.74-1.46 | 0.813 |  |
|  |  |  | Outlier corrected (MR-PRESSO) | 16 | 1.16 | 0.87-1.53 | 0.329 |  |
|  |  |  | MR-Egger | 17 | / | / | 0.702* |  |
| Coriobacteriales | 2.99% | 26.93 |  |  |  |  |  |  |
|  |  |  | Inverse-variance weighted (fixed) | 21 | 0.99 | 0.81-1.20 | 0.900 | 0.683 |
|  |  |  | Weighted median | 21 | 1.01 | 0.76-1.34 | 0.945 |  |
|  |  |  | MR-PRESSO test | 21 | 0.99 | 0.83-1.18 | 0.891 |  |
|  |  |  | MR-Egger | 21 | / | / | 0.960* |  |
| Desulfovibrionales | 2.13% | 30.69 |  |  |  |  |  |  |
|  |  |  | Inverse-variance weighted (rfixed) | 13 | 1.04 | 0.83-1.29 | 0.748 | 0.203 |
|  |  |  | Weighted median | 13 | 1.04 | 0.76-1.42 | 0.820 |  |
|  |  |  | MR-PRESSO test | 13 | 1.04 | 0.81-1.34 | 0.784 |  |
|  |  |  | MR-Egger | 13 | / | / | 0.622* |  |
| Enterobacteriales | 1.84% | 31.17 |  |  |  |  |  |  |
|  |  |  | Inverse-variance weighted (fixed) | 11 | 1.22 | 0.95-1.58 | 0.122 | 0.071 |
|  |  |  | Weighted median | 11 | 1.20 | 0.81-1.77 | 0.373 |  |
|  |  |  | MR-PRESSO test | 11 | 1.22 | 0.88-1.71 | 0.265 |  |
|  |  |  | MR-Egger | 11 | / | / | 0.603* |  |
| Erysipelotrichales | 1.65% | 23.71 |  |  |  |  |  |  |
|  |  |  | Inverse-variance weighted (fixed) | 13 | 1.04 | 0.80-1.36 | 0.747 | 0.450 |
|  |  |  | Weighted median | 13 | 1.17 | 0.82-1.67 | 0.391 |  |
|  |  |  | MR-PRESSO test | 13 | 1.04 | 0.80-1.36 | 0.752 |  |
|  |  |  | MR-Egger | 13 | / | / | 0.184* |  |
| Gastranaerophilales | 4.90% | 78.66 |  |  |  |  |  |  |
|  |  |  | Inverse-variance weighted (fixed) | 12 | 1.00 | 0.86-1.16 | 0.989 | 0.368 |
|  |  |  | Weighted median | 12 | 0.95 | 0.78-1.17 | 0.646 |  |
|  |  |  | MR-PRESSO test | 12 | 1.00 | 0.86-1.16 | 0.989 |  |
|  |  |  | MR-Egger | 12 | / | / | 0.712* |  |
| Lactobacillales | 3.12% | 31.02 |  |  |  |  |  |  |
|  |  |  | Inverse-variance weighted (fixed) | 19 | 1.02 | 0.84-1.24 | 0.823 | 0.118 |
|  |  |  | Weighted median | 19 | 1.01 | 0.74-1.37 | 0.955 |  |
|  |  |  | MR-PRESSO test | 19 | 1.02 | 0.81-1.29 | 0.852 |  |
|  |  |  | MR-Egger | 19 | / | / | 0.247* |  |
| Methanobacteriales | 7.23% | 119.05 |  |  |  |  |  |  |
|  |  |  | Inverse-variance weighted (fixed) | 12 | 0.97 | 0.86-1.09 | 0.611 | 0.107 |
|  |  |  | Weighted median | 12 | 0.99 | 0.84-1.17 | 0.915 |  |
|  |  |  | MR-PRESSO test | 12 | 0.97 | 0.84-1.12 | 0.691 |  |
|  |  |  | MR-Egger | 12 | / | / | 0.812* |  |
| MollicutesRF9 | 3.80% | 42.57 |  |  |  |  |  |  |
|  |  |  | Inverse-variance weighted (fixed) | 17 | 1.03 | 0.87-1.22 | 0.723 | 0.383 |
|  |  |  | Weighted median | 17 | 0.98 | 0.77-1.25 | 0.871 |  |
|  |  |  | MR-PRESSO test | 17 | 1.03 | 0.87-1.23 | 0.736 |  |
|  |  |  | MR-Egger | 17 | / | / | 0.737* |  |
| NB1n | 7.59% | 88.52 |  |  |  |  |  |  |
|  |  |  | Inverse-variance weighted (fixed) | 17 | 1.14 | 1.01-1.28 | 0.036 | 0.216 |
|  |  |  | Weighted median | 17 | 1.19 | 1.01-1.28 | 0.041 |  |
|  |  |  | MR-PRESSO test | 17 | 1.14 | 0.99-1.30 | 0.080 |  |
|  |  |  | MR-Egger | 17 | / | / | 0.740* |  |
| Pasteurellales | 5.52% | 56.29 |  |  |  |  |  |  |
|  |  |  | Inverse-variance weighted (fixed) | 19 | 0.96 | 0.83-1.10 | 0.558 | 0.243 |
|  |  |  | Weighted median | 19 | 1.08 | 0.88-1.33 | 0.452 |  |
|  |  |  | MR-PRESSO test | 19 | 0.96 | 0.82-1.12 | 0.601 |  |
|  |  |  | MR-Egger | 19 | / | / | 0.153* |  |
| Rhodospirillales | 3.39% | 45.92 |  |  |  |  |  |  |
|  |  |  | Inverse-variance weighted (fixed) | 14 | 1.07 | 0.89-1.27 | 0.479 | 0.750 |
|  |  |  | Weighted median | 14 | 0.99 | 0.78-1.25 | 0.935 |  |
|  |  |  | MR-PRESSO test | 14 | 1.07 | 0.92-1.23 | 0.417 |  |
|  |  |  | MR-Egger | 14 | / | / | 0.278* |  |
| Selenomonadales | 1.67% | 23.92 |  |  |  |  |  |  |
|  |  |  | Inverse-variance weighted (fixed) | 13 | 0.88 | 0.69-1.14 | 0.348 | 0.403 |
|  |  |  | Weighted median | 13 | 0.95 | 0.66-1.35 | 0.756 |  |
|  |  |  | MR-PRESSO test | 13 | 0.88 | 0.68-1.15 | 0.377 |  |
|  |  |  | MR-Egger | 13 | / | / | 0.961* |  |
| Verrucomicrobiales | 2.63% | 38.04 |  |  |  |  |  |  |
|  |  |  | Inverse-variance weighted (fixed) | 13 | 0.86 | 0.70-1.05 | 0.139 | 0.095 |
|  |  |  | Weighted median | 13 | 0.89 | 0.66-1.20 | 0.449 |  |
|  |  |  | MR-PRESSO test | 13 | 0.86 | 0.66-1.11 | 0.259 |  |
|  |  |  | MR-Egger | 13 | / | / | 0.931* |  |
| Victivallales | 4.81% | 92.58 |  |  |  |  |  |  |
|  |  |  | Inverse-variance weighted (fixed) | 10 | 0.96 | 0.82-1.12 | 0.590 | 0.732 |
|  |  |  | Weighted median | 10 | 0.93 | 0.77-1.14 | 0.506 |  |
|  |  |  | MR-PRESSO test | 10 | 0.96 | 0.85-1.09 | 0.529 |  |
|  |  |  | MR-Egger | 10 | / | / | 0.619* |  |
| Family |  |  |  |  |  |  |  |  |
| Acidaminococcaceae | 1.50% | 35.00 |  |  |  |  |  |  |
|  |  |  | Inverse-variance weighted (fixed) | 8 | 1.21 | 0.92-1.59 | 0.176 | 0.419 |
|  |  |  | Weighted median | 8 | 1.25 | 0.86-1.81 | 0.250 |  |
|  |  |  | MR-PRESSO test | 8 | 1.21 | 0.92-1.59 | 0.221 |  |
|  |  |  | MR-Egger | 8 | / | / | 0.662* |  |
| Actinomycetaceae | 1.76% | 65.52 |  |  |  |  |  |  |
|  |  |  | Inverse-variance weighted (fixed) | 5 | 0.95 | 0.73-1.24 | 0.717 | 0.506 |
|  |  |  | Weighted median | 5 | 0.91 | 0.65-1.27 | 0.578 |  |
|  |  |  | MR-PRESSO test | 5 | 0.95 | 0.75-1.21 | 0.711 |  |
|  |  |  | MR-Egger | 5 | / | / | 0.370* |  |
| Alcaligenaceae | 2.77% | 26.11 |  |  |  |  |  |  |
|  |  |  | Inverse-variance weighted (fixed) | 20 | 1.05 | 0.86-1.29 | 0.607 | 0.181 |
|  |  |  | Weighted median | 20 | 1.18 | 0.88-1.58 | 0.259 |  |
|  |  |  | MR-PRESSO test | 20 | 1.05 | 0.84-1.32 | 0.655 |  |
|  |  |  | MR-Egger | 20 | / | / | 0.038* |  |
| Bacteroidaceae | 1.23% | 22.84 |  |  |  |  |  |  |
|  |  |  | Inverse-variance weighted (fixed) | 10 | 1.16 | 0.86-1.56 | 0.339 | 0.735 |
|  |  |  | Weighted median | 10 | 1.21 | 0.80-1.82 | 0.361 |  |
|  |  |  | MR-PRESSO test | 10 | 1.16 | 0.91-1.47 | 0.274 |  |
|  |  |  | MR-Egger | 10 | / | / | 0.716* |  |
| BacteroidalesS24.7 | 3.06% | 57.94 |  |  |  |  |  |  |
|  |  |  | Inverse-variance weighted (fixed) | 10 | 1.11 | 0.92-1.34 | 0.257 | 0.452 |
|  |  |  | Weighted median | 10 | 1.14 | 0.89-1.45 | 0.299 |  |
|  |  |  | MR-PRESSO test | 10 | 1.11 | 0.93-1.34 | 0.282 |  |
|  |  |  | MR-Egger | 10 | / | / | 0.492* |  |
| Bifidobacteriaceae | 4.44% | 34.00 |  |  |  |  |  |  |
|  |  |  | Inverse-variance weighted (fixed) | 25 | 0.93 | 0.79-1.09 | 0.362 | 0.415 |
|  |  |  | Weighted median | 25 | 0.98 | 0.78-1.24 | 0.866 |  |
|  |  |  | MR-PRESSO test | 25 | 0.93 | 0.79-1.09 | 0.379 |  |
|  |  |  | MR-Egger | 25 | / | / | 0.452* |  |
| Christensenellaceae | 2.03% | 31.59 |  |  |  |  |  |  |
|  |  |  | Inverse-variance weighted (random) | 12 | 0.87 | 0.69-1.10 | 0.240 | 0.779 |
|  |  |  | Weighted median | 12 | 0.91 | 0.66-1.26 | 0.562 |  |
|  |  |  | MR-PRESSO test | 12 | 0.87 | 0.72-1.05 | 0.176 |  |
|  |  |  | MR-Egger | 12 | / | / | 0.599* |  |
| Clostridiaceae1 | 1.71% | 29.00 |  |  |  |  |  |  |
|  |  |  | Inverse-variance weighted (fixed) | 11 | 0.75 | 0.58-0.97 | 0.026 | 0.081 |
|  |  |  | Weighted median | 11 | 0.70 | 0.49-1.00 | 0.050 |  |
|  |  |  | MR-PRESSO test | 11 | 0.75 | 0.54-1.04 | 0.116 |  |
|  |  |  | MR-Egger | 11 | / | / | 0.647* |  |
| ClostridialesvadinBB60 | 3.73% | 41.81 |  |  |  |  |  |  |
|  |  |  | Inverse-variance weighted (fixed) | 17 | 0.93 | 0.79-1.10 | 0.420 | 0.141 |
|  |  |  | Weighted median | 17 | 0.90 | 0.70-1.16 | 0.413 |  |
|  |  |  | MR-PRESSO test | 17 | 0.93 | 0.77-1.13 | 0.502 |  |
|  |  |  | MR-Egger | 17 | / | / | 0.391* |  |
| Coriobacteriaceae | 2.99% | 26.93 |  |  |  |  |  |  |
|  |  |  | Inverse-variance weighted (fixed) | 21 | 0.99 | 0.81-1.20 | 0.900 | 0.683 |
|  |  |  | Weighted median | 21 | 1.01 | 0.76-1.34 | 0.945 |  |
|  |  |  | MR-PRESSO test | 20 | 0.99 | 0.83-1.18 | 0.891 |  |
|  |  |  | MR-Egger | 21 | / | / | 0.960* |  |
| Defluviitaleaceae | 3.59% | 52.53 |  |  |  |  |  |  |
|  |  |  | Inverse-variance weighted (fixed) | 13 | 1.19 | 1.00-1.42 | 0.052 | 0.958 |
|  |  |  | Weighted median | 13 | 1.10 | 0.88-1.39 | 0.401 |  |
|  |  |  | MR-PRESSO test | 13 | 1.19 | 1.06-1.34 | 0.011 |  |
|  |  |  | MR-Egger | 13 | / | / | 0.979* |  |
| Desulfovibrionaceae | 2.00% | 31.20 |  |  |  |  |  |  |
|  |  |  | Inverse-variance weighted (fixed) | 12 | 1.06 | 0.85-1.34 | 0.588 | 0.184 |
|  |  |  | Weighted median | 12 | 1.08 | 0.78-1.49 | 0.653 |  |
|  |  |  | MR-PRESSO test | 12 | 1.06 | 0.82-1.39 | 0.651 |  |
|  |  |  | MR-Egger | 12 | / | / | 0.759* |  |
| Enterobacteriaceae | 1.84% | 31.17 |  |  |  |  |  |  |
|  |  |  | Inverse-variance weighted (fixed) | 11 | 1.22 | 0.95-1.58 | 0.122 | 0.071 |
|  |  |  | Weighted median | 11 | 1.20 | 0.81-1.77 | 0.373 |  |
|  |  |  | MR-PRESSO test | 11 | 1.22 | 0.88-1.71 | 0.265 |  |
|  |  |  | MR-Egger | 11 | / | / | 0.589* |  |
| Erysipelotrichaceae | 1.65% | 23.71 |  |  |  |  |  |  |
|  |  |  | Inverse-variance weighted (fixed) | 13 | 1.04 | 0.80-1.36 | 0.747 | 0.450 |
|  |  |  | Weighted median | 13 | 1.17 | 0.82-1.67 | 0.391 |  |
|  |  |  | MR-PRESSO test | 13 | 1.04 | 0.80-1.36 | 0.752 |  |
|  |  |  | MR-Egger | 13 | / | / | 0.184* |  |
| FamilyXI (ID: 1936) | 6.97% | 137.38 |  |  |  |  |  |  |
|  |  |  | Inverse-variance weighted (fixed) | 10 | 1.01 | 0.90-1.14 | 0.838 | 0.358 |
|  |  |  | Weighted median | 10 | 1.00 | 0.85-1.19 | 0.956 |  |
|  |  |  | MR-PRESSO test | 10 | 1.01 | 0.89-1.15 | 0.850 |  |
|  |  |  | MR-Egger | 10 | / | / | 0.771* |  |
| FamilyXIII (ID: 1957) | 2.24% | 30.01 |  |  |  |  |  |  |
|  |  |  | Inverse-variance weighted (random) | 12 | 0.98 | 0.68-1.40 | 0.893 | 0.043 |
|  |  |  | Weighted median | 12 | 1.12 | 0.75-1.67 | 0.592 |  |
|  |  |  | MR-PRESSO test | 12 | 0.98 | 0.68-1.40 | 0.896 |  |
|  |  |  | MR-Egger | 12 | / | / | 0.202* |  |
| Lachnospiraceae | 2.61% | 27.33 |  |  |  |  |  |  |
|  |  |  | Inverse-variance weighted (fixed) | 18 | 1.18 | 0.96-1.45 | 0.120 | 0.605 |
|  |  |  | Weighted median | 18 | 1.14 | 0.84-1.55 | 0.387 |  |
|  |  |  | MR-PRESSO test | 18 | 1.18 | 0.97-1.43 | 0.114 |  |
|  |  |  | MR-Egger | 18 | / | / | 0.567* |  |
| Lactobacillaceae | 4.43% | 65.42 |  |  |  |  |  |  |
|  |  |  | Inverse-variance weighted (fixed) | 13 | 0.97 | 0.83-1.14 | 0.732 | 0.594 |
|  |  |  | Weighted median | 13 | 0.87 | 0.71-1.08 | 0.210 |  |
|  |  |  | MR-PRESSO test | 13 | 0.97 | 0.84-1.13 | 0.718 |  |
|  |  |  | MR-Egger | 13 | / | / | 0.650* |  |
| Methanobacteriaceae | 7.23% | 119.05 |  |  |  |  |  |  |
|  |  |  | Inverse-variance weighted (fixed) | 12 | 0.97 | 0.86-1.09 | 0.611 | 0.107 |
|  |  |  | Weighted median | 12 | 0.99 | 0.84-1.17 | 0.915 |  |
|  |  |  | MR-PRESSO test | 12 | 0.97 | 0.84-1.12 | 0.691 |  |
|  |  |  | MR-Egger | 12 | / | / | 0.812* |  |
| Oxalobacteraceae | 6.93% | 90.93 |  |  |  |  |  |  |
|  |  |  | Inverse-variance weighted (random) | 15 | 1.10 | 0.93-1.30 | 0.268 | 0.040 |
|  |  |  | Weighted median | 15 | 1.19 | 0.99-1.44 | 0.063 |  |
|  |  |  | MR-PRESSO test | 15 | 1.10 | 0.93-1.30 | 0.286 |  |
|  |  |  | MR-Egger | 15 | / | / | 0.448* |  |
| Pasteurellaceae | 5.52% | 56.29 |  |  |  |  |  |  |
|  |  |  | Inverse-variance weighted (fixed) | 19 | 0.96 | 0.83-1.10 | 0.558 | 0.243 |
|  |  |  | Weighted median | 19 | 1.08 | 0.88-1.33 | 0.452 |  |
|  |  |  | MR-PRESSO test | 19 | 0.96 | 0.82-1.12 | 0.601 |  |
|  |  |  | MR-Egger | 19 | / | / | 0.153* |  |
| Peptococcaceae | 2.80% | 52.72 |  |  |  |  |  |  |
|  |  |  | Inverse-variance weighted (fixed) | 10 | 1.04 | 0.85-1.27 | 0.702 | 0.288 |
|  |  |  | Weighted median | 10 | 0.93 | 0.70-1.24 | 0.630 |  |
|  |  |  | MR-PRESSO test | 10 | 1.04 | 0.83-1.30 | 0.735 |  |
|  |  |  | MR-Egger | 10 | / | / | 0.713* |  |
| Peptostreptococcaceae | 3.05% | 36.03 |  |  |  |  |  |  |
|  |  |  | Inverse-variance weighted (fixed) | 16 | 0.99 | 0.82-1.21 | 0.951 | 0.481 |
|  |  |  | Weighted median | 16 | 0.93 | 0.71-1.24 | 0.630 |  |
|  |  |  | MR-PRESSO test | 16 | 0.99 | 0.82-1.21 | 0.951 |  |
|  |  |  | MR-Egger | 16 | / | / | 0.545* |  |
| Porphyromonadaceae | 1.46% | 22.60 |  |  |  |  |  |  |
|  |  |  | Inverse-variance weighted (fixed) | 12 | 0.93 | 0.71-1.21 | 0.580 | 0.269 |
|  |  |  | Weighted median | 12 | 0.88 | 0.60-1.28 | 0.501 |  |
|  |  |  | MR-PRESSO test | 12 | 0.93 | 0.69-1.24 | 0.626 |  |
|  |  |  | MR-Egger | 12 | / | / | 0.502* |  |
| Prevotellaceae | 3.07% | 32.27 |  |  |  |  |  |  |
|  |  |  | Inverse-variance weighted (random) | 18 | 0.78 | 0.65-0.95 | 0.012 | 0.321 |
|  |  |  | Weighted median | 18 | 0.83 | 0.63-1.09 | 0.187 |  |
|  |  |  | MR-PRESSO test | 18 | 0.78 | 0.64-0.96 | 0.029 |  |
|  |  |  | MR-Egger | 18 | / | / | 0.428* |  |
| Rhodospirillaceae | 3.91% | 46.76 |  |  |  |  |  |  |
|  |  |  | Inverse-variance weighted (fixed) | 16 | 1.02 | 0.87-1.20 | 0.805 | 0.992 |
|  |  |  | Weighted median | 16 | 0.99 | 0.79-1.23 | 0.925 |  |
|  |  |  | MR-PRESSO test | 16 | 1.02 | 0.93-1.12 | 0.675 |  |
|  |  |  | MR-Egger | 16 | / | / | 0.657* |  |
| Rikenellaceae | 3.07% | 25.20 |  |  |  |  |  |  |
|  |  |  | Inverse-variance weighted (fixed) | 23 | 1.03 | 0.85-1.24 | 0.776 | 0.833 |
|  |  |  | Weighted median | 23 | 1.09 | 0.84-1.42 | 0.509 |  |
|  |  |  | MR-PRESSO test | 23 | 1.03 | 0.88-1.21 | 0.739 |  |
|  |  |  | MR-Egger | 23 | / | / | 0.831* |  |
| Ruminococcaceae | 1.90% | 29.58 |  |  |  |  |  |  |
|  |  |  | Inverse-variance weighted (fixed) | 12 | 0.86 | 0.65-1.14 | 0.238 | 0.218 |
|  |  |  | Weighted median | 12 | 0.85 | 0.61-1.18 | 0.334 |  |
|  |  |  | MR-PRESSO test | 12 | 0.86 | 0.65-1.14 | 0.322 |  |
|  |  |  | MR-Egger | 12 | / | / | 0.224* |  |
| Streptococcaceae | 2.70% | 26.71 |  |  |  |  |  |  |
|  |  |  | Inverse-variance weighted (fixed) | 19 | 1.01 | 0.82-1.23 | 0.956 | 0.396 |
|  |  |  | Weighted median | 19 | 0.97 | 0.72-1.29 | 0.810 |  |
|  |  |  | MR-PRESSO test | 19 | 1.01 | 0.82-1.23 | 0.958 |  |
|  |  |  | MR-Egger | 19 | / | / | 0.756* |  |
| Veillonellaceae | 3.79% | 34.40 |  |  |  |  |  |  |
|  |  |  | Inverse-variance weighted (fixed) | 21 | 0.80 | 0.86-1.22 | 0.790 | 0.293 |
|  |  |  | Weighted median | 21 | 1.14 | 0.90-1.45 | 0.285 |  |
|  |  |  | MR-PRESSO test | 21 | 1.02 | 0.85-1.23 | 0.806 |  |
|  |  |  | MR-Egger | 21 | / | / | 0.902* |  |
| Verrucomicrobiaceae | 2.63% | 38.04 |  |  |  |  |  |  |
|  |  |  | Inverse-variance weighted (fixed) | 13 | 0.86 | 0.70-1.05 | 0.139 | 0.096 |
|  |  |  | Weighted median | 13 | 0.89 | 0.66-1.20 | 0.448 |  |
|  |  |  | MR-PRESSO test | 13 | 0.86 | 0.66-1.11 | 0.259 |  |
|  |  |  | MR-Egger | 13 | / | / | 0.933* |  |
| Victivallaceae | 9.17% | 123.35 |  |  |  |  |  |  |
|  |  |  | Inverse-variance weighted (fixed) | 15 | 0.91 | 0.81-1.01 | 0.081 | 0.846 |
|  |  |  | Weighted median | 15 | 0.93 | 0.80-1.07 | 0.292 |  |
|  |  |  | MR-PRESSO test | 15 | 0.91 | 0.83-0.99 | 0.045 |  |
|  |  |  | MR-Egger | 15 | / | / | 0.690* |  |
| Genus |  |  |  |  |  |  |  |  |
| Clostridiuminnocuum | 5.90% | 104.48 |  |  |  |  |  |  |
|  |  |  | Inverse-variance weighted (fixed) | 11 | 0.87 | 0.76-1.00 | 0.046 | 0.625 |
|  |  |  | Weighted median | 11 | 0.89 | 0.74-1.07 | 0.200 |  |
|  |  |  | MR-PRESSO test | 11 | 0.87 | 0.77-0.98 | 0.050 |  |
|  |  |  | MR-Egger | 11 | / | / | 0.054* |  |
| Eubacteriumbrachy | 5.35% | 103.67 |  |  |  |  |  |  |
|  |  |  | Inverse-variance weighted (fixed) | 10 | 0.96 | 0.84-1.11 | 0.611 | 0.354 |
|  |  |  | Weighted median | 10 | 1.03 | 0.84-1.25 | 0.792 |  |
|  |  |  | MR-PRESSO test | 10 | 0.96 | 0.83-1.12 | 0.640 |  |
|  |  |  | MR-Egger | 10 | / | / | 0.709* |  |
| Eubacteriumcoprostanoligenes | 1.95% | 24.32 |  |  |  |  |  |  |
|  |  |  | Inverse-variance weighted (fixed) | 15 | 0.99 | 0.78-1.26 | 0.948 | 0.644 |
|  |  |  | Weighted median | 15 | 1.04 | 0.74-1.45 | 0.836 |  |
|  |  |  | MR-PRESSO test | 15 | 0.99 | 0.80-1.23 | 0.944 |  |
|  |  |  | MR-Egger | 15 | / | / | 0.643* |  |
| Eubacteriumeligens | 1.85% | 31.49 |  |  |  |  |  |  |
|  |  |  | Inverse-variance weighted (fixed) | 11 | 1.07 | 0.83-1.38 | 0.599 | 0.051 |
|  |  |  | Weighted median | 11 | 1.00 | 0.70-1.43 | 0.996 |  |
|  |  |  | MR-PRESSO test | 11 | 1.07 | 0.76-1.50 | 0.705 |  |
|  |  |  | Outlier corrected (MR-PRESSO) | 9 | 1.14 | 0.93-1.39 | 0.249 |  |
|  |  |  | MR-Egger | 9 | / | / | 0.075* |  |
| Eubacteriumfissicatena | 5.01% | 107.36 |  |  |  |  |  |  |
|  |  |  | Inverse-variance weighted (fixed) | 9 | 1.02 | 0.88-1.18 | 0.788 | 0.423 |
|  |  |  | Weighted median | 9 | 0.96 | 0.80-1.16 | 0.701 |  |
|  |  |  | MR-PRESSO test | 9 | 1.02 | 0.88-1.18 | 0.796 |  |
|  |  |  | MR-Egger | 9 | / | / | 0.147* |  |
| Eubacteriumhallii | 2.82% | 33.25 |  |  |  |  |  |  |
|  |  |  | Inverse-variance weighted (random) | 16 | 0.70 | 0.48-1.01 | 0.054 | 1.40E-04 |
|  |  |  | Weighted median | 16 | 0.84 | 0.61-1.16 | 0.283 |  |
|  |  |  | MR-PRESSO test | 16 | 0.70 | 0.48-1.01 | 0.073 |  |
|  |  |  | Outlier corrected (MR-PRESSO) | 15 | 0.80 | 0.63-1.01 | 0.083 |  |
|  |  |  | MR-Egger | 16 | / | / | 0.100* |  |
| Eubacteriumnodatum | 7.15% | 128.36 |  |  |  |  |  |  |
|  |  |  | Inverse-variance weighted (fixed) | 11 | 0.98 | 0.87-1.10 | 0.721 | 0.413 |
|  |  |  | Weighted median | 11 | 1.02 | 0.87-1.19 | 0.797 |  |
|  |  |  | MR-PRESSO test | 11 | 0.98 | 0.87-1.11 | 0.732 |  |
|  |  |  | MR-Egger | 11 | / | / | 0.518* |  |
| Eubacteriumoxidoreducens | 2.20% | 82.61 |  |  |  |  |  |  |
|  |  |  | Inverse-variance weighted (fixed) | 5 | 0.93 | 0.67-1.30 | 0.557 | 0.082 |
|  |  |  | Weighted median | 5 | 0.86 | 0.61-1.20 | 0.374 |  |
|  |  |  | MR-PRESSO test | 5 | 0.93 | 0.67-1.30 | 0.704 |  |
|  |  |  | MR-Egger | 5 | / | / | 0.048* |  |
| Eubacteriumrectale | 1.78% | 25.52 |  |  |  |  |  |  |
|  |  |  | Inverse-variance weighted (fixed) | 13 | 0.98 | 0.77-1.25 | 0.884 | 0.560 |
|  |  |  | Weighted median | 13 | 1.02 | 0.72-1.44 | 0.908 |  |
|  |  |  | MR-PRESSO test | 13 | 0.98 | 0.78-1.23 | 0.879 |  |
|  |  |  | MR-Egger | 13 | / | / | 0.157* |  |
| Eubacteriumruminantium | 5.88% | 60.26 |  |  |  |  |  |  |
|  |  |  | Inverse-variance weighted (fixed) | 19 | 1.04 | 0.90-1.19 | 0.609 | 0.738 |
|  |  |  | Weighted median | 19 | 1.07 | 0.87-1.30 | 0.526 |  |
|  |  |  | MR-PRESSO test | 19 | 1.04 | 0.92-1.17 | 0.568 |  |
|  |  |  | MR-Egger | 19 | / | / | 0.629* |  |
| Eubacteriumventriosum | 2.60% | 28.78 |  |  |  |  |  |  |
|  |  |  | Inverse-variance weighted (fixed) | 17 | 1.05 | 0.85-1.30 | 0.652 | 0.927 |
|  |  |  | Weighted median | 17 | 1.08 | 0.81-1.44 | 0.585 |  |
|  |  |  | MR-PRESSO test | 17 | 1.05 | 0.90-1.23 | 0.549 |  |
|  |  |  | MR-Egger | 17 | / | / | 0.600* |  |
| Eubacteriumxylanophilum | 2.11% | 32.91 |  |  |  |  |  |  |
|  |  |  | Inverse-variance weighted (fixed) | 12 | 1.06 | 0.84-1.34 | 0.625 | 0.321 |
|  |  |  | Weighted median | 12 | 1.02 | 0.73-1.41 | 0.918 |  |
|  |  |  | MR-PRESSO test | 12 | 1.06 | 0.83-1.36 | 0.656 |  |
|  |  |  | MR-Egger | 12 | / | / | 0.525* |  |
| Ruminococcusgauvreauii | 2.15% | 31.00 |  |  |  |  |  |  |
|  |  |  | Inverse-variance weighted (fixed) | 13 | 0.94 | 0.75-1.18 | 0.606 | 0.058 |
|  |  |  | Weighted median | 13 | 1.00 | 0.72-1.38 | 0.994 |  |
|  |  |  | MR-PRESSO test | 13 | 0.94 | 0.70-1.27 | 0.700 |  |
|  |  |  | MR-Egger | 13 | / | / | 0.479* |  |
| Ruminococcusgnavus | 4.08% | 64.94 |  |  |  |  |  |  |
|  |  |  | Inverse-variance weighted (fixed) | 12 | 1.08 | 0.92-1.26 | 0.367 | 0.059 |
|  |  |  | Weighted median | 12 | 0.96 | 0.77-1.19 | 0.691 |  |
|  |  |  | MR-PRESSO test | 12 | 1.08 | 0.87-1.33 | 0.508 |  |
|  |  |  | MR-Egger | 12 | / | / | 0.224* |  |
| Ruminococcustorques | 2.08% | 26.00 |  |  |  |  |  |  |
|  |  |  | Inverse-variance weighted (random) | 15 | 0.82 | 0.56-1.20 | 0.302 | 1.70E-04 |
|  |  |  | Weighted median | 15 | 0.91 | 0.64-1.30 | 0.598 |  |
|  |  |  | MR-PRESSO test | 15 | 0.82 | 0.56-1.20 | 0.320 |  |
|  |  |  | Outlier corrected (MR-PRESSO) | 15 | 0.96 | 0.72-1.28 | 0.776 |  |
|  |  |  | MR-Egger | 15 | / | / | 0.516* |  |
| Actinomyces | 2.66% | 62.61 |  |  |  |  |  |  |
|  |  |  | Inverse-variance weighted (fixed) | 8 | 0.92 | 0.76-1.13 | 0.436 | 0.693 |
|  |  |  | Weighted median | 8 | 0.93 | 0.72-1.20 | 0.564 |  |
|  |  |  | MR-PRESSO test | 8 | 0.92 | 0.79-1.09 | 0.375 |  |
|  |  |  | MR-Egger | 8 | / | / | 0.750* |  |
| Adlercreutzia | 3.11% | 49.06 |  |  |  |  |  |  |
|  |  |  | Inverse-variance weighted (fixed) | 12 | 1.09 | 0.90-1.31 | 0.383 | 0.986 |
|  |  |  | Weighted median | 12 | 1.07 | 0.84-1.36 | 0.599 |  |
|  |  |  | MR-PRESSO test | 12 | 1.09 | 0.98-1.20 | 0.140 |  |
|  |  |  | MR-Egger | 12 | / | / | 0.929* |  |
| Akkermansia | 2.63% | 38.06 |  |  |  |  |  |  |
|  |  |  | Inverse-variance weighted (fixed) | 13 | 0.86 | 0.70-1.05 | 0.139 | 0.096 |
|  |  |  | Weighted median | 13 | 0.89 | 0.66-1.20 | 0.447 |  |
|  |  |  | MR-PRESSO test | 13 | 0.86 | 0.66-1.11 | 0.259 |  |
|  |  |  | MR-Egger | 13 | / | / | 0.936* |  |
| Alistipes | 1.80% | 22.37 |  |  |  |  |  |  |
|  |  |  | Inverse-variance weighted (fixed) | 15 | 0.96 | 0.75-1.22 | 0.743 | 0.683 |
|  |  |  | Weighted median | 15 | 0.94 | 0.68-1.31 | 0.726 |  |
|  |  |  | MR-PRESSO test | 15 | 0.96 | 0.78-1.19 | 0.717 |  |
|  |  |  | MR-Egger | 15 | / | / | 0.574* |  |
| Allisonella | 6.06% | 131.44 |  |  |  |  |  |  |
|  |  |  | Inverse-variance weighted (fixed) | 9 | 1.03 | 0.91-1.18 | 0.614 | 0.265 |
|  |  |  | Weighted median | 9 | 1.07 | 0.90-1.28 | 0.428 |  |
|  |  |  | MR-PRESSO test | 9 | 1.03 | 0.89-1.20 | 0.664 |  |
|  |  |  | MR-Egger | 9 | / | / | 0.720* |  |
| Alloprevotella | 4.85% | 133.35 |  |  |  |  |  |  |
|  |  |  | Inverse-variance weighted (fixed) | 7 | 0.95 | 0.82-1.10 | 0.506 | 0.947 |
|  |  |  | Weighted median | 7 | 0.93 | 0.78-1.12 | 0.459 |  |
|  |  |  | MR-PRESSO test | 7 | 0.95 | 0.88-1.03 | 0.255 |  |
|  |  |  | MR-Egger | 7 | / | / | 0.988* |  |
| Anaerofilum | 5.45% | 88.11 |  |  |  |  |  |  |
|  |  |  | Inverse-variance weighted (fixed) | 12 | 1.00 | 0.87-1.15 | 0.996 | 0.584 |
|  |  |  | Weighted median | 12 | 1.02 | 0.83-1.24 | 0.874 |  |
|  |  |  | MR-PRESSO test | 12 | 1.00 | 0.88-1.14 | 0.996 |  |
|  |  |  | MR-Egger | 12 | / | / | 0.255* |  |
| Anaerostipes | 2.19% | 27.40 |  |  |  |  |  |  |
|  |  |  | Inverse-variance weighted (fixed) | 15 | 1.14 | 0.90-1.43 | 0.274 | 0.841 |
|  |  |  | Weighted median | 15 | 1.23 | 0.91-1.67 | 0.180 |  |
|  |  |  | MR-PRESSO test | 15 | 1.14 | 0.95-1.36 | 0.190 |  |
|  |  |  | MR-Egger | 15 | / | / | 0.951* |  |
| Anaerotruncus | 2.14% | 25.10 |  |  |  |  |  |  |
|  |  |  | Inverse-variance weighted (fixed) | 15 | 0.87 | 0.69-1.10 | 0.251 | 0.749 |
|  |  |  | Weighted median | 15 | 0.93 | 0.68-1.26 | 0.627 |  |
|  |  |  | MR-PRESSO test | 15 | 0.87 | 0.72-1.06 | 0.199 |  |
|  |  |  | MR-Egger | 15 | / | / | 0.640* |  |
| Bacteroides | 1.47% | 27.38 |  |  |  |  |  |  |
|  |  |  | Inverse-variance weighted (fixed) | 10 | 1.16 | 0.86-1.56 | 0.339 | 0.735 |
|  |  |  | Weighted median | 10 | 1.21 | 0.80-1.82 | 0.361 |  |
|  |  |  | MR-PRESSO test | 10 | 1.16 | 0.91-1.47 | 0.274 |  |
|  |  |  | MR-Egger | 10 | / | / | 0.716* |  |
| Barnesiella | 2.63% | 31.01 |  |  |  |  |  |  |
|  |  |  | Inverse-variance weighted (fixed) | 16 | 1.09 | 0.88-1.34 | 0.435 | 0.345 |
|  |  |  | Weighted median | 16 | 1.01 | 0.75-1.34 | 0.963 |  |
|  |  |  | MR-PRESSO test | 16 | 1.09 | 0.87-1.36 | 0.469 |  |
|  |  |  | MR-Egger | 16 | / | / | 0.399* |  |
| Bifidobacterium | 4.32% | 37.58 |  |  |  |  |  |  |
|  |  |  | Inverse-variance weighted (fixed) | 22 | 1.04 | 0.88-1.23 | 0.617 | 0.216 |
|  |  |  | Weighted median | 22 | 1.00 | 0.80-1.27 | 0.969 |  |
|  |  |  | MR-PRESSO test | 22 | 1.04 | 0.86-1.26 | 0.672 |  |
|  |  |  | MR-Egger | 22 | / | / | 0.527* |  |
| Bilophila | 2.82% | 31.30 |  |  |  |  |  |  |
|  |  |  | Inverse-variance weighted (random) | 17 | 1.11 | 0.86-1.43 | 0.434 | 0.039 |
|  |  |  | Weighted median | 17 | 1.01 | 0.76-1.35 | 0.941 |  |
|  |  |  | MR-PRESSO test | 17 | 1.11 | 0.86-1.43 | 0.446 |  |
|  |  |  | MR-Egger | 17 | / | / | 0.456* |  |
| Blautia | 2.01% | 28.89 |  |  |  |  |  |  |
|  |  |  | Inverse-variance weighted (fixed) | 13 | 1.14 | 0.90-1.44 | 0.284 | 0.546 |
|  |  |  | Weighted median | 13 | 1.14 | 0.83-1.58 | 0.422 |  |
|  |  |  | MR-PRESSO test | 13 | 1.14 | 0.91-1.42 | 0.281 |  |
|  |  |  | MR-Egger | 13 | / | / | 0.922* |  |
| Butyricicoccus | 1.95% | 40.56 |  |  |  |  |  |  |
|  |  |  | Inverse-variance weighted (random) | 9 | 0.92 | 0.62-1.36 | 0.670 | 0.008 |
|  |  |  | Weighted median | 9 | 0.89 | 0.63-1.27 | 0.527 |  |
|  |  |  | MR-PRESSO test | 9 | 0.92 | 0.62-1.36 | 0.681 |  |
|  |  |  | Outlier corrected (MR-PRESSO) | 8 | 0.83 | 0.60-1.15 | 0.300 |  |
|  |  |  | MR-Egger | 9 | / | / | 0.864* |  |
| Butyricimonas | 3.92% | 41.48 |  |  |  |  |  |  |
|  |  |  | Inverse-variance weighted (fixed) | 18 | 0.96 | 0.81-1.14 | 0.650 | 0.151 |
|  |  |  | Weighted median | 18 | 1.01 | 0.80-1.27 | 0.952 |  |
|  |  |  | MR-PRESSO test | 18 | 0.96 | 0.79-1.17 | 0.700 |  |
|  |  |  | MR-Egger | 18 | / | / | 0.819* |  |
| Butyrivibrio | 10.11% | 128.73 |  |  |  |  |  |  |
|  |  |  | Inverse-variance weighted (fixed) | 16 | 0.90 | 0.81-1.00 | 0.056 | 0.217 |
|  |  |  | Weighted median | 16 | 0.90 | 0.78-1.03 | 0.135 |  |
|  |  |  | MR-PRESSO test | 16 | 0.90 | 0.80-1.02 | 0.110 |  |
|  |  |  | MR-Egger | 16 | / | / | 0.090* |  |
| CandidatusSoleaferrea | 5.83% | 70.84 |  |  |  |  |  |  |
|  |  |  | Inverse-variance weighted (fixed) | 16 | 0.99 | 0.86-1.13 | 0.838 | 0.827 |
|  |  |  | Weighted median | 16 | 0.94 | 0.78-1.14 | 0.523 |  |
|  |  |  | MR-PRESSO test | 16 | 0.99 | 0.88-1.10 | 0.804 |  |
|  |  |  | MR-Egger | 16 | / | / | 0.805* |  |
| Catenibacterium | 3.28% | 124.43 |  |  |  |  |  |  |
|  |  |  | Inverse-variance weighted (fixed) | 5 | 0.90 | 0.64-1.27 | 0.563 | 0.050 |
|  |  |  | Weighted median | 5 | 0.97 | 0.75-1.26 | 0.821 |  |
|  |  |  | MR-PRESSO test | 5 | 0.90 | 0.64-1.27 | 0.595 |  |
|  |  |  | Outlier corrected (MR-PRESSO) | 4 | 1.09 | 0.92-1.29 | 0.403 |  |
|  |  |  | MR-Egger | 5 | / | / | 0.620* |  |
| ChristensenellaceaeR.7 | 1.50% | 25.33 |  |  |  |  |  |  |
|  |  |  | Inverse-variance weighted (fixed) | 11 | 0.86 | 0.65-1.13 | 0.277 | 0.118 |
|  |  |  | Weighted median | 11 | 0.90 | 0.62-1.30 | 0.580 |  |
|  |  |  | MR-PRESSO test | 11 | 0.86 | 0.61-1.21 | 0.402 |  |
|  |  |  | MR-Egger | 11 | / | / | 0.222* |  |
| Clostridiumsensustricto1 | 1.86% | 38.54 |  |  |  |  |  |  |
|  |  |  | Inverse-variance weighted (fixed) | 9 | 0.79 | 0.57-1.09 | 0.055 | 0.078 |
|  |  |  | Weighted median | 9 | 0.96 | 0.68-1.35 | 0.818 |  |
|  |  |  | MR-PRESSO test | 9 | 0.79 | 0.57-1.09 | 0.186 |  |
|  |  |  | MR-Egger | 9 | / | / | 0.035* |  |
| Collinsella | 1.93% | 27.67 |  |  |  |  |  |  |
|  |  |  | Inverse-variance weighted (random) | 13 | 0.93 | 0.67-1.29 | 0.667 | 0.024 |
|  |  |  | Weighted median | 13 | 0.87 | 0.61-1.23 | 0.425 |  |
|  |  |  | MR-PRESSO test | 13 | 0.93 | 0.67-1.29 | 0.675 |  |
|  |  |  | MR-Egger | 13 | / | / | 0.372* |  |
| Coprobacter | 5.29% | 73.18 |  |  |  |  |  |  |
|  |  |  | Inverse-variance weighted (random) | 14 | 0.97 | 0.79-1.19 | 0.752 | 0.030 |
|  |  |  | Weighted median | 14 | 1.00 | 0.80-1.25 | 0.990 |  |
|  |  |  | MR-PRESSO test | 13 | 0.97 | 0.79-1.19 | 0.757 |  |
|  |  |  | MR-Egger | 14 | / | / | 0.368* |  |
| Coprococcus1 | 2.38% | 31.91 |  |  |  |  |  |  |
|  |  |  | Inverse-variance weighted (fixed) | 14 | 0.96 | 0.77-1.20 | 0.722 | 0.721 |
|  |  |  | Weighted median | 14 | 0.90 | 0.67-1.22 | 0.511 |  |
|  |  |  | MR-PRESSO test | 14 | 0.96 | 0.80-1.16 | 0.687 |  |
|  |  |  | MR-Egger | 14 | / | / | 0.762* |  |
| Coprococcus2 | 2.36% | 36.88 |  |  |  |  |  |  |
|  |  |  | Inverse-variance weighted (fixed) | 12 | 1.06 | 0.85-1.31 | 0.624 | 0.996 |
|  |  |  | Weighted median | 12 | 1.08 | 0.82-1.41 | 0.595 |  |
|  |  |  | MR-PRESSO test | 12 | 1.06 | 0.95-1.17 | 0.318 |  |
|  |  |  | MR-Egger | 12 | / | / | 0.487* |  |
| Coprococcus3 | 1.62% | 27.50 |  |  |  |  |  |  |
|  |  |  | Inverse-variance weighted (fixed) | 11 | 1.11 | 0.85-1.45 | 0.448 | 0.406 |
|  |  |  | Weighted median | 11 | 1.14 | 0.79-1.64 | 0.472 |  |
|  |  |  | MR-PRESSO test | 11 | 1.11 | 0.84-1.46 | 0.474 |  |
|  |  |  | MR-Egger | 11 | / | / | 0.212* |  |
| DefluviitaleaceaeUCG011 | 3.18% | 54.72 |  |  |  |  |  |  |
|  |  |  | Inverse-variance weighted (fixed) | 11 | 1.25 | 1.03-1.50 | 0.023 | 0.964 |
|  |  |  | Weighted median | 11 | 1.18 | 0.93-1.49 | 0.180 |  |
|  |  |  | MR-PRESSO test | 11 | 1.25 | 1.11-1.39 | 0.003 |  |
|  |  |  | MR-Egger | 11 | / | / | 0.826* |  |
| Desulfovibrio | 3.18% | 50.13 |  |  |  |  |  |  |
|  |  |  | Inverse-variance weighted (fixed) | 12 | 1.16 | 0.96-1.40 | 0.122 | 0.161 |
|  |  |  | Weighted median | 12 | 1.07 | 0.81-1.40 | 0.651 |  |
|  |  |  | MR-PRESSO test | 12 | 1.16 | 0.93-1.45 | 0.219 |  |
|  |  |  | MR-Egger | 12 | / | / | 0.571* |  |
| Dialister | 2.16% | 33.65 |  |  |  |  |  |  |
|  |  |  | Inverse-variance weighted (fixed) | 12 | 0.92 | 0.74-1.16 | 0.495 | 0.151 |
|  |  |  | Weighted median | 12 | 1.00 | 0.73-1.37 | 0.988 |  |
|  |  |  | MR-PRESSO test | 12 | 0.92 | 0.71-1.21 | 0.580 |  |
|  |  |  | MR-Egger | 12 | / | / | 0.596* |  |
| Dorea | 1.82% | 26.17 |  |  |  |  |  |  |
|  |  |  | Inverse-variance weighted (fixed) | 13 | 0.91 | 0.72-1.16 | 0.454 | 0.874 |
|  |  |  | Weighted median | 13 | 0.89 | 0.65-1.22 | 0.474 |  |
|  |  |  | MR-PRESSO test | 13 | 0.91 | 0.76-1.09 | 0.338 |  |
|  |  |  | MR-Egger | 13 | / | / | 0.919* |  |
| Eggerthella | 4.13% | 79.01 |  |  |  |  |  |  |
|  |  |  | Inverse-variance weighted (fixed) | 10 | 0.88 | 0.75-1.04 | 0.132 | 0.646 |
|  |  |  | Weighted median | 10 | 1.00 | 0.80-1.25 | 0.991 |  |
|  |  |  | MR-PRESSO test | 10 | 0.88 | 0.76-1.02 | 0.120 |  |
|  |  |  | MR-Egger | 10 | / | / | 0.466* |  |
| Eisenbergiella | 4.52% | 72.37 |  |  |  |  |  |  |
|  |  |  | Inverse-variance weighted (fixed) | 12 | 1.08 | 0.92-1.25 | 0.346 | 0.518 |
|  |  |  | Weighted median | 12 | 1.22 | 0.99-1.51 | 0.064 |  |
|  |  |  | MR-PRESSO test | 12 | 1.08 | 0.93-1.24 | 0.348 |  |
|  |  |  | MR-Egger | 12 | / | / | 0.205* |  |
| Enterorhabdus | 3.22% | 67.98 |  |  |  |  |  |  |
|  |  |  | Inverse-variance weighted (fixed) | 9 | 0.82 | 0.68-0.98 | 0.032 | 0.732 |
|  |  |  | Weighted median | 9 | 0.85 | 0.66-1.08 | 0.171 |  |
|  |  |  | MR-PRESSO test | 9 | 0.82 | 0.71-0.95 | 0.029 |  |
|  |  |  | MR-Egger | 9 | / | / | 0.349* |  |
| Erysipelatoclostridium | 4.07% | 45.68 |  |  |  |  |  |  |
|  |  |  | Inverse-variance weighted (fixed) | 17 | 0.86 | 0.73-1.02 | 0.091 | 0.680 |
|  |  |  | Weighted median | 17 | 0.89 | 0.71-1.11 | 0.302 |  |
|  |  |  | MR-PRESSO test | 17 | 0.86 | 0.74-1.01 | 0.078 |  |
|  |  |  | MR-Egger | 17 | / | / | 0.702* |  |
| ErysipelotrichaceaeUCG003 | 3.28% | 34.53 |  |  |  |  |  |  |
|  |  |  | Inverse-variance weighted (fixed) | 18 | 1.07 | 0.89-1.30 | 0.455 | 0.382 |
|  |  |  | Weighted median | 18 | 0.96 | 0.74-1.24 | 0.758 |  |
|  |  |  | MR-PRESSO test | 18 | 1.07 | 0.88-1.30 | 0.479 |  |
|  |  |  | MR-Egger | 18 | / | / | 0.239* |  |
| Escherichia.Shigella | 2.97% | 37.39 |  |  |  |  |  |  |
|  |  |  | Inverse-variance weighted (fixed) | 15 | 1.14 | 0.93-1.39 | 0.224 | 0.687 |
|  |  |  | Weighted median | 15 | 1.11 | 0.84-1.46 | 0.463 |  |
|  |  |  | MR-PRESSO test | 15 | 1.14 | 0.95-1.36 | 0.192 |  |
|  |  |  | MR-Egger | 15 | / | / | 0.159* |  |
| Faecalibacterium | 2.60% | 37.59 |  |  |  |  |  |  |
|  |  |  | Inverse-variance weighted (fixed) | 13 | 0.97 | 0.78-1.21 | 0.813 | 0.285 |
|  |  |  | Weighted median | 13 | 1.00 | 0.72-1.37 | 0.979 |  |
|  |  |  | MR-PRESSO test | 13 | 0.97 | 0.77-1.24 | 0.832 |  |
|  |  |  | MR-Egger | 13 | / | / | 0.173* |  |
| FamilyXIIIAD3011 (ID: 11293) | 2.24% | 30.01 |  |  |  |  |  |  |
|  |  |  | Inverse-variance weighted (fixed) | 14 | 1.00 | 0.80-1.26 | 0.990 | 0.841 |
|  |  |  | Weighted median | 14 | 1.07 | 0.79-1.46 | 0.662 |  |
|  |  |  | MR-PRESSO test | 14 | 1.00 | 0.84-1.20 | 0.987 |  |
|  |  |  | MR-Egger | 14 | / | / | 0.953* |  |
| FamilyXIIIUCG001 (ID: 11294) | 1.94% | 36.25 |  |  |  |  |  |  |
|  |  |  | Inverse-variance weighted (fixed) | 10 | 1.21 | 0.95-1.54 | 0.122 | 0.717 |
|  |  |  | Weighted median | 10 | 1.27 | 0.90-1.78 | 0.174 |  |
|  |  |  | MR-PRESSO test | 10 | 1.21 | 0.99-1.48 | 0.096 |  |
|  |  |  | MR-Egger | 10 | / | / | 0.523* |  |
| Flavonifractor | 2.19% | 40.97 |  |  |  |  |  |  |
|  |  |  | Inverse-variance weighted (fixed) | 10 | 0.94 | 0.75-1.18 | 0.604 | 0.079 |
|  |  |  | Weighted median | 10 | 0.90 | 0.64-1.27 | 0.555 |  |
|  |  |  | MR-PRESSO test | 10 | 0.94 | 0.70-1.27 | 0.701 |  |
|  |  |  | MR-Egger | 10 | / | / | 0.584* |  |
| Fusicatenibacter | 2.60% | 24.45 |  |  |  |  |  |  |
|  |  |  | Inverse-variance weighted (fixed) | 20 | 0.97 | 0.79-1.19 | 0.772 | 0.071 |
|  |  |  | Weighted median | 20 | 0.98 | 0.72-1.33 | 0.889 |  |
|  |  |  | MR-PRESSO test | 20 | 0.97 | 0.75-1.25 | 0.816 |  |
|  |  |  | MR-Egger | 20 | / | / | 0.810* |  |
| Gordonibacter | 9.26% | 124.67 |  |  |  |  |  |  |
|  |  |  | Inverse-variance weighted (fixed) | 15 | 1.02 | 0.92-1.14 | 0.672 | 0.956 |
|  |  |  | Weighted median | 15 | 0.99 | 0.86-1.14 | 0.867 |  |
|  |  |  | MR-PRESSO test | 15 | 1.02 | 0.95-1.10 | 0.540 |  |
|  |  |  | MR-Egger | 15 | / | / | 0.420* |  |
| Haemophilus | 4.10% | 55.89 |  |  |  |  |  |  |
|  |  |  | Inverse-variance weighted (fixed) | 14 | 1.06 | 0.90-1.25 | 0.464 | 0.076 |
|  |  |  | Weighted median | 14 | 1.12 | 0.88-1.42 | 0.370 |  |
|  |  |  | MR-PRESSO test | 14 | 1.06 | 0.87-1.30 | 0.573 |  |
|  |  |  | MR-Egger | 14 | / | / | 0.940* |  |
| Holdemanella | 4.79% | 65.87 |  |  |  |  |  |  |
|  |  |  | Inverse-variance weighted (random) | 14 | 0.89 | 0.72-1.10 | 0.296 | 0.034 |
|  |  |  | Weighted median | 14 | 0.82 | 0.65-1.05 | 0.116 |  |
|  |  |  | MR-PRESSO test | 14 | 0.89 | 0.72-1.10 | 0.315 |  |
|  |  |  | MR-Egger | 14 | / | / | 0.220* |  |
| Holdemania | 4.84% | 51.77 |  |  |  |  |  |  |
|  |  |  | Inverse-variance weighted (random) | 18 | 0.92 | 0.76-1.11 | 0.375 | 0.049 |
|  |  |  | Weighted median | 18 | 1.06 | 0.84-1.33 | 0.611 |  |
|  |  |  | MR-PRESSO test | 18 | 0.92 | 0.76-1.11 | 0.388 |  |
|  |  |  | MR-Egger | 18 | / | / | 0.069* |  |
| Howardella | 6.28% | 111.64 |  |  |  |  |  |  |
|  |  |  | Inverse-variance weighted (fixed) | 11 | 0.95 | 0.83-1.08 | 0.440 | 0.910 |
|  |  |  | Weighted median | 11 | 0.97 | 0.82-1.14 | 0.685 |  |
|  |  |  | MR-PRESSO test | 11 | 0.95 | 0.87-1.04 | 0.286 |  |
|  |  |  | MR-Egger | 11 | / | / | 0.663* |  |
| Hungatella | 2.50% | 93.98 |  |  |  |  |  |  |
|  |  |  | Inverse-variance weighted (fixed) | 5 | 0.83 | 0.68-1.03 | 0.085 | 0.387 |
|  |  |  | Weighted median | 5 | 0.94 | 0.70-1.26 | 0.660 |  |
|  |  |  | MR-PRESSO test | 5 | 0.83 | 0.67-1.03 | 0.166 |  |
|  |  |  | MR-Egger | 5 | / | / | 0.326* |  |
| Intestinibacter | 2.71% | 33.98 |  |  |  |  |  |  |
|  |  |  | Inverse-variance weighted (fixed) | 15 | 0.95 | 0.78-1.15 | 0.599 | 0.157 |
|  |  |  | Weighted median | 15 | 0.99 | 0.75-1.32 | 0.971 |  |
|  |  |  | MR-PRESSO test | 15 | 0.95 | 0.76-1.19 | 0.660 |  |
|  |  |  | MR-Egger | 15 | / | / | 0.005* |  |
| Intestinimonas | 4.41% | 42.28 |  |  |  |  |  |  |
|  |  |  | Inverse-variance weighted (fixed) | 20 | 1.01 | 0.86-1.19 | 0.913 | 0.694 |
|  |  |  | Weighted median | 20 | 1.06 | 0.84-1.34 | 0.623 |  |
|  |  |  | MR-PRESSO test | 20 | 1.01 | 0.87-1.17 | 0.904 |  |
|  |  |  | MR-Egger | 20 | / | / | 0.910* |  |
| Lachnoclostridium | 1.86% | 23.18 |  |  |  |  |  |  |
|  |  |  | Inverse-variance weighted (fixed) | 15 | 0.90 | 0.71-1.15 | 0.416 | 0.421 |
|  |  |  | Weighted median | 15 | 1.02 | 0.74-1.40 | 0.916 |  |
|  |  |  | MR-PRESSO test | 15 | 0.90 | 0.71-1.16 | 0.436 |  |
|  |  |  | MR-Egger | 15 | / | / | 0.121* |  |
| Lachnospira | 0.95% | 25.08 |  |  |  |  |  |  |
|  |  |  | Inverse-variance weighted (fixed) | 7 | 0.89 | 0.51-1.57 | 0.693 | 0.009 |
|  |  |  | Weighted median | 7 | 0.70 | 0.41-1.20 | 0.196 |  |
|  |  |  | MR-PRESSO test | 7 | 0.89 | 0.51-1.57 | 0.707 |  |
|  |  |  | Outlier corrected (MR-PRESSO) | 6 | 0.74 | 0.45-1.20 | 0.278 |  |
|  |  |  | MR-Egger | 7 | / | / | 0.623* |  |
| LachnospiraceaeFCS020 | 3.11% | 34.54 |  |  |  |  |  |  |
|  |  |  | Inverse-variance weighted (fixed) | 17 | 1.16 | 0.96-1.40 | 0.117 | 0.427 |
|  |  |  | Weighted median | 17 | 1.17 | 0.90-1.52 | 0.239 |  |
|  |  |  | MR-PRESSO test | 17 | 1.16 | 0.96-1.40 | 0.140 |  |
|  |  |  | MR-Egger | 17 | / | / | 0.796* |  |
| LachnospiraceaeNC2004 | 3.49% | 66.37 |  |  |  |  |  |  |
|  |  |  | Inverse-variance weighted (fixed) | 10 | 0.99 | 0.83-1.18 | 0.923 | 0.908 |
|  |  |  | Weighted median | 10 | 1.07 | 0.86-1.34 | 0.521 |  |
|  |  |  | MR-PRESSO test | 10 | 0.99 | 0.88-1.12 | 0.889 |  |
|  |  |  | MR-Egger | 10 | / | / | 0.681* |  |
| LachnospiraceaeND3007 | 0.57% | 26.17 |  |  |  |  |  |  |
|  |  |  | Inverse-variance weighted (fixed) | 4 | 1.52 | 0.94-2.48 | 0.091 | 0.353 |
|  |  |  | Weighted median | 4 | 1.36 | 0.74-2.50 | 0.326 |  |
|  |  |  | MR-PRESSO test | 4 | 1.52 | 0.92-2.53 | 0.203 |  |
|  |  |  | MR-Egger | 4 | / | / | 0.145* |  |
| LachnospiraceaeNK4A136 | 2.75% | 32.32 |  |  |  |  |  |  |
|  |  |  | Inverse-variance weighted (fixed) | 16 | 0.82 | 0.67-1.00 | 0.054 | 0.984 |
|  |  |  | Weighted median | 16 | 0.85 | 0.64-1.12 | 0.254 |  |
|  |  |  | MR-PRESSO test | 16 | 0.82 | 0.73-0.93 | 0.007 |  |
|  |  |  | MR-Egger | 16 | / | / | 0.270 |  |
| LachnospiraceaeUCG001 | 3.38% | 42.75 |  |  |  |  |  |  |
|  |  |  | Inverse-variance weighted (fixed) | 15 | 0.81 | 0.67-0.97 | 0.023 | 0.527 |
|  |  |  | Weighted median | 15 | 0.75 | 0.58-0.96 | 0.025 |  |
|  |  |  | MR-PRESSO test | 15 | 0.81 | 0.68-0.96 | 0.033 |  |
|  |  |  | MR-Egger | 15 | / | / | 0.940* |  |
| LachnospiraceaeUCG004 | 2.28% | 28.45 |  |  |  |  |  |  |
|  |  |  | Inverse-variance weighted (fixed) | 15 | 0.96 | 0.78-1.19 | 0.728 | 0.834 |
|  |  |  | Weighted median | 15 | 0.97 | 0.73-1.29 | 0.852 |  |
|  |  |  | MR-PRESSO test | 15 | 0.96 | 0.81-1.14 | 0.671 |  |
|  |  |  | MR-Egger | 15 | / | / | 0.918* |  |
| LachnospiraceaeUCG008 | 4.84% | 66.62 |  |  |  |  |  |  |
|  |  |  | Inverse-variance weighted (fixed) | 14 | 1.04 | 0.89-1.21 | 0.625 | 0.630 |
|  |  |  | Weighted median | 14 | 1.00 | 0.82-1.22 | 0.994 |  |
|  |  |  | MR-PRESSO test | 14 | 1.04 | 0.90-1.19 | 0.600 |  |
|  |  |  | MR-Egger | 14 | / | / | 0.509* |  |
| LachnospiraceaeUCG010 | 2.52% | 36.40 |  |  |  |  |  |  |
|  |  |  | Inverse-variance weighted (fixed) | 13 | 1.24 | 1.00-1.53 | 0.045 | 0.148 |
|  |  |  | Weighted median | 13 | 1.35 | 1.00-1.81 | 0.050 |  |
|  |  |  | MR-PRESSO test | 13 | 1.24 | 0.96-1.60 | 0.119 |  |
|  |  |  | MR-Egger | 13 | / | / | 0.673* |  |
| Lactobacillus | 4.16% | 66.24 |  |  |  |  |  |  |
|  |  |  | Inverse-variance weighted (fixed) | 12 | 0.90 | 0.76-1.07 | 0.224 | 0.210 |
|  |  |  | Weighted median | 12 | 0.87 | 0.69-1.09 | 0.224 |  |
|  |  |  | MR-PRESSO test | 12 | 0.90 | 0.74-1.09 | 0.311 |  |
|  |  |  | MR-Egger | 12 | / | / | 0.919* |  |
| Lactococcus | 5.95% | 105.41 |  |  |  |  |  |  |
|  |  |  | Inverse-variance weighted (fixed) | 11 | 0.99 | 0.87-1.13 | 0.871 | 0.286 |
|  |  |  | Weighted median | 11 | 0.95 | 0.79-1.14 | 0.599 |  |
|  |  |  | MR-PRESSO test | 11 | 0.99 | 0.86-1.14 | 0.881 |  |
|  |  |  | MR-Egger | 11 | / | / | 0.792* |  |
| Marvinbryantia | 2.43% | 35.13 |  |  |  |  |  |  |
|  |  |  | Inverse-variance weighted (fixed) | 13 | 1.04 | 0.84-1.29 | 0.693 | 0.312 |
|  |  |  | Weighted median | 13 | 1.03 | 0.76-1.39 | 0.855 |  |
|  |  |  | MR-PRESSO test | 13 | 1.04 | 0.83-1.31 | 0.719 |  |
|  |  |  | MR-Egger | 13 | / | / | 0.455* |  |
| Methanobrevibacter | 4.61% | 110.62 |  |  |  |  |  |  |
|  |  |  | Inverse-variance weighted (fixed) | 8 | 1.10 | 0.95-1.28 | 0.185 | 0.103 |
|  |  |  | Weighted median | 8 | 1.05 | 0.86-1.29 | 0.627 |  |
|  |  |  | MR-PRESSO test | 8 | 1.10 | 0.91-1.34 | 0.344 |  |
|  |  |  | MR-Egger | 8 | / | / | 0.412* |  |
| Odoribacter | 1.45% | 30.01 |  |  |  |  |  |  |
|  |  |  | Inverse-variance weighted (fixed) | 9 | 1.38 | 1.04-1.83 | 0.024 | 0.707 |
|  |  |  | Weighted median | 9 | 1.42 | 0.97-2.07 | 0.070 |  |
|  |  |  | MR-PRESSO test | 9 | 1.38 | 1.10-1.74 | 0.026 |  |
|  |  |  | MR-Egger | 9 | / | / | 0.613* |  |
| Olsenella | 6.41% | 114.06 |  |  |  |  |  |  |
|  |  |  | Inverse-variance weighted (fixed) | 11 | 0.92 | 0.81-1.05 | 0.202 | 0.347 |
|  |  |  | Weighted median | 11 | 0.86 | 0.72-1.03 | 0.095 |  |
|  |  |  | MR-PRESSO test | 11 | 0.92 | 0.80-1.05 | 0.255 |  |
|  |  |  | MR-Egger | 11 | / | / | 0.069* |  |
| Oscillibacter | 4.29% | 51.30 |  |  |  |  |  |  |
|  |  |  | Inverse-variance weighted (fixed) | 16 | 0.90 | 0.77-1.05 | 0.190 | 0.327 |
|  |  |  | Weighted median | 16 | 0.98 | 0.79-1.23 | 0.881 |  |
|  |  |  | MR-PRESSO test | 16 | 0.90 | 0.76-1.06 | 0.236 |  |
|  |  |  | MR-Egger | 16 | / | / | 0.852* |  |
| Oscillospira | 2.17% | 40.72 |  |  |  |  |  |  |
|  |  |  | Inverse-variance weighted (fixed) | 10 | 0.87 | 0.70-1.19 | 0.228 | 0.224 |
|  |  |  | Weighted median | 10 | 0.92 | 0.68-1.24 | 0.580 |  |
|  |  |  | MR-PRESSO test | 10 | 0.87 | 0.67-1.13 | 0.321 |  |
|  |  |  | MR-Egger | 10 | / | / | 0.122* |  |
| Oxalobacter | 6.51% | 106.27 |  |  |  |  |  |  |
|  |  |  | Inverse-variance weighted (fixed) | 12 | 1.23 | 1.08-1.40 | 0.002 | 0.257 |
|  |  |  | Weighted median | 12 | 1.27 | 1.05-1.54 | 0.015 |  |
|  |  |  | MR-PRESSO test | 12 | 1.23 | 1.07-1.42 | 0.017 |  |
|  |  |  | MR-Egger | 12 | / | / | 0.618* |  |
| Parabacteroides | 1.47% | 27.38 |  |  |  |  |  |  |
|  |  |  | Inverse-variance weighted (fixed) | 10 | 0.94 | 0.72-1.23 | 0.639 | 0.854 |
|  |  |  | Weighted median | 10 | 0.97 | 0.67-1.39 | 0.852 |  |
|  |  |  | MR-PRESSO test | 10 | 0.94 | 0.77-1.14 | 0.539 |  |
|  |  |  | MR-Egger | 10 | / | / | 0.127* |  |
| Paraprevotella | 4.40% | 64.84 |  |  |  |  |  |  |
|  |  |  | Inverse-variance weighted (fixed) | 13 | 0.97 | 0.83-1.13 | 0.694 | 0.343 |
|  |  |  | Weighted median | 13 | 0.87 | 0.70-1.08 | 0.215 |  |
|  |  |  | MR-PRESSO test | 13 | 0.97 | 0.82-1.14 | 0.716 |  |
|  |  |  | MR-Egger | 13 | / | / | 0.705* |  |
| Parasutterella | 3.69% | 41.32 |  |  |  |  |  |  |
|  |  |  | Inverse-variance weighted (fixed) | 17 | 1.22 | 1.03-1.45 | 0.023 | 0.676 |
|  |  |  | Weighted median | 17 | 1.12 | 0.89-1.41 | 0.343 |  |
|  |  |  | MR-PRESSO test | 17 | 1.22 | 1.05-1.42 | 0.023 |  |
|  |  |  | MR-Egger | 17 | / | / | 0.610* |  |
| Peptococcus | 6.62% | 81.14 |  |  |  |  |  |  |
|  |  |  | Inverse-variance weighted (fixed) | 16 | 1.07 | 0.94-1.23 | 0.280 | 0.786 |
|  |  |  | Weighted median | 16 | 1.05 | 0.88-1.25 | 0.612 |  |
|  |  |  | MR-PRESSO test | 16 | 1.07 | 0.96-1.20 | 0.217 |  |
|  |  |  | MR-Egger | 16 | / | / | 0.588* |  |
| Phascolarctobacterium | 2.71% | 39.22 |  |  |  |  |  |  |
|  |  |  | Inverse-variance weighted (fixed) | 13 | 1.04 | 0.85-1.28 | 0.680 | 0.238 |
|  |  |  | Weighted median | 13 | 1.22 | 0.93-1.61 | 0.150 |  |
|  |  |  | MR-PRESSO test | 13 | 1.04 | 0.83-1.31 | 0.719 |  |
|  |  |  | MR-Egger | 13 | / | / | 0.706* |  |
| Prevotella7 | 7.34% | 121.04 |  |  |  |  |  |  |
|  |  |  | Inverse-variance weighted (fixed) | 12 | 1.02 | 0.90-1.15 | 0.757 | 0.564 |
|  |  |  | Weighted median | 12 | 1.03 | 0.87-1.22 | 0.746 |  |
|  |  |  | MR-PRESSO test | 12 | 1.02 | 0.91-1.15 | 0.747 |  |
|  | 5.00% | 48.25 | MR-Egger | 12 | / | / | 0.369* |  |
| Prevotella9 |  |  |  |  |  |  |  |  |
|  |  |  | Inverse-variance weighted (fixed) | 20 | 1.04 | 0.89-1.21 | 0.634 | 0.182 |
|  |  |  | Weighted median | 20 | 0.92 | 0.74-1.16 | 0.500 |  |
|  |  |  | MR-PRESSO test | 20 | 1.04 | 0.87-1.23 | 0.700 |  |
|  |  |  | MR-Egger | 20 | / | / | 0.100* |  |
| RikenellaceaeRC9 | 9.72% | 131.46 |  |  |  |  |  |  |
|  |  |  | Inverse-variance weighted (fixed) | 15 | 1.15 | 1.03-1.28 | 0.011 | 0.382 |
|  |  |  | Weighted median | 15 | 1.12 | 0.96-1.31 | 0.152 |  |
|  |  |  | MR-PRESSO test | 15 | 1.15 | 1.03-1.29 | 0.028 |  |
|  |  |  | MR-Egger | 15 | / | / | 0.144* |  |
| Romboutsia | 2.87% | 36.08 |  |  |  |  |  |  |
|  |  |  | Inverse-variance weighted (fixed) | 15 | 1.02 | 0.80-1.31 | 0.865 | 0.114 |
|  |  |  | Weighted median | 15 | 1.06 | 0.79-1.41 | 0.702 |  |
|  |  |  | MR-PRESSO test | 15 | 1.02 | 0.80-1.31 | 0.867 |  |
|  |  |  | MR-Egger | 15 | / | / | 0.492* |  |
| Roseburia | 2.57% | 26.88 |  |  |  |  |  |  |
|  |  |  | Inverse-variance weighted (fixed) | 18 | 0.98 | 0.83-1.25 | 0.836 | 0.312 |
|  |  |  | Weighted median | 18 | 1.17 | 0.87-1.56 | 0.299 |  |
|  |  |  | MR-PRESSO test | 18 | 0.98 | 0.78-1.23 | 0.861 |  |
|  |  |  | MR-Egger | 18 | / | / | 0.165* |  |
| Ruminiclostridium5 | 2.06% | 25.73 |  |  |  |  |  |  |
|  |  |  | Inverse-variance weighted (random) | 15 | 0.86 | 0.59-1.24 | 0.406 | 0.001 |
|  |  |  | Weighted median | 15 | 0.89 | 0.64-1.26 | 0.521 |  |
|  |  |  | MR-PRESSO test | 15 | 0.86 | 0.59-1.24 | 0.419 |  |
|  |  |  | Outlier corrected (MR-PRESSO) | 14 | 0.97 | 0.72-1.30 | 0.843 |  |
|  |  |  | MR-Egger | 15 | / | / | 0.619* |  |
| Ruminiclostridium6 | 3.28% | 36.50 |  |  |  |  |  |  |
|  |  |  | Inverse-variance weighted (random) | 17 | 1.00 | 0.72-1.37 | 0.989 | 8.48E-05 |
|  |  |  | Weighted median | 17 | 0.88 | 0.68-1.15 | 0.346 |  |
|  |  |  | MR-PRESSO test | 17 | 1.00 | 0.72-1.37 | 0.989 |  |
|  |  |  | Outlier corrected (MR-PRESSO) | 16 | 0.88 | 0.73-1.06 | 0.203 |  |
|  |  |  | MR-Egger | 17 | / | / | 0.098* |  |
| Ruminiclostridium9 | 2.06% | 25.75 |  |  |  |  |  |  |
|  |  |  | Inverse-variance weighted (fixed) | 15 | 1.04 | 0.83-1.31 | 0.732 | 0.174 |
|  |  |  | Weighted median | 15 | 1.14 | 0.81-1.60 | 0.458 |  |
|  |  |  | MR-PRESSO test | 15 | 1.04 | 0.80-1.36 | 0.772 |  |
|  |  |  | MR-Egger | 15 | / | / | 0.798* |  |
| RuminococcaceaeNK4A214 | 2.76% | 28.93 |  |  |  |  |  |  |
|  |  |  | Inverse-variance weighted (fixed) | 18 | 0.85 | 0.70-1.14 | 0.117 | 0.226 |
|  |  |  | Weighted median | 18 | 0.89 | 0.67-1.19 | 0.428 |  |
|  |  |  | MR-PRESSO test | 18 | 0.85 | 0.68-1.06 | 0.176 |  |
|  |  |  | MR-Egger | 18 | / | / | 0.380* |  |
| RuminococcaceaeUCG002 | 3.86% | 28.25 |  |  |  |  |  |  |
|  |  |  | Inverse-variance weighted (fixed) | 26 | 0.96 | 0.81-1.14 | 0.649 | 0.626 |
|  |  |  | Weighted median | 26 | 0.98 | 0.77-1.25 | 0.868 |  |
|  |  |  | MR-PRESSO test | 26 | 0.96 | 0.82-1.13 | 0.633 |  |
|  |  |  | MR-Egger | 26 | / | / | 0.038* |  |
| RuminococcaceaeUCG003 | 2.28% | 30.60 |  |  |  |  |  |  |
|  |  |  | Inverse-variance weighted (fixed) | 14 | 1.02 | 0.67-1.56 | 0.921 | 3.29E-06 |
|  |  |  | Weighted median | 14 | 0.86 | 0.62-1.18 | 0.354 |  |
|  |  |  | MR-PRESSO test | 14 | 1.02 | 0.67-1.56 | 0.923 |  |
|  |  |  | Outlier corrected (MR-PRESSO) | 13 | 0.85 | 0.67-1.07 | 0.194 |  |
|  |  |  | MR-Egger | 14 | / | / | 0.337* |  |
| RuminococcaceaeUCG004 | 2.57% | 43.94 |  |  |  |  |  |  |
|  |  |  | Inverse-variance weighted (fixed) | 11 | 1.00 | 0.82-1.23 | 0.980 | 0.877 |
|  |  |  | Weighted median | 11 | 1.05 | 0.80-1.38 | 0.708 |  |
|  |  |  | MR-PRESSO test | 11 | 1.00 | 0.87-1.16 | 0.973 |  |
|  |  |  | MR-Egger | 11 | / | / | 0.890* |  |
| RuminococcaceaeUCG005 | 2.73% | 30.20 |  |  |  |  |  |  |
|  |  |  | Inverse-variance weighted (fixed) | 17 | 1.04 | 0.85-1.27 | 0.707 | 0.610 |
|  |  |  | Weighted median | 17 | 1.08 | 0.83-1.40 | 0.559 |  |
|  |  |  | MR-PRESSO test | 17 | 1.04 | 0.86-1.25 | 0.691 |  |
|  |  |  | MR-Egger | 17 | / | / | 0.723* |  |
| RuminococcaceaeUCG009 | 4.30% | 59.42 |  |  |  |  |  |  |
|  |  |  | Inverse-variance weighted (fixed) | 13 | 0.81 | 0.69-0.96 | 0.014 | 0.559 |
|  |  |  | Weighted median | 13 | 0.83 | 0.66-1.05 | 0.100 |  |
|  |  |  | MR-PRESSO test | 13 | 0.81 | 0.69-0.95 | 0.023 |  |
|  |  |  | MR-Egger | 13 | / | / | 0.516* |  |
| RuminococcaceaeUCG010 | 1.51% | 35.03 |  |  |  |  |  |  |
|  |  |  | Inverse-variance weighted (fixed) | 8 | 1.17 | 0.89-1.54 | 0.274 | 0.423 |
|  |  |  | Weighted median | 8 | 1.11 | 0.76-1.62 | 0.593 |  |
|  |  |  | MR-PRESSO test | 8 | 1.17 | 0.88-1.54 | 0.312 |  |
|  |  |  | MR-Egger | 8 | / | / | 0.318* |  |
| RuminococcaceaeUCG011 | 4.90% | 118.09 |  |  |  |  |  |  |
|  |  |  | Inverse-variance weighted (fixed) | 8 | 1.00 | 0.87-1.16 | 0.995 | 0.594 |
|  |  |  | Weighted median | 8 | 0.96 | 0.80-1.15 | 0.660 |  |
|  |  |  | MR-PRESSO test | 8 | 1.00 | 0.88-1.14 | 0.995 |  |
|  |  |  | MR-Egger | 8 | / | / | 0.932* |  |
| RuminococcaceaeUCG013 | 2.09% | 26.01 |  |  |  |  |  |  |
|  |  |  | Inverse-variance weighted (fixed) | 15 | 0.91 | 0.73-1.14 | 0.397 | 0.555 |
|  |  |  | Weighted median | 15 | 1.12 | 0.82-1.53 | 0.465 |  |
|  |  |  | MR-PRESSO test | 15 | 0.91 | 0.73-1.12 | 0.387 |  |
|  |  |  | MR-Egger | 15 | / | / | 0.141* |  |
| RuminococcaceaeUCG014 | 2.88% | 31.95 |  |  |  |  |  |  |
|  |  |  | Inverse-variance weighted (fixed) | 17 | 1.32 | 1.08-1.61 | 0.007 | 0.602 |
|  |  |  | Weighted median | 17 | 1.42 | 1.08-1.86 | 0.012 |  |
|  |  |  | MR-PRESSO test | 17 | 1.32 | 1.09-1.58 | 0.010 |  |
|  |  |  | MR-Egger | 17 | / | / | 0.800* |  |
| Ruminococcus1 | 2.14% | 28.60 |  |  |  |  |  |  |
|  |  |  | Inverse-variance weighted (fixed) | 14 | 1.02 | 0.81-1.29 | 0.869 | 0.299 |
|  |  |  | Weighted median | 14 | 1.03 | 0.74-1.45 | 0.855 |  |
|  |  |  | MR-PRESSO test | 14 | 1.02 | 0.79-1.31 | 0.881 |  |
|  |  |  | MR-Egger | 14 | / | / | 0.962* |  |
| Ruminococcus2 | 2.76% | 34.63 |  |  |  |  |  |  |
|  |  |  | Inverse-variance weighted (random) | 15 | 1.05 | 0.85-1.30 | 0.669 | 0.552 |
|  |  |  | Weighted median | 15 | 1.01 | 0.75-1.36 | 0.936 |  |
|  |  |  | MR-PRESSO test | 15 | 1.05 | 0.85-1.29 | 0.660 |  |
|  |  |  | MR-Egger | 15 | / | / | 0.577* |  |
| Sellimonas | 9.32% | 144.84 |  |  |  |  |  |  |
|  |  |  | Inverse-variance weighted (fixed) | 13 | 0.95 | 0.85-1.06 | 0.381 | 0.071 |
|  |  |  | Weighted median | 13 | 0.93 | 0.79-1.09 | 0.385 |  |
|  |  |  | MR-PRESSO test | 13 | 0.95 | 0.83-1.10 | 0.507 |  |
|  |  |  | MR-Egger | 13 | / | / | 0.214* |  |
| Senegalimassilia | 2.58% | 60.59 |  |  |  |  |  |  |
|  |  |  | Inverse-variance weighted (fixed) | 8 | 1.04 | 0.85-1.27 | 0.721 | 0.089 |
|  |  |  | Weighted median | 8 | 0.96 | 0.73-1.25 | 0.757 |  |
|  |  |  | MR-PRESSO test | 8 | 1.04 | 0.79-1.35 | 0.796 |  |
|  |  |  | MR-Egger | 8 | / | / | 0.339* |  |
| Slackia | 2.81% | 66.15 |  |  |  |  |  |  |
|  |  |  | Inverse-variance weighted (fixed) | 8 | 1.06 | 0.87-1.28 | 0.568 | 0.405 |
|  |  |  | Weighted median | 8 | 1.03 | 0.80-1.32 | 0.844 |  |
|  |  |  | MR-PRESSO test | 8 | 1.06 | 0.87-1.29 | 0.592 |  |
|  |  |  | MR-Egger | 8 | / | / | 0.956* |  |
| Streptococcus | 2.68% | 26.56 |  |  |  |  |  |  |
|  |  |  | Inverse-variance weighted (fixed) | 19 | 0.88 | 0.72-1.08 | 0.217 | 0.417 |
|  |  |  | Weighted median | 19 | 0.76 | 0.57-1.03 | 0.073 |  |
|  |  |  | MR-PRESSO test | 19 | 0.88 | 0.72-1.08 | 0.240 |  |
|  |  |  | MR-Egger | 19 | / | / | 0.630* |  |
| Subdoligranulum | 1.86% | 24.81 |  |  |  |  |  |  |
|  |  |  | Inverse-variance weighted (fixed) | 14 | 1.09 | 0.86-1.38 | 0.490 | 0.803 |
|  |  |  | Weighted median | 14 | 1.06 | 0.77-1.45 | 0.720 |  |
|  |  |  | MR-PRESSO test | 14 | 1.09 | 0.90-1.32 | 0.412 |  |
|  |  |  | MR-Egger | 14 | / | / | 0.524* |  |
| Sutterella | 1.97% | 30.70 |  |  |  |  |  |  |
|  |  |  | Inverse-variance weighted (fixed) | 12 | 0.98 | 0.78-1.23 | 0.868 | 0.655 |
|  |  |  | Weighted median | 12 | 1.02 | 0.76-1.37 | 0.910 |  |
|  |  |  | MR-PRESSO test | 12 | 0.98 | 0.80-1.20 | 0.855 |  |
|  |  |  | MR-Egger | 12 | / | / | 0.748* |  |
| Terrisporobacter | 2.14% | 66.94 |  |  |  |  |  |  |
|  |  |  | Inverse-variance weighted (fixed) | 6 | 0.94 | 0.76-1.18 | 0.605 | 0.818 |
|  |  |  | Weighted median | 6 | 0.93 | 0.71-1.21 | 0.589 |  |
|  |  |  | MR-PRESSO test | 6 | 0.94 | 0.82-1.09 | 0.473 |  |
|  |  |  | MR-Egger | 6 | / | / | 0.979* |  |
| Turicibacter | 3.35% | 45.43 |  |  |  |  |  |  |
|  |  |  | Inverse-variance weighted (fixed) | 14 | 1.13 | 0.95-1.35 | 0.180 | 0.420 |
|  |  |  | Weighted median | 14 | 1.12 | 0.88-1.42 | 0.373 |  |
|  |  |  | MR-PRESSO test | 14 | 1.13 | 0.94-1.35 | 0.209 |  |
|  |  |  | MR-Egger | 14 | / | / | 0.011* |  |
| Tyzzerella3 | 6.44% | 90.12 |  |  |  |  |  |  |
|  |  |  | Inverse-variance weighted (fixed) | 14 | 0.99 | 0.86-1.13 | 0.865 | 0.447 |
|  |  |  | Weighted median | 14 | 0.98 | 0.81-1.19 | 0.853 |  |
|  |  |  | MR-PRESSO test | 14 | 0.99 | 0.86-1.13 | 0.867 |  |
|  |  |  | MR-Egger | 14 | / | / | 0.028* |  |
| Veillonella | 2.77% | 47.44 |  |  |  |  |  |  |
|  |  |  | Inverse-variance weighted (fixed) | 11 | 0.95 | 0.78-1.17 | 0.637 | 0.382 |
|  |  |  | Weighted median | 11 | 0.91 | 0.70-1.19 | 0.512 |  |
|  |  |  | MR-PRESSO test | 11 | 0.95 | 0.77-1.18 | 0.658 |  |
|  |  |  | MR-Egger | 11 | / | / | 0.903* |  |
| Victivallis | 7.29% | 130.96 |  |  |  |  |  |  |
|  |  |  | Inverse-variance weighted (fixed) | 11 | 1.00 | 0.89-1.13 | 0.948 | 0.963 |
|  |  |  | Weighted median | 11 | 0.96 | 0.83-1.12 | 0.619 |  |
|  |  |  | MR-PRESSO test | 11 | 1.00 | 0.93-1.08 | 0.916 |  |
|  |  |  | MR-Egger | 11 | / | / | 0.542* |  |
| Veillonella | 2.77% | 47.44 |  |  |  |  |  |  |
|  |  |  | Inverse-variance weighted (fixed) | 11 | 0.98 | 0.92-1.04 | 0.469 | 0.468 |
|  |  |  | Weighted median | 11 | 0.98 | 0.90-1.06 | 0.544 |  |
|  |  |  | MR-PRESSO test | 11 | 0.98 | 0.92-1.04 | 0.479 |  |
|  |  |  | MR-Egger | 11 | / | / | 0.561* |  |
| Victivallis | 7.29% | 130.96 |  |  |  |  |  |  |
|  |  |  | Inverse-variance weighted (fixed) | 13 | 1.00 | 0.97-1.03 | 0.944 | 0.105 |
|  |  |  | Weighted median | 13 | 1.03 | 0.98-1.08 | 0.291 |  |
|  |  |  | MR-PRESSO test | 13 | 1.00 | 0.96-1.04 | 0.956 |  |
|  |  |  | MR-Egger | 13 | / | / | 0.516* |  |

abbreviations: CI, confidence interval; MR, Mendelian randomization; MR-PRESSO test, MR Pleiotropy RESidual Sum and Outlier test; OR, odds ratio; SNP, single nucleotide polymorphism. *P-value of the intercept from MR-Egger regression analysis.

**Supplementary Table 5** Characteristics of the genetic variants associated with six bacterial that have been identified to be associated with the risk of IBD, UC and CD.

| Gut microbiome | SNP | Chr | Position | Effect allele | Beta | SE | *P*-value |
| --- | --- | --- | --- | --- | --- | --- | --- |
| Genus *Eubacteriumhalliigroup* | rs10798999 | 1 | 34308917 | C | 0.060 | 0.013 | 2.61E-06 |
| Genus *Eubacteriumhalliigroup* | rs17474256 | 1 | 104524676 | G | 0.081 | 0.018 | 9.45E-06 |
| Genus *Eubacteriumhalliigroup* | rs138531890 | 3 | 195238086 | A | 0.153 | 0.035 | 5.43E-06 |
| Genus *Eubacteriumhalliigroup* | rs28584818 | 3 | 64664456 | A | 0.126 | 0.027 | 4.43E-06 |
| Genus *Eubacteriumhalliigroup* | rs6550770 | 3 | 23663416 | T | -0.198 | 0.044 | 4.82E-06 |
| Genus *Eubacteriumhalliigroup* | rs949971 | 3 | 110283389 | T | -0.054 | 0.012 | 3.29E-06 |
| Genus *Eubacteriumhalliigroup* | rs13116360 | 4 | 111885431 | T | 0.154 | 0.030 | 2.94E-07 |
| Genus *Eubacteriumhalliigroup* | rs17074066 | 4 | 183709536 | T | -0.081 | 0.019 | 9.35E-06 |
| Genus *Eubacteriumhalliigroup* | rs10808115 | 7 | 100635375 | A | -0.050 | 0.011 | 4.42E-06 |
| Genus *Eubacteriumhalliigroup* | rs60254196 | 7 | 148856720 | A | -0.052 | 0.011 | 2.70E-06 |
| Genus *Eubacteriumhalliigroup* | rs10501370 | 11 | 58040621 | C | -0.116 | 0.025 | 5.42E-06 |
| Genus *Eubacteriumhalliigroup* | rs117748144 | 11 | 11771637 | T | -0.127 | 0.029 | 7.86E-06 |
| Genus *Eubacteriumhalliigroup* | rs78056098 | 11 | 123789877 | G | -0.051 | 0.011 | 8.29E-06 |
| Genus *Eubacteriumhalliigroup* | rs74018587 | 15 | 62014160 | C | 0.209 | 0.044 | 3.70E-06 |
| Genus *Eubacteriumhalliigroup* | rs630939 | 18 | 48384463 | C | -0.051 | 0.011 | 9.16E-06 |
| Genus *Eubacteriumhalliigroup* | rs281379 | 19 | 49214274 | A | -0.050 | 0.011 | 9.33E-06 |
| Genus *Coprococcus2* | rs61823518 | 1 | 223688236 | A | -0.096 | 0.022 | 6.68E-06 |
| Genus *Coprococcus2* | rs6677933 | 1 | 112139008 | C | -0.080 | 0.016 | 1.19E-06 |
| Genus *Coprococcus2* | rs9426473 | 1 | 4210455 | A | 0.073 | 0.016 | 6.31E-06 |
| Genus *Coprococcus2* | rs12634070 | 3 | 180544880 | T | 0.074 | 0.016 | 9.95E-06 |
| Genus *Coprococcus2* | rs72680320 | 4 | 131125786 | T | -0.065 | 0.014 | 2.27E-06 |
| Genus *Coprococcus2* | rs10070053 | 5 | 34794789 | A | 0.059 | 0.014 | 7.65E-06 |
| Genus *Coprococcus2* | rs6894272 | 5 | 33114638 | T | -0.113 | 0.025 | 9.53E-06 |
| Genus *Coprococcus2* | rs10121347 | 9 | 16806694 | C | 0.093 | 0.022 | 8.31E-06 |
| Genus *Coprococcus2* | rs2482516 | 9 | 25554068 | C | 0.075 | 0.016 | 4.72E-06 |
| Genus *Coprococcus2* | rs35890118 | 10 | 129715759 | A | -0.067 | 0.015 | 8.26E-06 |
| Genus *Coprococcus2* | rs1958519 | 14 | 86833255 | T | 0.067 | 0.014 | 1.58E-06 |
| Genus *Coprococcus2* | rs59936925 | 20 | 49266770 | A | 0.117 | 0.023 | 9.38E-07 |
| Genus *Enterorhabdus* | rs114731706 | 2 | 19611953 | T | 0.182 | 0.038 | 2.17E-06 |
| Genus *Enterorhabdus* | rs77655283 | 2 | 236439879 | G | 0.133 | 0.030 | 5.88E-06 |
| Genus *Enterorhabdus* | rs11098863 | 4 | 127336602 | T | -0.097 | 0.016 | 3.06E-09 |
| Genus *Enterorhabdus* | rs9470637 | 6 | 37567189 | A | -0.076 | 0.017 | 5.70E-06 |
| Genus *Enterorhabdus* | rs2051957 | 7 | 89709883 | C | 0.084 | 0.019 | 8.90E-06 |
| Genus *Enterorhabdus* | rs10098492 | 8 | 113109756 | T | 0.132 | 0.029 | 6.41E-06 |
| Genus *Enterorhabdus* | rs7923280 | 10 | 20280612 | A | 0.086 | 0.017 | 5.24E-07 |
| Genus *Enterorhabdus* | rs3017103 | 11 | 62174193 | A | 0.098 | 0.021 | 2.94E-06 |
| Genus *Enterorhabdus* | rs73331712 | 12 | 69515102 | T | 0.262 | 0.055 | 4.85E-06 |
| Genus *Enterorhabdus* | rs424715 | 19 | 35863028 | T | 0.082 | 0.017 | 4.41E-06 |
| Genus *LachnospiraceaeUCG001* | rs12131224 | 1 | 166199362 | C | 0.117 | 0.026 | 7.40E-06 |
| Genus *LachnospiraceaeUCG001* | rs2050911 | 1 | 82384407 | G | 0.075 | 0.015 | 1.11E-06 |
| Genus *LachnospiraceaeUCG001* | rs78848836 | 1 | 53773248 | A | -0.119 | 0.026 | 3.38E-06 |
| Genus *LachnospiraceaeUCG001* | rs79476906 | 1 | 108102470 | T | -0.087 | 0.020 | 8.27E-06 |
| Genus *LachnospiraceaeUCG001* | rs437876 | 3 | 42568440 | T | 0.078 | 0.014 | 7.17E-08 |
| Genus *LachnospiraceaeUCG001* | rs985416 | 3 | 148269083 | C | 0.097 | 0.018 | 1.46E-07 |
| Genus *LachnospiraceaeUCG001* | rs9403580 | 6 | 145006780 | C | 0.108 | 0.023 | 3.47E-06 |
| Genus *LachnospiraceaeUCG001* | rs62496417 | 7 | 96965504 | T | -0.075 | 0.017 | 5.88E-06 |
| Genus *LachnospiraceaeUCG001* | rs7341608 | 8 | 56839500 | T | -0.078 | 0.018 | 9.48E-06 |
| Genus *LachnospiraceaeUCG001* | rs10815577 | 9 | 7273249 | C | -0.068 | 0.014 | 1.72E-06 |
| Genus *LachnospiraceaeUCG001* | rs573933 | 9 | 14477851 | T | -0.108 | 0.023 | 3.11E-06 |
| Genus *LachnospiraceaeUCG001* | rs2371284 | 12 | 56256262 | T | -0.076 | 0.017 | 7.56E-06 |
| Genus *LachnospiraceaeUCG001* | rs4981345 | 14 | 21455973 | T | -0.068 | 0.015 | 6.09E-06 |
| Genus *LachnospiraceaeUCG001* | rs74034332 | 16 | 79092359 | G | 0.168 | 0.038 | 3.33E-06 |
| Genus *LachnospiraceaeUCG001* | rs7213933 | 17 | 36155074 | T | -0.082 | 0.018 | 9.02E-06 |
| Genus *LachnospiraceaeUCG001* | rs8104225 | 19 | 13962924 | A | 0.089 | 0.020 | 8.04E-06 |
| Genus *Oxalobacter* | rs4428215 | 3 | 171947435 | G | 0.130 | 0.024 | 7.51E-08 |
| Genus *Oxalobacter* | rs36057338 | 4 | 189935314 | G | 0.208 | 0.042 | 8.80E-07 |
| Genus *Oxalobacter* | rs1569853 | 6 | 38550301 | T | -0.138 | 0.030 | 3.65E-06 |
| Genus *Oxalobacter* | rs10464997 | 8 | 20902693 | G | 0.138 | 0.029 | 3.30E-06 |
| Genus *Oxalobacter* | rs6993398 | 8 | 115560689 | G | 0.127 | 0.028 | 7.13E-06 |
| Genus *Oxalobacter* | rs12002250 | 9 | 19682558 | A | 0.217 | 0.047 | 1.42E-06 |
| Genus *Oxalobacter* | rs736744 | 9 | 87514407 | C | 0.118 | 0.021 | 2.57E-08 |
| Genus *Oxalobacter* | rs3862635 | 11 | 126582578 | C | -0.172 | 0.039 | 9.19E-06 |
| Genus *Oxalobacter* | rs11108500 | 12 | 96819204 | A | -0.199 | 0.043 | 3.74E-06 |
| Genus *Oxalobacter* | rs111966731 | 15 | 93941937 | T | 0.213 | 0.047 | 7.30E-06 |
| Genus *Oxalobacter* | rs6071435 | 20 | 59502888 | T | -0.106 | 0.021 | 1.07E-06 |
| Genus *Oxalobacter* | rs6000536 | 22 | 37421469 | C | -0.131 | 0.025 | 2.06E-07 |
| Genus *RuminococcaceaeUCG014* | rs10495392 | 1 | 237422989 | C | -0.082 | 0.019 | 9.96E-06 |
| Genus *RuminococcaceaeUCG014* | rs74060145 | 1 | 15322582 | C | -0.116 | 0.025 | 8.71E-06 |
| Genus *RuminococcaceaeUCG014* | rs439810 | 2 | 119467367 | G | -0.058 | 0.013 | 7.04E-06 |
| Genus *RuminococcaceaeUCG014* | rs72809222 | 2 | 57205854 | T | 0.067 | 0.014 | 2.41E-06 |
| Genus *RuminococcaceaeUCG014* | rs995642 | 2 | 134854659 | C | 0.060 | 0.013 | 1.90E-06 |
| Genus *RuminococcaceaeUCG014* | rs12638134 | 3 | 101334609 | T | 0.058 | 0.012 | 1.21E-06 |
| Genus *RuminococcaceaeUCG014* | rs73186226 | 3 | 123829361 | G | -0.099 | 0.022 | 6.72E-06 |
| Genus *RuminococcaceaeUCG014* | rs10941294 | 5 | 36435597 | C | -0.122 | 0.026 | 2.40E-06 |
| Genus *RuminococcaceaeUCG014* | rs115777838 | 5 | 26110626 | T | -0.188 | 0.039 | 4.62E-07 |
| Genus *RuminococcaceaeUCG014* | rs62478832 | 7 | 109564034 | T | -0.058 | 0.013 | 6.04E-06 |
| Genus *RuminococcaceaeUCG014* | rs34402072 | 8 | 3636548 | C | -0.069 | 0.016 | 9.80E-06 |
| Genus *RuminococcaceaeUCG014* | rs56105232 | 9 | 14363769 | G | 0.139 | 0.030 | 2.91E-06 |
| Genus *RuminococcaceaeUCG014* | rs853612 | 10 | 119928413 | A | -0.053 | 0.012 | 9.75E-06 |
| Genus *RuminococcaceaeUCG014* | rs10791168 | 11 | 131659000 | A | -0.066 | 0.015 | 9.76E-06 |
| Genus *RuminococcaceaeUCG014* | rs61898819 | 11 | 126024042 | A | 0.061 | 0.014 | 9.92E-06 |
| Genus *RuminococcaceaeUCG014* | rs77627087 | 16 | 87759750 | C | 0.068 | 0.015 | 7.43E-06 |
| Genus *RuminococcaceaeUCG014* | rs79640386 | 18 | 4084336 | T | -0.111 | 0.025 | 8.74E-06 |
| Genus *RuminococcaceaeUCG014* | rs17296933 | 19 | 49175964 | C | -0.083 | 0.019 | 7.34E-06 |

Abbreviations: Chr, chromosome; SE, standard error; SNP, single nucleotide polymorphism.

**Supplementary Table 6** Effect estimates of the associations between IBD, UC, CD and risk of six bacterial traits in the MR analyses.

| Exposure | Gut microbiota | Number of SNPs | Methods | OR (95% CI) | *P* value |  |
| --- | --- | --- | --- | --- | --- | --- |
|  |  |  |  |  |  |  |
| IBD |  |  |  |  |  |  |
|  | Genus *Eubacteriumhalliigroup* |  |  |  |  |  |
|  |  | 122 | Inverse-variance weighted | 0.98(0.96-1.00) | 0.078 |  |
|  |  | 122 | Weighted median | 0.99(0.95-1.02) | 0.447 |  |
|  |  | 122 | MR-PRESSO test | 0.98(0.95-1.00) | 0.116 |  |
|  |  | 122 | MR-Egger | \ | 0.352* |  |
|  | Genus *Coprococcus2* |  |  |  |  |  |
|  |  | 121 | Inverse-variance weighted | 1.02(0.99-1.04) | 0.156 |  |
|  |  | 121 | Weighted median | 1.02(0.98-1.06) | 0.430 |  |
|  |  | 121 | MR-PRESSO test | 1.02(0.99-1.04) | 0.167 |  |
|  |  | 121 | MR-Egger | \ | 0.684* |  |
|  | Genus *Enterorhabdus* |  |  |  |  |  |
|  |  | 121 | Inverse-variance weighted | 0.98(0.95-1.01) | 0.238 |  |
|  |  | 121 | Weighted median | 1.01(0.97-1.07) | 0.564 |  |
|  |  | 121 | MR-PRESSO test | 0.98(0.95-1.01) | 0.243 |  |
|  |  | 121 | MR-Egger | \ | 0.503* |  |
|  | Genus *LachnospiraceaeUCG001* |  |  |  |  |  |
|  |  | 121 | Inverse-variance weighted | 1.00(0.97-1.02) | 0.849 |  |
|  |  | 121 | Weighted median | 1.01(0.97-1.06) | 0.538 |  |
|  |  | 121 | MR-PRESSO test | 1.00(0.97-1.03) | 0.860 |  |
|  |  | 121 | MR-Egger |  | 0.174* |  |
|  | Genus *Oxalobacter* |  |  |  |  |  |
|  |  | 121 | Inverse-variance weighted | 1.01(0.97-1.05) | 0.659 |  |
|  |  | 121 | Weighted median | 1.05(0.98-1.12) | 0.178 |  |
|  |  | 121 | MR-PRESSO test | 1.01(0.97-1.05) | 0.656 |  |
|  |  | 121 | MR-Egger | \ | 0.634* |  |
|  | Genus *RuminococcaceaeUCG014* |  |  |  |  |  |
|  |  | 122 | Inverse-variance weighted | 1.00(0.98-1.02) | 0.942 |  |
|  |  | 122 | Weighted median | 1.02(0.98-1.06) | 0.287 |  |
|  |  | 121 | MR-PRESSO test | 1.00(0.98-1.02) | 0.942 |  |
|  |  | 122 | MR-Egger | \ | 0.799* |  |
| UC |  |  |  |  |  |  |
|  | Genus *Eubacteriumhalliigroup* |  |  |  |  |  |
|  |  | 80 | Inverse-variance weighted | 0.99(0.96-1.01) | 0.295 |  |
|  |  | 80 | Weighted median | 0.98(0.95-1.02) | 0.379 |  |
|  |  | 80 | MR-PRESSO test | 0.99(0.96-1.01) | 0.298 |  |
|  |  | 80 | MR-Egger | \ | 0.666* |  |
|  | Genus *Coprococcus2* |  |  |  |  |  |
|  |  | 80 | Inverse-variance weighted | 1.01(0.99-1.04) | 0.378 |  |
|  |  | 80 | Weighted median | 1.01(0.97-1.05) | 0.680 |  |
|  |  | 80 | MR-PRESSO test | 1.01(0.98-1.04) | 0.397 |  |
|  |  | 80 | MR-Egger | \ | 0.731* |  |
|  | Genus *Enterorhabdus* |  |  |  |  |  |
|  |  | 79 | Inverse-variance weighted | 0.97(0.94-1.00) | 0.084 |  |
|  |  | 79 | Weighted median | 1.01(0.97-1.07) | 0.564 |  |
|  |  | 79 | MR-PRESSO test | 0.97(0.94-1.00) | 0.065 |  |
|  |  | 79 | MR-Egger | \ | 0.300* |  |
|  | Genus *LachnospiraceaeUCG001* |  |  |  |  |  |
|  |  | 80 | Inverse-variance weighted | 0.99(0.96-1.02) | 0.562 |  |
|  |  | 80 | Weighted median | 0.99(0.95-1.03) | 0.653 |  |
|  |  | 80 | MR-PRESSO test | 0.99(0.96-1.02) | 0.565 |  |
|  |  | 80 | MR-Egger |  | 0.901* |  |
|  | Genus *Oxalobacter* |  |  |  |  |  |
|  |  | 79 | Inverse-variance weighted | 0.98(0.94-1.03) | 0.452 |  |
|  |  | 79 | Weighted median | 1.02(0.96-1.09) | 0.565 |  |
|  |  | 79 | MR-PRESSO test | 0.98(0.94-1.03) | 0.459 |  |
|  |  | 79 | MR-Egger |  | 0.966 |  |
|  | Genus *RuminococcaceaeUCG014* |  |  |  |  |  |
|  |  | 80 | Inverse-variance weighted | 1.02(0.99-1.05) | 0.152 |  |
|  |  | 80 | Weighted median | 1.03(0.99-1.07) | 0.128 |  |
|  |  | 80 | MR-PRESSO test | 1.02(0.99-1.050 | 0.156 |  |
|  |  | 80 | MR-Egger | \ | 0.429* |  |
| CD |  |  |  |  |  |  |
|  | Genus *Eubacteriumhalliigroup* |  |  |  |  |  |
|  |  | 109 | Inverse-variance weighted | 0.99(0.97-1.01) | 0.381 |  |
|  |  | 109 | Weighted median | 0.99(0.96-1.02) | 0.543 |  |
|  |  | 109 | MR-PRESSO test | 0.99(0.96-1.01) | 0.383 |  |
|  |  | 109 | MR-Egger | \ | 0.194* |  |
|  | Genus *Coprococcus2* |  |  |  |  |  |
|  |  | 109 | Inverse-variance weighted | 1.02(0.99-1.04) | 0.147 |  |
|  |  | 109 | Weighted median | 1.02(0.98-1.05) | 0.343 |  |
|  |  | 109 | MR-PRESSO test | 1.02(0.99-1.04) | 0.153 |  |
|  |  | 109 | MR-Egger | \ | 0.126* |  |
|  | Genus *Enterorhabdus* |  |  |  |  |  |
|  |  | 109 | Inverse-variance weighted | 0.99(0.97-1.02) | 0.567 |  |
|  |  | 109 | Weighted median | 1.02(0.98-1.07) | 0.268 |  |
|  |  | 109 | MR-PRESSO test | 0.99(0.96-1.02) | 0.594 |  |
|  |  | 109 | MR-Egger | \ | 0.199* |  |
|  | Genus *LachnospiraceaeUCG001* |  |  |  |  |  |
|  |  | 109 | Inverse-variance weighted | 1.01(0.98-1.04) | 0.473 |  |
|  |  | 109 | Weighted median | 1.01(0.98-1.05) | 0.533 |  |
|  |  | 109 | MR-PRESSO test | 1.01(0.98-1.04) | 0.475 |  |
|  |  | 109 | MR-Egger |  | 0.008 |  |
|  | Genus *Oxalobacter* |  |  |  |  |  |
|  |  | 109 | Inverse-variance weighted | 1.01(0.98-1.05) | 0.405 |  |
|  |  | 109 | Weighted median | 1.03(0.98-1.09) | 0.279 |  |
|  |  | 109 | MR-PRESSO test | 1.01(0.98-1.05) | 0.407 |  |
|  |  | 109 | MR-Egger | \ | 0.781* |  |
|  | Genus *RuminococcaceaeUCG014* |  |  |  |  |  |
|  |  | 109 | Inverse-variance weighted | 0.99(0.97-1.01) | 0.338 |  |
|  |  | 109 | Weighted median | 0.98(0.95-1.01) | 0.173 |  |
|  |  | 109 | MR-PRESSO test | 0.99(0.97-1.01) | 0.279 |  |
|  |  | 109 | MR-Egger | \ | 0.189* |  |

Abbreviations: CD, crohn's disease; CI, confidence interval; IBD, inflammatory bowel disease; MR, Mendelian randomization; MR-PRESSO test, MR Pleiotropy RESidual Sum and Outlier test; OR, odds ratio; SNP, single nucleotide polymorphism. **P*-value of the intercept from MR-Egger regression analysis
